# Genome-driven integrated classification of breast cancer validated in over 7,500 samples

**DOI:** 10.1186/s13059-014-0431-1

**Published:** 2014-08-28

**Authors:** H Raza Ali, Oscar M Rueda, Suet-Feung Chin, Christina Curtis, Mark J Dunning, Samuel AJR Aparicio, Carlos Caldas

**Affiliations:** Cancer Research UK Cambridge Institute, University of Cambridge, Li Ka Shing Centre, Robinson Way, CB2 0RE Cambridge, UK; Department of Pathology, University of Cambridge, Tennis Court Road, CB2 1QP Cambridge, UK; Department of Oncology, University of Cambridge, Addenbrooke’s Hospital, Hills Road, CB2 0QQ Cambridge, UK; Cambridge Experimental Cancer Medicine Centre and NIHR Cambridge Biomedical, Research Centre, Cambridge University Hospitals NHS, Hills Road, CB2 0QQ Cambridge, UK; Keck School of Medicine, University of Southern California, CA 90033 California, USA; Department of Molecular Oncology, British Columbia Cancer Research Centre, Vancouver, V5Z 1L3 British Columbia, Canada

## Abstract

**Background:**

IntClust is a classification of breast cancer comprising 10 subtypes based on molecular drivers identified through the integration of genomic and transcriptomic data from 1,000 breast tumors and validated in a further 1,000. We present a reliable method for subtyping breast tumors into the IntClust subtypes based on gene expression and demonstrate the clinical and biological validity of the IntClust classification.

**Results:**

We developed a gene expression-based approach for classifying breast tumors into the ten IntClust subtypes by using the ensemble profile of the index discovery dataset. We evaluate this approach in 983 independent samples for which the combined copy-number and gene expression IntClust classification was available. Only 24 samples are discordantly classified. Next, we compile a consolidated external dataset composed of a further 7,544 breast tumors. We use our approach to classify all samples into the IntClust subtypes. All ten subtypes are observable in most studies at comparable frequencies. The IntClust subtypes are significantly associated with relapse-free survival and recapitulate patterns of survival observed previously. In studies of neo-adjuvant chemotherapy, IntClust reveals distinct patterns of chemosensitivity. Finally, patterns of expression of genomic drivers reported by TCGA (The Cancer Genome Atlas) are better explained by IntClust as compared to the PAM50 classifier.

**Conclusions:**

IntClust subtypes are reproducible in a large meta-analysis, show clinical validity and best capture variation in genomic drivers. IntClust is a driver-based breast cancer classification and is likely to become increasingly relevant as more targeted biological therapies become available.

**Electronic supplementary material:**

The online version of this article (doi:10.1186/s13059-014-0431-1) contains supplementary material, which is available to authorized users.

## Background

The classification of breast tumors based on morphology (histological type and grade) and two key markers, estrogen receptor (ER) and human epidermal growth factor receptor 2 (HER2), remains the mainstay of current clinical practice. Early attempts to improve this situation by using genomic technology focused on data-driven methods including unsupervised transcriptome-based classification [1-3] and gene signatures trained against a specific clinical outcome [4-6]. However, this approach is not based on the underlying molecular changes which ultimately constitute a tumor’s oncogenic drive. More recent genomic studies have begun to reveal the complexity of the landscape of somatic alterations in breast cancer at the levels of mutations and copy number alterations (CNAs) [7-12]. The strategy for discriminating between driver and passenger events amongst these somatic alterations has, for non-synonymous mutations, focused on identification of genes more frequently mutated than expected by chance in a given collection of tumor samples. Although this approach has required some adjustment owing to the non-random background mutation rates in cancer genomes [13] and may be complemented by accounting for the pattern of mutational distribution within genes [14], it does provide a roadmap for the comprehensive identification of all driver mutations if a sufficiently large sample size is interrogated [15]. In the case of CNAs, an additional strategy has been to integrate genomic and transcriptomic data in order to identify areas of recurrent alteration associated with deregulated gene expression (expression quantitative trait loci (eQTLs)) [16-18]. Importantly, the balance between somatic mutations and alterations in copy number has been investigated as part of the The Cancer Genome Atlas (TCGA) pan-cancer analysis of 12 tumor types [19]. Investigation of a shortlist of ‘selected functional events’ revealed an approximately inverse relationship between mutation and CNAs with some tumor types dominated by mutations deemed ‘M-class’ (for example, renal cell carcinoma and colorectal adenocarcinoma), while others were dominated by CNAs deemed ‘C-class’ [19]. Prototypical ‘C-class’ tumor types were ovarian and breast cancer. This analysis highlights the need for a classification scheme based on the pattern of somatic driver alterations in a particular tumor, which, in the case of breast tumors, is dominated by CNAs. Using the largest sample collection with extensive genomic, transcriptomic and clinical annotation in existence, we previously described a scheme for classifying breast tumors into 10 subtypes based on the pattern of CNAs which exert a concordant effect on gene expression in *cis* (eQTLs). This classification was named IntClust owing to the clustering of tumors based on the integration of genomic and transcriptomic data [20] to find probable driver events [17]. The scheme remains the only genome-wide driver-based classification of breast cancer that reconciles tumor genomes with their transcriptomes and, as such, has significant potential for rational patient stratification [21]. Further validation of the clinical and biological significance of this approach requires a reliable method to subtype tumors in independent cohorts assayed on different platforms. This is, in part, due to the relative scarcity of studies for which both high-resolution copy-number and transcriptomic data are available, since the classification requires both data types. Here, we have overcome this hurdle by developing a flexible method for tumor subtyping which only requires gene expression data and is not limited to specific platforms. This gene expression-based classifier has enabled us to investigate the IntClust classification in the numerous translational studies for which transcriptomic and clinical data are publically available. Here, we report on the reproducibility of IntClust subtypes, their clinical validity and the extent to which they capture the landscape of somatic driver alterations in breast cancer using these external independent studies.

## Results

### Characteristic gene-expression profiles for assignment to IntClust subtype

**Figure 1:**
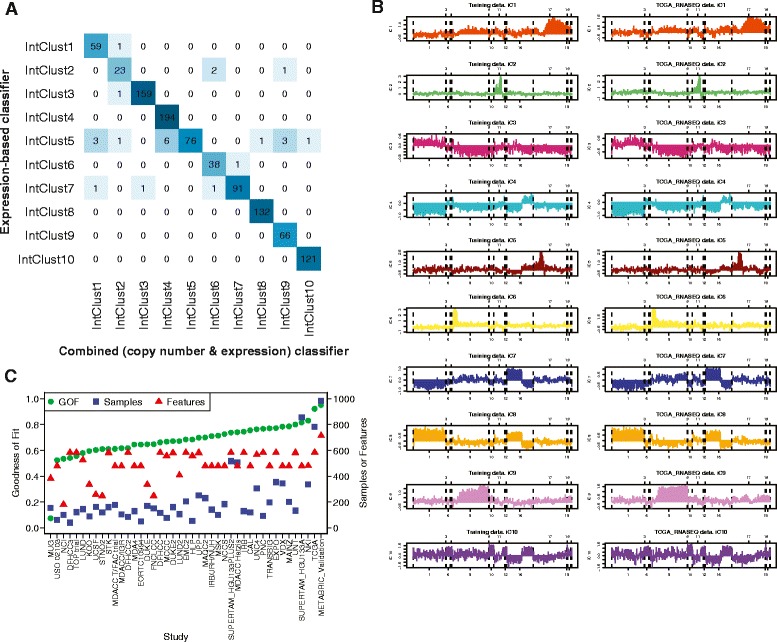
**Reproducible IntClust gene expression profiles enable accurate classification. (A)** Cross-tabulation of IntClust subtypes classified according to the combined (copy number and gene expression) classifier and the expression-based classifier in the METABRIC validation dataset (N = 983). Intensity of box colors is proportional to the depicted value. **(B)** Comparison of average gene-expression profiles for all 10 IntClust groups in the METABRIC discovery set (left) and TCGA samples (right). The x-axis is genomic position and the y-axis is z-score log2-normalised gene expression level. **(C)** Scatter plot of the goodness of fit, number of samples and number of available features for expression-based IntClust classification by each study. GOF, goodness of fit.

We used the dataset in which the IntClust subtypes were originally discovered (N = 997) to train a gene expression-based classifier. The selected genes corresponded to particular *cis* eQTLs which were in the original clustering algorithm [17]. A panel of 612 genes (some represented by more than one probe) were used for subtype assignment. They represent all gene expression features identified using integrative clustering [20] in the original study [17]. Based on these 612 genes, characteristic patterns of expression observed between subtypes provided a template by which new samples could be classified using Prediction Analysis of Microarrays (PAM) software [22]. This method was designed to account for differences in platform and includes some redundancy such that it can accommodate missing genes by retraining the algorithm against the index dataset for optimal subtype assignment. This is achieved by re-estimation of centroids for each of the 10 clusters by comparison to the METABRIC discovery dataset based on the available feature (gene) set in a particular study. These newly estimated centroids are then used for subtype assignment. In order to evaluate the accuracy of this classifier we applied it to the samples of the original IntClust validation study (N = 983). These samples had previously been classified using the combined feature set of a combination of gene expression (Illumina HT-12 v3 platform) and copy number (Affy SNP 6.0 arrays). Assignment based on the expression classifier was concordant with combined CNA-gene expression classification in 98% of samples (Figure 1A), demonstrating the efficacy of the approach. We also evaluated the influence of using all 714 probes (some genes were represented by more than one probe) compared to 612 genes (each represented by one probe) using samples from the METABRIC validation study. These data, depicted as a cross-tabulation in Additional file 1, show that 94.7% of 983 samples were concordantly classified. We applied this expression-based method to external independent datasets available in public repositories (Additional file 2) on a study-by-study basis, which in total included 7,544 breast tumors. We found that the characteristic patterns of gene expression were highly reproducible within the majority of studies. Figure 1B illustrates the characteristic gene expression profiles of the features used for IntClust classification by each subtype for both the index dataset and, for comparison, RNA-seq samples from the TCGA breast cancer marker paper [8] classified using our method. The depicted profiles represent an average of all samples within a particular subtype. In order to confirm that the gene expression profile of each IntClust subtype was underpinned by characteristic CNAs, we plotted the copy number profiles of the TCGA samples which had been assigned an IntClust subtype based on gene expression (Additional file 3). These subtype CNA profiles were similar to those in the original METABRIC study (Additional file 3). Correlation statistics between copy number profiles of METABRIC and TCGA samples within IntClust subtypes were computed and are presented in Additional file 4. These correlations between TCGA samples within one IntClust group compared to all METABRIC IntClust groups consistently show that the highest correlation was between samples of the same IntClust subtype.

In order to quantify the efficacy of our method by study, we used a correlation statistic to estimate the goodness of fit of the classification model where a score of 1.0 indicates perfect correlation between the gene expression profiles of new samples and those contained within the index dataset. Figure 1C depicts the correlation (goodness of fit) statistics, number of samples and number of features (of a possible 714) for every study. This comparison of average gene expression profiles by subtype indicates a striking conservation of patterns across studies with the average correlation being 0.69. The highest correlation of 0.95 was, as expected, associated with the METABRIC validation dataset. The next highest correlation of 0.92 related to RNA-seq samples from TCGA. The lowest correlation was a significant outlier among studies at 0.1. Although it was not possible to definitively determine the basis for this poor correlation, we note that the distribution of *ESR1* and *ERBB2* expression was not bimodal for this study and, in general, there appeared to be a low signal-to-noise ratio. The Pearson’s correlation coefficient between goodness of fit and number of samples per study was 0.53 and between goodness of fit and number of features per study was 0.38. As a comparator, we also classified samples into the ‘intrinsic subtypes’ using the PAM50 classifier [23] and into four molecular subtypes based on three genes (*ESR1*, *ERBB2* and *AURKA*) using the SCMGENE classifier [24]. We evaluated the effect of platform variability on subtype assignment by using 475 samples from the TCGA study for which gene expression data had been collected using both RNA-seq and microarrays. Cross-tabulations of subtype assignment with Kappa-agreement statistics, by data type (RNA-seq or microarray) for each of the three classifiers (SCMGENE, PAM50 and IntClust) are presented in Additional file 5. The agreement between classifiers was 93.1% for SCMGENE, 93.7% for PAM50 and 81.3% for IntClust. It should be noted that the number of possible classes significantly influences the rate of concordance for a classification. The expected agreement by chance alone for SCMGENE (four groups) was 29.8%, for PAM50 (five groups) was 33.2% while for IntClust (ten groups) was 12.0%. Similarly, when interpreting the importance of discordantly classified cases, the number of possible classes should be taken into account since the relative difference between classes is likely to be smaller for a classification comprising a larger number of possible groups.

We also applied our classifier to a large panel of cell lines from two data repositories (Sanger COSMIC database and the Cancer Cell Lines Encyclopedia (CCLE)). We applied three versions of our classifier to these data: copy number data alone, gene expression alone and the combined copy number/gene expression feature set. The goodness of fit statistics for these classifiers are depicted in a scatter plot in Additional file 6. Overall, the copy number-based classifier performed better than the expression-based or combined classifier. The ensemble goodness of fit for the copy number-based classifier was 0.74 using the Sanger dataset and 0.75 using the CCLE dataset, compared with the ensemble average goodness of fit for the expression-based classifier, which was 0.47 using the Sanger dataset and 0.62 using the CCLE dataset. These differences may be due to variation in culture conditions and passages, which are more likely to be reflected in gene expression than in CNAs. Weighted scatterplots depicting cell line classification according to classifier type and by dataset are presented in Additional file 6. Similarly, comparison of classification between PAM50 and SCMGENE datasets are depicted in Additional file 7. There was considerable variability in subtype assignment for cell lines according to the origin of the data for all classifiers. This highlights the challenge of reliable cell line classification, which is likely due to drift over time and variability in cell culture conditions. Our findings show that, on average, copy-number profiles of cell lines are more similar to primary tumors than gene expression profiles and ought to be preferentially used for their classification into molecular subtypes. Details of molecular subtype assignment for each cell line by data source are presented in Additional file 8.

### IntClust subtypes are reproducible entities observable across studies

**Figure 2:**
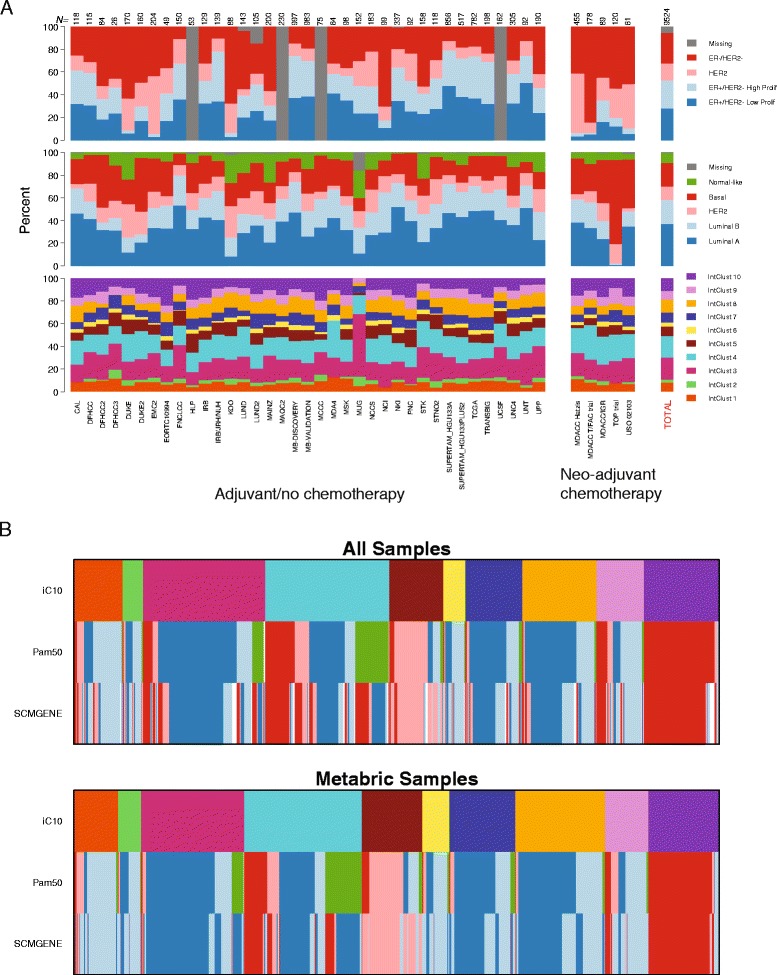
**Distribution of IntClust and transcriptome-based subtypes by study. (A)** Bar charts depicting the proportion of samples that belong to each subtype for IntClust (bottom panel), PAM50-based (middle panel), and SCMGENE-based (top panel) classification by study. The total number of samples in each study (N) is depicted at the top of the bars. **(B)** Bar charts depicting the relative proportions of PAM50 and SCMGENE subtypes within IntClust subtypes, separately for the METABRIC and external studies.

The platform and feature flexibility of our classifier enabled the classification of a large collection of independent samples. For comparison we also classified tumors into the ‘intrinsic’ subtypes using the PAM50 and SCMGENE classifiers [23,24]. The relative proportions of the 10 IntClust subtypes were similar across studies, including the CNA-devoid IntClust 4 group (Figure 2A) where the relative proportion ranged from 33% in the MDA4 study to 11% in the MCCC study, and all 10 subtypes could be identified in all but 6 of 42 studies. In three of these six studies, all ten subtypes except IntClust 2 could be identified. This is not surprising since in the original METABRIC study IntClust 2 is the least frequent of the 10 subtypes, comprising just 4.5% of tumors in the discovery dataset.

A subset of patients in some of the studies received neo-adjuvant (before definitive surgery) chemotherapy, and tissue would have been derived from biopsies or fine needle aspirates. Here, we note that based even on these samples, IntClust subtype could be reliably assigned and resulted in proportions comparable to those from studies in patients who did not receive neo-adjuvant chemotherapy (Figure 2A). This implies that it is possible to reliably assign tumors to IntClust subtypes based on biopsy material alone as might be undertaken in clinical practice. Overall, similar proportions of each of the 10 subtypes were found in external studies in comparison to the METABRIC reference study (Figure 2B). Moreover, the relative composition of each IntClust subtype in terms of the proportion of different ‘intrinsic’ subtypes that comprised it was very similar between the METABRIC study and external samples (Figure 2B). The inverse of the plot in Figure 2B, depicting the IntClust subtype composition of each of the ‘intrinsic’ subtypes classified according to PAM50 and SCMGENE is presented in Additional file 9.

### IntClust subtypes are associated with reproducible survival patterns

**Figure 3:**
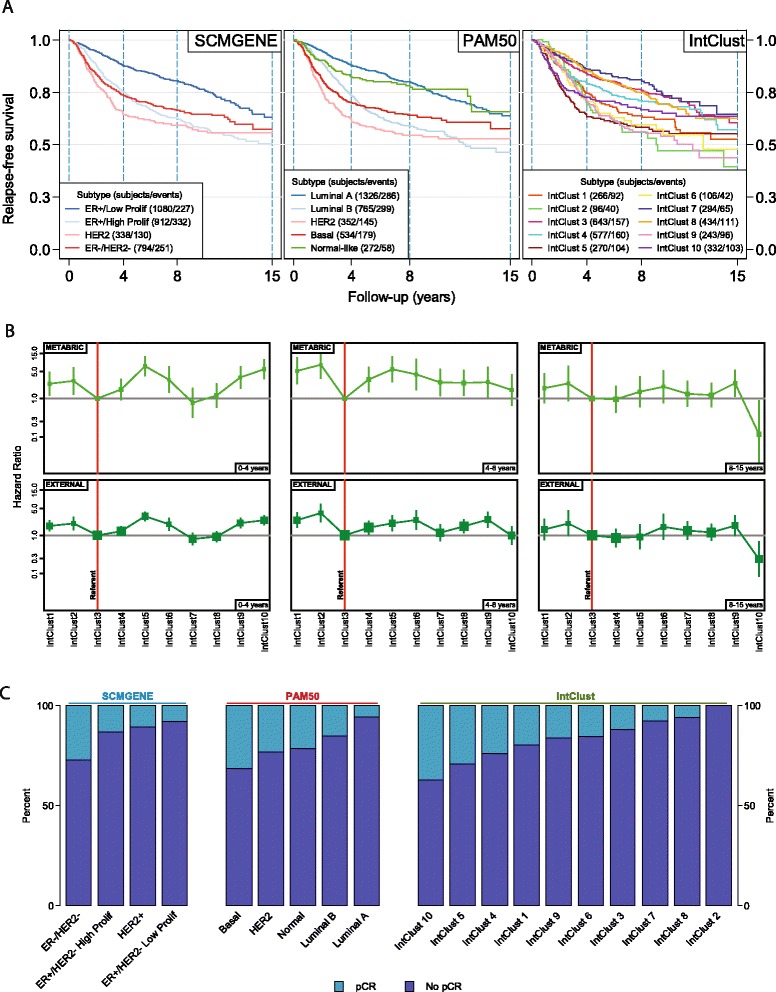
**Association between subtype and clinical outcome. (A)** Survival plots by subtype for external studies with available time-to-event data. The METABRIC study is excluded. **(B)** Comparison of univariable hazard ratios (boxes) and 95% confidence intervals (vertical lines) for IntClust subtypes, taking IntClust 3 as referent, for each of three follow-up brackets (0 to 4 years, 4 to 8 years, 8 to 15 years) separately for cases in the METABRIC study and those in external studies. Box sizes are weighted according to sample size. **(C)** Bar-charts depicting the proportion of tumors that underwent pathological complete response (pCR) by molecular subtype in all external studies of neo-adjuvant chemotherapy.

One important measure of a novel method for disease classification is the degree to which subtypes show an association with clinical outcome. Here, we have undertaken an extensive comparative analysis of the PAM50, SCMGENE and IntClust classifiers. Figure 3A depicts relapse-free survival plots of subtypes by all three classifiers for all cases with available data from external studies (cases from the METABRIC study have been excluded). Patterns of survival of the IntClust subtypes in these independent cases are similar to those in the original METABRIC study (Additional file 10). To assess this formally we conducted a comparative analysis of the hazard associated with each IntClust subtype in METABRIC (against death from breast cancer) and all external studies (against relapse-free survival). Figure 3B depicts hazard ratios of IntClust subtypes, taking IntClust 3 as the referent, for each of three brackets of follow-up time (0 to 4 years, 4 to 8 years, and 8 to 15 years) for patients in the METABRIC study and patients in external studies separately. Patterns of relative hazard by IntClust subtype observed in the METABRIC study were reproduced in external studies in each of the three follow-up brackets. For example, IntClust 1 and IntClust 2 were consistently associated with increased hazard with a slightly higher hazard ratio for IntClust 2 compared with IntClust 1 consistently between METABRIC and external studies. Changing patterns of hazard are also well illustrated by this analysis, particularly the qualitative shift in hazard associated with IntClust 10, which, again, is reproduced in external studies.

In order to evaluate the relative contribution of each classifier to the prediction of relapse-free survival, we compared the discrimination of survival prediction models. These models comprised the molecular (SCMGENE, PAM50, IntClust) subtypes as categorical variables and were adjusted for tumor size (<1, 1 to 2, 2 to 3, 3 to 5, >5 cm), node status (negative versus positive) and histological grade (1, 2 or 3). The coefficients for these models were derived using Cox-regression in the METABRIC dataset and then applied to external studies with available data in order to avoid over-optimistic estimates. Harrell’s C-index was used to estimate the relative discrimination of models where an index of 1 reflects perfect discrimination between high and low risk patients while an index of less than 0.5 reflects discrimination which is no better than chance. We conducted analyses separately by ER status and within three brackets of follow-up time (0 to 4, 4 to 8 and 8 to 15 years) in order to account for violations of Cox-proportional hazards assumption [25] and to estimate differences in model performance for short- versus long-term survival prediction. Additional file 11 depicts the results of these analyses. In general, the performance of all three models was significantly better in ER-positive breast cancer, particularly during the first 5 years of follow-up, compared with ER-negative disease. The relative performance of the three models was comparable in both ER-positive and ER-negative breast cancer. Both IntClust (*P* = 0.005) and SCMGENE (*P* = 0.03) significantly outperformed PAM50 in the prediction of late events (8 to 15 years) in ER-positive breast cancer (Additional file 11). However, it should be noted that, particularly for late events (81 events in ER-positive disease), these analyses may be underpowered and, as a consequence, preclude robust conclusions being drawn. These analyses show that the IntClust classifier performs at least as well as transcriptome-based classification in the prediction of relapse-free survival.

### IntClust subtypes show large differences in chemosensitivity

A second determinant of the relative utility of a disease classification scheme is whether differences in chemosensitivity are reflected in different subtypes. In order to investigate this, we used a collection of breast cancer studies where patients had received neo-adjuvant cytotoxic chemotherapy [26-29] and for whom data on pathological complete response (pCR) were available (N = 871). A tumor is said to have undergone pCR if, following surgery, no residual tumor cells remain upon pathological examination. pCR has been shown to be a powerful predictor of long-term survival [30]. Distinct patterns of pCR between molecular subtypes of breast cancer have been reported previously, with the highest rates observed in ER-negative tumors and the lowest in ER-positive HER2-negative tumors [31]. Similarly, distinct patterns of pCR were observed by molecular subtype (Figure 3C). The highest rates of pCR by IntClust subtyping were observed within the IntClust 10 subtype at 37% (45/121) compared with the highest rate by PAM50 classification within the basal-like subtype at 31% (101/322) and the highest rate by SCMGENE classification within the ER-/HER2- subtype at 27% (125/463). The lowest rates of pCR by IntClust subtyping were observed within the IntClust 2 subtype at 0% (0/20) compared with the lowest rate by PAM50 classification within the luminal A subtype at 6% (15/265) and the lowest rate by SCMGENE classification within the ER+/HER2-, low proliferation subtype at 8% (4/51). We next conducted a formal comparison of the relative value of each classifier in predicting pCR after adjustment for clinical variables (tumor and lymph node stage and histological grade). We evaluated the discrimination of prediction models using the area under the curve (AUC) from a receiver operating characteristic (ROC) analysis. Odds ratios were based on a logistic-regression model again derived from the largest external study (N = 435) [29] and subsequently tested in the remaining data (N = 436) in order to avoid over-optimistic estimates. The performance of the three models was very similar and not significantly different, with SCMGENE classification returning an AUC of 0.64 (95% confidence interval (CI) 0.56 to 0.72 PAM50 classification returning an AUC of 0.67 (95% CI 0.60 to 0.75), while the IntClust classifier returned an AUC of 0.66 (95% CI 0.58 to 0.74) (Additional file 11). These data show that IntClust is as accurate a predictor of pCR to cytotoxic chemotherapy as PAM50 or SCMGENE classification.

### Breast cancer genomic drivers are best represented by IntClust subtypes

**Figure 4:**
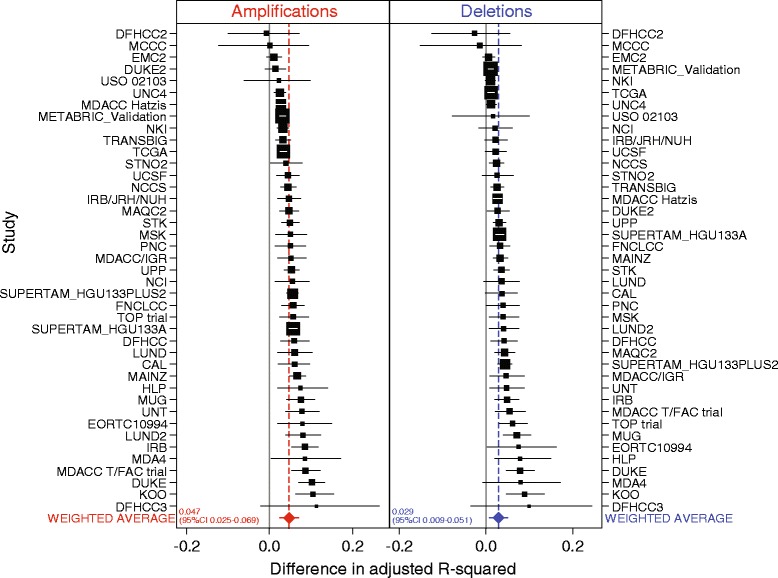
**Explained variation in gene expression levels of genes contained within TCGA-defined regions of recurrent copy number alteration in breast cancer.** Forest plots of the average differences in adjusted R-squared statistics between classifiers (IntClust and PAM50) by study according to genes within loci recurrently amplified (red) or deleted (blue) in breast cancer. Boxes represent point estimates where box size is weighted according to study sample size and horizontal lines depict 95% CIs. Point estimates and confidence intervals are based on bootstrap resampling of 1,000 replicates. Diamonds depict the weighted average difference.

We next investigated the extent to which copy number-driven breast cancer genes are captured by the IntClust classification compared with PAM50 or SCMGENE classification. We used an independent list of copy number aberrations which were reported by TCGA as occurring recurrently in breast cancer [8]. We determined the degree to which the variation in expression of genes contained within these regions of CNA (Additional file 12) is explained by molecular subtype using data from all external studies (excluding the METABRIC discovery study). In a one-way analysis of variance (ANOVA) we took gene expression as the dependent variable and molecular subtype as independent variables. The explained variation in gene expression by molecular subtype was estimated using an adjusted R-squared statistic within each study. This was conducted separately for genes contained within regions of amplification (N = 409) and deletion (N = 3,485). An average adjusted R-squared statistic was computed for each study. These statistics represent the average explained variation of gene expression for every amplified or deleted gene per study. In order to determine whether IntClust or PAM50 classification better explained these patterns of gene expression, we subtracted the average R-squared for IntClust from that of PAM50 per study. Figure 4 depicts the average of these differences for amplified and deleted genes where a positive value denotes that, on average, variation in gene expression is better explained by IntClust and a negative value denotes better explanation by transcriptome-based (PAM50) classification. We calculated 95% CIs using the percentile method based on bootstrap resampling of 1,000 replicates. The diamonds depict the average across all studies weighted by study size. In both amplified and deleted genes, the weighted average is a positive value (0.05 for amplified genes and 0.03 for deleted genes), indicating that variation in gene expression is significantly better explained by IntClust. Similarly, IntClust better explained patterns of expression for these genes than SCMGENE, as detailed in Additional file 13. A ranked list of the top 50 amplified and the top 50 deleted genes explained by IntClust and those better explained by IntClust than PAM50 with their R-squared values is depicted in Additional file 14. Box plots depicting the distribution of expression by IntClust subtype for the top 50 genes explained by IntClust are provided as Additional file 15.

## Discussion

The landscape of somatic alterations in breast cancer is complex and heterogeneous. This variety is reflected in the diverse clinical behavior of breast tumors and provides critical insight for the development of rational therapies. Therefore, a method for capturing this complexity which can be readily implemented in a clinical setting is urgently required. We have extensively investigated the potential of the IntClust classification to meet this need, in terms of its reproducibility, association with clinical outcome and representation of copy number-driven cancer genes. We find that IntClust subtypes are observable across studies, are significantly associated with clinical outcome and best capture the repertoire of breast cancer genomic drivers. These data provide a compelling rationale for IntClust as a driver-based molecular taxonomy with considerable potential for clinical application. Indeed, a recent clinical trial (SAFIR01) shows that CNAs are the drivers for which targeted therapies are most frequently identified in breast cancer [32].

IntClust subtypes were observed across studies at comparable frequencies. This important observation demonstrates that these entities are reproducible and represent true breast tumor subtypes. The discovery study used for identifying the IntClust groups comprised 997 tumors from five centers spanning two continents [17]. This approach was adopted in order to accrue a sufficient sample size representative of the whole of the breast cancer population. Therefore, a robust classifier of IntClust subtypes should identify these groups in external studies, just as we have observed. We also note that *TP53*, one of the two most frequently mutated genes in breast cancer, is mutated at comparable frequencies across IntClust subtypes in both METABRIC and TCGA [33].

The clinical validity of the IntClust subtypes has here been demonstrated by their association with relapse-free survival and propensity to undergo pCR in studies of neo-adjuvant chemotherapy. An important observation was the recapitulation of survival patterns originally observed in the METABRIC study [17]. This shows that the IntClust subtypes are biologically distinct, readily discernible entities associated with widely variable but predictable clinical behavior. We compared the performance of prediction models which contained either transcriptome-based or IntClust subtypes in their ability to discriminate between patients at higher versus lower risk of disease relapse or resistance to chemotherapy. These models performed similarly. Since the IntClust subtypes were conceived with the intention of best representing breast tumor biology as defined by the genome, survival was not taken into account [17]. It should, however, be noted that an association with survival is not the sole arbiter of the validity of a biological classification. Data-driven approaches designed to generate models for risk stratification of breast cancer patients have largely uncovered proliferation-related genes which, while they are indisputably effective predictors of survival, do not provide additional insight into the biology underlying their expression [34]. Equally, an example of an important disease entity which does not significantly improve prediction of survival is lobular breast carcinoma. Patients with these tumors, which are characterized by single-file morphology and loss of E-cadherin expression, have been convincingly shown to experience patterns of survival indistinguishable from patients with the more common invasive ductal carcinoma [35], yet the diagnosis of lobular carcinoma is routine, critical for appropriate long-term clinical management and highlights a patient subgroup potentially amenable to novel targeted therapies. A comparable example concerns the distinction between IntClust 2 and IntClust 1. IntClust 2 tumors are characterized by amplification of 11q13/14 encompassing *CCND1*, *EMSY* [36] and *PAK1*, whereas IntClust 1 tumors harbor 17q23 amplification encompassing *RPS6KB1*, *PPM1D*, *PTRH2* and *APPBP2* [17]. Both subgroups comprise high-risk, mostly ER-positive tumors. The unadjusted 10 year relapse-free survival observed in external studies was 64% for patients with IntClust 1 tumors and 49% for patients with IntClust 2 tumors. However, no tumors in the IntClust 2 subtype underwent pCR (0/20) whereas tumors in the IntClust 1 subtype showed the fourth highest rates of pCR at 20% (15/76). Although these observations require validation, they suggest that in spite of a similar aggressive clinical course, IntClust 2 tumors are chemoresistant in comparison to IntClust 1 tumors. This difference, highlighted by IntClust subtyping and likely attributable to differences in amplification-driven oncogenes, is worthy of further investigation. Here, IntClust 2 tumors represented just 3.1% (298/9,524) of patients; nonetheless, this group experiences some of the poorest survival of all subgroups. This dismal prognosis may, in part, be explained by our observation that IntClust 2 tumors are entirely chemoresistant. These patients warrant consideration of alternative therapeutic modalities and represent a priority for the development of novel targeted therapies. This subtype is not identified by any other breast cancer classification scheme. Such observations highlight the important benefits of rational tumor classification based on molecular drivers.

Based on an independent list of recurrent CNAs in breast cancer and using samples compiled from external studies [8], we found that the IntClust classification best explains expression levels of genes which fall within these loci. This finding reiterates the nature of IntClust as a biological classification which explains characteristic gene expression profiles in terms of their genomic drivers. We have conducted an unbiased comparison by including all genes that fall within loci reported as recurrently altered by an independent group (TCGA); however, it should be noted that the magnitude of explained variation differed greatly between genes (Additional file 16). The explained variation of a large proportion of genes showed little difference between classifiers whereas a subset showed large differences (Additional file 16). This is likely due to the fact that the majority of genes included within these loci are passengers which do not confer a growth advantage to proliferating tumor cells. Somatic CNAs are a relatively common event among breast cancer genomes and a long-standing problem has been to identify genes which amount to drivers within recurrently altered genomic loci. Although criteria for their characterization have been proposed [37], particularly for amplified genes, they stipulate multiple lines of independent evidence which require considerable resources and, as such, have not been generated for most loci. Moreover, it is possible that in some instances where a minimal region of amplification contains more than one gene, such as the 11q13/14 locus which defines IntClust 2, that adjacent genes may act in a concerted manner to confer a selective growth advantage just as has been observed in lung cancer [38]. The conception of IntClust was pragmatic in attempting to minimize the influence of passenger genes. Three strategies were employed to this end. First, the discovery study was large (997 samples), enabling reliable identification of regions of recurrent CNA. Second, only the top 1,000 *cis* eQTLs were included for classification based on the strength of association between alteration in copy number and levels of gene expression. Third, clustering retained only those features which contributed to the separation of tumors into distinct subgroups (754 features) [17]. This approach provides the most definitive scheme for breast tumor classification based on the pattern of copy number-driven genes. It is likely, therefore, that our unbiased comparison of explained variation in the expression of genes within recurrent CNAs underestimates the extent to which IntClust reflects the expression of genomic drivers within these regions. Nonetheless, our analysis does demonstrate that IntClust best captures variation in levels of gene expression of copy number-driven breast cancer genes.

## Conclusions

We have developed an expression-based method for classification of breast tumors into the IntClust subtypes. We used this method and public datasets of breast tumor transcriptomes to investigate the validity of IntClust. We confirmed that the IntClust subtypes are reproducible entities, demonstrated their association with clinical outcome and found that IntClust best captures expression patterns of breast cancer drivers. Our method is a powerful tool for independent researchers to investigate the significance of IntClusters. Moreover, our findings highlight the potential of IntClust in the era of targeted therapies. Our classifier lays the foundation for the generation of a clinical test to assign tumors to IntClust subtypes.

## Materials and methods

### Development of the IntClust expression-based classifier

We modified the method for IntClust classification which was originally reported for subtype validation [17]. Probes were re-annotated to hg19 and some eliminated because of ambiguous genomic matching (where a probe sequence matched to more than one position in the reference genome). Some genes were represented by more than one probe, reflecting the design of the Illumina beadarray ht12v3 microarray, in which probes can represent different parts of a gene. Our method followed three steps in classifying a new set of samples. In the first step features were matched. Copy number features were matched either by genomic position or gene name, while expression features were matched by probe name (METABRIC study) or gene name. This was performed by the function matchFeatures. In the second step data were normalized to the distribution of the METABRIC discovery set. We scaled each gene to a z-score. This was achieved using the normalizeFeatures function. The function also implements other normalization methods from the CONOR R package [39]. In the third step a classifier was trained using the probes that were matched using the pamr R package [22], based on shrunken centroids. The optimal threshold was chosen by cross-validation, so different runs produced slightly different classifications unless we set a random number seed. That is, centroids were re-estimated based on the features available in different platforms against the METABRIC discovery dataset for each of the 10 clusters. The iC10 function was used for this step.

Several quality statistics were included as part of our method for inspection of results. A goodness of fit, which was a Pearson correlation coefficient, was computed. It represented the correlation between the average (across all samples) gene expression profile for each cohort and the centroids from the training data set, within each IntClust subtype for those genes where data were available in the external study. In short, the statistic represents a measure of the similarity, in terms of gene expression, of IntClust subtypes from external studies compared with the training data set. We plotted centroids in order to inspect their representation within each subtype in the test dataset - several functions are included in the iC10 package to achieve this. We have made our method freely available for download as an R package under the name 'iC10' at CRAN [40].

We applied this method to breast cancer gene-expression datasets available in public repositories. A large proportion of these studies had previously been compiled and curated by Haibe-Kains *et al*. [24] and we downloaded these data directly from the authors’ website [24]. Additional details, including Gene Expression Omnibus (GEO) accession numbers of included studies are detailed in Additional file 2. It is possible that data for some patients have been uploaded more than once, particularly if those patients participated in more than one study. We took three precautions against inadvertent inclusion of replicate records in our analyses: 1) only cases with a unique GEO identifier were retained; 2) cases identified by Haibe-Kains *et al*. as replicates were removed; and 3) cases identified by the doppelgangR package [41] as replicates based on highly correlated gene expression profiles were further investigated. Those cases which, in addition to correlated gene expression, also showed concordant values for tumor stage, node stage, histological grade and, in the case of neo-adjuvant studies, pCR were also removed. Cases identified as probable replicates by this strategy almost exactly overlapped with those annotated as replicates by Haibe-Kains *et al*. with only an additional three cases being removed. For each dataset, the iC10 package was run with expression data only (using probe names for the METABRIC study and gene names for the rest) and normalizing each probe to a z-score ('scale' option in the function normalizeFeatures). PAM50 classification was conducted accounting for imbalances in ER status, as defined in [17]. SCMGENE classification was conducted using the genefu package in R, available at Bioconductor.

In order to classify breast cancer cell lines, we used copy number and gene expression data from two collections of cell lines: Sanger COSMIC database and CCLE. Copy number data from the COSMIC database consisted of segmented copy number calls. The CCLE database provided copy number data on 579 genes (optimal IntClust classification requires 612 genes) as the summarized log ratio for each gene. Nevertheless, the fit of the IntClust classifier based on copy number was similar for both datasets (0.74 for COSMIC and 0.75 for CCLE). We noted that some cell lines are characterized by copy number amplification of both *ERBB2* (IntClust 5) and 8q24 (IntClust 9), which contains the *MYC* oncogene. In these cases the classifier mostly assigned an IntClust 9 subtype (HCC1419, HCC1569, MDA-MB-453, OCUB-M, ZR-75-30). As a comparison, 10% (28/268) of primary tumors with amplification of *ERBB2* also showed co-amplification of *MYC* in 1,980 samples from the METABRIC study. Cell lines were also classified in IntClust subtypes based on gene expression alone and combined copy number/gene expression and into PAM50 and SCMGENE subtypes based on gene expression alone.

### Statistical analysis of the association between subtype and clinical outcome

Associations between subtype and survival were estimated using Cox regression. Of the studies with available time-to-event data, relapse-free survival was available for some and distant metastasis-free survival for others. Our survival time variable comprised relapse-free survival but where this was unavailable distant metastasis-free survival was used.

Comparison of univariable hazard ratios associated with IntClust subtype between the METABRIC (disease-specific survival) and external studies (relapse-free survival) (Figure 3B) was conducted by using IntClust 3 as the referent class, separately for three brackets of follow-up time (0 to 4, 4 to 8 and 8 to 15 years).

Performance of predictive models was assessed as follows: Cox regression models which contained either PAM50 or IntClust as a categorical variable and were adjusted for tumor size (<1, 1 to 2, 2 to 3, 3 to 5, >5 cm), node status (negative versus positive) and histological grade (1, 2 or 3) as continuous variables were fit within the METABRIC study (the largest study) against available time-to-event data (disease-specific survival). These models were stratified by each of the five centers of the METABRIC consortium. Separate models were fit for ER-positive and ER-negative breast cancer within three time brackets (0 to 4, 4 to 8 and 8 to 15 years) in order to investigate differences in model performance in short- versus long-term survival and to account for violations of the proportional hazards assumption. The coefficients derived from these models were then applied to external studies with available data. Comparison of model discrimination in this test population was conducted using the method suggested by Newson [42] using Harrell’s C-index implemented using the somersd and lincom commands in Stata [42].

Associations between subtype and pCR were estimated using logistic regression. Logistic regression models comprising either PAM50 or IntClust as categorical variables and adjusted for tumor size (T-stage), positive lymph nodes (N-stage) and histological grade were fit in the largest study of neo-adjuvant chemotherapy [29]. Coefficients derived from these models were then applied to the remaining test data. Model discrimination in the test data was estimated using the AUC from a ROC analysis. These analyses were conducted using Intercooled Stata version 11.2 (Stata Corp, College Station, Texas, USA).

### Comparative evaluation of the representation of genomic drivers by subtype

For each gene in each list of amplified and deleted genes, we fitted an ANOVA linear model relating the expression of that gene to IntClust groups or the PAM50 groups. We measured the goodness of fit of these two models using the adjusted R-squared - a measure that accounts for differences in degrees of freedom of the two models when the models have been completely pre-specified [43]. We computed the differences in adjusted R-squared for each gene and averaged them for each gene list. CIs were obtained using 1,000 bootstrap replicates with the percentile method implemented in the package boot [44]. An overall mean for all studies was computed weighting each study by its size. These analyses were conducted using R version 3.1.0 [45].

Annotated R and Stata code used to generate the reported analyses is provided as Additional file 17.

### Data availability

Data from the METABRIC study is deposited in the European Genome-phenome Archive and can be downloaded from [46]. The IDs for expression are: EGAD00010000210 (discovery) and EGAD00010000211 (validation). The IDs for copy number are: EGAD00010000213 (discovery) and EGAD00010000215 (validation). Details of data sources, including accession codes for all other studies, are provided in Additional file 2.

## Additional files

Additional file 1:
**Cross-tabulation of IntClust subtypes classified using either all probes or one probe per gene in the METABRIC validation study.**
Additional file 2:
**Summary of studies.** Tables of studies included in the analysis. Additional file 3:
**Comparison of copy-number profiles of gene expression-defined IntClust subtypes between METABRIC and TCGA studies.**
Additional file 4:
**Scatter plots depicting the correlation between copy number profiles of tumors classified into IntClust subtypes from the TCGA versus the METABRIC discovery study.**
Additional file 5:
**Comparison of subtyping using RNA-seq or microarray.** Cross-tabulations and summary statistics of subtypes classified into SCMGENE, PAM50 and IntClust subtypes using gene expression data based on either RNA-seq or microarray in 475 samples from TCGA. Additional file 6:
**IntClust subtyping of cell lines.** Scatter plot depicting the goodness of fit for IntClust classification of breast cancer cell lines from both the Sanger COSMIC and CCLE datasets, using copy number alone, gene expression alone and a combination of copy number and gene expression. Weighted scatter plots of the concordance of IntClust classification according to different permutations of the classifier (copy number alone, gene expression alone and a combination of copy number and gene expression) and datasets (Sanger COSMIC and CCLE). Additional file 7:
**PAM50 and SCMGENE subtyping of cell lines.** Weighted scatter plots and cross tabulations of PAM50 and SCMGENE subtypes of cell lines according to dataset (Sanger COSMIC and CCLE). Additional file 8:
**Molecular subtyping of cell lines.** SCMGENE, PAM50 and IntClust subtypes of breast cancer cell lines based on data from both datasets (Sanger COSMIC and CCLE). Additional file 9:
**Distribution of molecular subtypes of breast tumors within subtypes classified by PAM50 or SCMGENE.**
Additional file 10:
**Kaplan-Meier survival plots by molecular subtype restricted to patients recruited to the METABRIC study.**
Additional file 11:
**Comparison of predictive models including either IntClust, PAM50 or SCMGENE subtypes.** **(A)** C-indices and 95% confidence intervals, by ER-positive (left) and ER-negative (right) breast cancer, for prediction models adjusted for tumor size, node status and histological grade, by each of three brackets of follow-up time. **(B)** Receiver-operating-characteristic curves for the performance of logistic regression models adjusted for tumor size, node status and histological grade and containing IntClust or transcriptome-based subtypes, for prediction of pathological complete response. Additional file 12:
**Lists of genes included in genomic loci reported as recurrently amplified or deleted in breast cancer by the TCGA.**
Additional file 13:
**Difference in adjusted R-squared between IntClust and SCMGENE classifiers by study.**
Additional file 14:
**Ranked list of the top 50 genes with R-squared values explained by IntClust (top panel) and those better explained by IntClust compared to PAM50 (bottom panel).** R-squared values are based on analysis-of-variance (ANOVA) models using molecular subtype and levels of gene expression. Additional file 15:
**Boxplots depicting the distribution of expression levels for samples within the METABRIC validation study and TCGA study, for top genes explained by IntClust.**
Additional file 16:
**Scatter plots depicting adjusted R-squared statistics for ANOVA models for all amplified and deleted genes by study.**
Additional file 17:
**Annotated R and Stata code for reproduction of reported analyses.**

## Abbreviations

ANOVA: analysis of variance
AUC: area under the curve
CCLE: Cancer Cell Lines Encyclopedia
CI: confidence interval
CNA: copy number aberration
eQTL: expression quantitative trait locus
ER: estrogen receptor
GEO: Gene Expression Omnibus
HER2: human epidermal growth factor receptor 2
PAM: Prediction Analysis of Microarrays
pCR: pathological complete response
ROC: receiver operating characteristic
