# Supplementary material for: Genome-driven integrated classification of breast cancer validated in over 7,500 samples
Source: Genome Biol. 2014 Aug 28;15(8):431. doi: 10.1186/s13059-014-0431-1 (PMC4166472; doi:10.1186/s13059-014-0431-1)
Supplement: Additional file 1: — Cross-tabulation of IntClust subtypes classified using either all probes or one probe per gene in the METABRIC validation study. [file 13059_2014_431_MOESM1_ESM.pdf]

Additional file 1 – Cross-tabulation of IntClust subtypes classified using either all probes or one probe per gene in the METABRIC validation study

Probe \ Gene	IntClust1	IntClust2	IntClust3	IntClust4	IntClust5	IntClust6	IntClust7	IntClust8	IntClust9	IntClust10
IntClust1	56	0	0	1	0	0	0	0	2	1
IntClust2	0	21	0	0	0	2	1	0	2	0
IntClust3	1	0	149	2	1	1	1	5	0	0
IntClust4	1	0	3	185	1	0	1	0	0	3
IntClust5	0	0	0	3	86	0	0	0	1	1
IntClust6	1	1	0	0	0	35	0	0	2	0
IntClust7	2	0	3	2	1	0	84	0	2	0
IntClust8	0	0	2	0	0	0	0	130	0	0
IntClust9	0	0	0	0	1	0	0	0	64	1
IntClust10	0	0	0	0	0	0	0	0	0	121

Kappa = 0.9
Expected agreement = 12.7%
Agreement = 94.7%
Samples = 983
