# Supplementary material for: Genome-driven integrated classification of breast cancer validated in over 7,500 samples
Source: Genome Biol. 2014 Aug 28;15(8):431. doi: 10.1186/s13059-014-0431-1 (PMC4166472; doi:10.1186/s13059-014-0431-1)
Supplement: Additional file 2: — Summary of studies. Tables of studies included in the analysis. [file 13059_2014_431_MOESM2_ESM.pdf]

Additional file 2 (Page **1** of **4**)

	STUDY										
	CAL*	DFHCC*	DFHCC2*	DFHCC3*	DUKE*	DUKE2*	EMC2*	EORTC10994*	FNCLCC*	HLP*	Total
Source	AE: E-TABM-158	GEO: GSE19615	GEO: GSE18864	GEO: GSE3744	GEO: GSE3143	GEO: GSE6961	GEO: GSE12276	GEO: GSE1561	GEO: GSE7017	AE: E-TABM-543	
Survival time	RFS	DMFS	-	-	-	-	-	-	-	-	
Neoadjuvant therapy	No	No	No	No	No	No	No	No	No	No	
**IntClust**											
IntClust 1	10	10	8	3	9	13	17	3	11	2	86
IntClust 2	0	3	1	2	8	4	8	1	6	1	34
IntClust 3	18	27	18	6	28	29	44	7	44	6	227
IntClust 4	25	18	15	4	41	39	33	7	26	9	217
IntClust 5	8	8	8	3	21	16	18	4	20	9	115
IntClust 6	4	4	3	1	8	4	3	2	1	2	32
IntClust 7	7	10	9	3	14	10	21	6	11	5	96
IntClust 8	17	11	3	0	16	9	15	4	10	5	90
IntClust 9	9	7	9	2	10	14	11	3	11	4	80
IntClust 10	20	17	10	2	15	22	34	12	10	10	152
**PAM50**											
Luminal A	54	47	26	8	19	33	67	16	79	17	366
Luminal B	26	18	6	3	23	11	37	10	40	9	183
HER2	5	18	11	4	50	32	29	4	14	8	175
Basal	26	29	39	8	37	76	59	15	15	14	318
Normal	6	3	2	3	40	8	12	4	2	4	84
Missing	1	0	0	0	1	0	0	0	0	1	3
**SCMGENE**											
ER+/HER2- Low Prolif	37	35	15	6	9	27	5	8	53	0	195
ER+/HER2- High Prolif	35	33	15	6	5	20	5	12	48	0	179
HER2+	16	11	10	2	48	34	105	11	32	0	269
ER-/HER2-	30	36	44	12	108	79	89	18	17	0	433
Missing	0	0	0	0	0	0	0	0	0	53	53

*Datasets collected and curated by Haibe-Kains et al JNCI 2013 and downloaded from http://compbio.dfci.harvard.edu/pubs/sbtpaper. Presented source details are taken from Haibe-Kains et al JNCI 2013.

RFS = Relapse-free-survival

DMFS = Distant-metastasis-free-survival

	IRB *	IRB/JRH/NUH	KOO*	LUND*	LUND2*	MAINZ*	MAQC2*	MB-DISCOVERY	MB-VALIDATION	MCCC*	Total
Source		GEO:GSE45255	Authors' website	GEO: GSE5325	GEO: GSE5325	GEO: GSE11121	GEO: GSE20194			GEO: GSE19177	
Survival time	-	DMFS	-	-	-	DMFS	-	BCSS	BCSS	-	
Neoadjuvant therapy	No	No	No	No	No	No	No	No	No	No	
**IntClust**											
IntClust 1	9	12	5	12	5	12	17	76	60	7	215
IntClust 2	4	3	5	4	3	5	6	45	26	4	105
IntClust 3	20	26	15	28	13	46	41	156	160	15	520
IntClust 4	26	29	18	38	22	32	46	167	194	8	580
IntClust 5	10	14	11	9	11	18	21	94	91	4	283
IntClust 6	5	2	3	4	4	4	11	44	39	1	117
IntClust 7	10	11	8	7	14	20	11	109	94	7	291
IntClust 8	16	19	12	20	11	26	23	143	132	8	410
IntClust 9	7	10	4	8	7	16	20	67	66	6	211
IntClust 10	22	13	7	13	15	21	34	96	121	15	357
**PAM50**											
Luminal A	55	56	7	41	37	41	89	466	252	25	1069
Luminal B	22	35	15	24	10	41	46	268	220	19	700
HER2	16	18	24	20	22	29	26	87	153	5	400
Basal	32	19	18	32	26	36	50	118	211	24	566
Normal	4	10	22	24	9	50	17	58	141	2	337
Missing	0	1	2	2	1	3	2	0	6	0	17
**SCMGENE**											
ER+/HER2- Low Prolif	41	47	2	28	27	34	0	368	374	0	921
ER+/HER2- High Prolif	26	61	3	28	24	27	0	368	312	0	849
HER2+	20	16	23	8	10	25	0	110	102	0	314
ER-/HER2-	42	15	60	73	28	114	0	151	195	0	678
Missing	0	0	0	6	16	0	230	0	0	75	327

*Datasets collected and curated by Haibe-Kains et al JNCI 2013 and downloaded from http://compbio.dfci.harvard.edu/pubs/sbtpaper. Presented source details are taken from Haibe-Kains et al JNCI 2013.
RFS = Relapse-free-survival
DMFS = Distant-metastasis-free-survival

	MDA4*	MDACC Hatzis	MDACC T/FAC trial	MDACC/IGR	MSK*	MUG*	NCCS*	NCI*	NKI*	PNC*	Total
Source	MDACC DB	GEO:GSE25066	GEO:GSE20271	GEO:GSE22093	GEO: GSE2603	GEO: GSE10510	GEO: GSE5364	Authors' website	Rosetta Inpharmatics		
Survival time	-				DMFS	-	-	RFS	RFS		
Neoadjuvant therapy	No	Yes	Yes	Yes	No	No	No	No	No	No	
**IntClust**											
IntClust 1	8	50	14	5	11	7	12	4	28	5	144
IntClust 2	0	11	5	3	2	13	6	0	6	1	47
IntClust 3	11	92	31	13	13	84	29	20	55	14	362
IntClust 4	21	100	54	22	19	25	38	25	55	16	375
IntClust 5	0	12	11	8	9	3	21	10	29	14	117
IntClust 6	3	13	5	4	4	0	8	3	16	1	57
IntClust 7	6	31	11	5	5	9	14	7	36	7	131
IntClust 8	6	34	14	7	11	4	20	14	45	9	164
IntClust 9	2	41	13	7	9	7	16	6	16	9	126
IntClust 10	7	71	20	15	15	0	19	10	51	16	224
**PAM50**											
Luminal A	27	174	59	21	31	16	49	29	174	36	616
Luminal B	16	91	40	14	21	35	37	35	72	17	378
HER2	2	22	18	14	8	22	37	14	26	12	175
Basal	15	142	42	34	31	18	32	14	60	26	414
Normal	4	25	19	5	7	36	27	7	4	1	135
Missing	0	1	0	1	0	25	1	0	1	0	29
**SCMGENE**											
ER+/HER2- Low Prolif	26	13	8	14	33	35	43	10	115	23	320
ER+/HER2- High Prolif	11	13	3	17	22	37	43	7	100	25	278
HER2+	6	240	16	18	7	34	56	12	54	22	465
ER-/HER2-	21	189	151	40	36	46	41	70	64	22	680
Missing	0	0	0	0	0	0	0	0	4	0	4

*Datasets collected and curated by Haibe-Kains et al JNCI 2013 and downloaded from http://compbio.dfci.harvard.edu/pubs/sbtpaper. Presented source details are taken from Haibe-Kains et al JNCI 2013.
RFS = Relapse-free-survival
DMFS = Distant-metastasis-free-survival

	STK	STNO2	SUPERTAM_HGU133A	SUPERTAM_HGU133PLUS2	TCGA	TOP trial	TRANSBIG	UCSF	UNC4	UNT	Total
Source	GEO: GSE1456		GEO: GSE6532/GSE9195, GEO: GSE17705, GEO: GSE12093	GEO: GSE2109, GEO: GSE6532/GSE9195	TCGA portal	GEO: GSE16446	GEO:GEO: GSE7390	Authors' website	UNC DB	GEO: GSE2990	
Survival time	RFS	RFS	DMFS	DMFS			RFS	RFS	RFS	RFS	
Neoadjuvant therapy	No	No	No	No	No	Yes	No	No	No	No	
**IntClust**											
IntClust 1	13	4	67	36	70	9	19	15	23	3	259
IntClust 2	6	7	26	25	20	0	4	6	7	2	103
IntClust 3	43	29	169	68	134	23	35	42	57	26	626
IntClust 4	36	25	157	119	112	28	26	34	62	18	617
IntClust 5	10	15	57	42	55	10	17	18	33	7	264
IntClust 6	6	1	25	20	32	3	6	4	16	4	117
IntClust 7	14	7	81	38	85	13	23	10	24	8	303
IntClust 8	12	9	125	75	98	7	23	12	27	10	398
IntClust 9	10	10	67	45	66	16	11	8	22	8	263
IntClust 10	8	11	82	49	110	11	34	13	34	6	358
**PAM50**											
Luminal A	33	39	399	222	376	1	96	65	110	47	1388
Luminal B	39	25	217	114	173	1	31	44	47	18	709
HER2	26	26	67	43	68	21	22	19	31	10	333
Basal	23	23	110	80	136	89	42	28	77	9	617
Normal	35	5	62	54	28	8	7	5	38	8	250
Missing	2	0	1	4	1	0	0	1	2	0	11
**SCMGENE**											
ER+/HER2- Low Prolif	36	32	407	195	286	14	62	0	97	46	1175
ER+/HER2- High Prolif	28	32	267	177	286	9	74	0	79	22	974
HER2+	10	19	70	49	70	31	15	0	38	12	314
ER-/HER2-	84	35	112	96	140	66	47	0	91	12	683
Missing	0	0	0	0	0	0	0	162	0	0	162

*Datasets collected and curated by Haibe-Kains et al JNCI 2013 and downloaded from http://compbio.dfci.harvard.edu/pubs/sbtpaper. Presented source details are taken from Haibe-Kains et al JNCI 2013.

RFS = Relapse-free-survival

DMFS = Distant-metastasis-free-survival
