# Supplementary material for: Genome-driven integrated classification of breast cancer validated in over 7,500 samples
Source: Genome Biol. 2014 Aug 28;15(8):431. doi: 10.1186/s13059-014-0431-1 (PMC4166472; doi:10.1186/s13059-014-0431-1)

Additional file 3 – Comparison of copy-number profiles of gene expression defined IntClust subtypes between METABRIC and TCGA studies

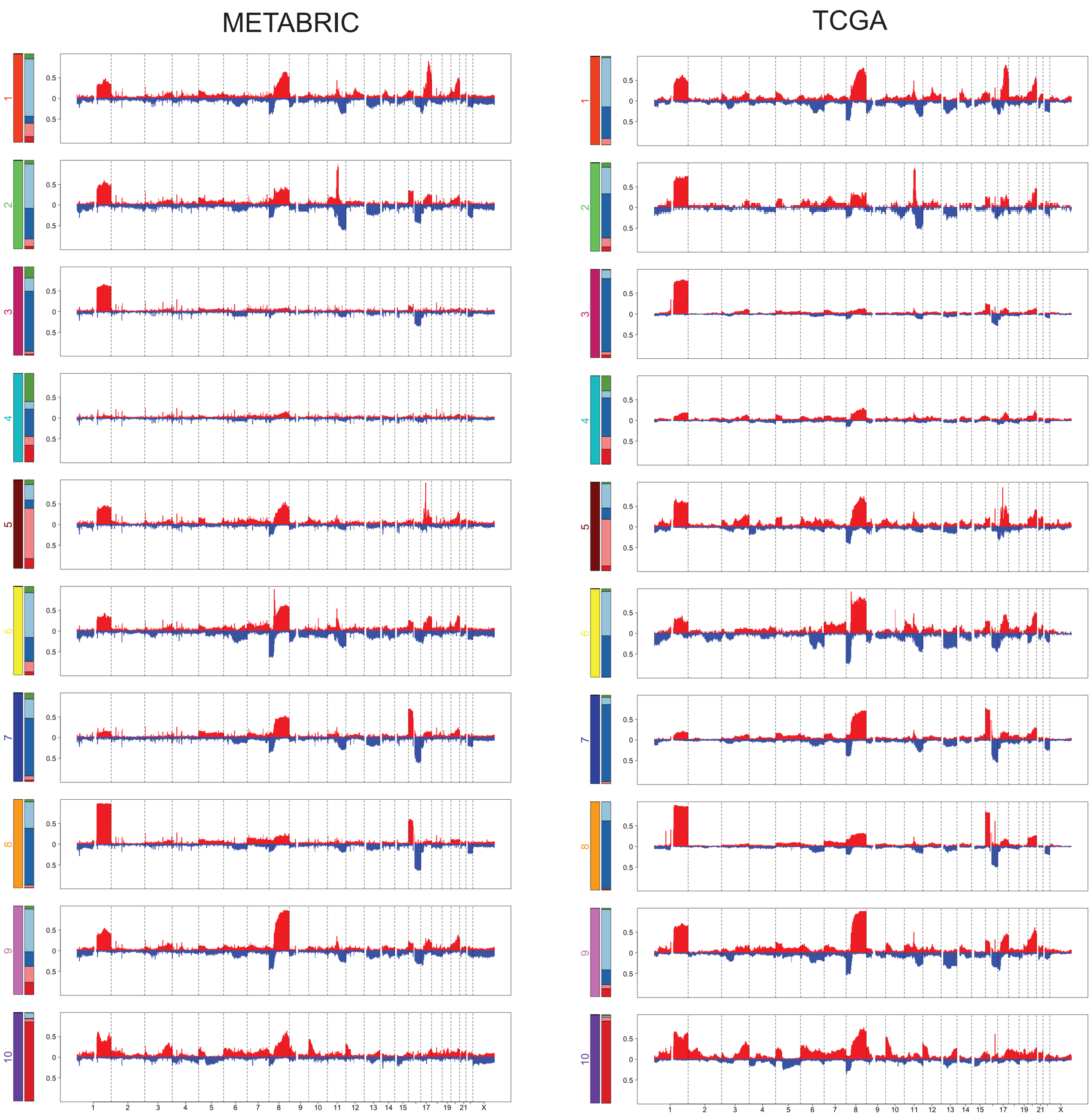

Supplement: Additional file 3: — Comparison of copy-number profiles of gene expression-defined IntClust subtypes between METABRIC and TCGA studies. [file 13059_2014_431_MOESM3_ESM.pdf]
