# Supplementary material for: Genome-driven integrated classification of breast cancer validated in over 7,500 samples
Source: Genome Biol. 2014 Aug 28;15(8):431. doi: 10.1186/s13059-014-0431-1 (PMC4166472; doi:10.1186/s13059-014-0431-1)

Additional file 4 – Scatter plots depicting the correlation between copy-number profiles of tumours classified into IntClust subtypes from the TCGA versus the METABRIC discovery study

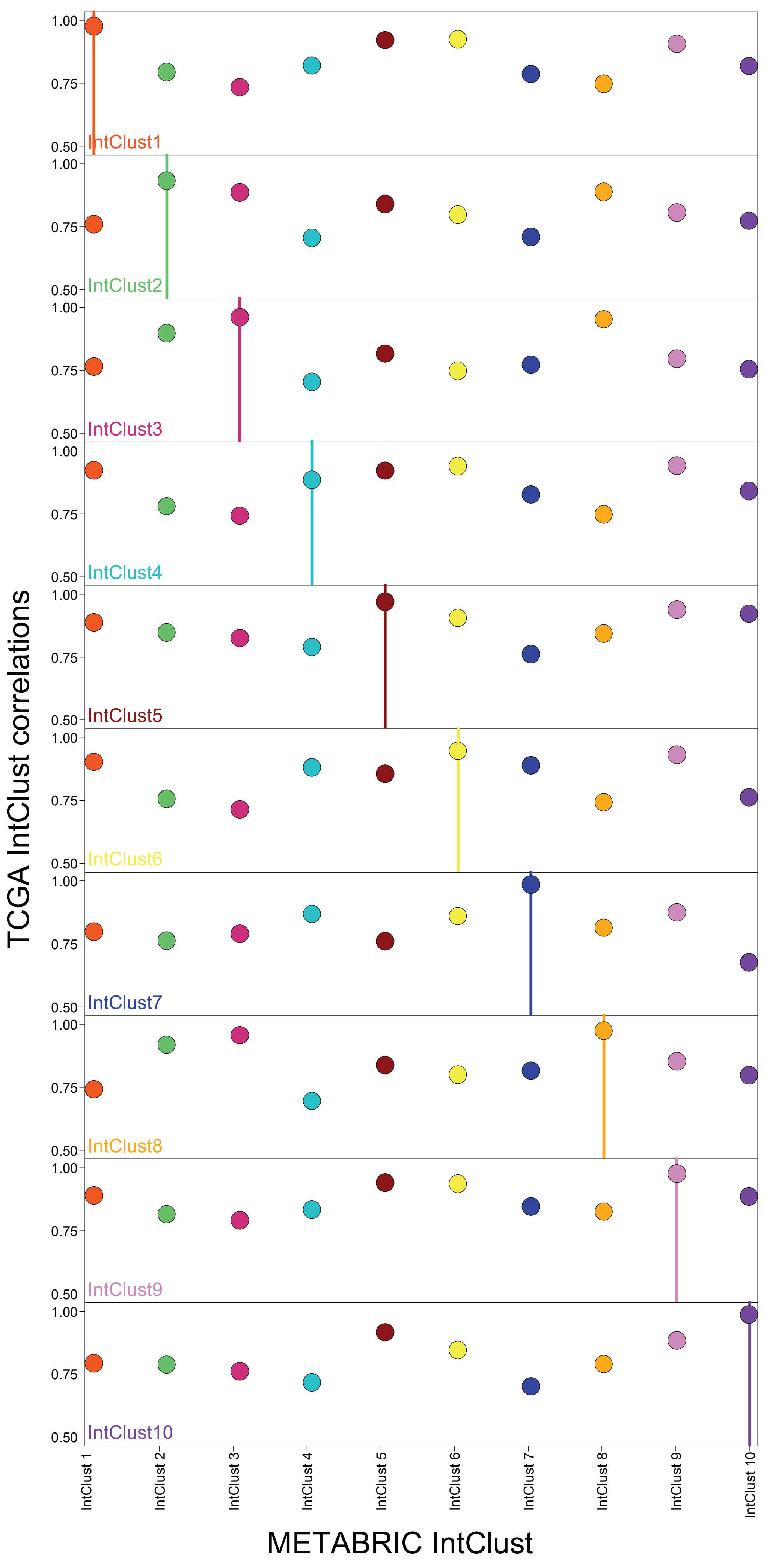

Supplement: Additional file 4: — Scatter plots depicting the correlation between copy number profiles of tumors classified into IntClust subtypes from the TCGA versus the METABRIC discovery study. [file 13059_2014_431_MOESM4_ESM.pdf]
