# Supplementary material for: Genome-driven integrated classification of breast cancer validated in over 7,500 samples
Source: Genome Biol. 2014 Aug 28;15(8):431. doi: 10.1186/s13059-014-0431-1 (PMC4166472; doi:10.1186/s13059-014-0431-1)
Supplement: Additional file 5: — Comparison of subtyping using RNA-seq or microarray. Cross-tabulations and summary statistics of subtypes classified into SCMGENE, PAM50 and IntClust subtypes using gene expression data based on either RNA-seq or microarray in 475 samples from TCGA. [file 13059_2014_431_MOESM5_ESM.pdf]

Additional file 5 – Comparison of subtyping using RNA-seq or microarray.
Cross-tablulations and summary statistics of subtypes classified into SCMGENE, PAM50 and IntClust subtypes using gene expression data based on either RNA-seq or microarray in 475 samples from TCGA

Array \ RNA-seq	ER_posHER2_negLowProlif	ER_posHER2_negHighProlif	HER2	ER_negHER2_neg
ER_posHER2_negLowProlif	166	11	0	1
ER_posHER2_negHighProlif	9	145	0	1
HER2	1	3	47	1
ER_negHER2_neg	0	6	0	84

Kappa = 0.9
Expected agreement = 29.8%
Agreement = 93.1%
Samples = 475

Array \ RNA-seq	LuminalA	LuminalB	HER2	Basal	Normal
LuminalA	226	4	2	2	3
LuminalB	5	86	3	0	0
HER2	0	3	40	0	1
Basal	2	0	1	87	0
Normal	3	0	0	1	6

Kappa = 0.9
Expected agreement = 33.2%
Agreement = 93.7%
Samples = 475

Array \ RNA-seq	IntClust1	IntClust2	IntClust3	IntClust4	IntClust5	IntClust6	IntClust7	IntClust8	IntClust9	IntClust10
IntClust1	39	0	3	1	1	1	2	1	0	0
IntClust2	1	9	0	0	0	0	0	0	1	0
IntClust3	0	1	56	5	0	0	2	9	0	1
IntClust4	1	1	3	48	0	2	4	0	0	1
IntClust5	1	1	1	5	31	0	1	0	0	0
IntClust6	0	0	0	1	0	12	0	0	0	0
IntClust7	3	1	2	4	0	0	40	0	1	0
IntClust8	0	1	10	1	0	0	0	45	2	0
IntClust9	1	0	0	0	1	1	2	1	32	2
IntClust10	0	0	0	1	1	0	0	1	2	74

Kappa = 0.8
Expected agreement = 12.0%
Agreement = 81.3%
Samples = 475
