# Supplementary figures and images for: Genome-driven integrated classification of breast cancer validated in over 7,500 samples
Source: Genome Biol. 2014 Aug 28;15(8):431. doi: 10.1186/s13059-014-0431-1 (PMC4166472; doi:10.1186/s13059-014-0431-1)

Additional file 6 – IntClust subtyping of cell lines

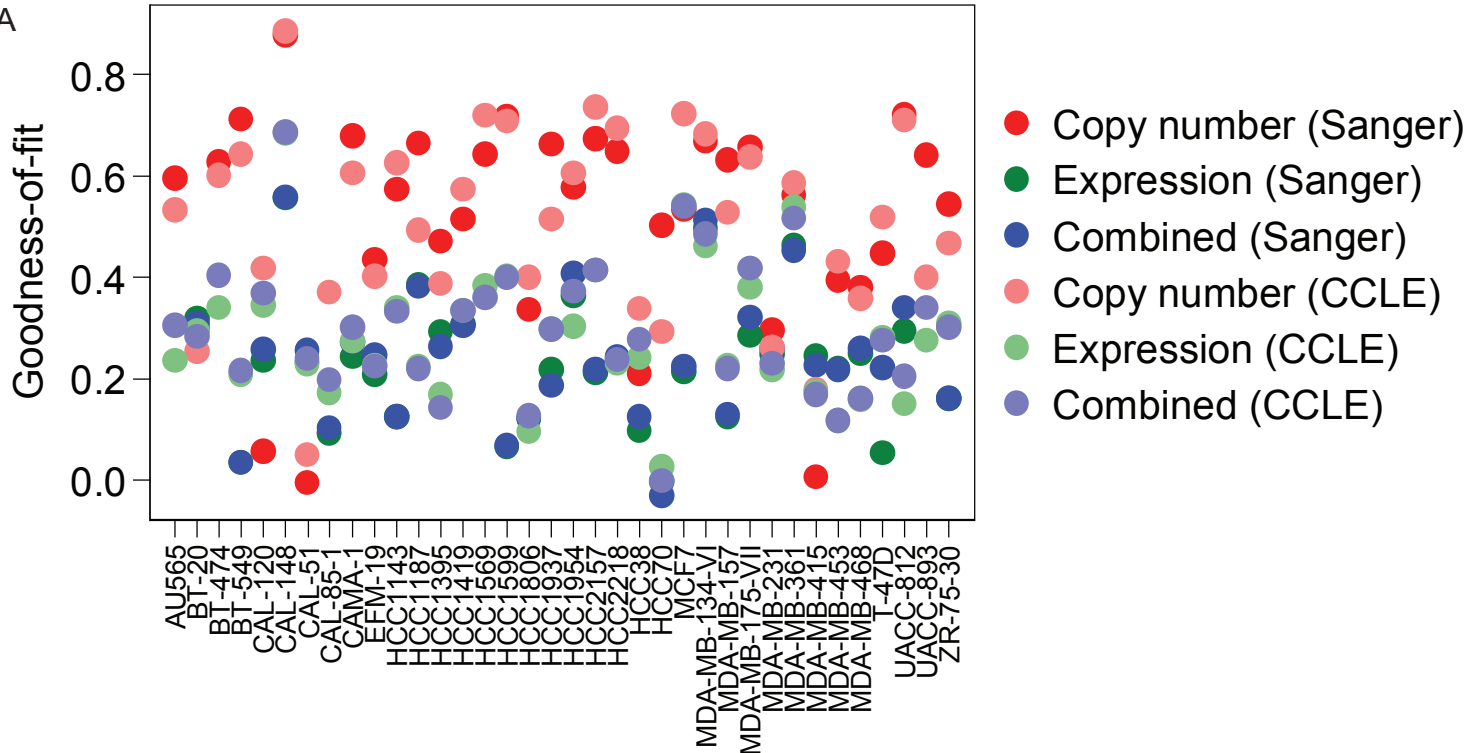

**B**

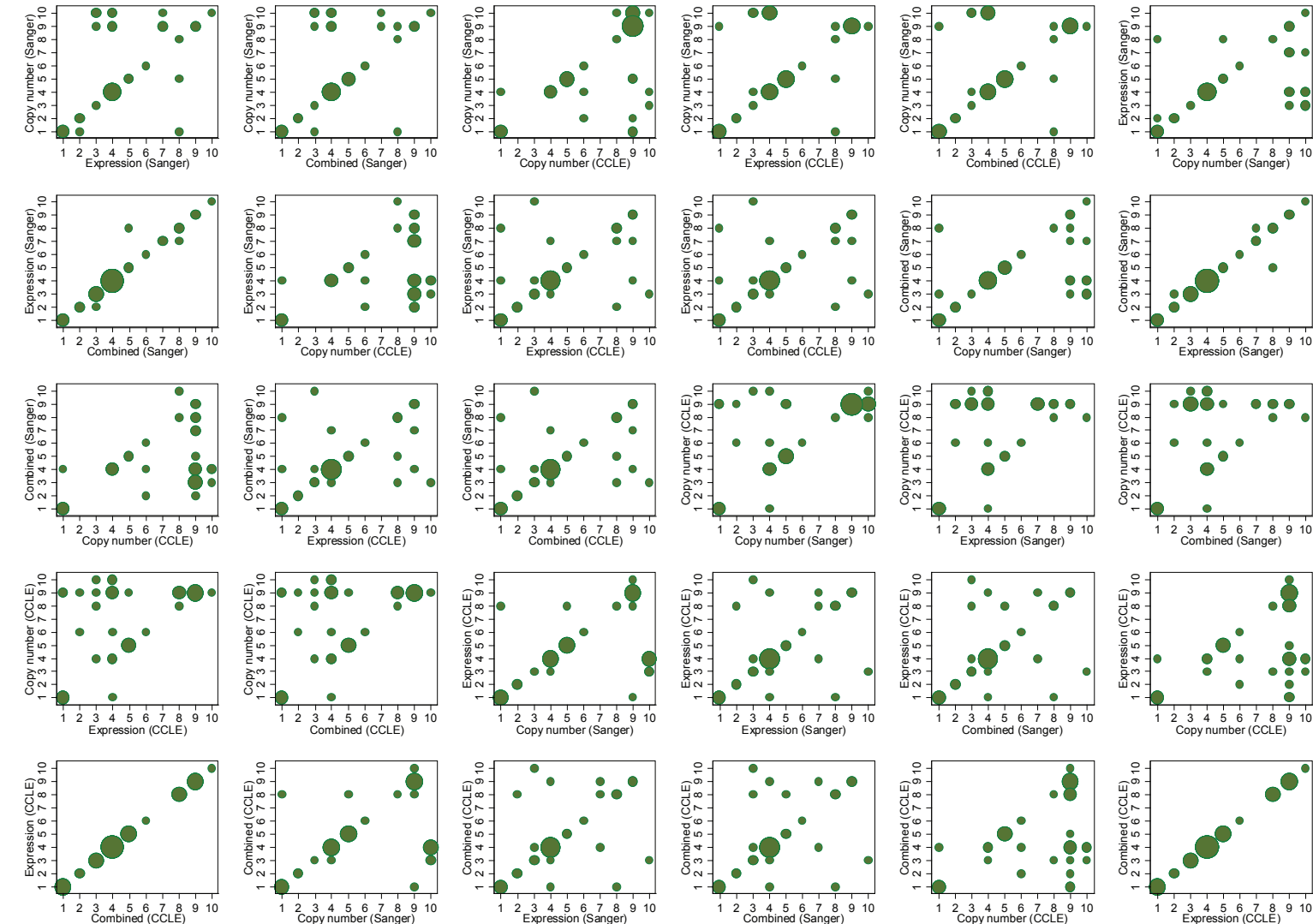

Supplement: Additional file 6: — IntClust subtyping of cell lines. Scatter plot depicting the goodness of fit for IntClust classification of breast cancer cell lines from both the Sanger COSMIC and CCLE datasets, using copy number alone, gene expression alone and a combination of copy number and gene expression. Weighted scatter plots of the concordance of IntClust classification according to different permutations of the classifier (copy number alone, gene expression alone and a combination of copy number and gene expression) and datasets (Sanger COSMIC and CCLE). [file 13059_2014_431_MOESM6_ESM.pdf]

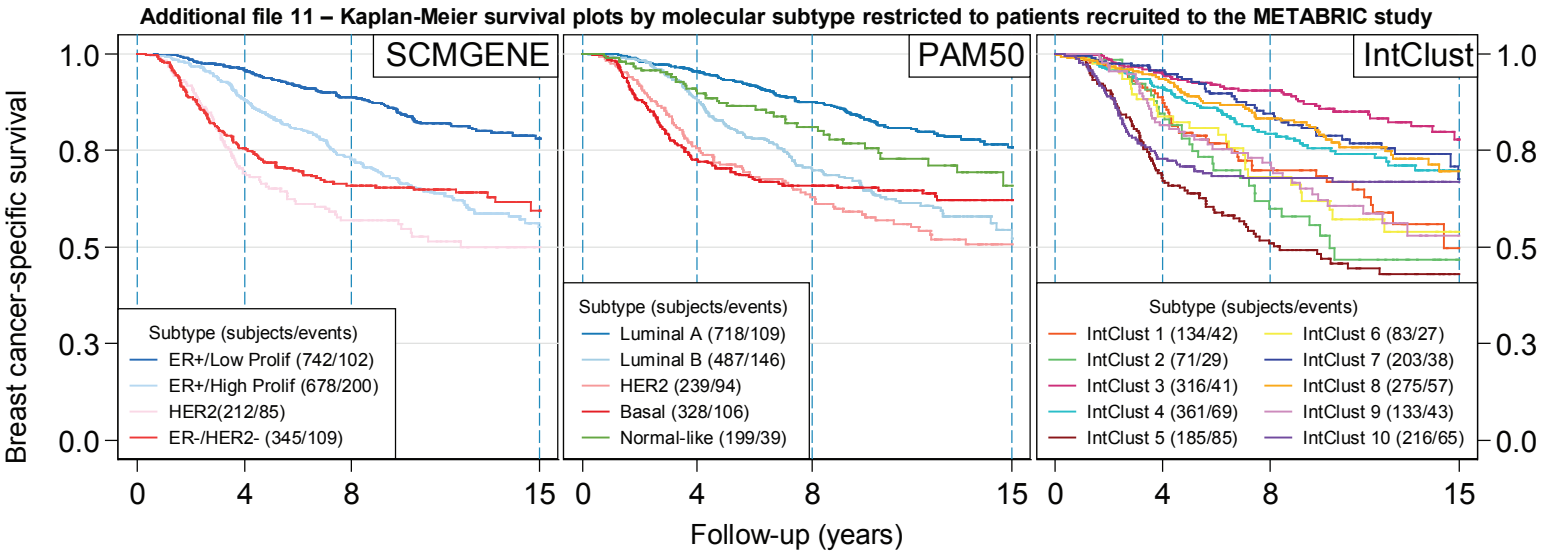

Supplement: Additional file 10: — Kaplan-Meier survival plots by molecular subtype restricted to patients recruited to the METABRIC study. [file 13059_2014_431_MOESM10_ESM.pdf]

# **Additional file 11- Comparison of predictive models including either IntClust or PAM50 subtypes**

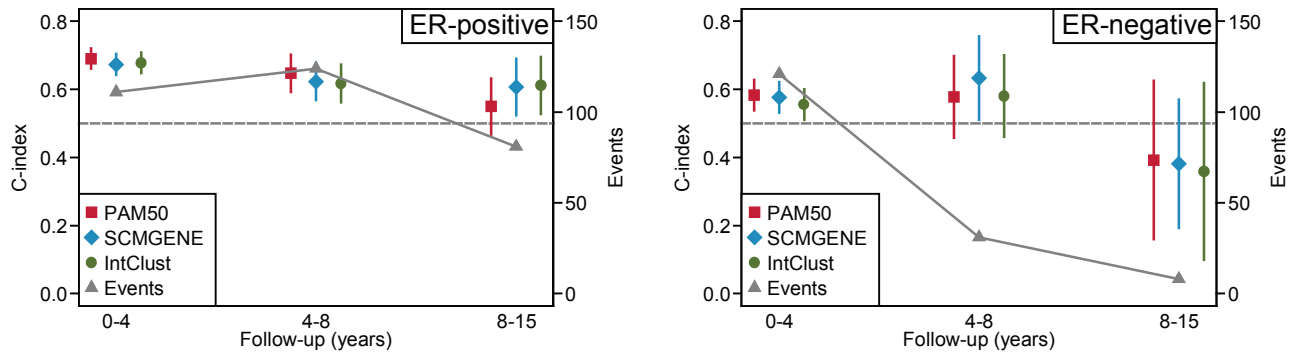

**B**

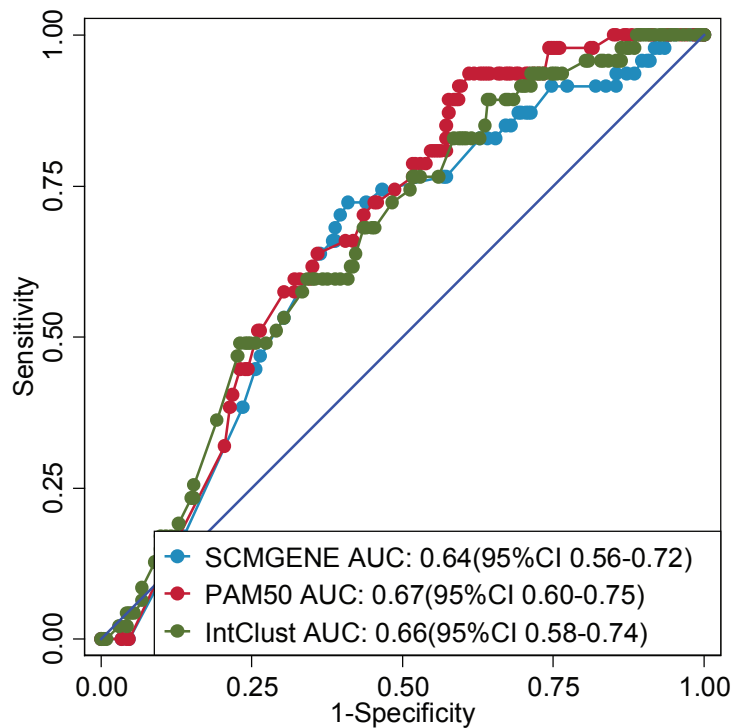

Supplement: Additional file 11: — Comparison of predictive models including either IntClust, PAM50 or SCMGENE subtypes. (A) C-indices and 95% confidence intervals, by ER-positive (left) and ER-negative (right) breast cancer, for prediction models adjusted for tumor size, node status and histological grade, by each of three brackets of follow-up time. (B) Receiver-operating-characteristic curves for the performance of logistic regression models adjusted for tumor size, node status and histological grade and containing IntClust or transcriptome-based subtypes, for prediction of pathological complete response. [file 13059_2014_431_MOESM11_ESM.pdf]

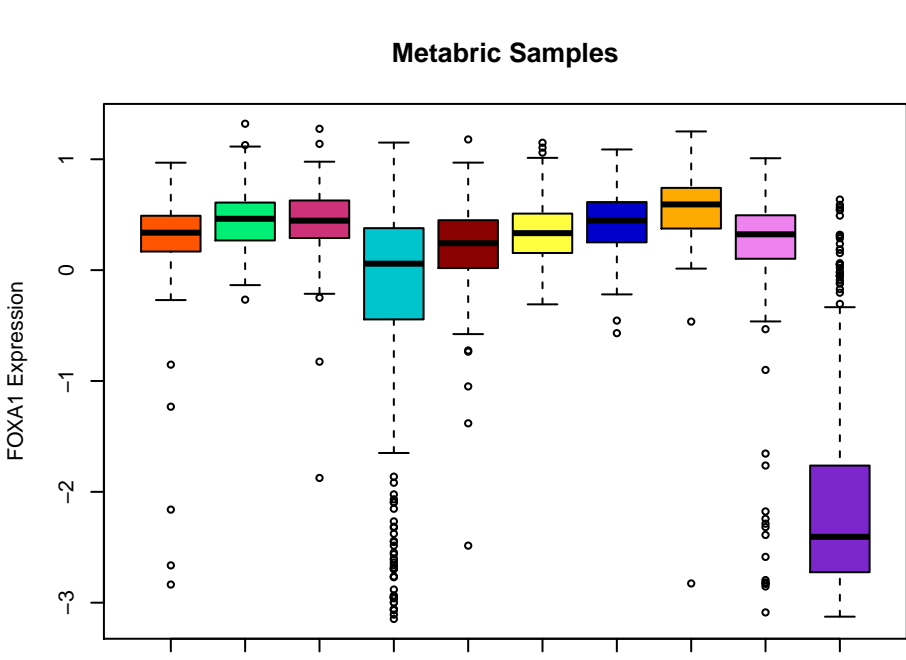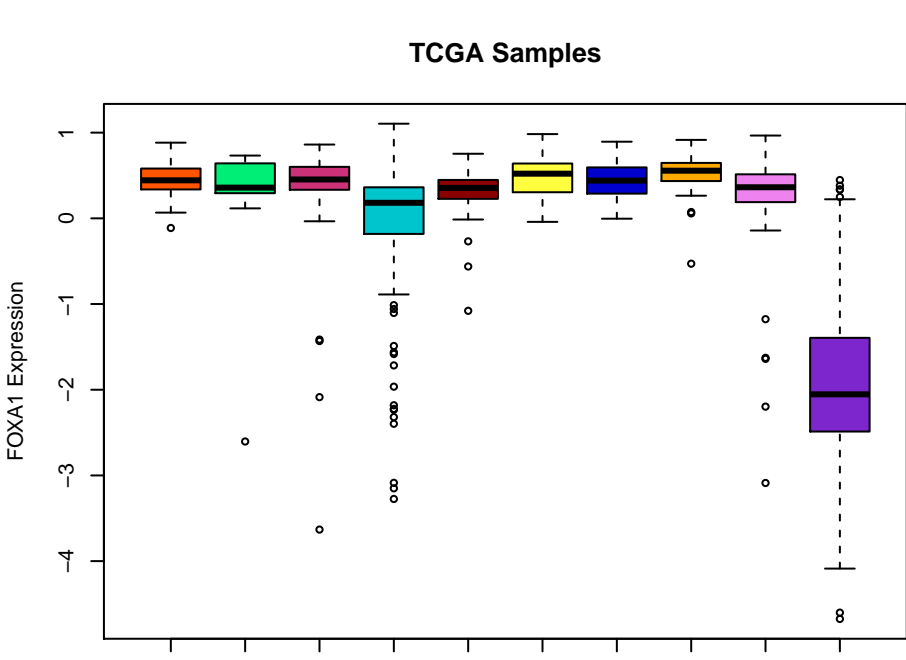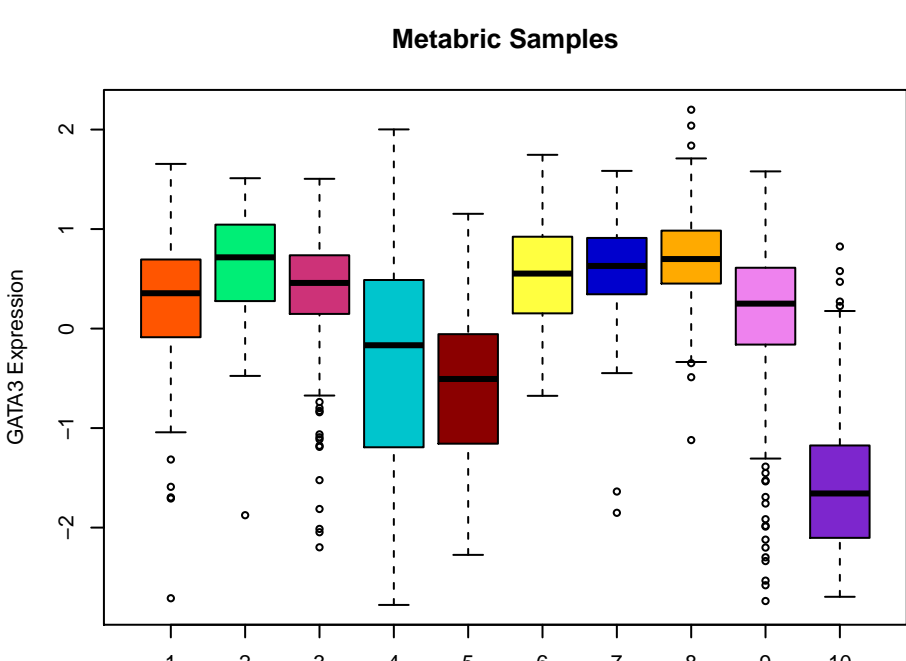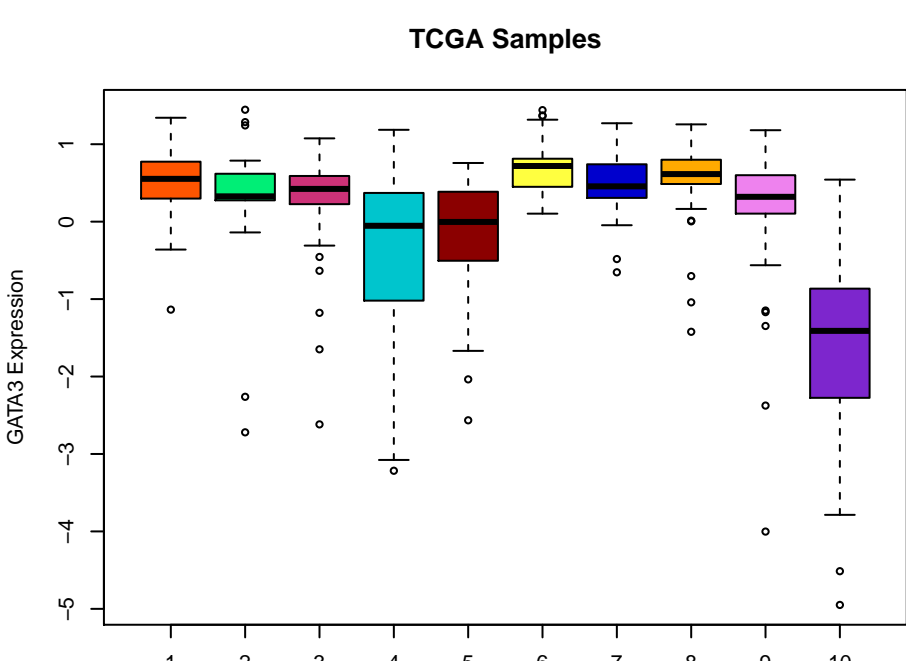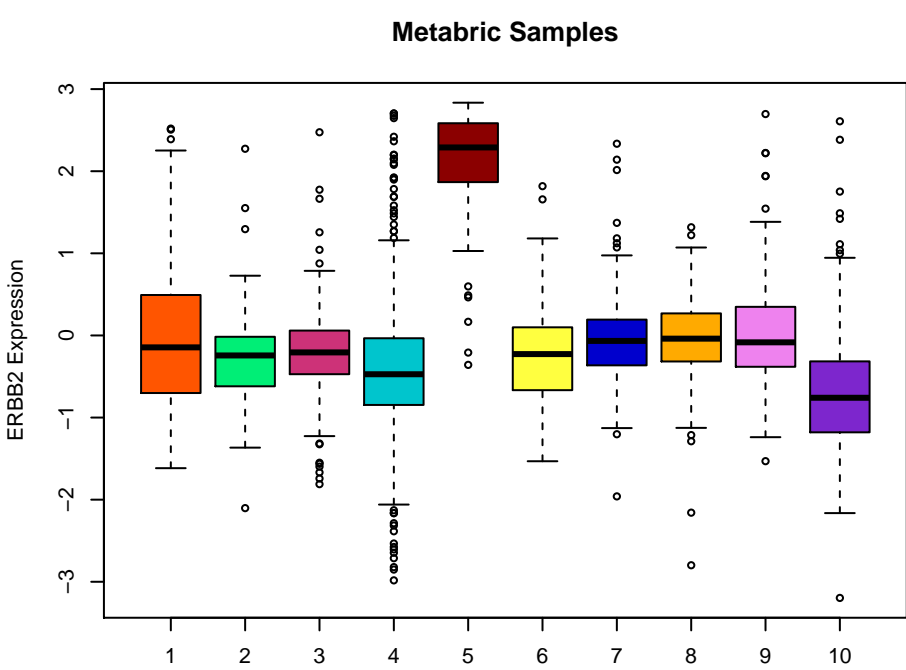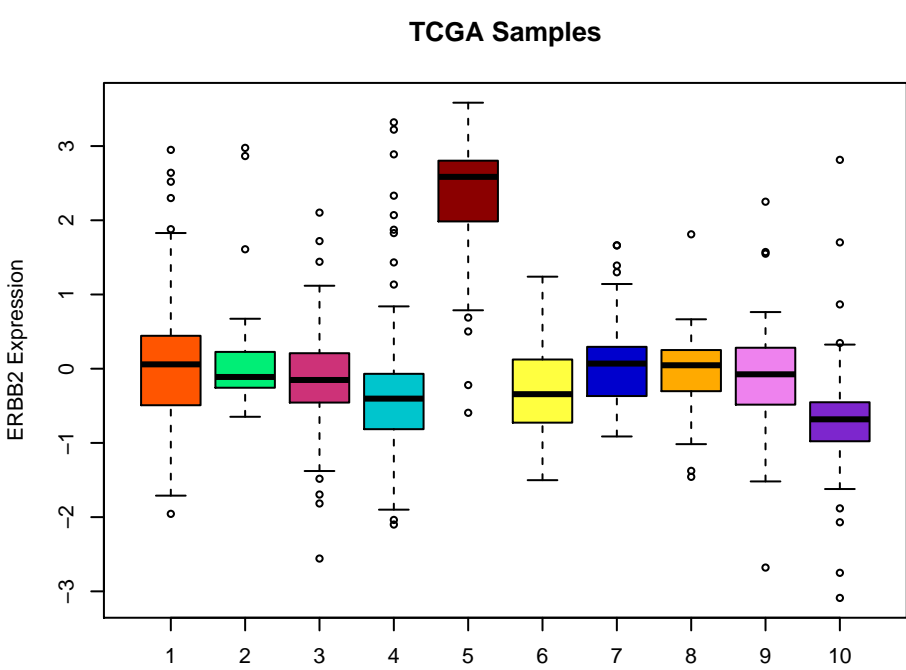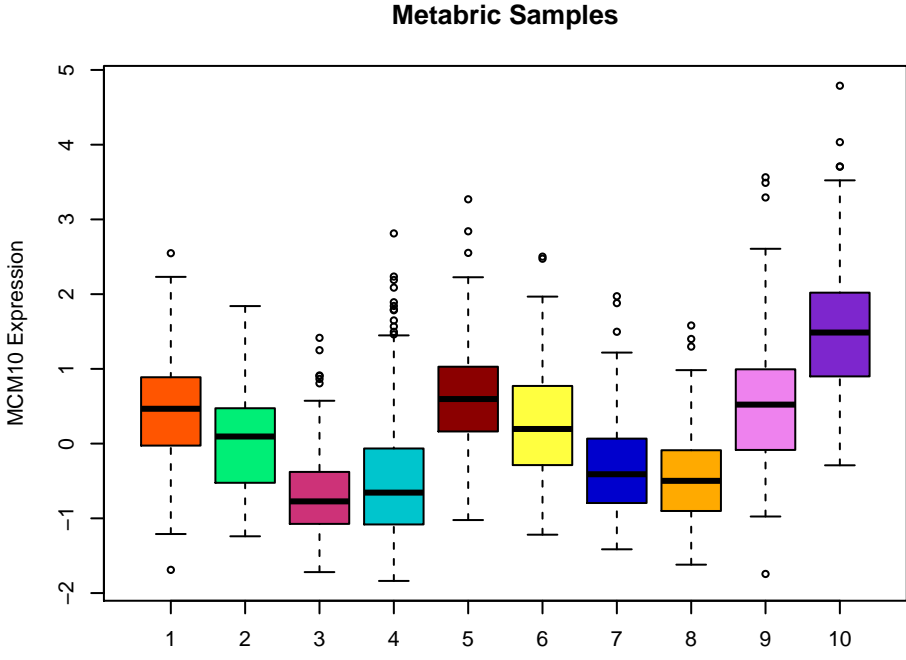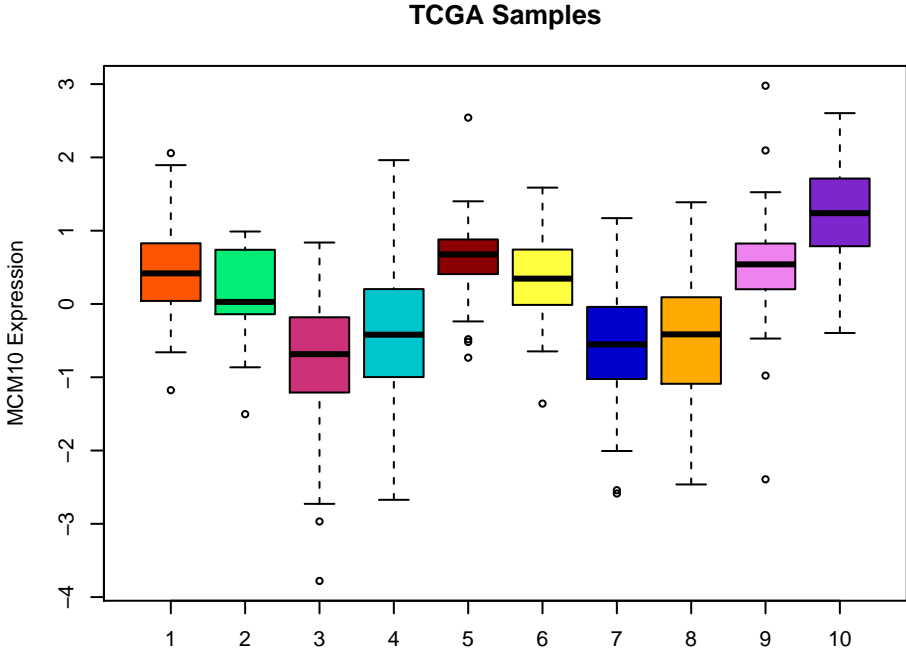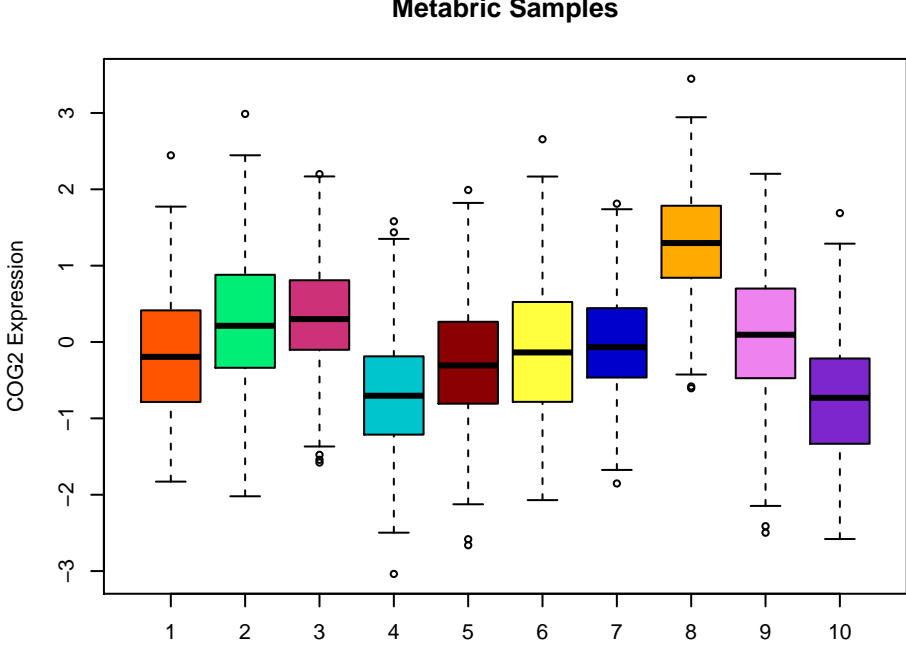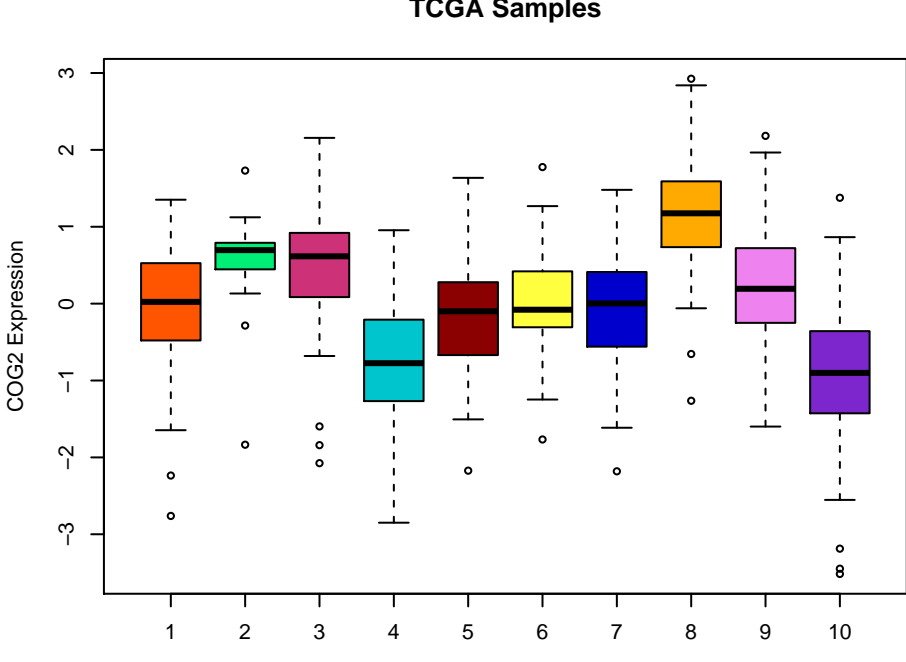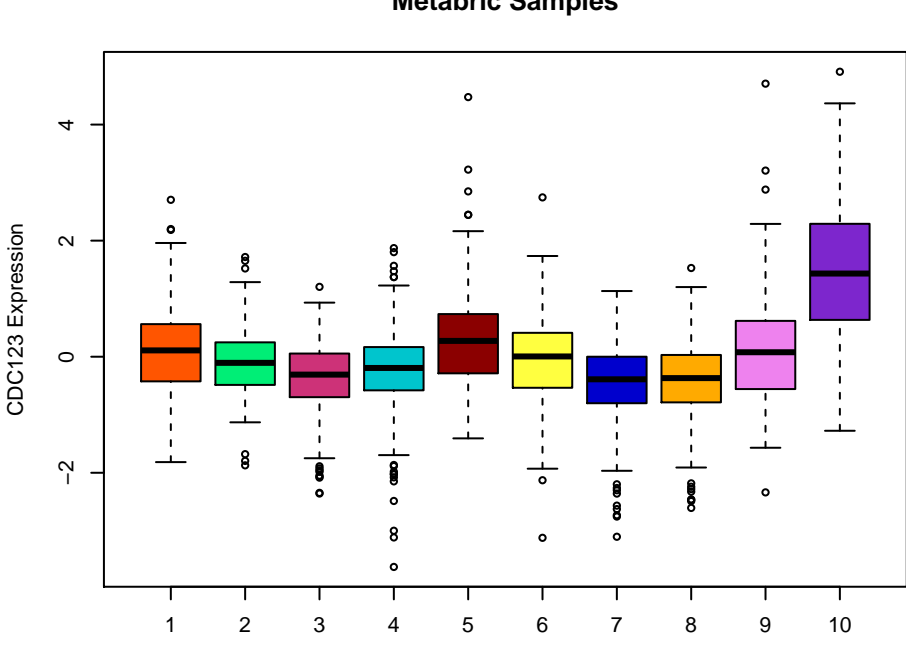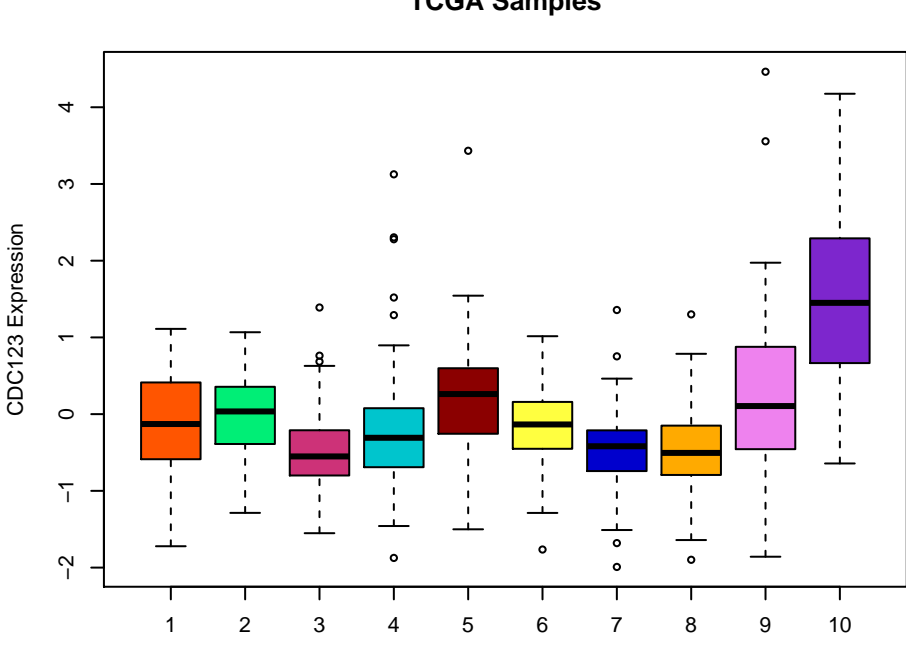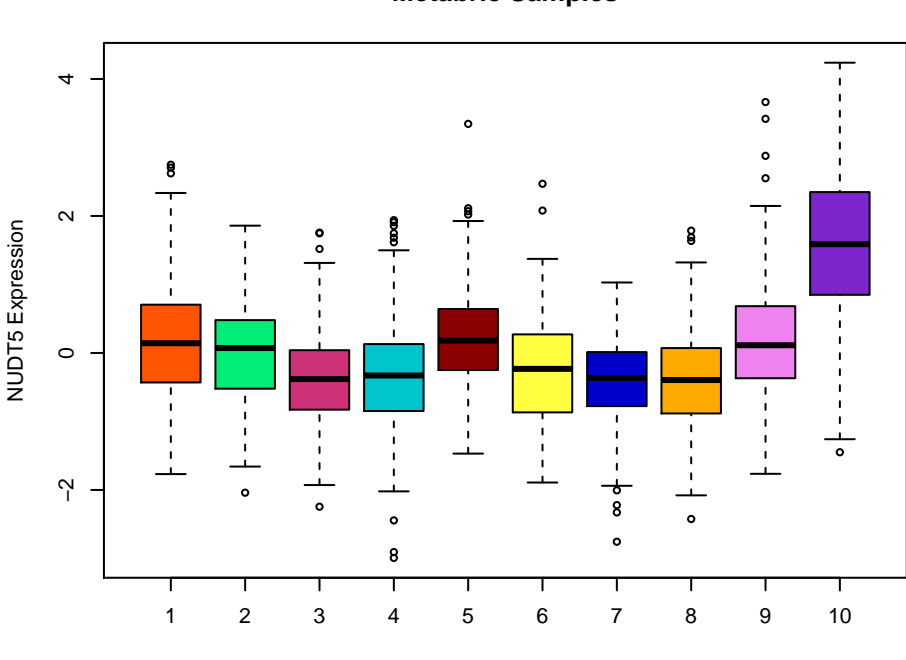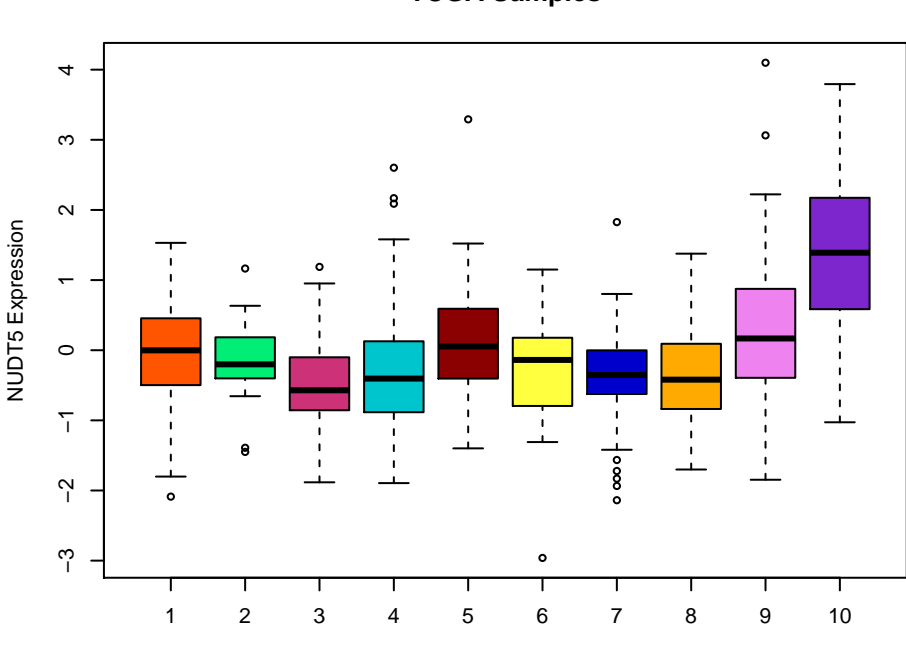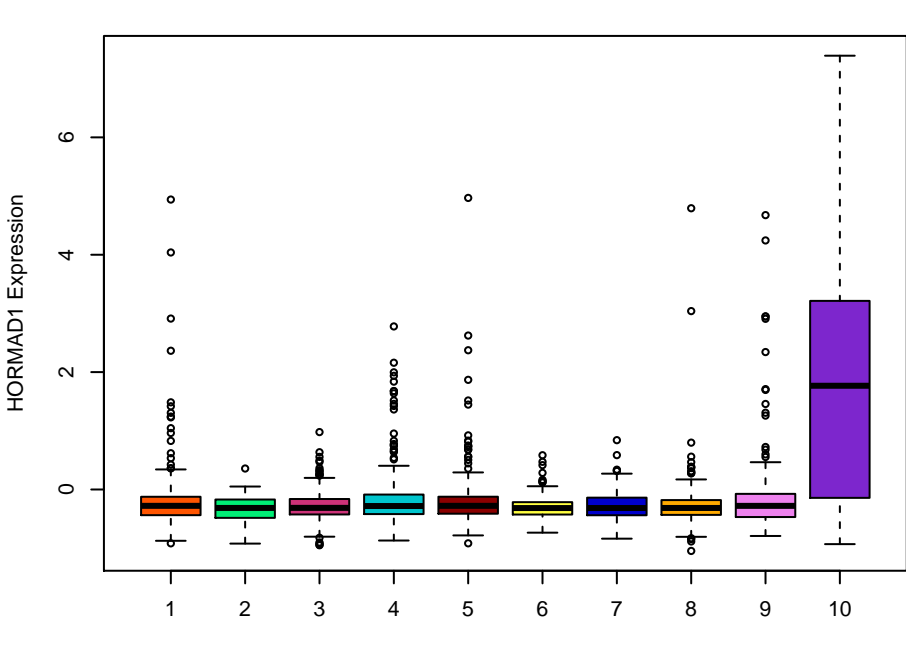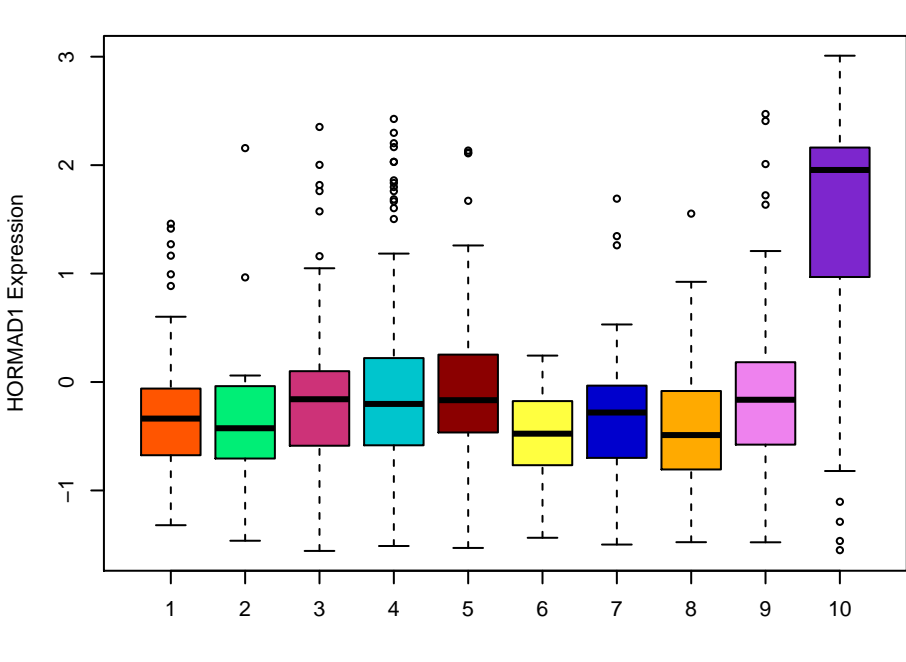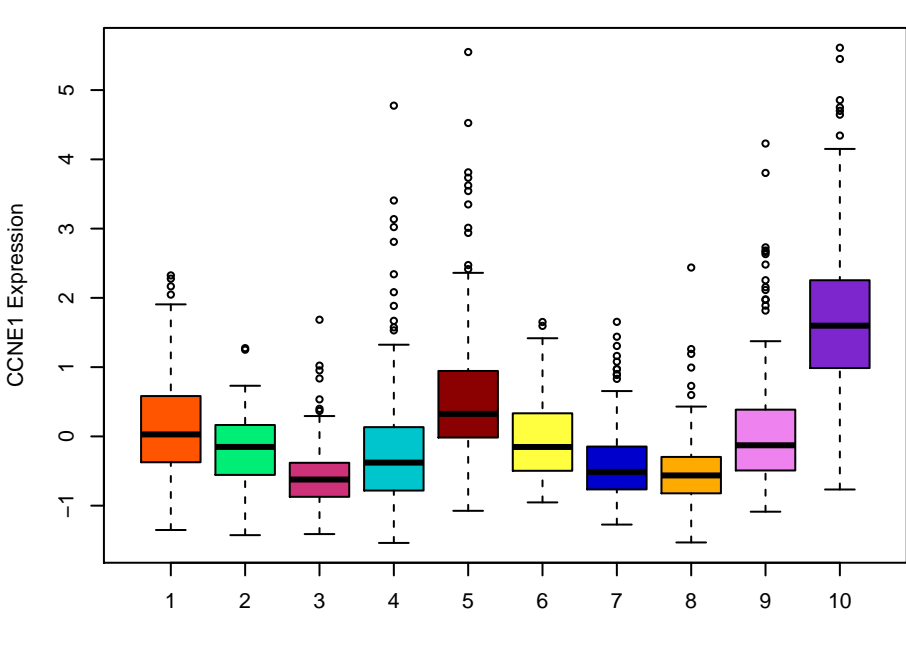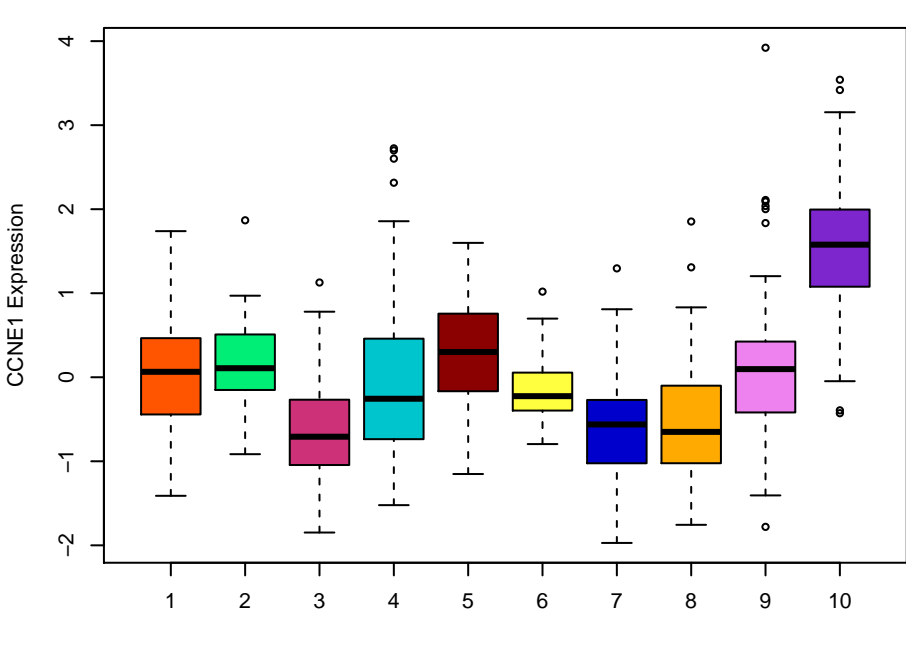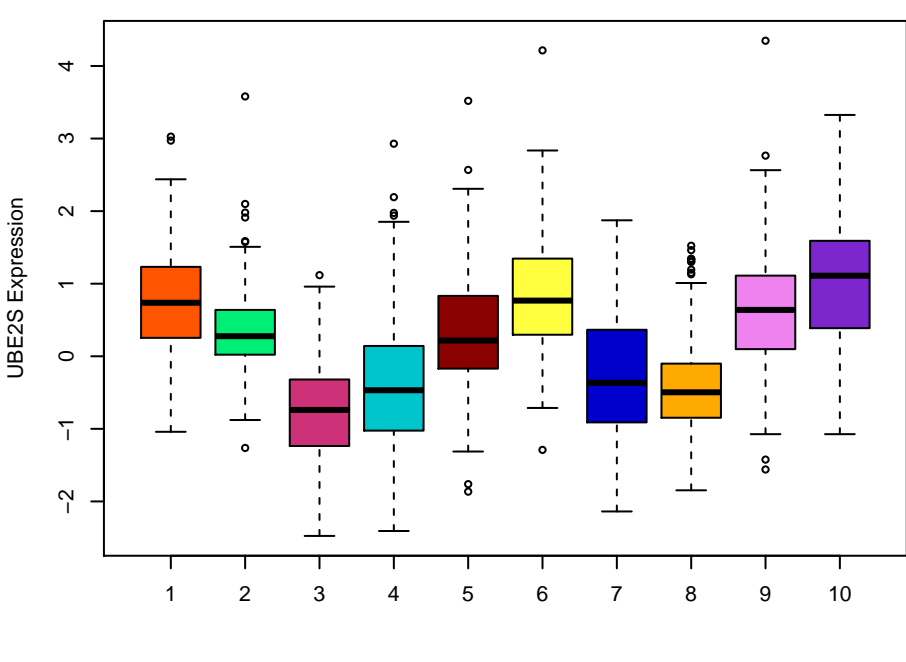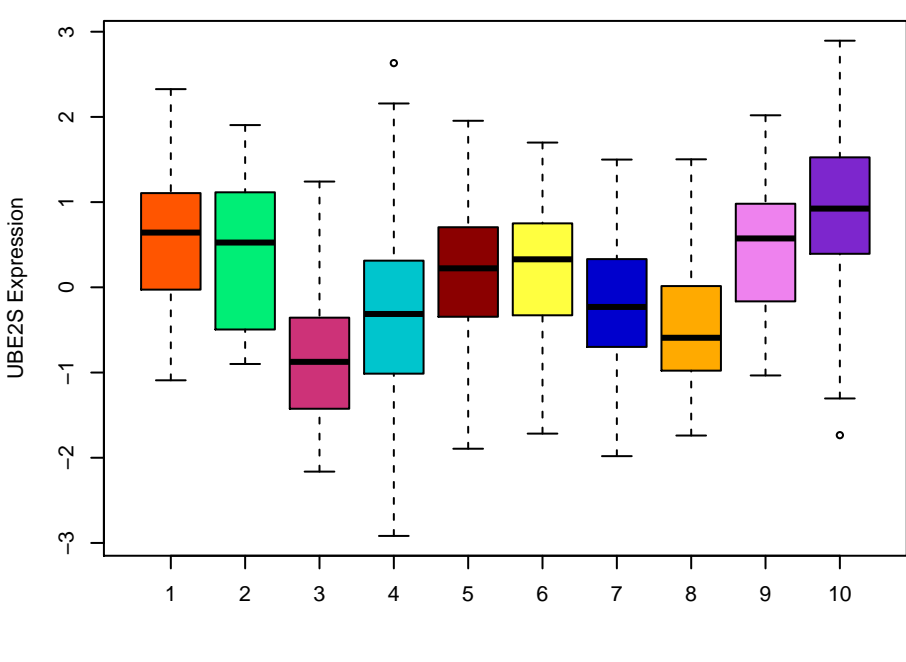

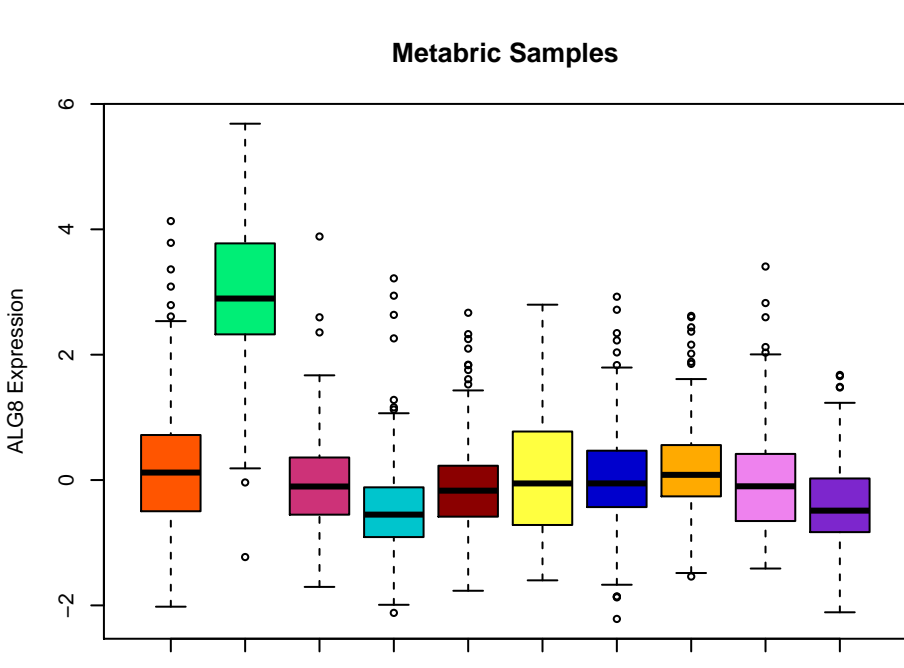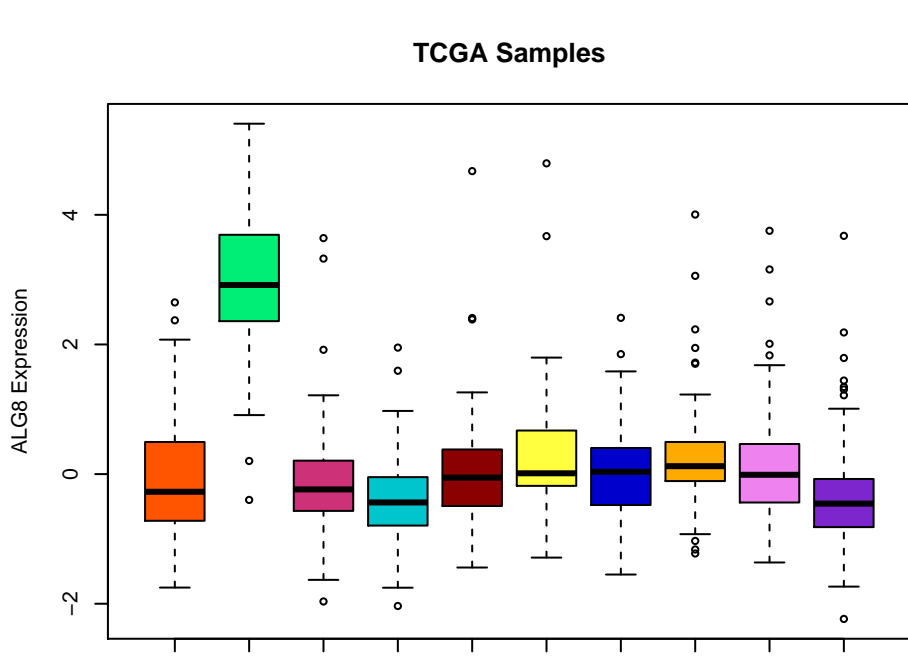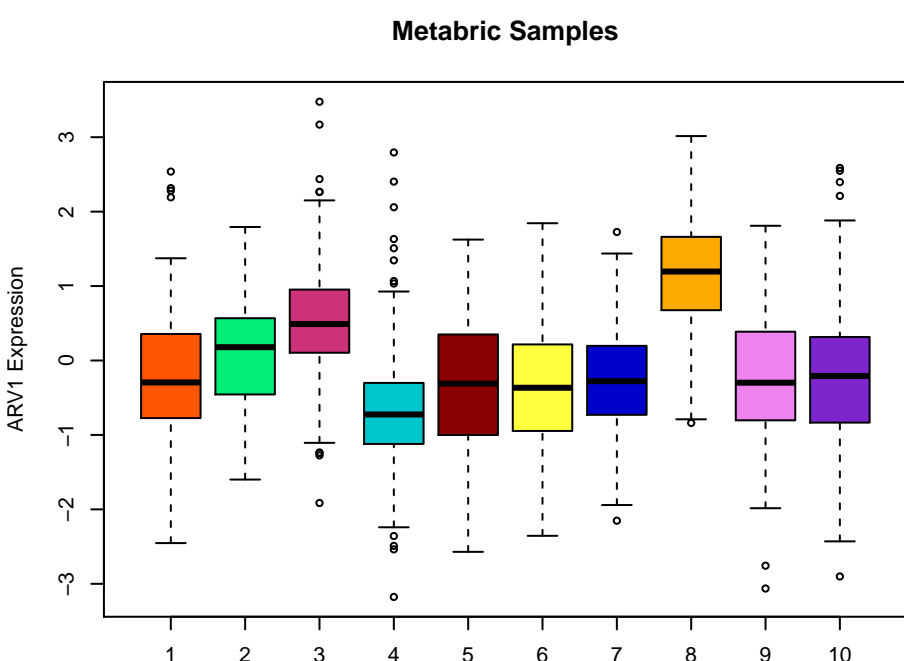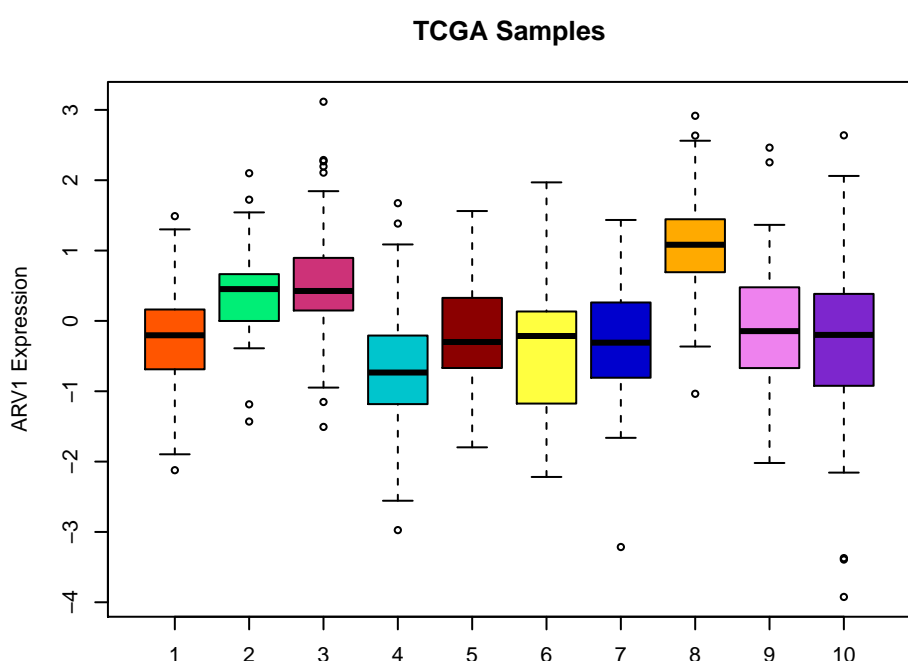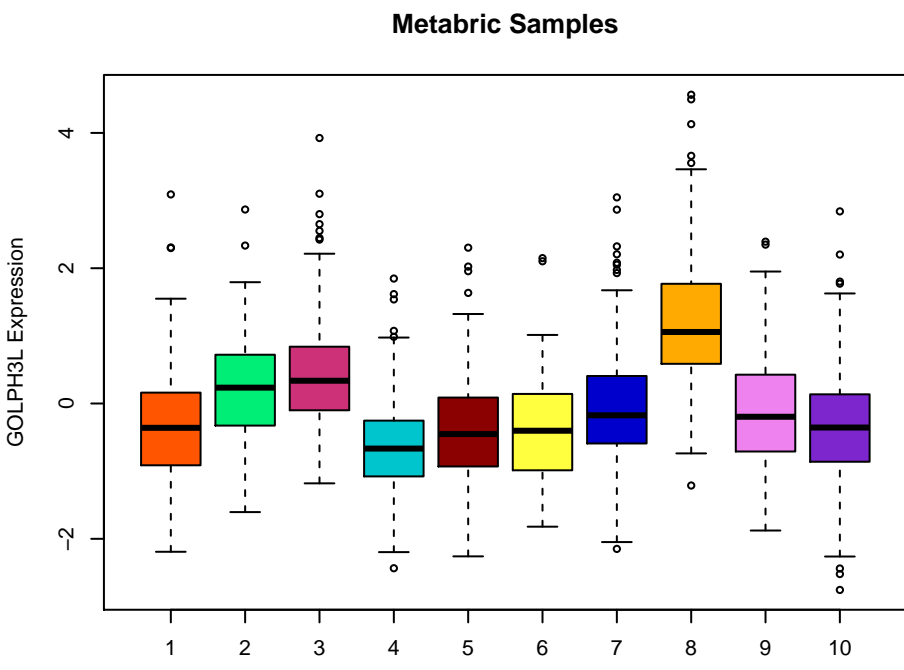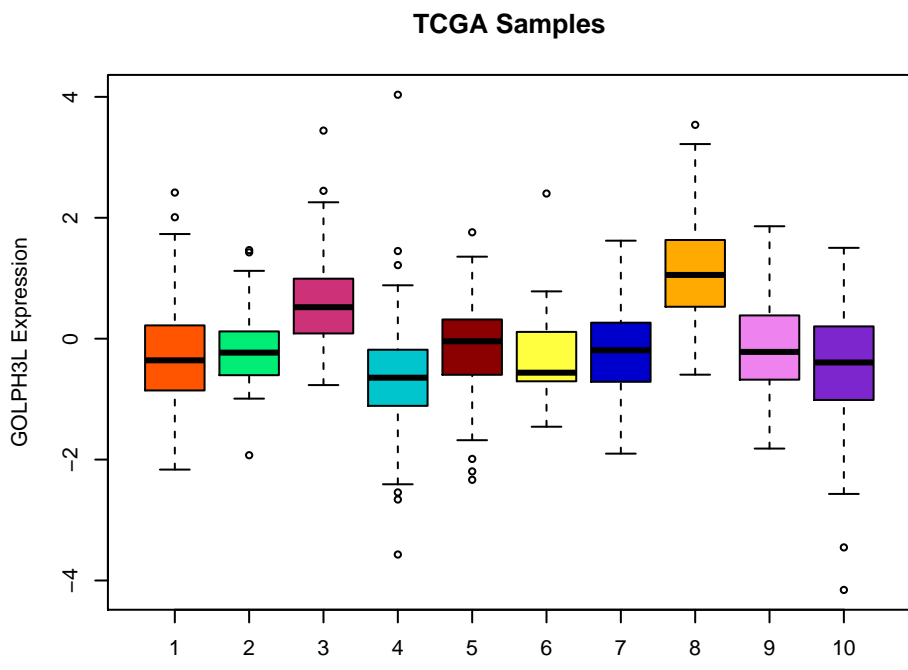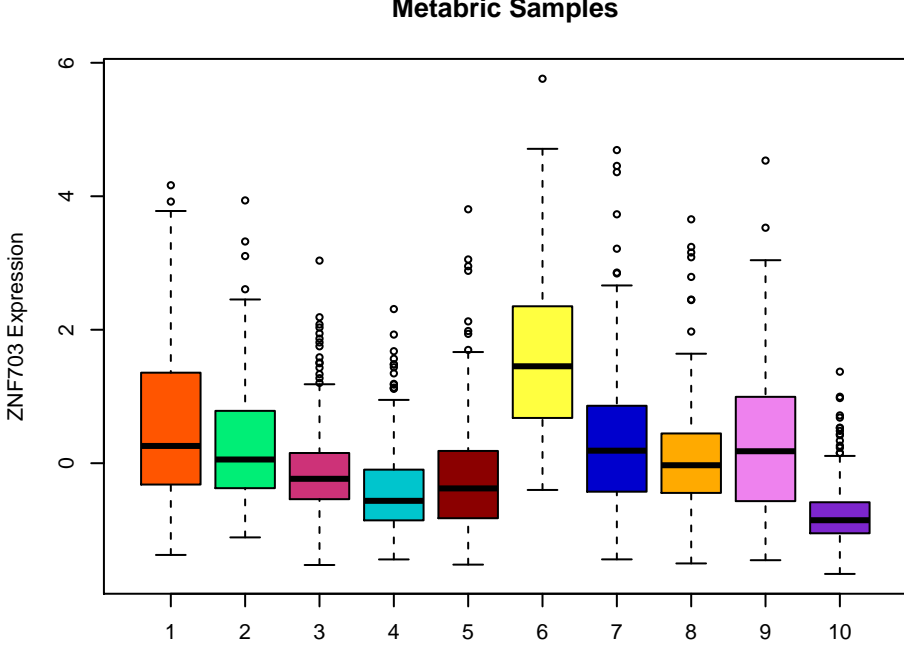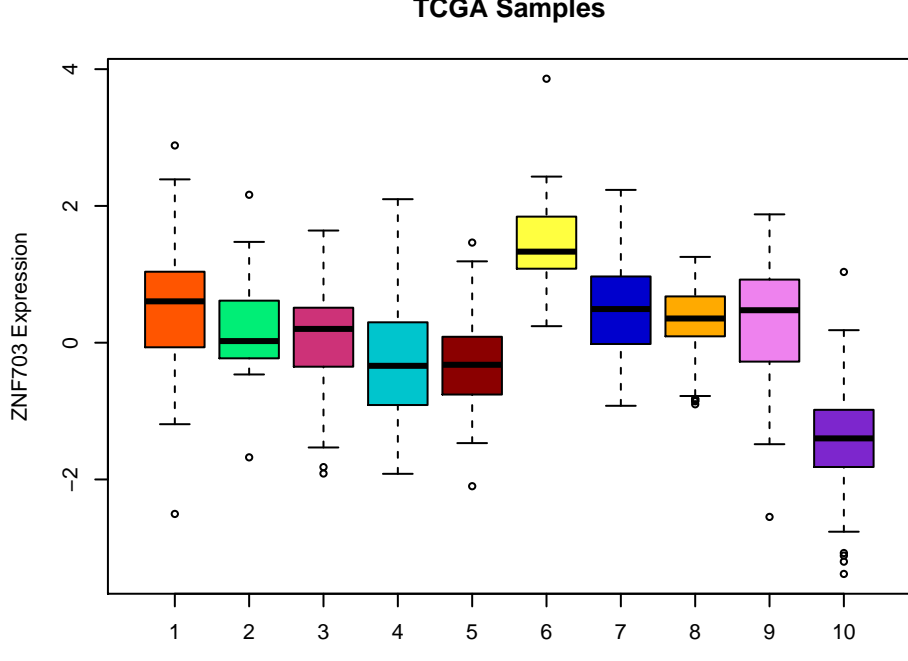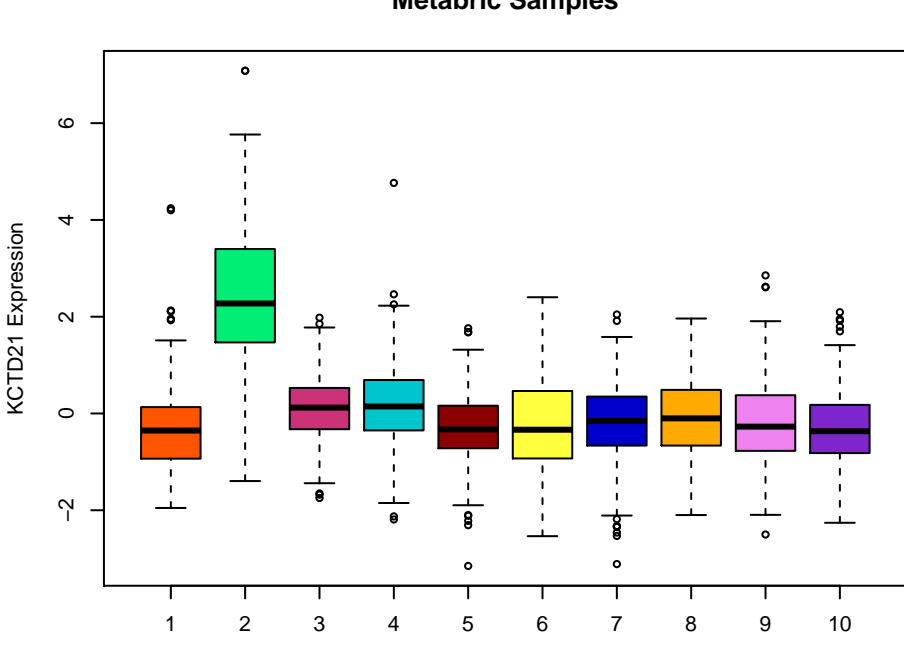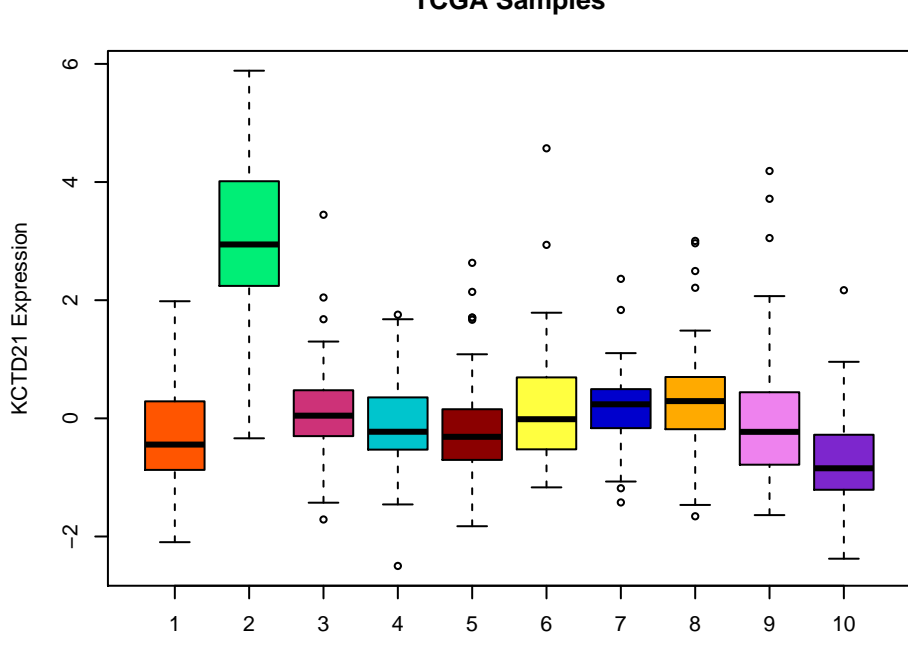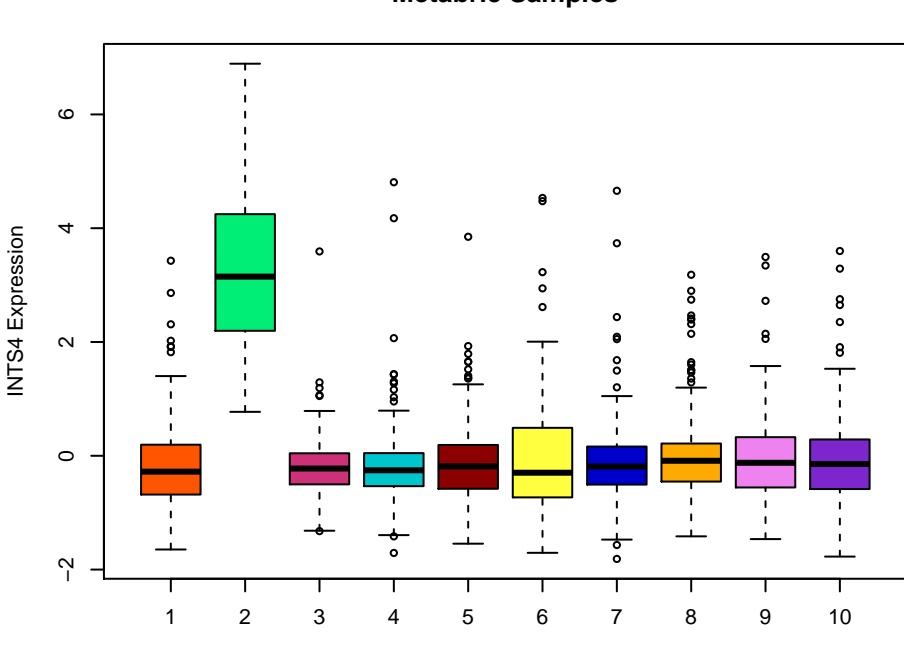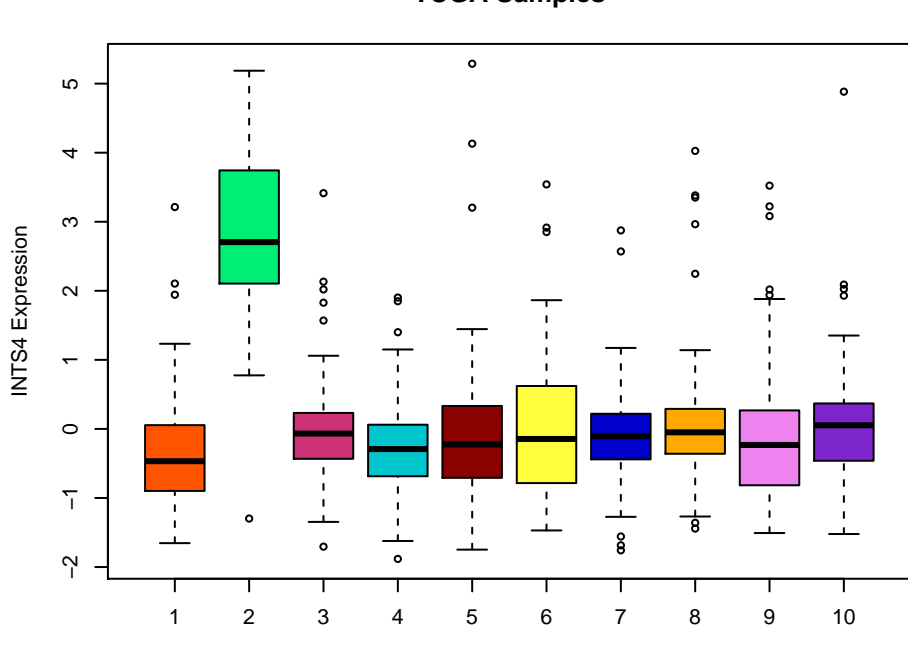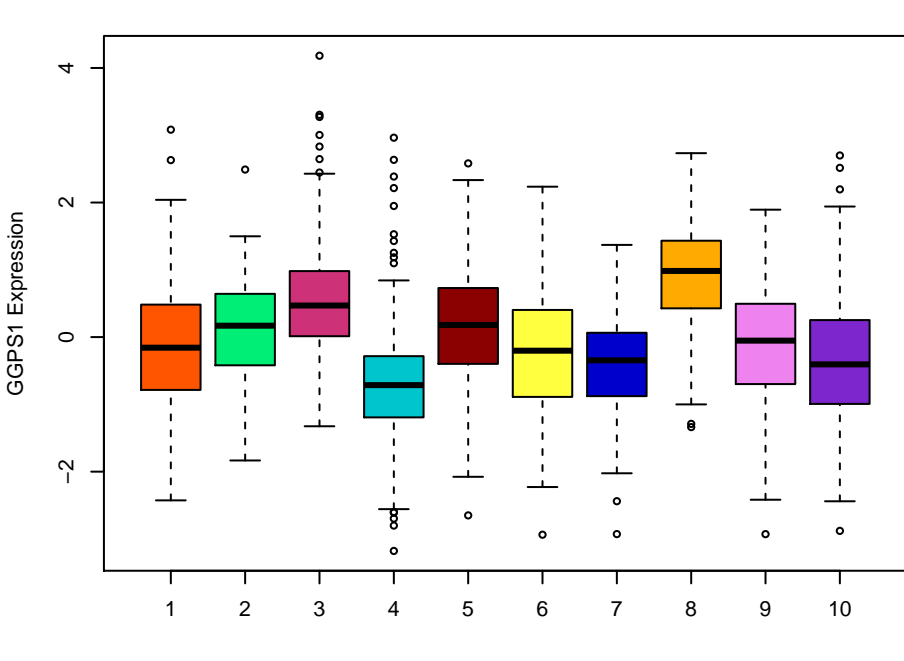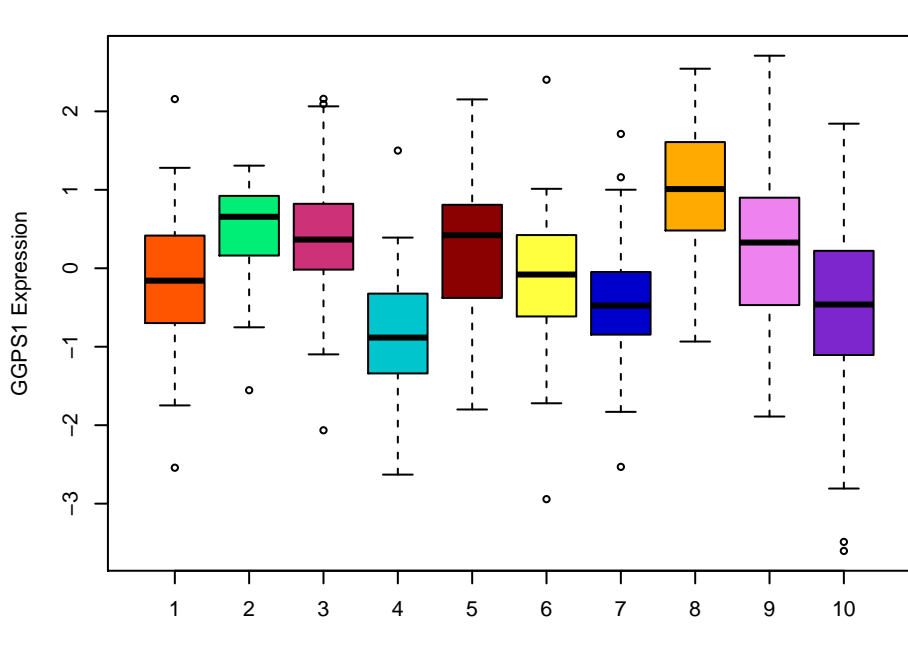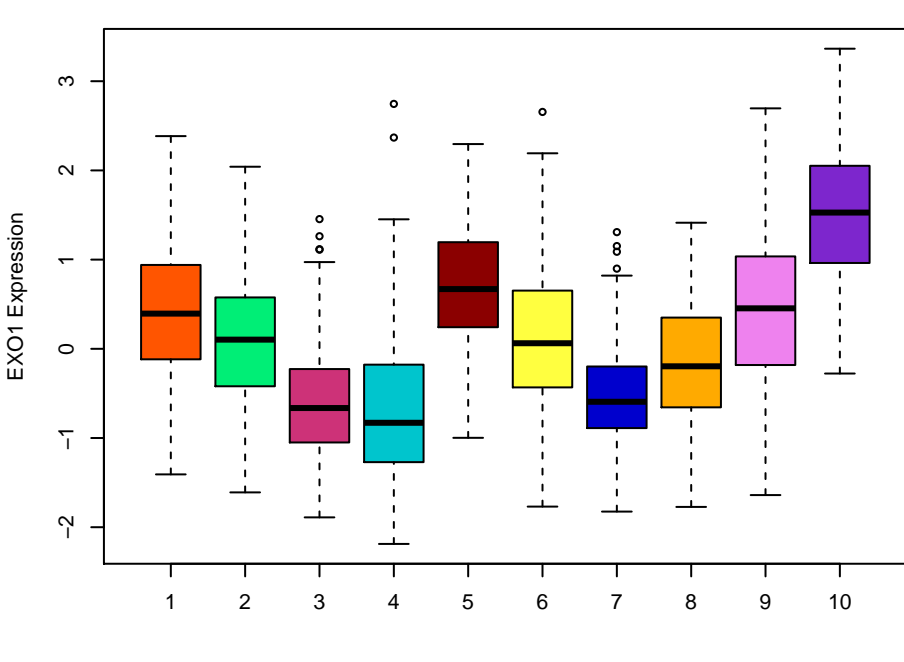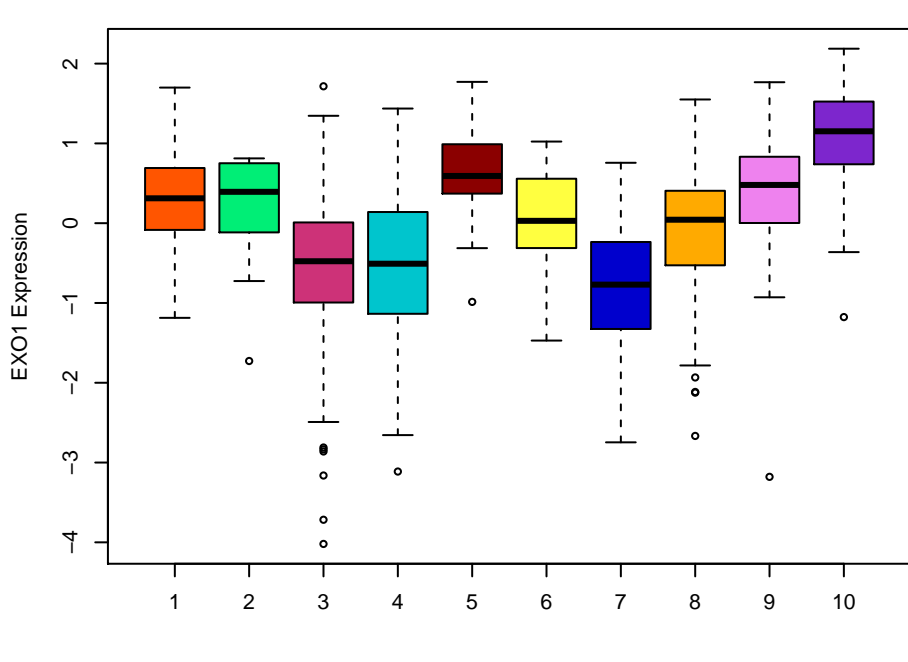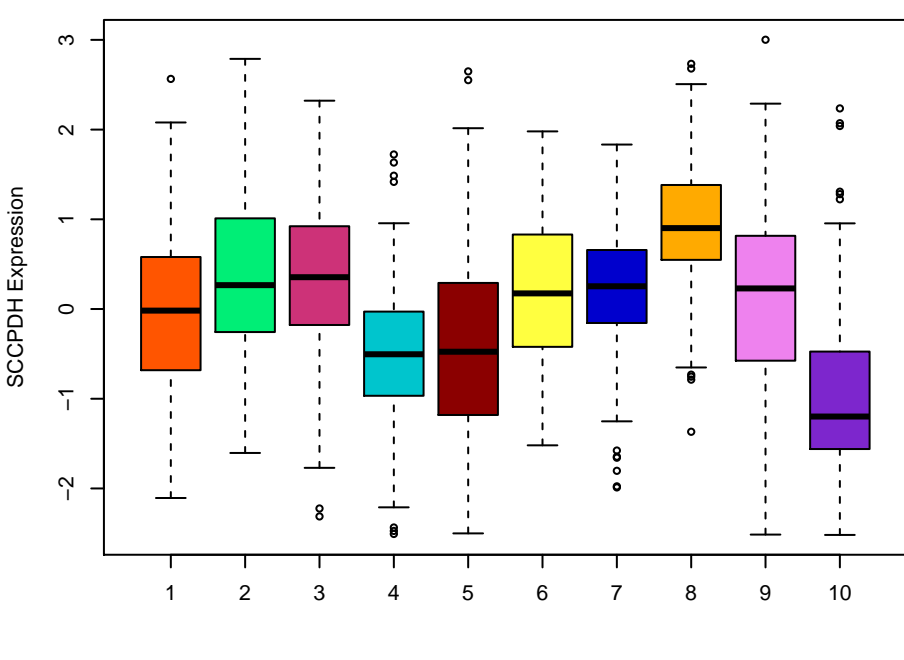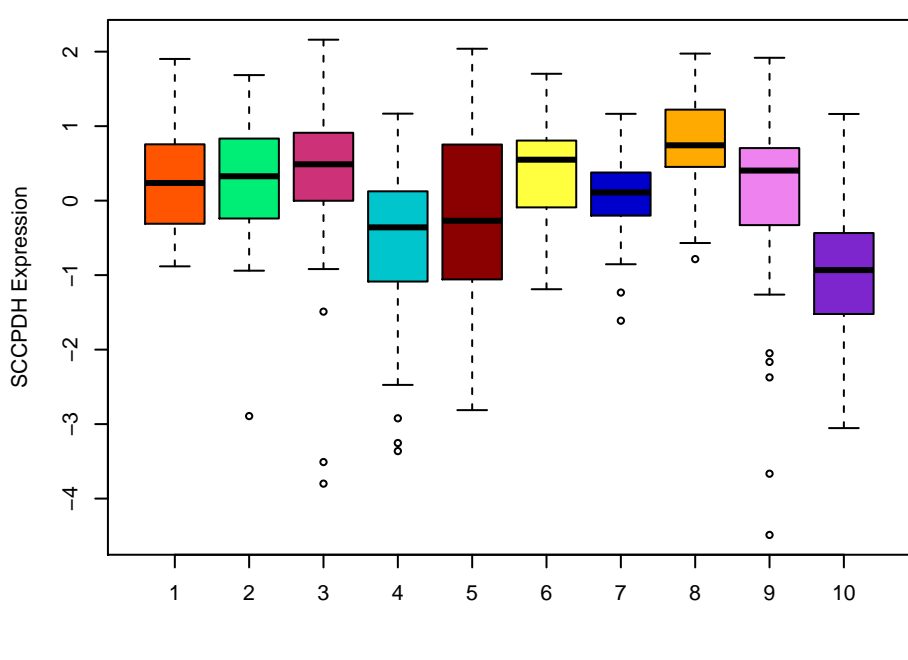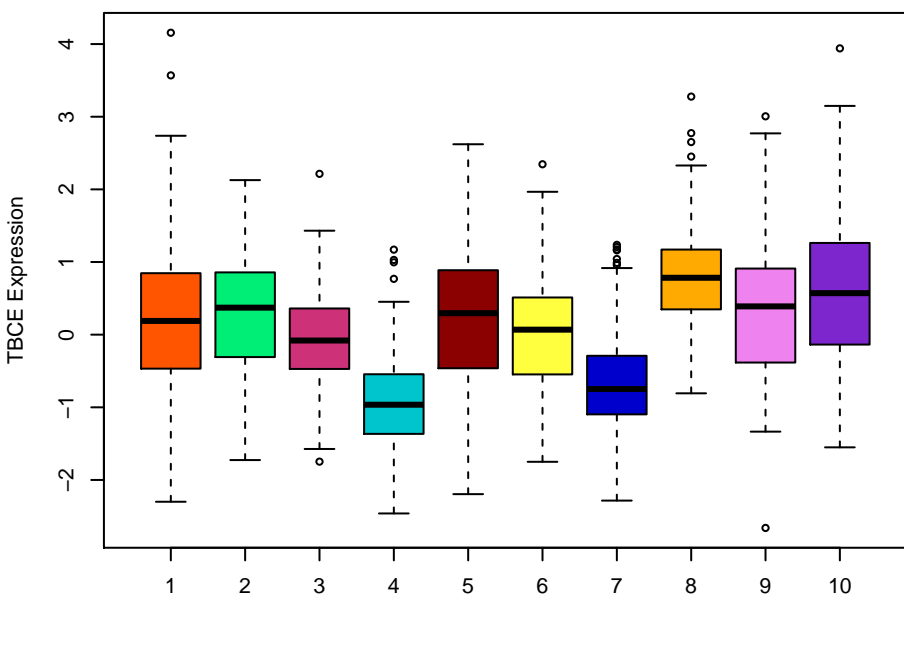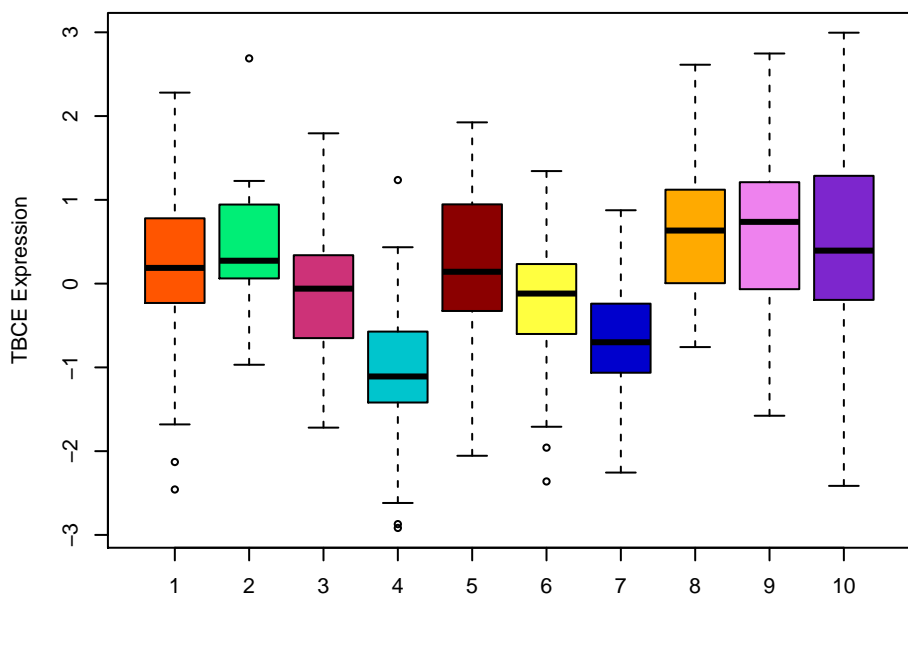

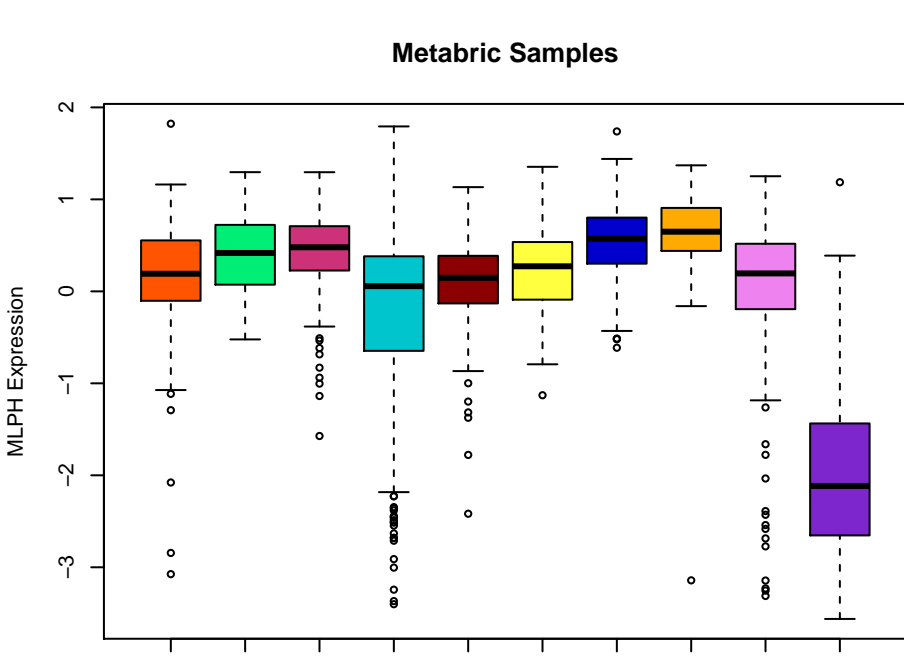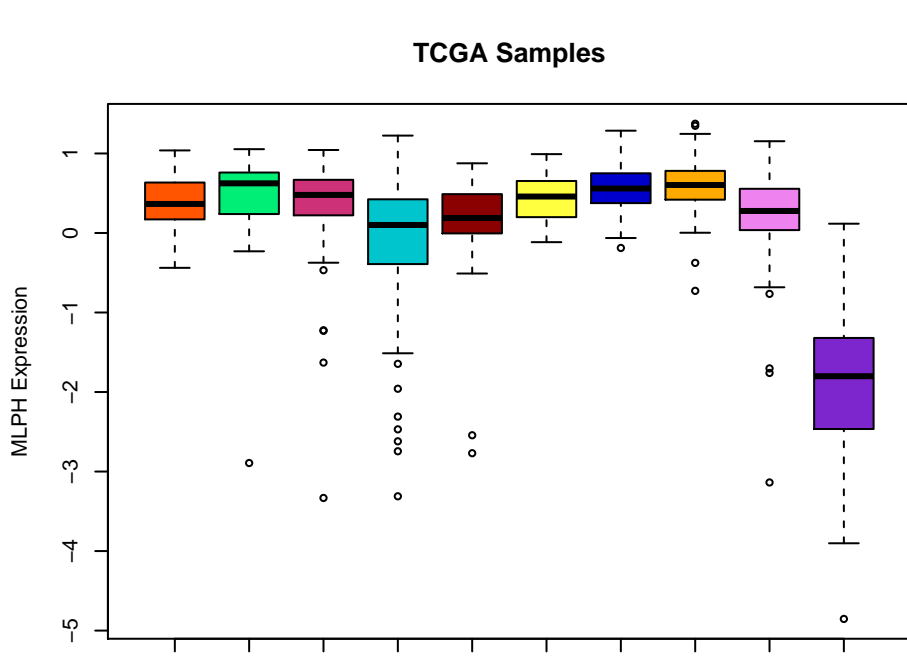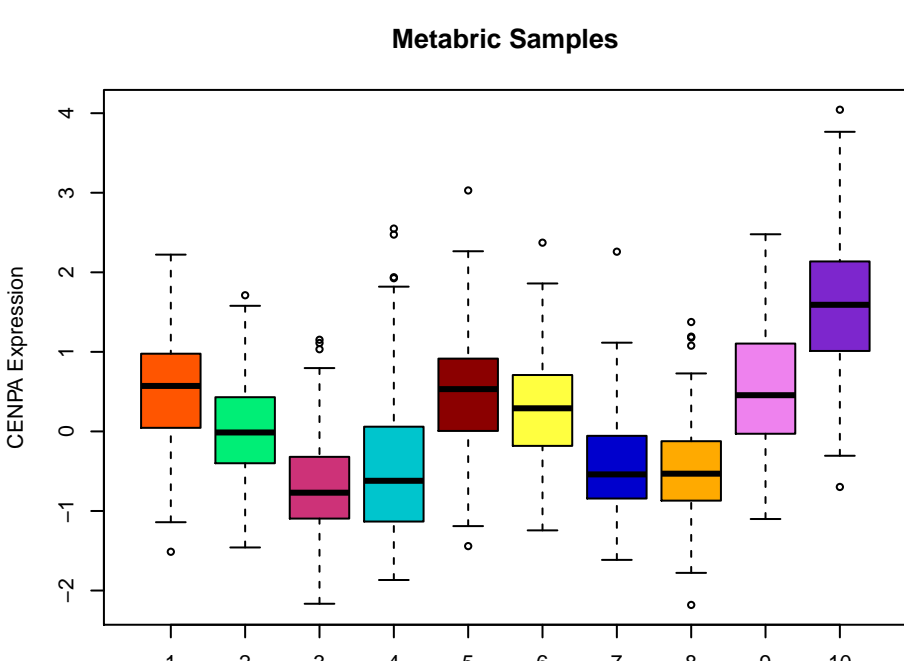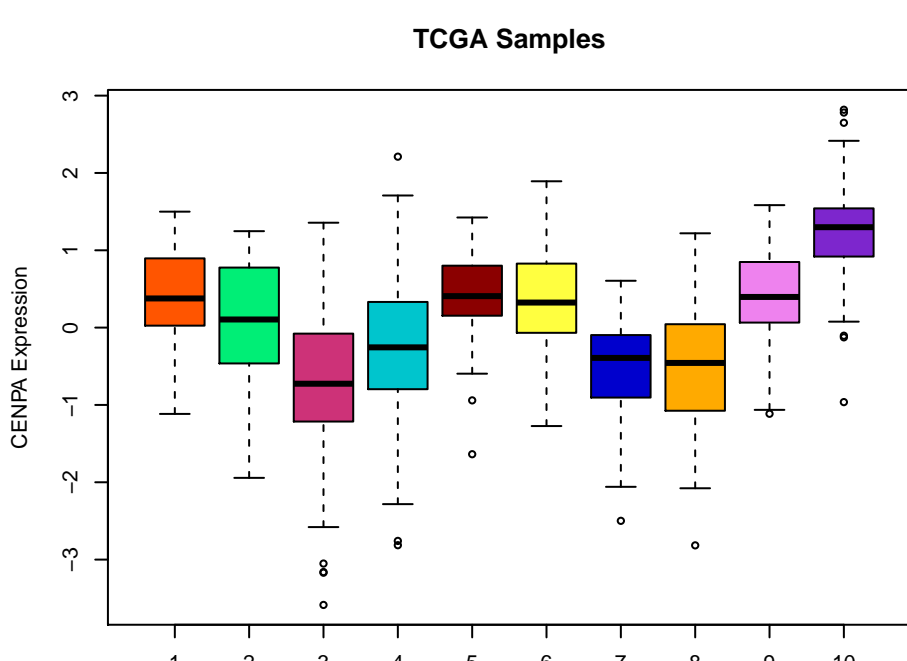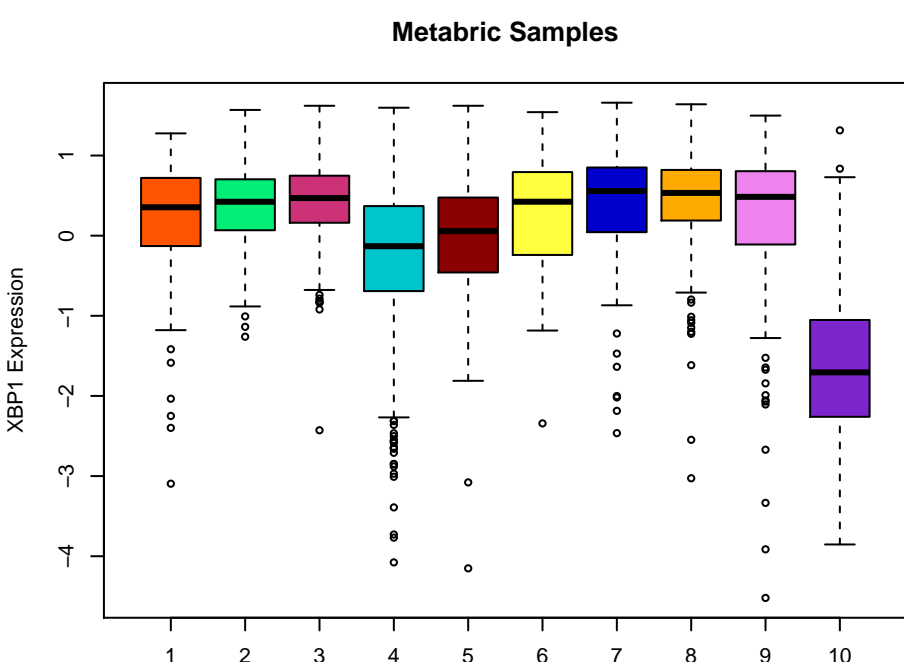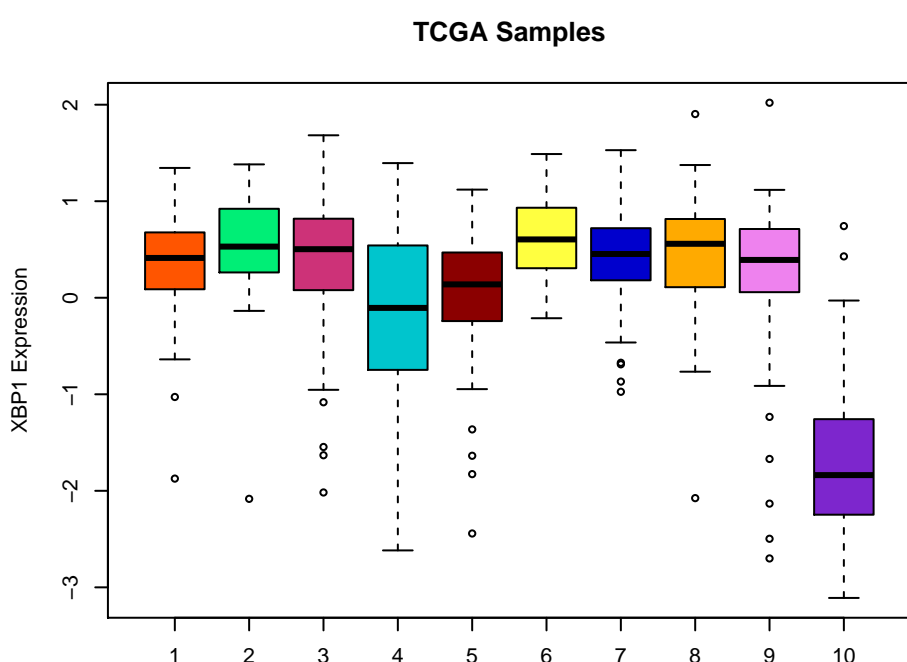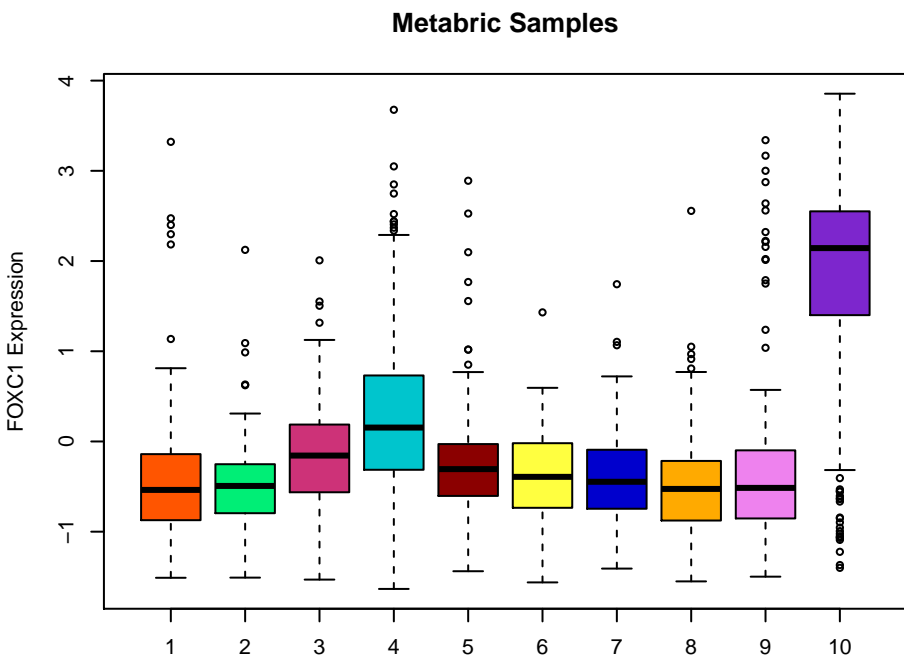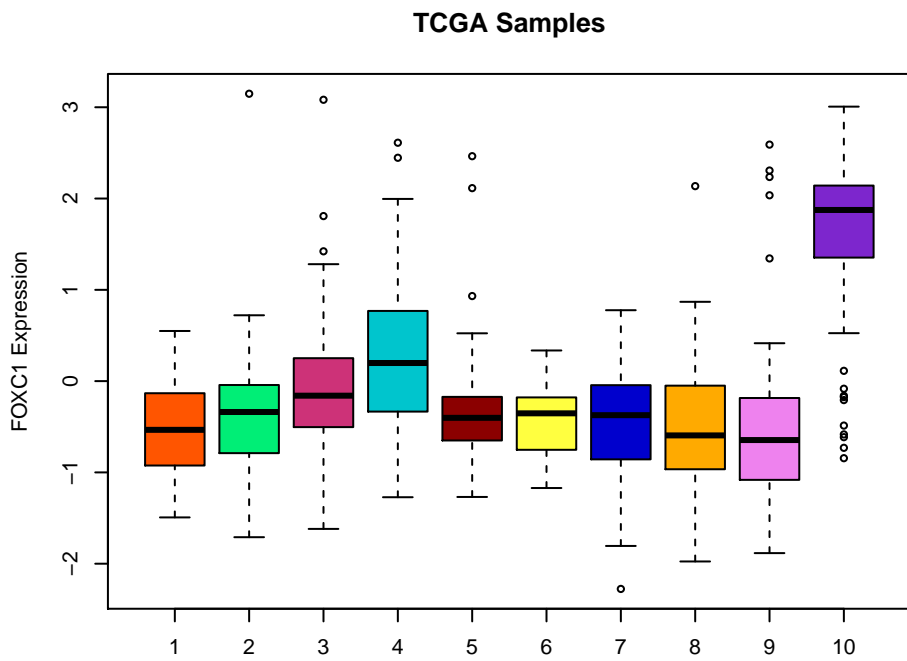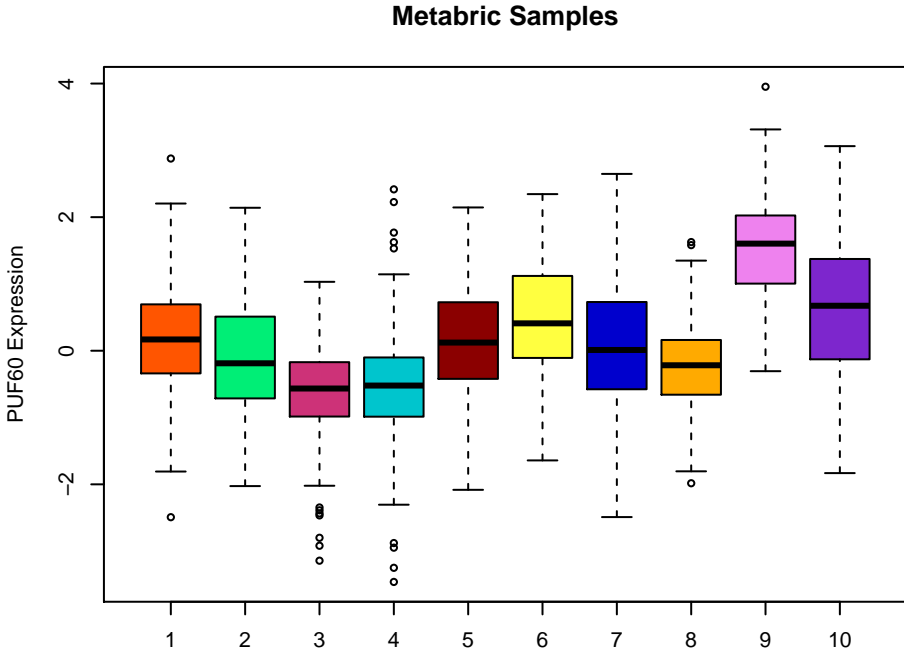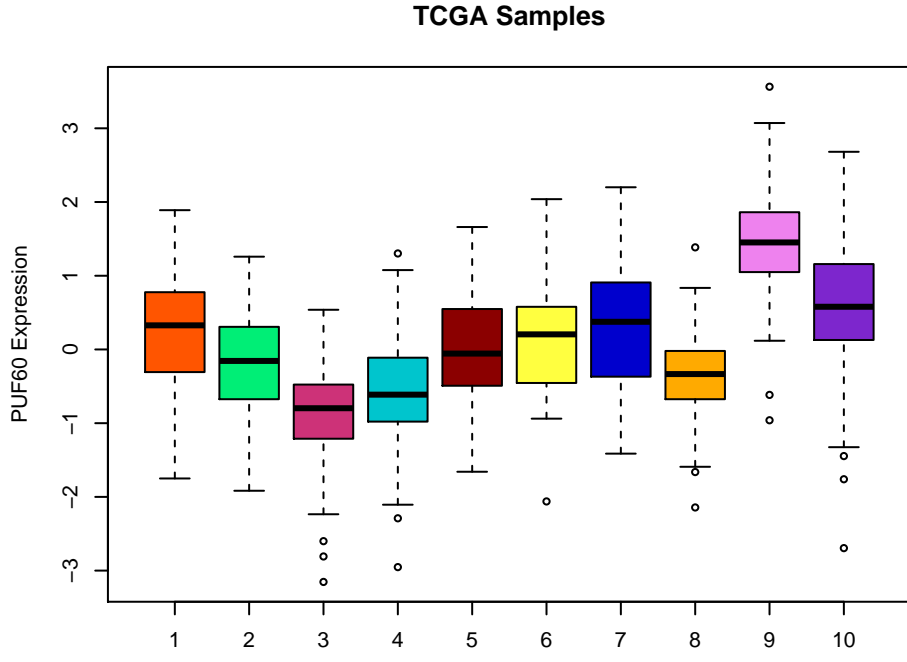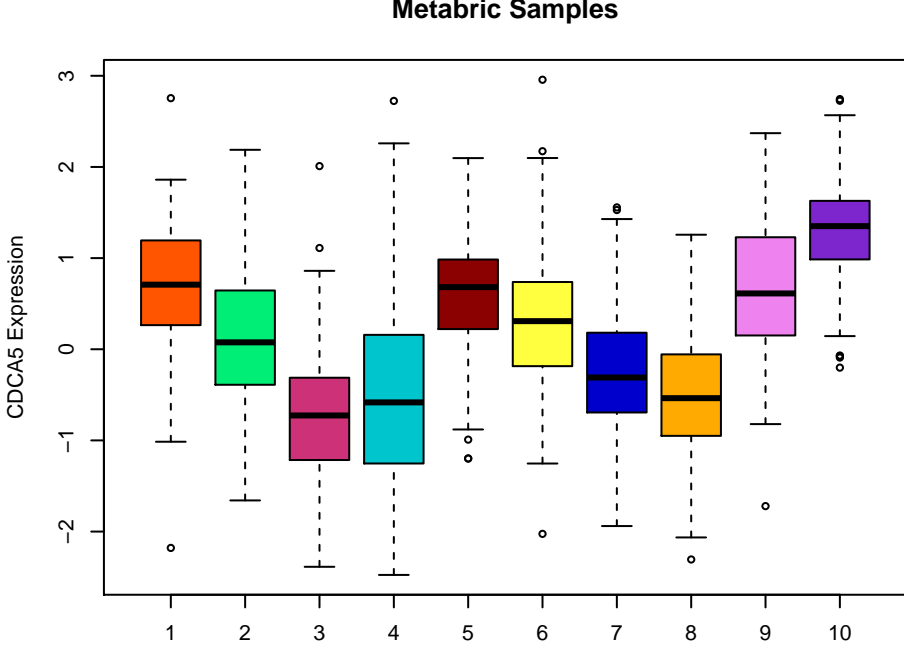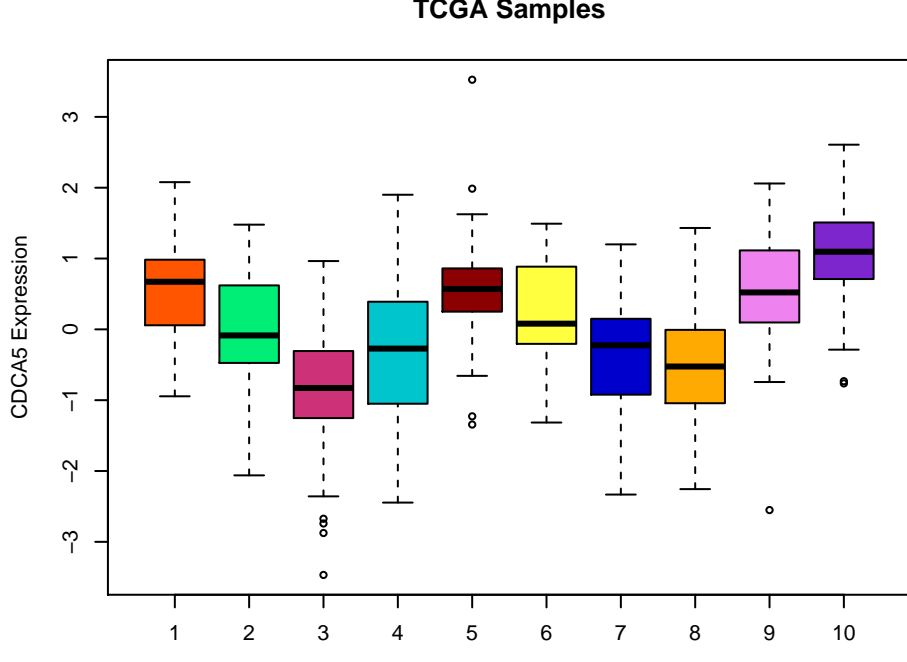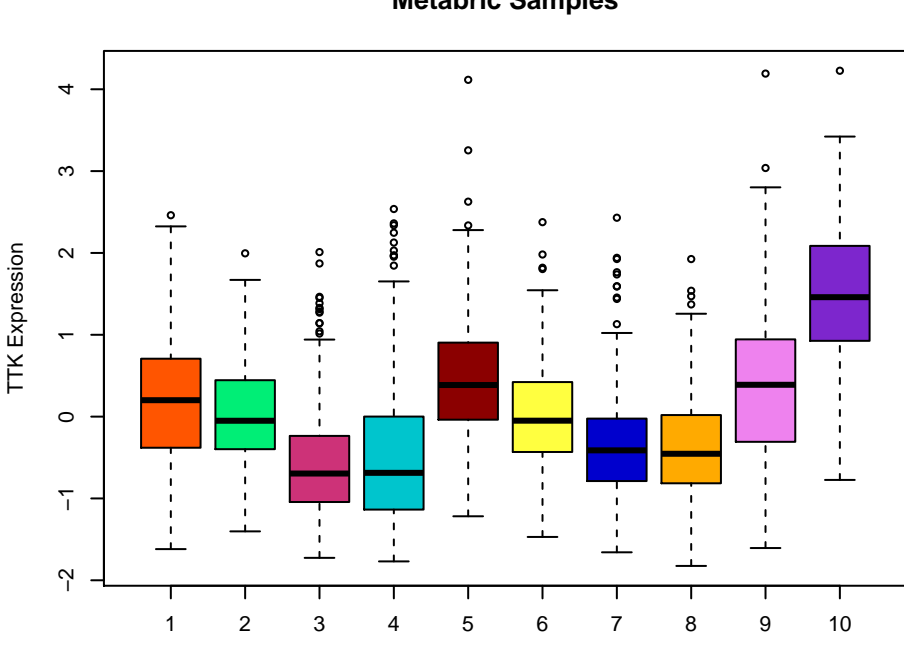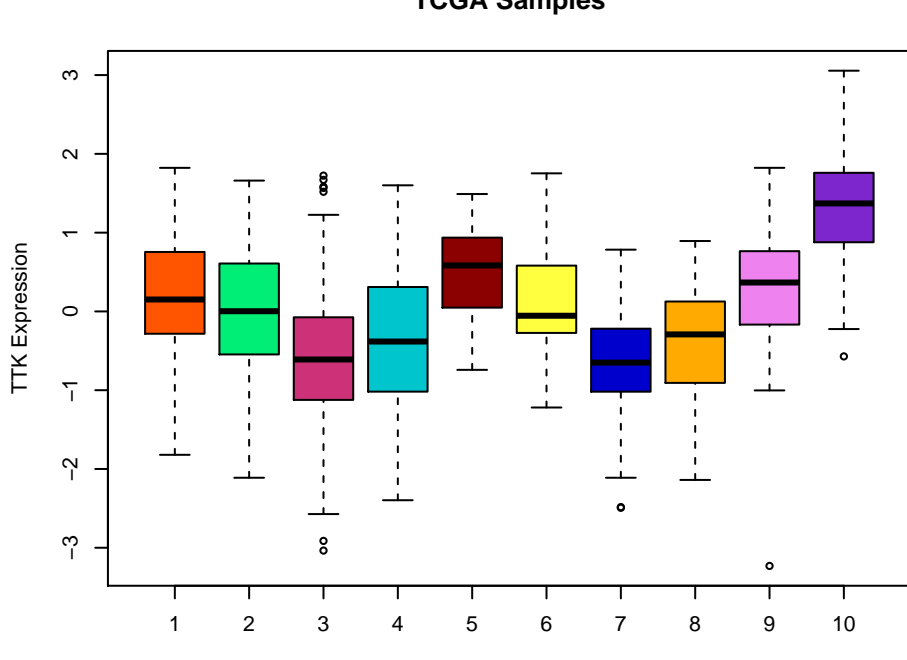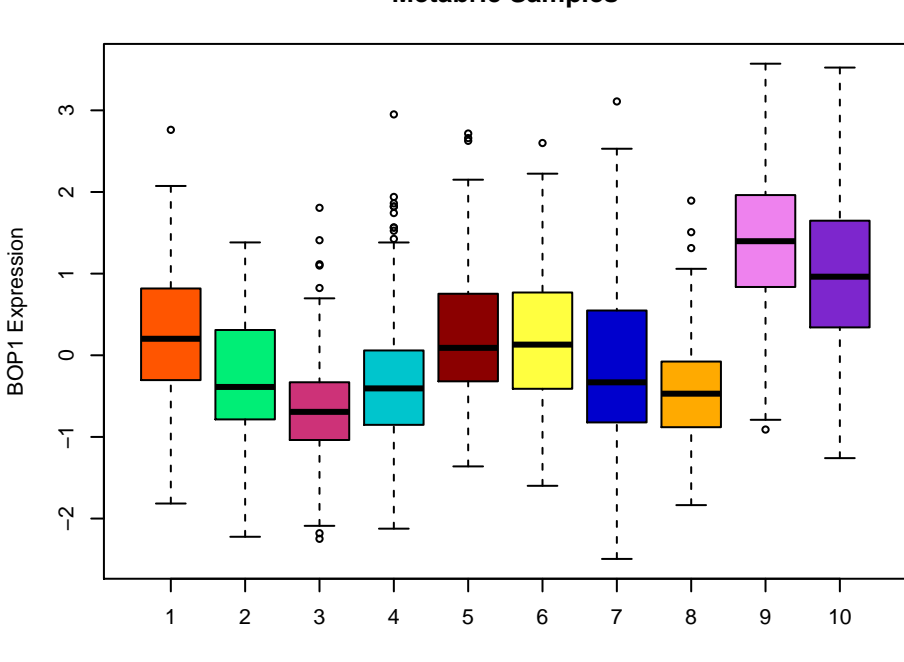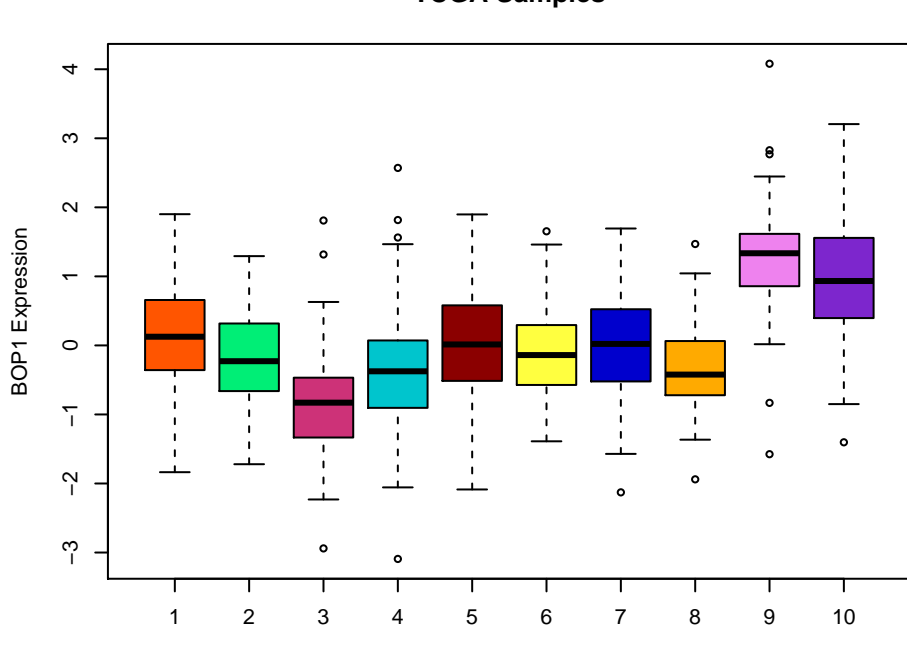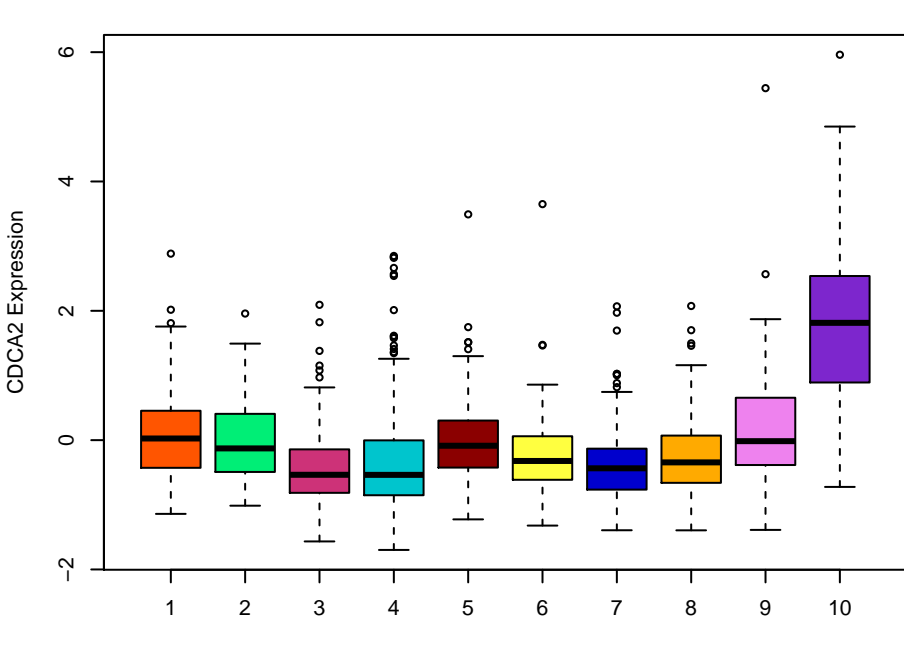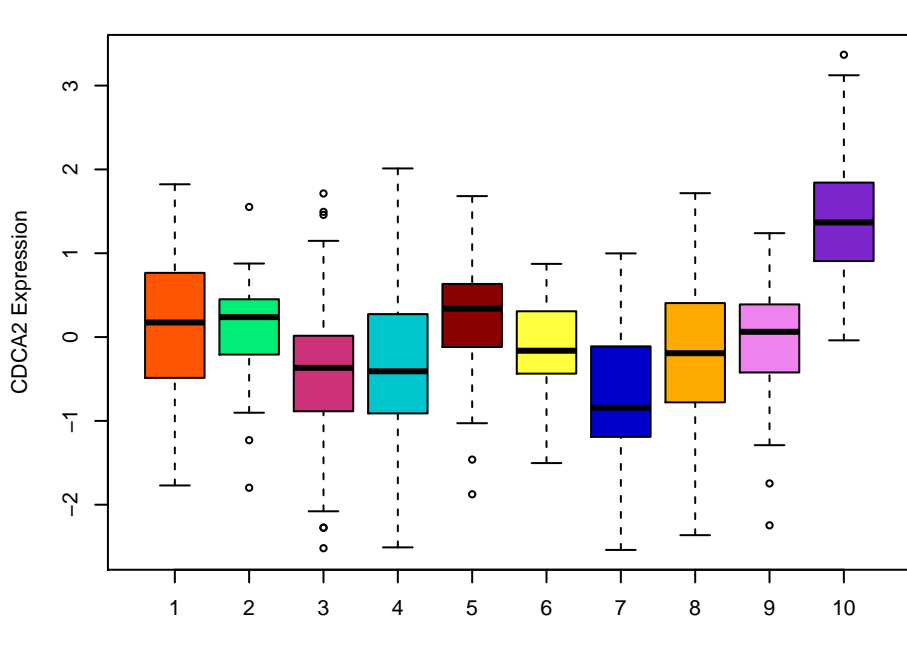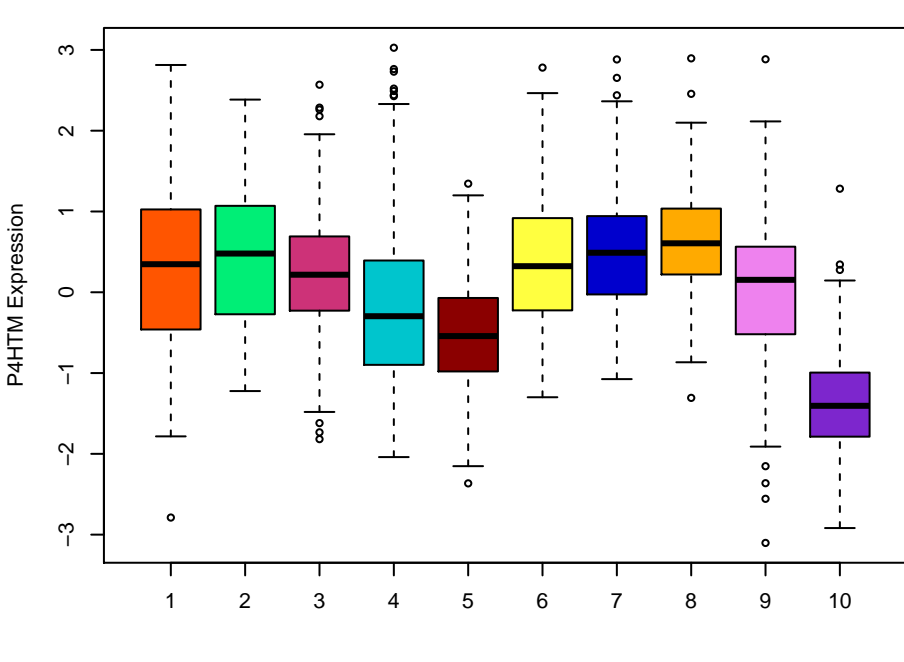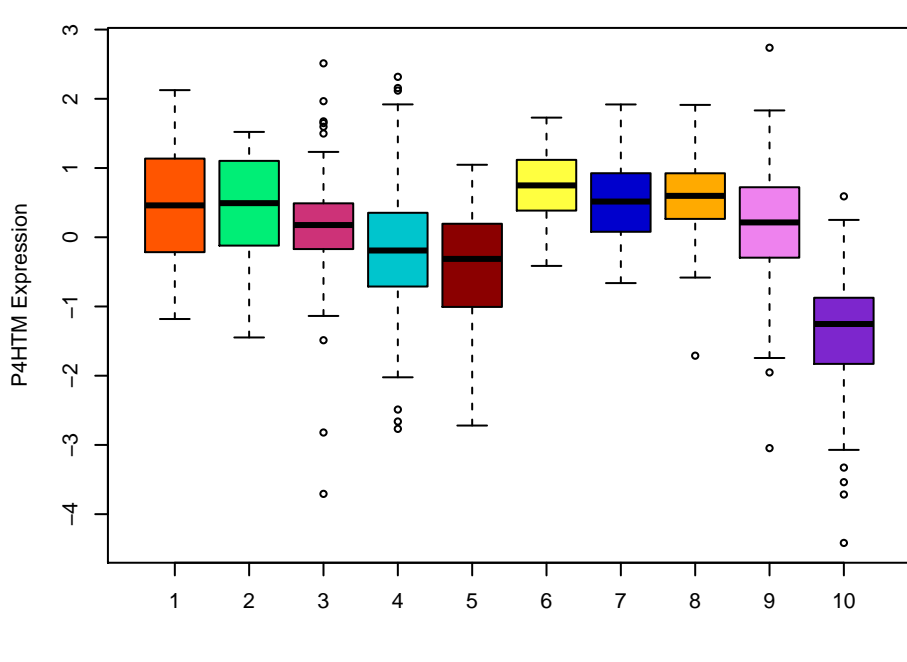

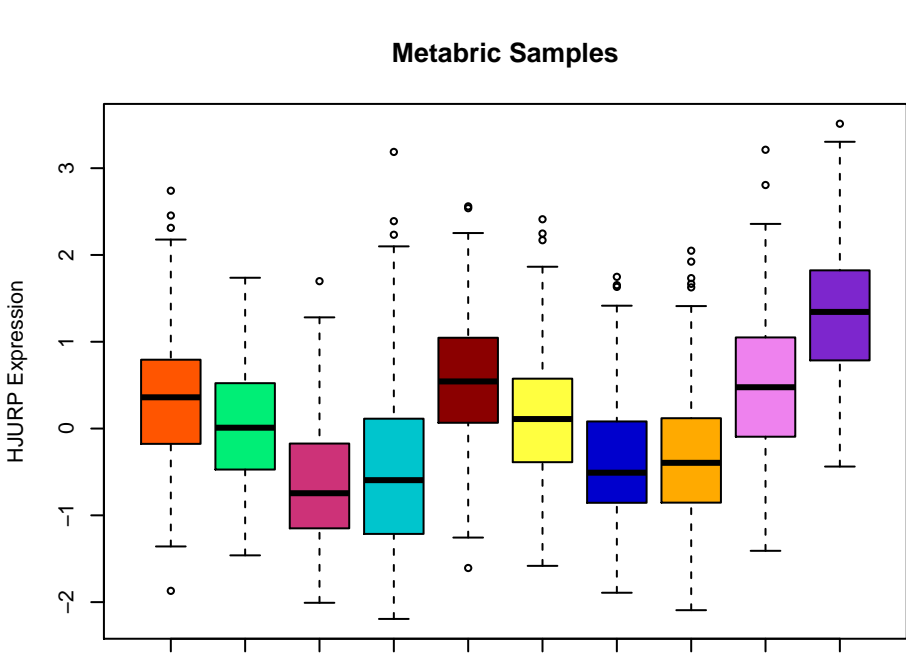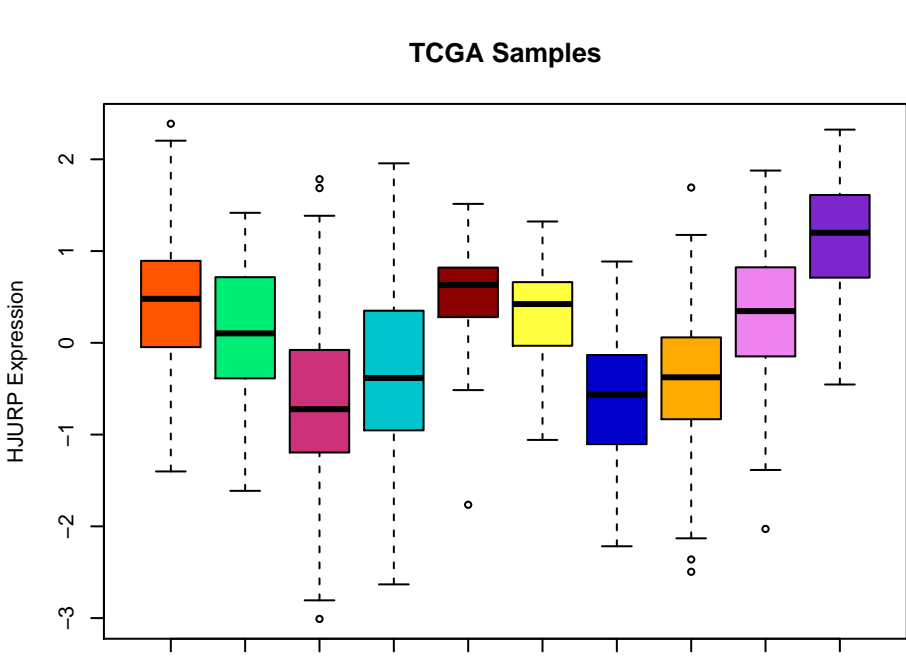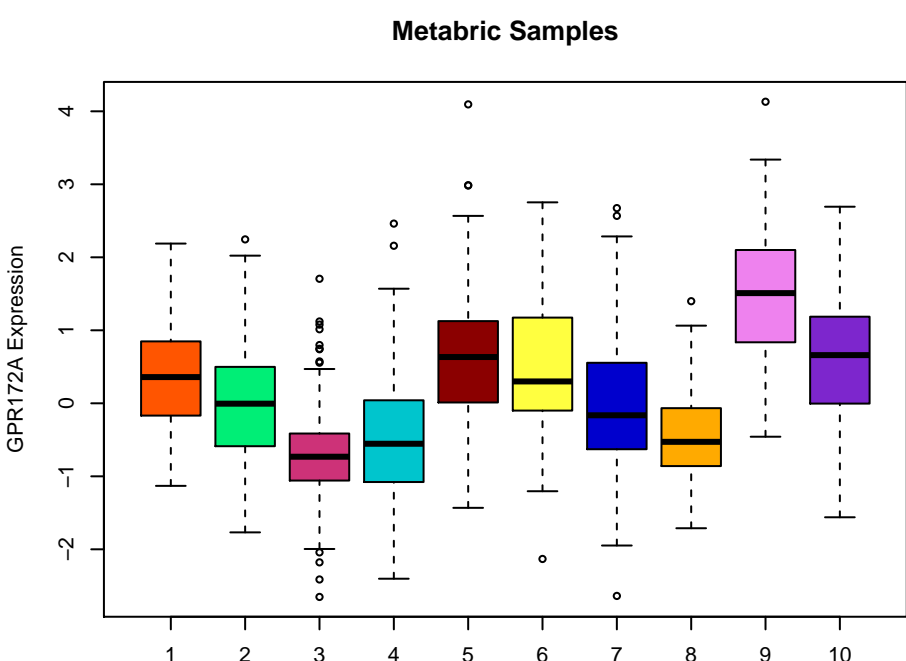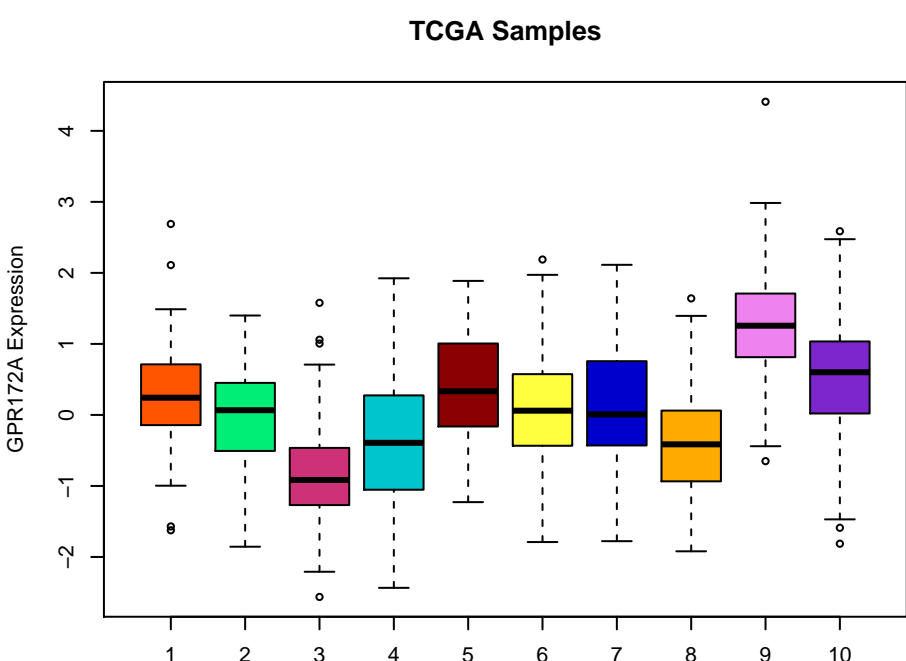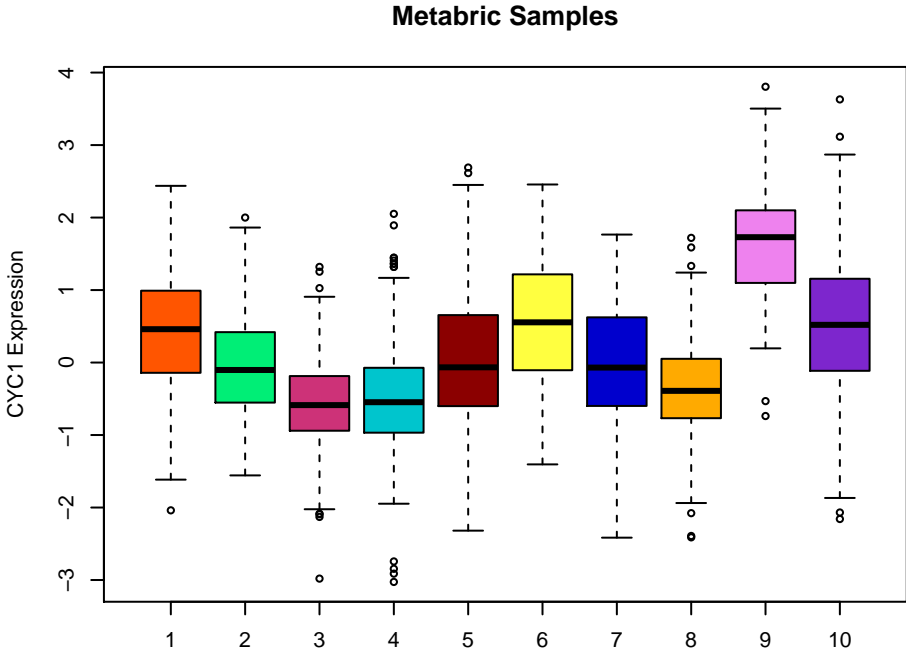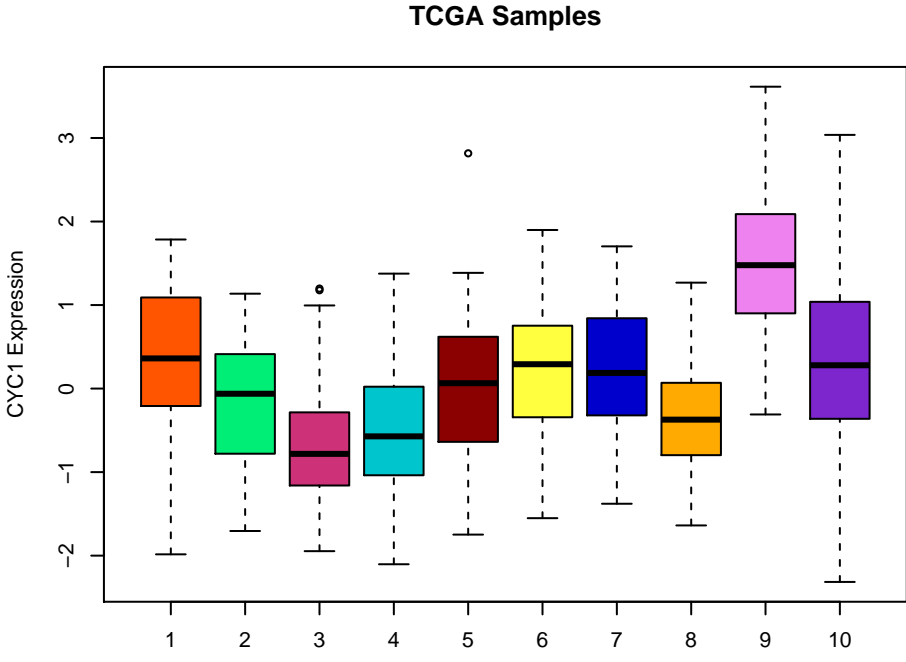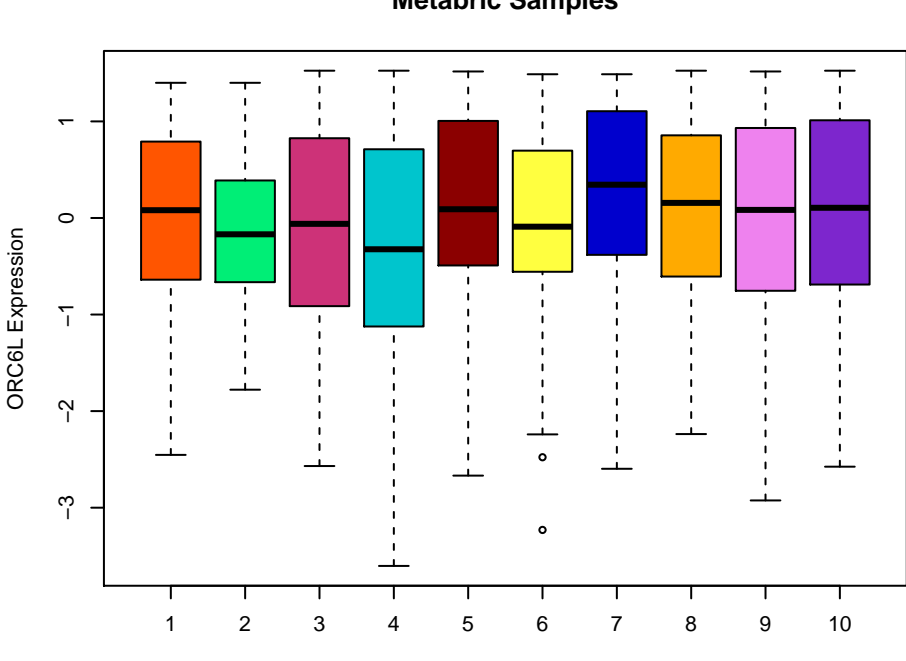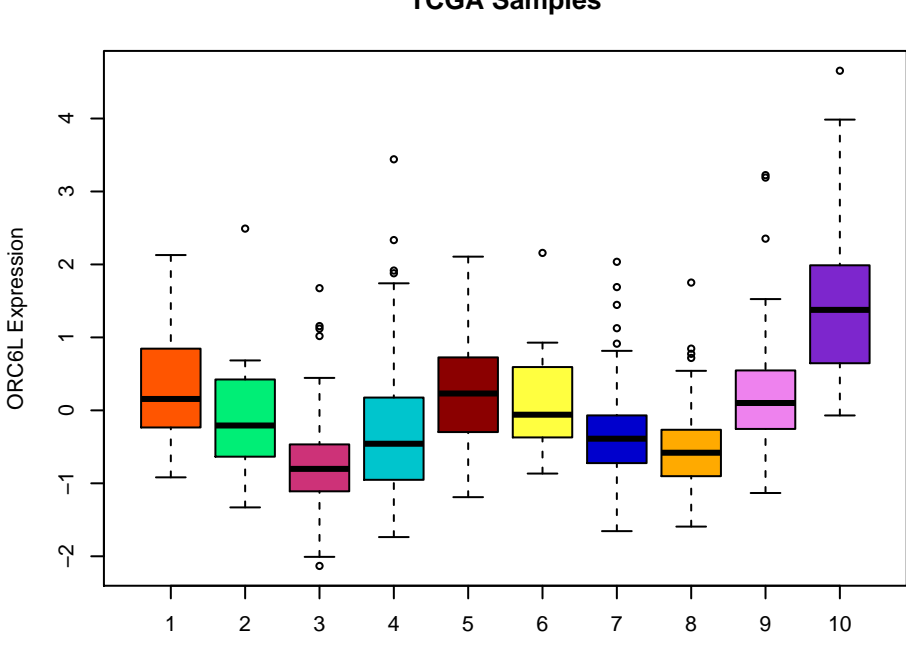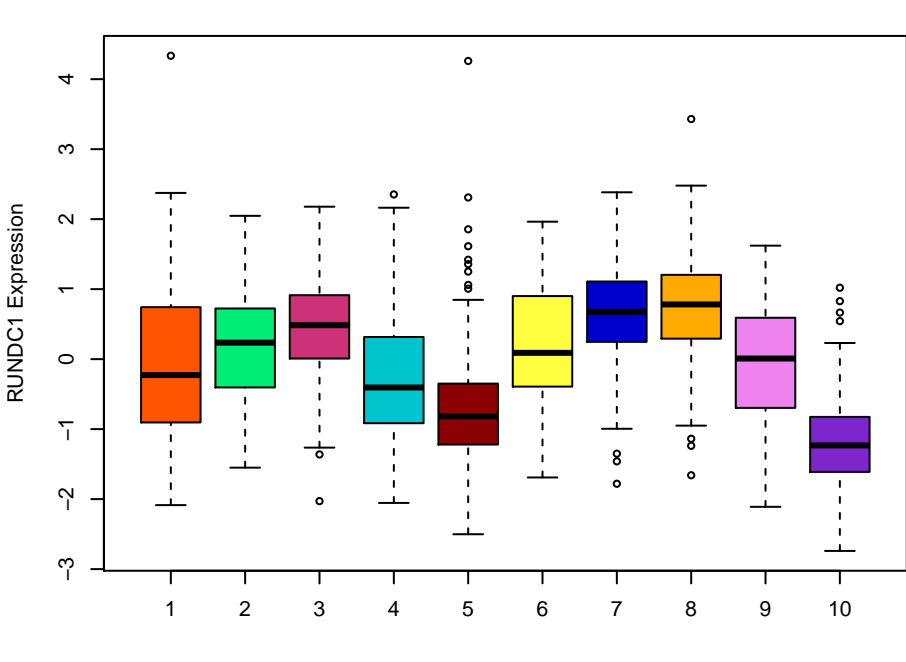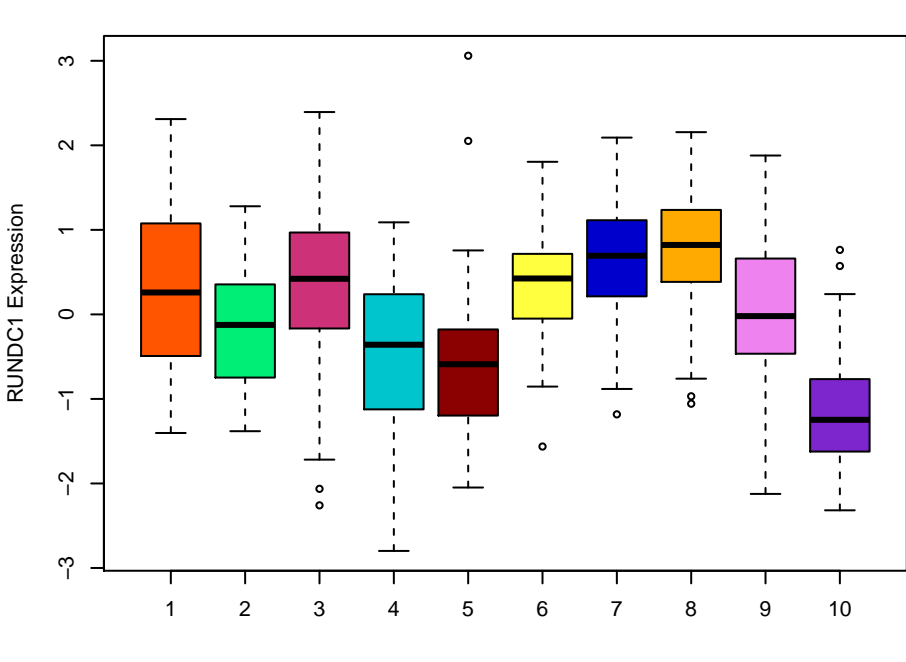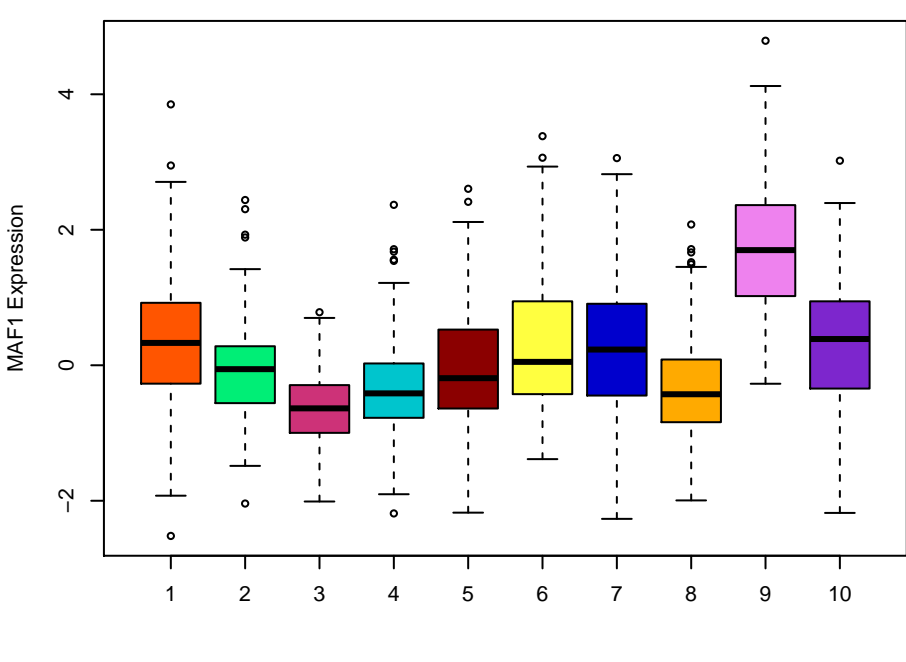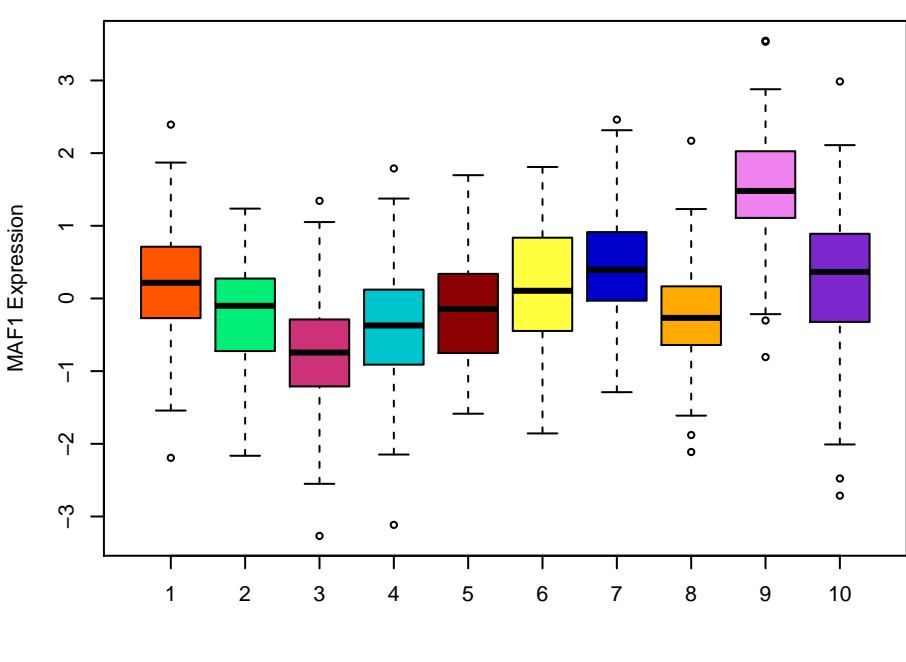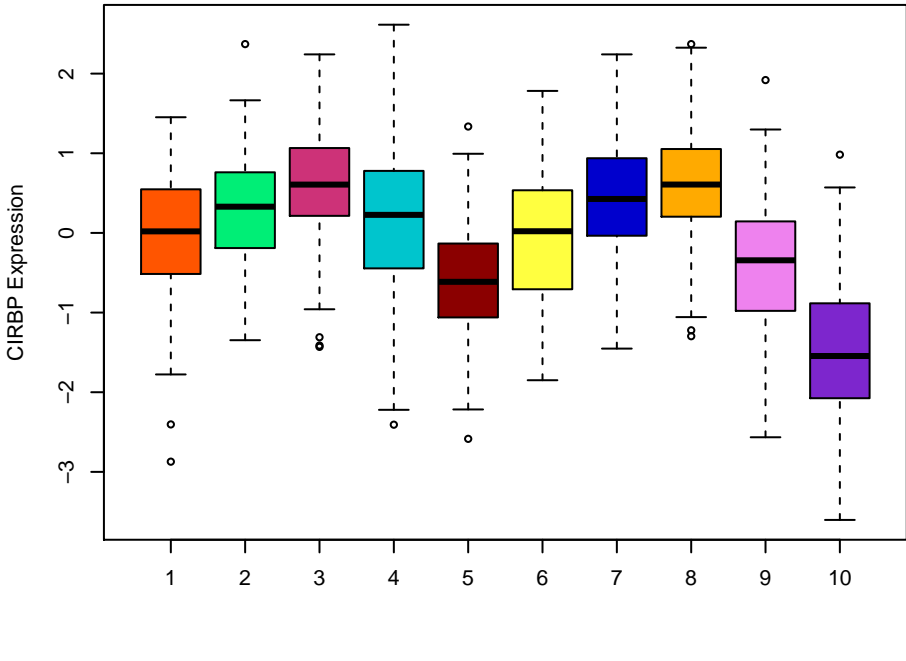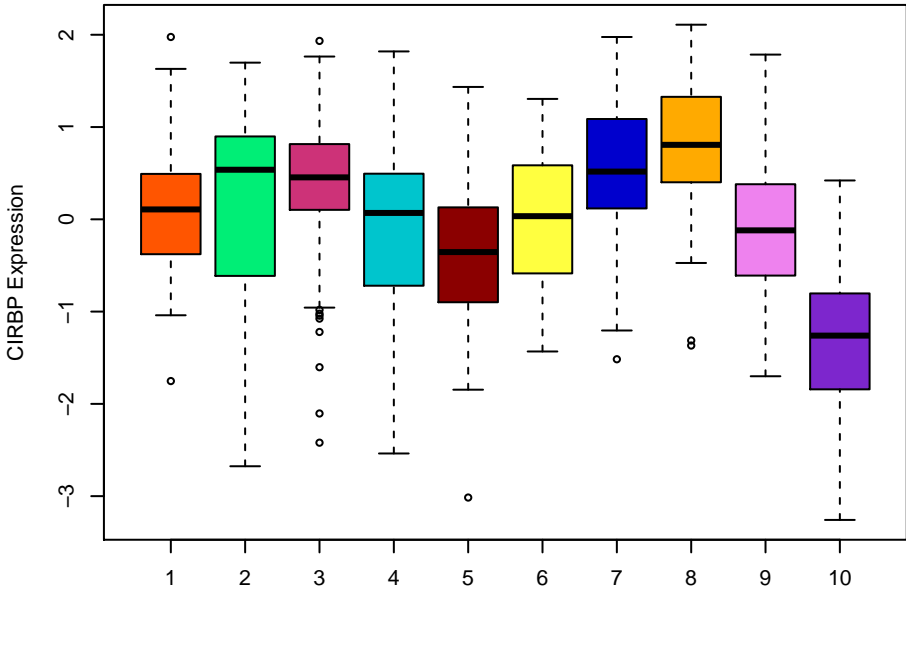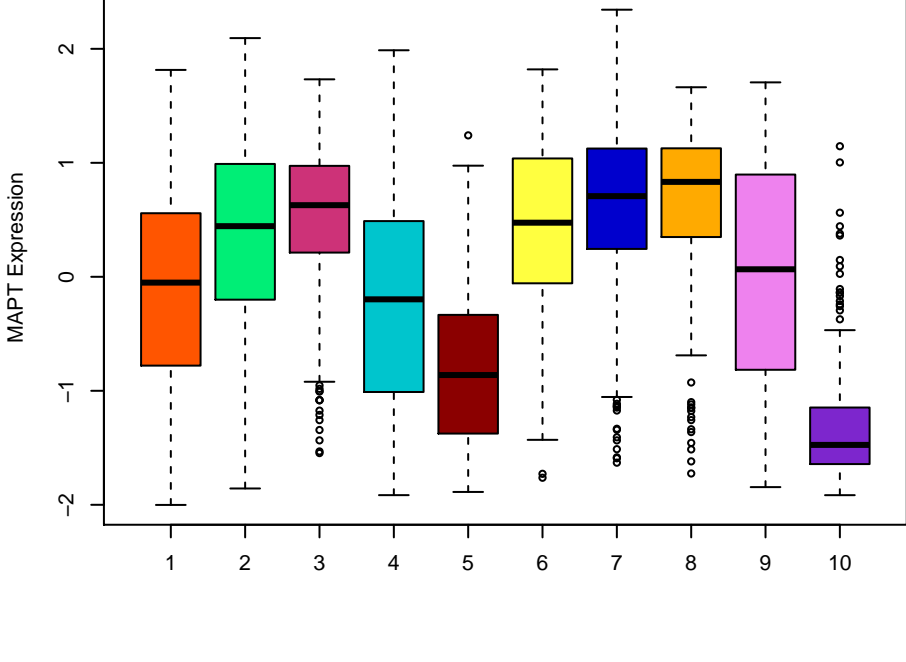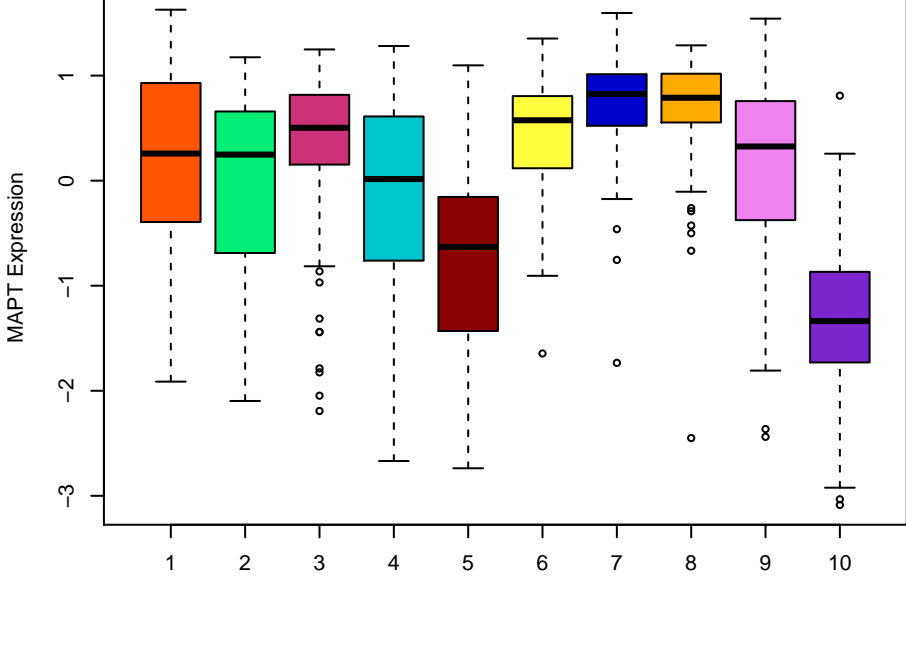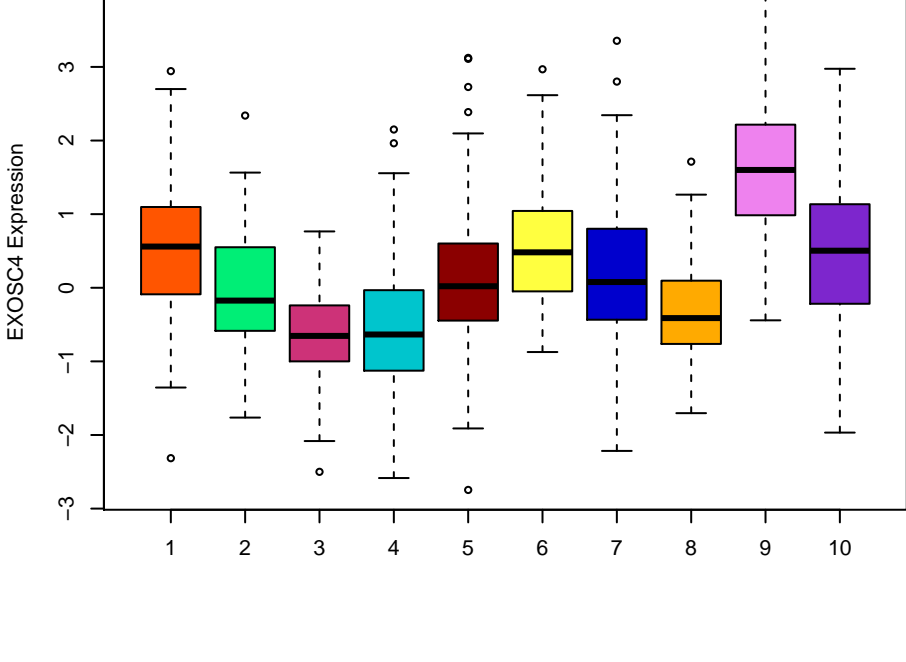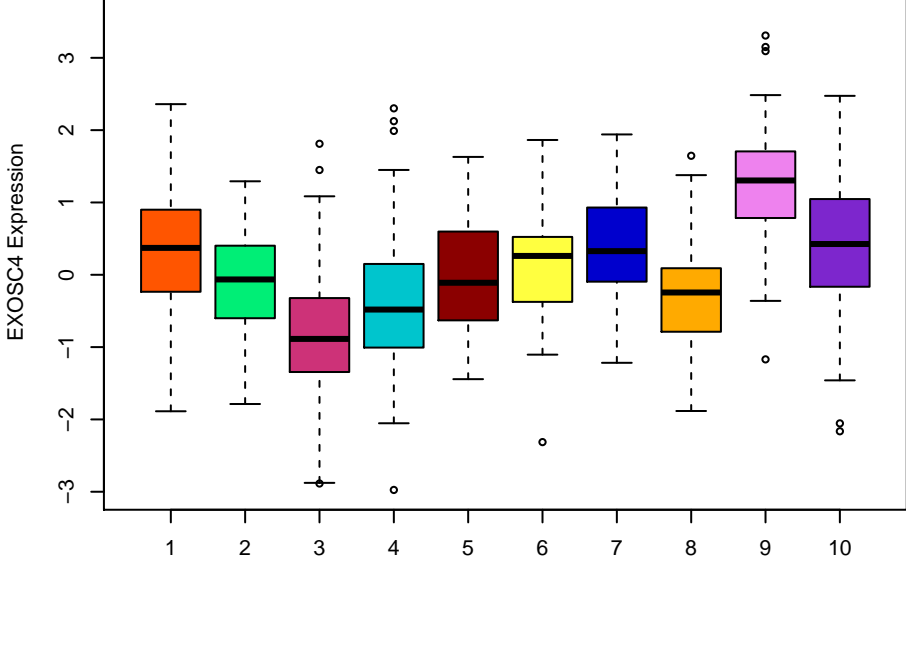

Supplement: Additional file 15: — Boxplots depicting the distribution of expression levels for samples within the METABRIC validation study and TCGA study, for top genes explained by IntClust. [file 13059_2014_431_MOESM15_ESM.pdf]
