# Supplementary material for: Genome-driven integrated classification of breast cancer validated in over 7,500 samples
Source: Genome Biol. 2014 Aug 28;15(8):431. doi: 10.1186/s13059-014-0431-1 (PMC4166472; doi:10.1186/s13059-014-0431-1)

Additional file 7 – PAM50 and SCMGENE subtyping of cell lines

Weighted scatter plots and cross tabulations of PAM50 and SCMGENE subtypes of cell lines according to dataset (Sanger COSMIC and CCLE).

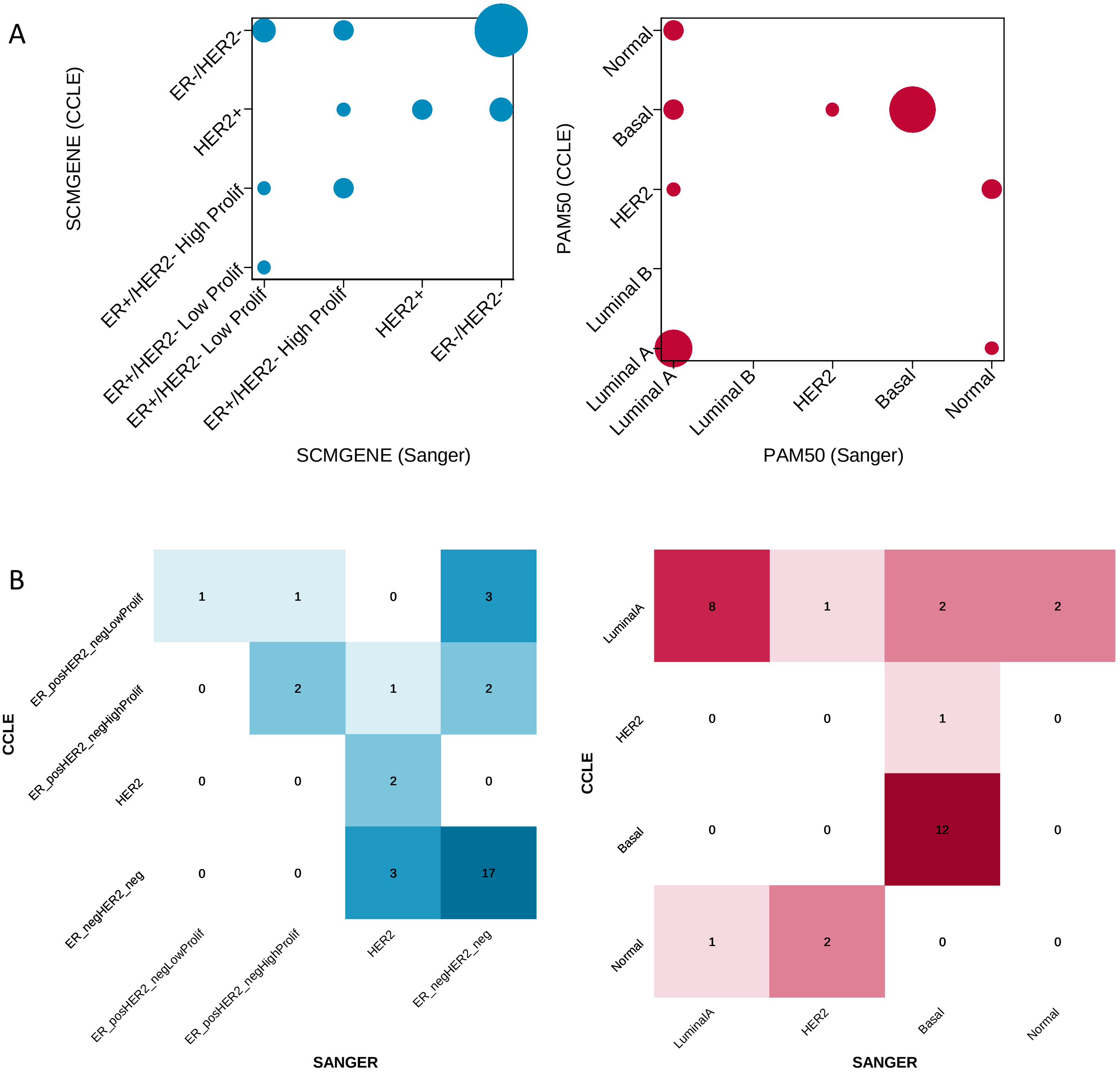

Supplement: Additional file 7: — PAM50 and SCMGENE subtyping of cell lines. Weighted scatter plots and cross tabulations of PAM50 and SCMGENE subtypes of cell lines according to dataset (Sanger COSMIC and CCLE). [file 13059_2014_431_MOESM7_ESM.pdf]
