# Supplementary material for: Genome-driven integrated classification of breast cancer validated in over 7,500 samples
Source: Genome Biol. 2014 Aug 28;15(8):431. doi: 10.1186/s13059-014-0431-1 (PMC4166472; doi:10.1186/s13059-014-0431-1)
Supplement: Additional file 12: — Lists of genes included in genomic loci reported as recurrently amplified or deleted in breast cancer by the TCGA. [file 13059_2014_431_MOESM12_ESM.pdf]

Gene	Type
ERBB2	Amplification
PGAP3	Amplification
CCND1	Amplification
ORAOV1	Amplification
ZNF703	Amplification
CLNS1A	Amplification
NDUFC2	Amplification
PAK1	Amplification
THRSP	Amplification
GAB2	Amplification
C11orf67	Amplification
RSF1	Amplification
USP35	Amplification
KCTD14	Amplification
ALG8	Amplification
INTS4	Amplification
GDPD4	Amplification
AQP11	Amplification
KCTD21	Amplification
TUBD1	Amplification
RPS6KB1	Amplification
MYC	Amplification
ZNF217	Amplification
IKBKB	Amplification
POLB	Amplification
VDAC3	Amplification
DKK4	Amplification
hsa-mir-587	Amplification
?|553137	Amplification
hsa-mir-4257	Amplification
CTSS	Amplification
ENSA	Amplification
MCL1	Amplification
ADAMTSL4	Amplification
GOLPH3L	Amplification
HORMAD1	Amplification
IGF1R	Amplification
PGPEP1L	Amplification
FAM169B	Amplification
hsa-mir-1279	Amplification
CPM	Amplification
LYZ	Amplification
YEATS4	Amplification
FRS2	Amplification
CPSF6	Amplification

MDM2	Amplification
TPD52	Amplification
HEY1	Amplification
MRPS28	Amplification
PAG1	Amplification
ZBTB10	Amplification
ZNF704	Amplification
DDAH1	Amplification
PIK3CA	Amplification
KCNMB2	Amplification
KCNMB3	Amplification
ZNF639	Amplification
MFN1	Amplification
GNB4	Amplification
ZMAT3	Amplification
TBL1XR1	Amplification
CCNE1	Amplification
ANXA11	Amplification
SFTPD	Amplification
MBL1P	Amplification
PPIF	Amplification
ZMIZ1	Amplification
C10orf57	Amplification
EIF5AL1	Amplification
LOC219347	Amplification
PLAC9	Amplification
ZCCHC24	Amplification
LOC283050	Amplification
LOC650623	Amplification
SFTPA1	Amplification
SFTPA2	Amplification
hsa-mir-3124	Amplification
hsa-mir-3123	Amplification
hsa-mir-1537	Amplification
hsa-mir-1182	Amplification
ACTN2	Amplification
ADSS	Amplification
AGT	Amplification
CHML	Amplification
LYST	Amplification
CHRM3	Amplification
FH	Amplification
GALNT2	Amplification
GNG4	Amplification
HNRNPU	Amplification
KCNK1	Amplification

LGALS8	Amplification
MTR	Amplification
NID1	Amplification
RGS7	Amplification
RYR2	Amplification
TARBP1	Amplification
TBCE	Amplification
GPR137B	Amplification
TSNAX	Amplification
ZNF124	Amplification
GNPAT	Amplification
KMO	Amplification
EXO1	Amplification
GGPS1	Amplification
TOMM20	Amplification
CEP170	Amplification
AKT3	Amplification
ZNF238	Amplification
CAPN9	Amplification
SDCCAG8	Amplification
COG2	Amplification
RBM34	Amplification
OPN3	Amplification
TRIM58	Amplification
AHCTF1	Amplification
OR1C1	Amplification
OR2M4	Amplification
OR2L2	Amplification
OR2L1P	Amplification
OR2T1	Amplification
DISC2	Amplification
DISC1	Amplification
PPPDE1	Amplification
SCCPDH	Amplification
ARID4B	Amplification
EGLN1	Amplification
KIAA1383	Amplification
KIF26B	Amplification
HEATR1	Amplification
ZNF692	Amplification
ERO1LB	Amplification
FMN2	Amplification
ZNF695	Amplification
SIPA1L2	Amplification
ZP4	Amplification
TFB2M	Amplification

GREM2	Amplification
SMYD3	Amplification
ARV1	Amplification
TTC13	Amplification
PGBD5	Amplification
ZNF669	Amplification
ZNF672	Amplification
PCNXL2	Amplification
SH3BP5L	Amplification
OR2G3	Amplification
OR2G2	Amplification
OR2C3	Amplification
C1orf124	Amplification
C1orf57	Amplification
EFCAB2	Amplification
KIAA1804	Amplification
ZNF496	Amplification
C1orf198	Amplification
ZNF670	Amplification
NLRP3	Amplification
FAM36A	Amplification
OR2M5	Amplification
OR2M3	Amplification
OR2T12	Amplification
OR14C36	Amplification
OR2T34	Amplification
OR2T10	Amplification
OR2T4	Amplification
OR2T11	Amplification
OR2B11	Amplification
WDR64	Amplification
C1orf131	Amplification
EDARADD	Amplification
SLC35F3	Amplification
B3GALNT2	Amplification
C1orf150	Amplification
LOC148824	Amplification
LOC149134	Amplification
EXOC8	Amplification
CNST	Amplification
PLD5	Amplification
C1orf100	Amplification
OR2T6	Amplification
C1orf101	Amplification
PGBD2	Amplification
OR2L13	Amplification

OR14A16	Amplification
NCRNA0020[illegible]	Amplification
VN1R5	Amplification
LOC339535	Amplification
OR6F1	Amplification
OR2W3	Amplification
OR2T8	Amplification
OR2T3	Amplification
OR2T29	Amplification
IRF2BP2	Amplification
FAM89A	Amplification
C1orf31	Amplification
C1orf229	Amplification
OR2M1P	Amplification
OR11L1	Amplification
OR2L8	Amplification
OR2AK2	Amplification
OR2L3	Amplification
OR2M2	Amplification
OR2T33	Amplification
OR2M7	Amplification
OR2G6	Amplification
OR2T2	Amplification
OR2T5	Amplification
OR14I1	Amplification
OR2T27	Amplification
OR2T35	Amplification
TRIM67	Amplification
MAP1LC3C	Amplification
OR2W5	Amplification
OR13G1	Amplification
LOC646627	Amplification
SNORA14B	Amplification
LOC1001303	Amplification
TSNAX-DISC1	Amplification
IL11	Amplification
PTPRH	Amplification
RPL28	Amplification
SYT5	Amplification
TNNI3	Amplification
TNNT1	Amplification
U2AF2	Amplification
SAPS1	Amplification
HSPBP1	Amplification
UBE2S	Amplification
CCDC106	Amplification

EPN1	Amplification
ZNF580	Amplification
ZNF581	Amplification
PPP1R12C	Amplification
EPS8L1	Amplification
NAT14	Amplification
ISOC2	Amplification
BRSK1	Amplification
SUV420H2	Amplification
FIZ1	Amplification
ZNF628	Amplification
RDH13	Amplification
COX6B2	Amplification
TMEM190	Amplification
ZNF524	Amplification
ZNF784	Amplification
ZNF579	Amplification
NLRP11	Amplification
TMEM86B	Amplification
SSC5D	Amplification
TMEM150B	Amplification
FAM71E2	Amplification
NLRP9	Amplification
RFPL4A	Amplification
C19orf51	Amplification
SBK2	Amplification
SHISA7	Amplification
NINJ2	Amplification
RAD52	Amplification
KDM5A	Amplification
SLC6A12	Amplification
SLC6A13	Amplification
ERC1	Amplification
WNK1	Amplification
CCDC77	Amplification
B4GALNT3	Amplification
HSN2	Amplification
IQSEC3	Amplification
FAM138D	Amplification
LOC1002887	Amplification
AFM	Amplification
AFP	Amplification
ALB	Amplification
ANKRD17	Amplification
COX18	Amplification
ITPR1	Amplification

BHLHE40	Amplification
SUMF1	Amplification
EGOT	Amplification
FOXA1	Amplification
MIPOL1	Amplification
CD59	Amplification
CSTF3	Amplification
WT1	Amplification
HIPK3	Amplification
EIF3M	Amplification
C11orf41	Amplification
FBXO3	Amplification
WIT1	Amplification
TCP11L1	Amplification
PRRG4	Amplification
QSER1	Amplification
DEPDC7	Amplification
LOC283267	Amplification
CCDC73	Amplification
EGFR	Amplification
LANCL2	Amplification
VOPP1	Amplification
41166	Amplification
FKBP9L	Amplification
LOC442308	Amplification
PHKB	Amplification
ALOX5AP	Amplification
HMGB1	Amplification
UBL3	Amplification
USPL1	Amplification
KATNAL1	Amplification
LOC1001889	Amplification
USP22	Amplification
EDN1	Amplification
GCNT2	Amplification
GMPR	Amplification
HIVEP1	Amplification
JARID2	Amplification
MAK	Amplification
NEDD9	Amplification
ATXN1	Amplification
TFAP2A	Amplification
TPMT	Amplification
DEK	Amplification
GCM2	Amplification
CD83	Amplification

NUP153	Amplification
RANBP9	Amplification
CAP2	Amplification
SIRT5	Amplification
MYLIP	Amplification
TBC1D7	Amplification
NOL7	Amplification
FAM8A1	Amplification
TMEM14C	Amplification
GFOD1	Amplification
ELOVL2	Amplification
PAK1IP1	Amplification
CCDC90A	Amplification
KIF13A	Amplification
TMEM14B	Amplification
DTNBP1	Amplification
C6orf105	Amplification
C6orf114	Amplification
KDM1B	Amplification
RBM24	Amplification
RNF182	Amplification
PHACTR1	Amplification
LOC221710	Amplification
SYCP2L	Amplification
C6orf218	Amplification
C6orf52	Amplification
NHLRC1	Amplification
ERVFRDE1	Amplification
TMEM170B	Amplification
hsa-mir-3155	Amplification
hsa-mir-548c	Amplification
ATP5C1	Amplification
CALML3	Amplification
AKR1C4	Amplification
KLF6	Amplification
AKR1C1	Amplification
AKR1C2	Amplification
GATA3	Amplification
GDI2	Amplification
IL2RA	Amplification
IL15RA	Amplification
ITIH2	Amplification
PFKFB3	Amplification
PFKP	Amplification
PHYH	Amplification
PRKCQ	Amplification

PRPF18	Amplification
AKR1C3	Amplification
CDC123	Amplification
USP6NL	Amplification
OPTN	Amplification
NET1	Amplification
PITRM1	Amplification
CELF2	Amplification
NUDT5	Amplification
SEPHS1	Amplification
KIN	Amplification
UPF2	Amplification
CALML5	Amplification
ANKRD16	Amplification
C10orf18	Amplification
SEC61A2	Amplification
MCM10	Amplification
DHTKD1	Amplification
FRMD4A	Amplification
CAMK1D	Amplification
SFMBT2	Amplification
ECHDC3	Amplification
ASB13	Amplification
TUBAL3	Amplification
ITIH5	Amplification
AKR1E2	Amplification
CCDC3	Amplification
TAF3	Amplification
FBXO18	Amplification
RBM17	Amplification
UCN3	Amplification
SFTA1P	Amplification
UCMA	Amplification
BEND7	Amplification
LOC254312	Amplification
C10orf47	Amplification
LOC283070	Amplification
LOC338588	Amplification
AKR1CL1	Amplification
tAKR	Amplification
FLJ45983	Amplification
LOC1002160	Amplification
hsa-mir-125	Deletion
hsa-mir-129	Deletion
ALPL	Deletion
C1QA	Deletion

C1QB	Deletion
C1QC	Deletion
CAPZB	Deletion
CASP9	Deletion
TNFRSF8	Deletion
CDA	Deletion
CDC42	Deletion
CLCN6	Deletion
CLCNKA	Deletion
CLCNKB	Deletion
CORT	Deletion
DDOST	Deletion
DFFA	Deletion
ECE1	Deletion
EPHA2	Deletion
EPHA8	Deletion
MTOR	Deletion
HSPG2	Deletion
HTR6	Deletion
MFAP2	Deletion
MTHFR	Deletion
NBL1	Deletion
NPPA	Deletion
NPPB	Deletion
PAX7	Deletion
PEX14	Deletion
PGD	Deletion
PLA2G2A	Deletion
PLA2G5	Deletion
PLOD1	Deletion
EXOSC10	Deletion
RAP1GAP	Deletion
RSC1A1	Deletion
SDHB	Deletion
SRM	Deletion
TNFRSF1B	Deletion
ZBTB17	Deletion
PRDM2	Deletion
AKR7A2	Deletion
ALDH4A1	Deletion
EIF4G3	Deletion
DHRS3	Deletion
CROCC	Deletion
ZBTB40	Deletion
MFN2	Deletion
CELA3A	Deletion

ANGPTL7	Deletion
MAD2L2	Deletion
PDPN	Deletion
MASP2	Deletion
MST1P2	Deletion
MST1P9	Deletion
PADI2	Deletion
CTRC	Deletion
AKR7A3	Deletion
SPEN	Deletion
KIAA0090	Deletion
PLEKHM2	Deletion
OTUD3	Deletion
KAZ	Deletion
DNAJC16	Deletion
UBR4	Deletion
ATP13A2	Deletion
TARDBP	Deletion
CELA3B	Deletion
PADI4	Deletion
C1orf144	Deletion
FBXO2	Deletion
FBXO6	Deletion
PLA2G2D	Deletion
HSPB7	Deletion
HSPC157	Deletion
UBIAD1	Deletion
PADI1	Deletion
PLA2G2E	Deletion
HP1BP3	Deletion
CELA2B	Deletion
MRTO4	Deletion
PADI3	Deletion
WNT4	Deletion
FBXO42	Deletion
RNF186	Deletion
FBLIM1	Deletion
PQLC2	Deletion
CASZ1	Deletion
TMEM51	Deletion
ARHGEF10L	Deletion
VPS13D	Deletion
CAMK2N1	Deletion
NBPF1	Deletion
NECAP2	Deletion
RCC2	Deletion

AGTRAP	Deletion
PTCHD2	Deletion
KIF17	Deletion
MIIP	Deletion
CELA2A	Deletion
PLA2G2F	Deletion
PINK1	Deletion
PRAMEF1	Deletion
PRAMEF2	Deletion
EFHD2	Deletion
C1orf89	Deletion
MUL1	Deletion
AGMAT	Deletion
TAS1R2	Deletion
ACTL8	Deletion
USP48	Deletion
NBPF3	Deletion
DDI2	Deletion
CROCCL1	Deletion
IGSF21	Deletion
KIAA2013	Deletion
C1orf158	Deletion
FBXO44	Deletion
CROCCL2	Deletion
FHAD1	Deletion
AADACL3	Deletion
IFFO2	Deletion
KLHDC7A	Deletion
VWA5B1	Deletion
UBXN10	Deletion
ARHGEF19	Deletion
C1orf127	Deletion
C1orf64	Deletion
FAM43B	Deletion
C1orf126	Deletion
AKR7L	Deletion
TMCO4	Deletion
SLC25A34	Deletion
ESPNP	Deletion
AADACL4	Deletion
PRAMEF5|34	Deletion
HNRNPCL1	Deletion
PRAMEF9|34	Deletion
PRAMEF10	Deletion
FAM131C	Deletion
PADI6	Deletion

C1orf187	Deletion
SPATA21	Deletion
APITD1	Deletion
TMEM82	Deletion
PRAMEF12	Deletion
PRAMEF8|39	Deletion
PRAMEF18|3	Deletion
PRAMEF17	Deletion
PLA2G2C	Deletion
PRAMEF4	Deletion
PRAMEF13	Deletion
SH2D5	Deletion
PRAMEF3	Deletion
LDLRAD2	Deletion
PRAMEF11	Deletion
PRAMEF6	Deletion
LOC440563	Deletion
UQCRHL	Deletion
C1orf151	Deletion
PRAMEF20|6	Deletion
LOC649330	Deletion
PRAMEF22	Deletion
PRAMEF16	Deletion
SNORA59B|6	Deletion
PRAMEF14|7	Deletion
FLJ37453	Deletion
CSMD1	Deletion
ATP5D	Deletion
CIRBP	Deletion
EFNA2	Deletion
GPX4	Deletion
STK11	Deletion
SBNO2	Deletion
C19orf24	Deletion
MUM1	Deletion
MIDN	Deletion
C19orf23	Deletion
C19orf26	Deletion
RB1	Deletion
LPAR6	Deletion
CDKN2A	Deletion
CDKN2B	Deletion
MTAP	Deletion
C9orf53	Deletion
CDKN2BAS	Deletion
PTEN	Deletion

KILLIN	Deletion
hsa-mir-4269	Deletion
hsa-mir-3133	Deletion
hsa-mir-149	Deletion
AGXT	Deletion
KIF1A	Deletion
BOK	Deletion
COL6A3	Deletion
DTYMK	Deletion
GBX2	Deletion
GPC1	Deletion
GPR35	Deletion
HDLBP	Deletion
NDUFA10	Deletion
41154	Deletion
PDCD1	Deletion
PPP1R7	Deletion
SPP2	Deletion
PER2	Deletion
LRRFIP1	Deletion
HDAC4	Deletion
FARP2	Deletion
ARL4C	Deletion
RAMP1	Deletion
STK25	Deletion
COPS8	Deletion
CAPN10	Deletion
PASK	Deletion
ATG4B	Deletion
SH3BP4	Deletion
SNED1	Deletion
TRAF3IP1	Deletion
ANO7	Deletion
PRLH	Deletion
THAP4	Deletion
ANKMY1	Deletion
SCLY	Deletion
ASB1	Deletion
UGT1A10	Deletion
UGT1A8	Deletion
UGT1A7	Deletion
UGT1A6	Deletion
UGT1A5	Deletion
UGT1A9	Deletion
UGT1A4	Deletion
UGT1A1	Deletion

UGT1A3	Deletion
HJURP	Deletion
HES6	Deletion
CXCR7	Deletion
RNPEPL1	Deletion
GAL3ST2	Deletion
RAB17	Deletion
TRPM8	Deletion
MLPH	Deletion
IQCA1	Deletion
C2orf54	Deletion
ILKAP	Deletion
ING5	Deletion
MGC16025	Deletion
AGAP1	Deletion
TWIST2	Deletion
NEU4	Deletion
MTERFD2	Deletion
UBE2F	Deletion
OTOS	Deletion
MYEOV2	Deletion
OR6B3	Deletion
LOC151174	Deletion
MSL3L2	Deletion
C2orf85	Deletion
DUSP28	Deletion
ESPNL	Deletion
RBM44	Deletion
AQP12A	Deletion
KLHL30	Deletion
OR6B2	Deletion
ASB18	Deletion
DNAJB3	Deletion
LOC643387	Deletion
PRR21	Deletion
AQP12B	Deletion
D2HGDH	Deletion
LOC728323	Deletion
PP14571	Deletion
hsa-mir-1305	Deletion
SLC25A4	Deletion
CASP3	Deletion
DCTD	Deletion
F11	Deletion
ACSL1	Deletion
FAT1	Deletion

FRG1	Deletion
ING2	Deletion
IRF2	Deletion
KLKB1	Deletion
MTNR1A	Deletion
TLR3	Deletion
SORBS2	Deletion
DUX4|22947	Deletion
FAM149A	Deletion
PDLIM3	Deletion
CLDN22	Deletion
UFSP2	Deletion
CDKN2AIP	Deletion
ODZ3	Deletion
LRP2BP	Deletion
TUBB4Q	Deletion
STOX2	Deletion
KIAA1430	Deletion
C4orf41	Deletion
MLF1IP	Deletion
WWC2	Deletion
SNX25	Deletion
MGC45800	Deletion
ZFP42	Deletion
ENPP6	Deletion
C4orf38	Deletion
RWDD4A	Deletion
CCDC111	Deletion
TRIML2	Deletion
CCDC110	Deletion
CYP4V2	Deletion
TRIML1	Deletion
ANKRD37	Deletion
HELT	Deletion
FAM92A3	Deletion
C4orf47	Deletion
FRG2	Deletion
SLED1	Deletion
LOC653544|(	Deletion
LOC653545	Deletion
hsa-mir-744	Deletion
MAP2K4	Deletion
GPR31	Deletion
KIF25	Deletion
MLLT4	Deletion
TCP10	Deletion

C6orf123	Deletion
UNC93A	Deletion
SMOC2	Deletion
FRMD1	Deletion
TTLL2	Deletion
DACT2	Deletion
TCP10L2	Deletion
C6orf124	Deletion
HGC6.3	Deletion
OPCML	Deletion
IGSF9B	Deletion
NCAPD3	Deletion
ACAD8	Deletion
B3GAT1	Deletion
THYN1	Deletion
JAM3	Deletion
GLB1L2	Deletion
VPS26B	Deletion
GLB1L3	Deletion
SPATA19	Deletion
LOC283174	Deletion
LOC1001282	Deletion
hsa-mir-4271	Deletion
hsa-mir-711	Deletion
hsa-mir-2115	Deletion
hsa-mir-566	Deletion
hsa-mir-425	Deletion
hsa-mir-191	Deletion
hsa-mir-135a	Deletion
hsa-let-7g	Deletion
ACY1	Deletion
ALAS1	Deletion
AMT	Deletion
APEH	Deletion
RHOA	Deletion
SLC25A20	Deletion
CAMP	Deletion
CDC25A	Deletion
CISH	Deletion
COL7A1	Deletion
DAG1	Deletion
DOCK3	Deletion
DUSP7	Deletion
CELSR3	Deletion
GNAI2	Deletion
GNAT1	Deletion

GPX1	Deletion
GRM2	Deletion
HYAL1	Deletion
IMPDH2	Deletion
ITIH1	Deletion
ITIH3	Deletion
ITIH4	Deletion
LAMB2	Deletion
MST1	Deletion
MST1R	Deletion
PFKFB4	Deletion
PLXNB1	Deletion
PRKAR2A	Deletion
PRKCD	Deletion
QARS	Deletion
RPL29	Deletion
SEMA3F	Deletion
NEK4	Deletion
TCTA	Deletion
TKT	Deletion
TNNC1	Deletion
UBA7	Deletion
USP4	Deletion
UQCRC1	Deletion
IFRD2	Deletion
MAPKAPK3	Deletion
SEMA3B	Deletion
MANF	Deletion
BAP1	Deletion
HYAL3	Deletion
HYAL2	Deletion
BSN	Deletion
RRP9	Deletion
CACNA2D2	Deletion
VPRBP	Deletion
IP6K1	Deletion
PARP3	Deletion
RBM6	Deletion
RBM5	Deletion
NME6	Deletion
TRAIP	Deletion
ARIH2	Deletion
NPRL2	Deletion
USP19	Deletion
SLC38A3	Deletion
CYB561D2	Deletion

TMEM115	Deletion
WDR6	Deletion
RASSF1	Deletion
NISCH	Deletion
TREX1	Deletion
TUSC2	Deletion
TWF2	Deletion
LAMB2L	Deletion
RAD54L2	Deletion
STAB1	Deletion
NAT6	Deletion
ABHD14A	Deletion
POC1A	Deletion
NDUFAF3	Deletion
DNAH1	Deletion
GNL3	Deletion
SPCS1	Deletion
RBM15B	Deletion
GMPPB	Deletion
C3orf18	Deletion
SHISA5	Deletion
ZMYND10	Deletion
TEX264	Deletion
CCDC72	Deletion
ZNF589	Deletion
HEMK1	Deletion
IP6K2	Deletion
SFMBT1	Deletion
NCKIPSD	Deletion
PHF7	Deletion
TLR9	Deletion
P4HTM	Deletion
QRICH1	Deletion
DALRD3	Deletion
PBRM1	Deletion
DCP1A	Deletion
GLT8D1	Deletion
SEMA3G	Deletion
PCBP4	Deletion
RNF123	Deletion
CCDC71	Deletion
NT5DC2	Deletion
SLC26A6	Deletion
CAMKV	Deletion
CCDC51	Deletion
WDR82	Deletion

ATRIP	Deletion
NICN1	Deletion
MON1A	Deletion
ABHD14B	Deletion
UCN2	Deletion
RFT1	Deletion
GPR62	Deletion
IQCF1	Deletion
GLYCTK	Deletion
PPM1M	Deletion
C3orf45	Deletion
KLHDC8B	Deletion
FBXW12	Deletion
CCDC36	Deletion
C3orf62	Deletion
TMEM110	Deletion
AMIGO3	Deletion
CDHR4	Deletion
C3orf54	Deletion
IQCF2	Deletion
IQCF5	Deletion
MUSTN1	Deletion
IQCF3	Deletion
TMEM89	Deletion
IQCF6	Deletion
LOC440957	Deletion
SPINK8	Deletion
C3orf71	Deletion
LOC646498	Deletion
SNORD19	Deletion
SNORD69	Deletion
SNORD19B	Deletion
C3orf74	Deletion
hsa-mir-311[illegible]	Deletion
hsa-mir-315[illegible]	Deletion
BAGE	Deletion
TPTE	Deletion
C21orf15	Deletion
BAGE2	Deletion
C21orf99	Deletion
LIPI	Deletion
POTED	Deletion
C21orf81	Deletion
LOC1001322[illegible]	Deletion
MLL3	Deletion
hsa-mir-311[illegible]	Deletion

hsa-mir-3118	Deletion
hsa-mir-1268	Deletion
hsa-mir-211	Deletion
APBA2	Deletion
BCL8	Deletion
GABRA5	Deletion
GABRB3	Deletion
GABRG3	Deletion
IPW	Deletion
TRPM1	Deletion
NDN	Deletion
OCA2	Deletion
SNRPN	Deletion
TJP1	Deletion
UBE3A	Deletion
MKRN3	Deletion
PAR5	Deletion
HERC2	Deletion
SNURF	Deletion
MTMR15	Deletion
CYFIP1	Deletion
FAM189A1	Deletion
C15orf2	Deletion
DKFZP434L18	Deletion
KLF13	Deletion
MAGEL2	Deletion
MTMR10	Deletion
NDNL2	Deletion
ATP10A	Deletion
NIPA2	Deletion
CHRFAM7A	Deletion
FAM7A3|898	Deletion
ARHGAP11B	Deletion
TUBGCP5	Deletion
NIPA1	Deletion
PAR1	Deletion
OR4N4	Deletion
GOLGA6L1	Deletion
GOLGA8G|28	Deletion
GOLGA9P	Deletion
SNORD109B|	Deletion
SNORD115-1	Deletion
WHAMML1	Deletion
POTEB|3390	Deletion
PAR4	Deletion
PAR-SN	Deletion

GOLGA8E	Deletion
OR4M2	Deletion
OR4N3P	Deletion
HERC2P2	Deletion
NF1P1|4402:	Deletion
WHAMML2	Deletion
LOC646214	Deletion
CXADRP2	Deletion
SNORD116-1	Deletion
GOLGA6L6	Deletion
LOC727924	Deletion
GOLGA8C	Deletion
PWRN1	Deletion
PWRN2	Deletion
SNORD116-1	Deletion
SNORD116-2	Deletion
SNORD116-3	Deletion
SNORD116-4	Deletion
SNORD116-5	Deletion
SNORD116-8	Deletion
SNORD116-1	Deletion
SNORD116-1	Deletion
SNORD116-1	Deletion
SNORD116-1	Deletion
SNORD116-1	Deletion
SNORD116-1	Deletion
SNORD116-1	Deletion
SNORD116-1	Deletion
SNORD116-2	Deletion
SNORD116-2	Deletion
SNORD116-2	Deletion
SNORD116-2	Deletion
SNORD116-2	Deletion
SNORD115-2	Deletion
SNORD116-2	Deletion
SNORD116-2	Deletion
SNORD115-3	Deletion
SNORD115-4	Deletion
SNORD115-5	Deletion
SNORD115-6	Deletion
SNORD115-7	Deletion
SNORD115-8	Deletion
SNORD115-9	Deletion
SNORD115-1	Deletion
SNORD115-1	Deletion
SNORD115-1	Deletion

SNORD115-1	Deletion
SNORD115-1	Deletion
SNORD115-1	Deletion
SNORD115-2	Deletion
SNORD115-2	Deletion
SNORD115-2	Deletion
SNORD115-2	Deletion
SNORD115-3	Deletion
SNORD115-3	Deletion
SNORD115-3	Deletion
SNORD115-3	Deletion
SNORD115-3	Deletion
SNORD115-3	Deletion
SNORD115-3	Deletion
SNORD115-3	Deletion
SNORD115-4	Deletion
SNORD115-4	Deletion
SNORD115-4	Deletion
SNORD116-2	Deletion
SNORD116-2	Deletion
SNORD115-4	Deletion
HBII-52-24	Deletion
HBII-52-27	Deletion
HBII-52-28	Deletion
HBII-52-45	Deletion
HBII-52-46	Deletion
GOLGA8F|1C	Deletion
GOLGA8DP	Deletion
FAM7A2	Deletion
IL6ST	Deletion
MAP3K1	Deletion
DDX4	Deletion
GPBP1	Deletion
ANKRD55	Deletion
C5orf35	Deletion
IL31RA	Deletion
MIER3	Deletion
ACTBL2	Deletion
hsa-mir-4298	Deletion
hsa-mir-675	Deletion
hsa-mir-483	Deletion
hsa-mir-210	Deletion
AP2A2	Deletion
ASCL2	Deletion
CARS	Deletion
CD81	Deletion

CD151	Deletion
CDKN1C	Deletion
CTSD	Deletion
DRD4	Deletion
DUSP8	Deletion
HRAS	Deletion
IGF2	Deletion
INS	Deletion
IRF7	Deletion
KCNQ1	Deletion
LSP1	Deletion
MUC2	Deletion
MUC6	Deletion
NAP1L4	Deletion
SLC22A18	Deletion
SLC22A18AS	Deletion
POLR2L	Deletion
PSMD13	Deletion
RNH1	Deletion
MRPL23	Deletion
RPLP2	Deletion
SCT	Deletion
TALDO1	Deletion
TH	Deletion
TSPAN4	Deletion
TNNI2	Deletion
TNNT3	Deletion
PHLDA2	Deletion
RASSF7	Deletion
IFITM1	Deletion
BRSK2	Deletion
TSPAN32	Deletion
TSSC4	Deletion
IFITM3	Deletion
DEAF1	Deletion
IFITM2	Deletion
KCNQ1OT1	Deletion
PKP3	Deletion
SIRT3	Deletion
C11orf21	Deletion
TRPM5	Deletion
IGF2AS	Deletion
BET1L	Deletion
CEND1	Deletion
CDHR5	Deletion
TOLLIP	Deletion

LRDD	Deletion
KCNQ1DN	Deletion
PNPLA2	Deletion
PHRF1	Deletion
SIGIRR	Deletion
RIC8A	Deletion
EPS8L2	Deletion
CHID1	Deletion
SLC25A22	Deletion
ATHL1	Deletion
PTDSS2	Deletion
MOB2	Deletion
SYT8	Deletion
ODF3	Deletion
OSBPL5	Deletion
LRRC56	Deletion
MRGPRE	Deletion
LOC143666	Deletion
SCGB1C1	Deletion
NLRP6	Deletion
C11orf35	Deletion
H19	Deletion
EFCAB4A	Deletion
TMEM80	Deletion
C11orf36	Deletion
ANO9	Deletion
LOC338651	Deletion
B4GALNT4	Deletion
PDDC1	Deletion
MRGPRG	Deletion
KRTAP5-1	Deletion
KRTAP5-3	Deletion
KRTAP5-4	Deletion
IFITM5	Deletion
FAM99A	Deletion
KRTAP5-5	Deletion
KRTAP5-2	Deletion
KRTAP5-6	Deletion
SNORA52	Deletion
SNORA54	Deletion
INS-IGF2	Deletion
MUC5B	Deletion
FAM99B	Deletion
LOC1001331	Deletion
LOC1001335	Deletion
hsa-mir-1260	Deletion

hsa-mir-625	Deletion
ACTN1	Deletion
ACYP1	Deletion
ARG2	Deletion
ZFP36L1	Deletion
ENTPD5	Deletion
DIO2	Deletion
DLST	Deletion
EIF2S1	Deletion
ERH	Deletion
ESRRB	Deletion
FNTB	Deletion
FOS	Deletion
FUT8	Deletion
GPX2	Deletion
GSTZ1	Deletion
GTF2A1	Deletion
LTBP2	Deletion
MAX	Deletion
MAP3K9	Deletion
ALDH6A1	Deletion
PGF	Deletion
PIGH	Deletion
PSEN1	Deletion
ABCD4	Deletion
RAD51L1	Deletion
SEL1L	Deletion
SFRS5	Deletion
SLC8A3	Deletion
SLC10A1	Deletion
TGFB3	Deletion
TSHR	Deletion
DPF3	Deletion
NUMB	Deletion
ADAM21	Deletion
ADAM20	Deletion
DCAF5	Deletion
ALKBH1	Deletion
EIF2B2	Deletion
PNMA1	Deletion
NRXN3	Deletion
SPTLC2	Deletion
RGS6	Deletion
KIAA0247	Deletion
KIAA0317	Deletion
MED6	Deletion

GPHN	Deletion
VTI1B	Deletion
BATF	Deletion
NPC2	Deletion
AHSA1	Deletion
ACOT2	Deletion
TMED10	Deletion
C14orf1	Deletion
VASH1	Deletion
SNW1	Deletion
PCNX	Deletion
TTLL5	Deletion
ANGEL1	Deletion
ZFYVE26	Deletion
TTC9	Deletion
FLRT2	Deletion
SIPA1L1	Deletion
DCAF4	Deletion
PLEK2	Deletion
MLH3	Deletion
POMT2	Deletion
COQ6	Deletion
FCF1	Deletion
RDH11	Deletion
COX16	Deletion
ATP6V1D	Deletion
ZFYVE1	Deletion
EXD2	Deletion
C14orf115	Deletion
SYNJ2BP	Deletion
SLC39A9	Deletion
FLVCR2	Deletion
C14orf118	Deletion
YLPM1	Deletion
C14orf162	Deletion
ADCK1	Deletion
TMEM63C	Deletion
GALNTL1	Deletion
PLEKHH1	Deletion
ZNF410	Deletion
NGB	Deletion
RBM25	Deletion
VIPAR	Deletion
SMOC1	Deletion
C14orf4	Deletion
MPP5	Deletion

FAM164C	Deletion
C14orf169	Deletion
C14orf45	Deletion
C14orf156	Deletion
DNAL1	Deletion
RPS6KL1	Deletion
STON2	Deletion
KIAA1737	Deletion
PAPLN	Deletion
CHURC1	Deletion
C14orf43	Deletion
LIN52	Deletion
NEK9	Deletion
C14orf179	Deletion
C14orf148	Deletion
JDP2	Deletion
ISCA2	Deletion
ACOT4	Deletion
RDH12	Deletion
ADAM21P1	Deletion
LOC145474	Deletion
PTGR2	Deletion
FAM161B	Deletion
C14orf166B	Deletion
ISM2	Deletion
C14orf145	Deletion
FAM71D	Deletion
TMEM229B	Deletion
C14orf174	Deletion
PROX2	Deletion
ZDHHC22	Deletion
TMED8	Deletion
C14orf178	Deletion
SNORD56B	Deletion
VSX2	Deletion
RAB15	Deletion
HEATR4	Deletion
C14orf181	Deletion
UPF0639	Deletion
C14orf53	Deletion
ACOT1	Deletion
ACOT6	Deletion
LOC645431	Deletion
TMEM90A	Deletion
SNORA79	Deletion
hsa-mir-431[illegible]	Deletion

hsa-mir-2117	Deletion
ACLY	Deletion
AOC2	Deletion
ARL4D	Deletion
ATP6V0A1	Deletion
BRCA1	Deletion
FMNL1	Deletion
CNP	Deletion
CRHR1	Deletion
DHX8	Deletion
DUSP3	Deletion
ETV4	Deletion
EZH1	Deletion
FZD2	Deletion
G6PC	Deletion
KAT2A	Deletion
GFAP	Deletion
CCR10	Deletion
GRN	Deletion
HCRT	Deletion
HSD17B1	Deletion
IFI35	Deletion
ITGA2B	Deletion
NBR1	Deletion
MAPT	Deletion
ADAM11	Deletion
MEOX1	Deletion
MPP2	Deletion
MPP3	Deletion
NAGLU	Deletion
NMT1	Deletion
NSF	Deletion
PPY	Deletion
PYY	Deletion
RAB5C	Deletion
RPL27	Deletion
SLC4A1	Deletion
STAT3	Deletion
STAT5A	Deletion
STAT5B	Deletion
MLX	Deletion
DNAJC7	Deletion
TUBG1	Deletion
UBTF	Deletion
RND2	Deletion
CNTNAP1	Deletion

AOC3	Deletion
BECN1	Deletion
MAP3K14	Deletion
EFTUD2	Deletion
PLEKHM1	Deletion
LRRC37A	Deletion
HDAC5	Deletion
GJC1	Deletion
PSME3	Deletion
NBR2	Deletion
RAMP2	Deletion
VAT1	Deletion
SC65	Deletion
HEXIM1	Deletion
C1QL1	Deletion
RUNDC3A	Deletion
GPATCH8	Deletion
KCNH4	Deletion
C17orf88	Deletion
TUBG2	Deletion
NKIRAS2	Deletion
CCDC56	Deletion
PSMC3IP	Deletion
SOST	Deletion
ARL17A|513	Deletion
SLC25A39	Deletion
HIGD1B	Deletion
LRRC37A4	Deletion
KLHL11	Deletion
ATXN7L3	Deletion
FKBP10	Deletion
WNK4	Deletion
C17orf53	Deletion
TMUB2	Deletion
DHX58	Deletion
ACBD4	Deletion
DCAKD	Deletion
PLEKHH3	Deletion
DBF4B	Deletion
COASY	Deletion
AARSD1	Deletion
TTC25	Deletion
VPS25	Deletion
TMEM101	Deletion
GHDC	Deletion
LOC90586	Deletion

G6PC3	Deletion
ASB16	Deletion
HSPB9	Deletion
PLCD3	Deletion
TMEM106A	Deletion
NT5C3L	Deletion
C17orf46	Deletion
HEXIM2	Deletion
LSM12	Deletion
CCDC43	Deletion
CNTD1	Deletion
CD300LG	Deletion
KIF18B	Deletion
RUNDC1	Deletion
C17orf69	Deletion
NAGS	Deletion
FAM134C	Deletion
IMP5	Deletion
SH3D20	Deletion
ARHGAP27	Deletion
STH	Deletion
KIAA1267	Deletion
C17orf105	Deletion
FAM171A2	Deletion
C17orf104	Deletion
PTRF	Deletion
KLHL10	Deletion
C17orf65	Deletion
LOC388387	Deletion
CCDC103	Deletion
MGC57346	Deletion
LRRC37A2	Deletion
ARL17B	Deletion
LOC644172	Deletion
LOC1001289	Deletion
LOC1001301	Deletion
LOC1001305	Deletion
LOC1001339	Deletion
LOC1001909	Deletion
BPHL	Deletion
SERPINB1	Deletion
F13A1	Deletion
FOXF2	Deletion
FOXC1	Deletion
GMDS	Deletion
IRF4	Deletion

NQO2	Deletion
SERPINB6	Deletion
SERPINB9	Deletion
RREB1	Deletion
SSR1	Deletion
TUBB2A	Deletion
RIPK1	Deletion
PRPF4B	Deletion
CDYL	Deletion
LY86	Deletion
PECI	Deletion
FARS2	Deletion
RPP40	Deletion
FAM50B	Deletion
NRN1	Deletion
EXOC2	Deletion
WRNIP1	Deletion
DUSP22	Deletion
LYRM4	Deletion
SLC22A23	Deletion
FOXQ1	Deletion
HUS1B	Deletion
C6orf195	Deletion
C6orf145	Deletion
C6orf146	Deletion
LOC285768	Deletion
LOC285780	Deletion
MYLK4	Deletion
TUBB2B	Deletion
FAM136B	Deletion
PSMG4	Deletion
DKFZP686I15	Deletion
C6orf201	Deletion
PPP1R3G	Deletion
hsa-mir-378c	Deletion
hsa-mir-4297	Deletion
hsa-mir-202	Deletion
ADAM8	Deletion
BNIP3	Deletion
CYP2E1	Deletion
ECHS1	Deletion
INPP5A	Deletion
MGMT	Deletion
MKI67	Deletion
PTPRE	Deletion
UTF1	Deletion

GLRX3	Deletion
DPYSL4	Deletion
TUBGCP2	Deletion
DUX4|22947	Deletion
VENTX	Deletion
CALY	Deletion
PPP2R2D	Deletion
LRRC27	Deletion
GPR123	Deletion
NKX6-2	Deletion
KNDC1	Deletion
MTG1	Deletion
SYCE1	Deletion
PRAP1	Deletion
ZNF511	Deletion
CLRN3	Deletion
C10orf91	Deletion
PWWP2B	Deletion
PAOX	Deletion
EBF3	Deletion
C10orf93	Deletion
TCERG1L	Deletion
C10orf125	Deletion
JAKMIP3	Deletion
STK32C	Deletion
FOXI2	Deletion
FRG2B	Deletion
SPRN	Deletion
NPS	Deletion
LOC619207	Deletion
LOC653544|	Deletion
LOC728410	Deletion
CYB5A	Deletion
GALR1	Deletion
MBP	Deletion
NFATC1	Deletion
ZNF236	Deletion
CTDP1	Deletion
ZNF516	Deletion
TSHZ1	Deletion
TXNL4A	Deletion
ADNP2	Deletion
KCNG2	Deletion
SALL3	Deletion
C18orf55	Deletion
ZNF407	Deletion

CNDP2	Deletion
C18orf22	Deletion
PQLC1	Deletion
NETO1	Deletion
PARD6G	Deletion
CNDP1	Deletion
FAM69C	Deletion
CBLN2	Deletion
FBXO15	Deletion
ZADH2	Deletion
C18orf62	Deletion
LOC284276	Deletion
ATP9B	Deletion
LOC400657	Deletion
HSBP1L1	Deletion
LOC1001305	Deletion
FOXO3	Deletion
NR2E1	Deletion
SNX3	Deletion
CD164	Deletion
SEC63	Deletion
SESN1	Deletion
OSTM1	Deletion
ARMC2	Deletion
LACE1	Deletion
SCML4	Deletion
C6orf182	Deletion
PPIL6	Deletion
hsa-mir-339	Deletion
GNA12	Deletion
GPER	Deletion
LFNG	Deletion
NUDT1	Deletion
PDGFA	Deletion
PRKAR1B	Deletion
MAFK	Deletion
MAD1L1	Deletion
EIF3B	Deletion
KIAA0415	Deletion
ADAP1	Deletion
IQCE	Deletion
SUN1	Deletion
INTS1	Deletion
SNX8	Deletion
FTSJ2	Deletion
GET4	Deletion

CYP2W1	Deletion
HEATR2	Deletion
CHST12	Deletion
RADIL	Deletion
PAPOLB	Deletion
FAM20C	Deletion
MICALL2	Deletion
TTYH3	Deletion
PSMG3	Deletion
C7orf50	Deletion
CARD11	Deletion
ZFAND2A	Deletion
COX19	Deletion
KIAA1908	Deletion
GPR146	Deletion
AMZ1	Deletion
TMEM184A	Deletion
C7orf27	Deletion
SDK1	Deletion
FOXK1	Deletion
MMD2	Deletion
TFAMP1	Deletion
UNCX	Deletion
ELFN1	Deletion
RNF216L	Deletion
hsa-mir-425(	Deletion
hsa-mir-760	Deletion
hsa-mir-320l	Deletion
hsa-mir-553	Deletion
hsa-mir-137	Deletion
hsa-mir-197	Deletion
ABCA4	Deletion
ADORA3	Deletion
AGL	Deletion
ALX3	Deletion
AMPD1	Deletion
AMPD2	Deletion
AMY1A|276|	Deletion
AMY2A	Deletion
AMY2B	Deletion
RHOC	Deletion
ATP1A1	Deletion
ATP5F1	Deletion
BRDT	Deletion
CAPZA1	Deletion
CASQ2	Deletion

CD2	Deletion
CD53	Deletion
CD58	Deletion
CHI3L2	Deletion
CLCA1	Deletion
CNN3	Deletion
COL11A1	Deletion
CSF1	Deletion
CTBS	Deletion
DBT	Deletion
DPYD	Deletion
DR1	Deletion
S1PR1	Deletion
CELSR2	Deletion
EXTL2	Deletion
F3	Deletion
GBP1	Deletion
GBP2	Deletion
GBP3	Deletion
GFI1	Deletion
GCLM	Deletion
GNAI3	Deletion
GNAT2	Deletion
GNG5	Deletion
GSTM1	Deletion
GSTM2	Deletion
GSTM3	Deletion
GSTM4	Deletion
GSTM5	Deletion
GTF2B	Deletion
IGSF3	Deletion
CYR61	Deletion
KCNA2	Deletion
KCNA3	Deletion
KCNA10	Deletion
KCNC4	Deletion
KCND3	Deletion
MOV10	Deletion
NGF	Deletion
NHLH2	Deletion
NRAS	Deletion
OVGP1	Deletion
PRKACB	Deletion
PKN2	Deletion
PSMA5	Deletion
ABCD3	Deletion

RAP1A	Deletion
SNORD21	Deletion
RPL5	Deletion
SORT1	Deletion
SARS	Deletion
SLC16A1	Deletion
STXBP3	Deletion
SYCP1	Deletion
TAF13	Deletion
TGFBR3	Deletion
TSHB	Deletion
VCAM1	Deletion
WNT2B	Deletion
CSDE1	Deletion
EVI5	Deletion
CDC7	Deletion
BCAR3	Deletion
LMO4	Deletion
CDC14A	Deletion
RTCD1	Deletion
BCL10	Deletion
SLC16A4	Deletion
41167	Deletion
ARHGAP29	Deletion
CLCA3P	Deletion
CLCA2	Deletion
HS2ST1	Deletion
LRIG2	Deletion
LPPR4	Deletion
TSPAN2	Deletion
BCAS2	Deletion
CEPT1	Deletion
VAV3	Deletion
HBXIP	Deletion
AP4B1	Deletion
PHTF1	Deletion
AHCYL1	Deletion
GLMN	Deletion
DDX20	Deletion
CLCA4	Deletion
MTF2	Deletion
NTNG1	Deletion
WDR47	Deletion
CLCC1	Deletion
KIAA1107	Deletion
SLC35A3	Deletion

LRRC8B	Deletion
LPAR3	Deletion
DDAH1	Deletion
RWDD3	Deletion
PTPN22	Deletion
SNORA66	Deletion
CHIA	Deletion
GPSM2	Deletion
SLC25A24	Deletion
DNTTIP2	Deletion
TMED5	Deletion
SH3GLB1	Deletion
SNX7	Deletion
TRIM33	Deletion
DPH5	Deletion
GPR88	Deletion
CCDC76	Deletion
RSBN1	Deletion
ZNHIT6	Deletion
PALMD	Deletion
FNBP1L	Deletion
ST7L	Deletion
PRPF38B	Deletion
LRRC8D	Deletion
PRMT6	Deletion
MCOLN3	Deletion
SLC22A15	Deletion
RNPC3	Deletion
C1orf103	Deletion
CTTNBP2NL	Deletion
C1orf183	Deletion
CCBL2	Deletion
TMEM167B	Deletion
OLFML3	Deletion
AMIGO1	Deletion
ODF2L	Deletion
KIAA1324	Deletion
PTBP2	Deletion
DNASE2B	Deletion
SPATA1	Deletion
HIAT1	Deletion
RBM15	Deletion
DCLRE1B	Deletion
WDR77	Deletion
EPS8L3	Deletion
RPAP2	Deletion

DENND2D	Deletion
RPF1	Deletion
SIKE1	Deletion
VANGL1	Deletion
GPR61	Deletion
SYDE2	Deletion
ZNF644	Deletion
LRRC8C	Deletion
PROK1	Deletion
PSRC1	Deletion
C1orf203	Deletion
FAM40A	Deletion
C1orf59	Deletion
GBP4	Deletion
GBP5	Deletion
SSX2IP	Deletion
OLFM3	Deletion
WDR63	Deletion
C1orf161	Deletion
SLC44A3	Deletion
ATXN7L2	Deletion
C1orf194	Deletion
LRRC39	Deletion
DRAM2	Deletion
C1orf88	Deletion
C1orf162	Deletion
SYT6	Deletion
SAMD13	Deletion
C1orf52	Deletion
TMEM56	Deletion
NBPF4	Deletion
SLC30A7	Deletion
LOC149620	Deletion
DENND2C	Deletion
GBP6	Deletion
LPPR5	Deletion
FNDC7	Deletion
SASS6	Deletion
HFM1	Deletion
UBL4B	Deletion
ALG14	Deletion
HIPK1	Deletion
AFARP1	Deletion
EPHX4	Deletion
AKNAD1	Deletion
MCOLN2	Deletion

COL24A1	Deletion
MAGI3	Deletion
FAM19A3	Deletion
FAM102B	Deletion
SYPL2	Deletion
CYB561D1	Deletion
ZNF326	Deletion
BTBD8	Deletion
PPM1J	Deletion
LOC339524	Deletion
CCDC18	Deletion
MYBPHL	Deletion
BARHL2	Deletion
HSP90B3P	Deletion
GBP7	Deletion
C1orf146	Deletion
FAM69A	Deletion
SLC6A17	Deletion
UOX	Deletion
FRRS1	Deletion
LOC400759	Deletion
C1orf180	Deletion
BCL2L15	Deletion
GEMIN8P4	Deletion
RBMXL1	Deletion
CYMP	Deletion
LOC648740	Deletion
NBPF6	Deletion
SCARNA2	Deletion
TUBA3C	Deletion
ZMYM2	Deletion
ZMYM5	Deletion
MPHOSPH8	Deletion
PSPC1	Deletion
TPTE2	Deletion
DKFZp686A1	Deletion
LOC284232	Deletion
LOC348021	Deletion
LOC1001019	Deletion
hsa-mir-430	Deletion
hsa-mir-117	Deletion
ACADS	Deletion
BCL7A	Deletion
SCARB1	Deletion
COX6A1	Deletion
EIF2B1	Deletion

STX2	Deletion
GOLGA3	Deletion
GTF2H3	Deletion
HPD	Deletion
MMP17	Deletion
MSI1	Deletion
P2RX4	Deletion
P2RX7	Deletion
PEBP1	Deletion
PLA2G1B	Deletion
POLE	Deletion
PRKAB1	Deletion
PSMD9	Deletion
PXMP2	Deletion
PXN	Deletion
RAN	Deletion
RFC5	Deletion
RPLP0	Deletion
CLIP1	Deletion
SFRS8	Deletion
HNF1A	Deletion
UBC	Deletion
ZNF10	Deletion
ZNF26	Deletion
ZNF84	Deletion
ZNF140	Deletion
CDK2AP1	Deletion
ULK1	Deletion
DENR	Deletion
OASL	Deletion
DYNLL1	Deletion
SFRS9	Deletion
GPR109B	Deletion
HIP1R	Deletion
PIWIL1	Deletion
CABP1	Deletion
NCOR2	Deletion
KNTC1	Deletion
MLEC	Deletion
RNF10	Deletion
MPHOSPH9	Deletion
CAMKK2	Deletion
ZNF268	Deletion
TMED2	Deletion
GCN1L1	Deletion
RAB35	Deletion

SNRNP35	Deletion
CIT	Deletion
FZD10	Deletion
MLXIP	Deletion
P2RX2	Deletion
SETD1B	Deletion
ANKLE2	Deletion
SIRT4	Deletion
ABCB9	Deletion
RIMBP2	Deletion
ATP6V0A2	Deletion
HSPB8	Deletion
GPR81	Deletion
GALNT9	Deletion
ARL6IP4	Deletion
TAOK3	Deletion
POP5	Deletion
ANAPC5	Deletion
TRIAP1	Deletion
RHOF	Deletion
VSIG10	Deletion
SBNO1	Deletion
ZCCHC8	Deletion
CHFR	Deletion
WSB2	Deletion
DIABLO	Deletion
PITPNM2	Deletion
EP400	Deletion
DHX37	Deletion
FBRSL1	Deletion
DDX55	Deletion
SUDS3	Deletion
C12orf43	Deletion
VPS33A	Deletion
RSRC2	Deletion
AACS	Deletion
NOC4L	Deletion
B3GNT4	Deletion
OGFOD2	Deletion
VPS37B	Deletion
TCTN2	Deletion
RNF34	Deletion
CCDC92	Deletion
PUS1	Deletion
COQ5	Deletion
SRRM4	Deletion

CCDC62	Deletion
KDM2B	Deletion
UNC119B	Deletion
ORAI1	Deletion
C12orf65	Deletion
TMEM132C	Deletion
CCDC64	Deletion
MGC16384	Deletion
TMEM132B	Deletion
LOC116437	Deletion
TMEM132D	Deletion
SLC15A4	Deletion
SPPL3	Deletion
BRI3BP	Deletion
FAM101A	Deletion
ZNF664	Deletion
TMEM120B	Deletion
WDR66	Deletion
GLT1D1	Deletion
LOC144742	Deletion
CCDC60	Deletion
PGAM5	Deletion
RILPL2	Deletion
DNAH10	Deletion
LRRC43	Deletion
GPR133	Deletion
MORN3	Deletion
NME2P1	Deletion
GATC	Deletion
C12orf27	Deletion
DDX51	Deletion
GPR109A	Deletion
LOC338799	Deletion
EP400NL|34	Deletion
RILPL1	Deletion
IL31	Deletion
TMEM233	Deletion
SETD8	Deletion
SNORA49	Deletion
LOC1001285	Deletion
LOC1001302	Deletion
LOC1001909	Deletion
ZNF605	Deletion
EFNA5	Deletion
RAB9BP1	Deletion
PTPRD	Deletion

CSNK2A1	Deletion
SOX12	Deletion
TCF15	Deletion
RBCK1	Deletion
TRIB3	Deletion
NRSN2	Deletion
DEFB126	Deletion
ZCCHC3	Deletion
SCRT2	Deletion
C20orf54	Deletion
TBC1D20	Deletion
C20orf96	Deletion
SRXN1	Deletion
DEFB127	Deletion
DEFB129	Deletion
DEFB125	Deletion
DEFB128	Deletion
DEFB132	Deletion
hsa-mir-1826	Deletion
DNAJA2	Deletion
ZNF267	Deletion
ORC6L	Deletion
VPS35	Deletion
SHCBP1	Deletion
GPT2	Deletion
MYLK3	Deletion
ANKRD26P1	Deletion
LOC146481	Deletion
LOC283914	Deletion
SLC6A10P	Deletion
C16orf87	Deletion
HERC2P4	Deletion
UBE2MP1	Deletion
TP53TG3B|7	Deletion
hsa-mir-4310	Deletion
hsa-mir-1282	Deletion
hsa-mir-147b	Deletion
hsa-mir-627	Deletion
hsa-mir-626	Deletion
B2M	Deletion
BUB1B	Deletion
CAPN3	Deletion
CKMT1B	Deletion
CYP19A1	Deletion
DUT	Deletion
EPB42	Deletion

FBN1	Deletion
FGF7	Deletion
GABPB1	Deletion
GALK2	Deletion
GANC	Deletion
GATM	Deletion
GCHFR	Deletion
PDIA3	Deletion
HDC	Deletion
ITPKA	Deletion
IVD	Deletion
LTK	Deletion
MAP1A	Deletion
MEIS2	Deletion
MFAP1	Deletion
PLCB2	Deletion
MAPK6	Deletion
RAD51	Deletion
SLC12A1	Deletion
SORD	Deletion
SPINT1	Deletion
SRP14	Deletion
THBS1	Deletion
TP53BP1	Deletion
TYRO3	Deletion
SLC30A4	Deletion
EIF3J	Deletion
JMJD7-PLA2(	Deletion
SNAP23	Deletion
USP8	Deletion
SLC28A2	Deletion
COPS2	Deletion
TGM5	Deletion
PPIP5K1	Deletion
SECISBP2L	Deletion
LCMT2	Deletion
BCL2L10	Deletion
RASGRP1	Deletion
SERF2	Deletion
GNB5	Deletion
SLC27A2	Deletion
GPR176	Deletion
CHP	Deletion
OIP5	Deletion
BAHD1	Deletion
CEP152	Deletion

MAPKBP1	Deletion
RTF1	Deletion
MGA	Deletion
DMXL2	Deletion
VPS39	Deletion
AP4E1	Deletion
CCNDBP1	Deletion
EID1	Deletion
C15orf63	Deletion
TMEM87A	Deletion
RPAP1	Deletion
PLDN	Deletion
RPUSD2	Deletion
TUBGCP4	Deletion
SCG3	Deletion
TMOD3	Deletion
TMOD2	Deletion
EHD4	Deletion
DUOX2	Deletion
MYEF2	Deletion
NDUFAF1	Deletion
NUSAP1	Deletion
SPTBN5	Deletion
CTDSPL2	Deletion
DUOX1	Deletion
DLL4	Deletion
INO80	Deletion
TRPM7	Deletion
PPP1R14D	Deletion
ZNF770	Deletion
FLJ10038	Deletion
HAUS2	Deletion
FAM82A2	Deletion
DNAJC17	Deletion
PAK6	Deletion
DTWD1	Deletion
CASC5	Deletion
VPS18	Deletion
SQRDL	Deletion
ZFP106	Deletion
SPATA5L1	Deletion
CHAC1	Deletion
ATP8B4	Deletion
WDR76	Deletion
TMEM62	Deletion
SEMA6D	Deletion

SPG11	Deletion
ELL3	Deletion
C15orf48	Deletion
C15orf41	Deletion
SPPL2A	Deletion
ZFYVE19	Deletion
FRMD5	Deletion
DISP2	Deletion
ATPBD4	Deletion
C15orf57	Deletion
C15orf23	Deletion
BMF	Deletion
SHF	Deletion
DUOXA1	Deletion
CHST14	Deletion
CASC4	Deletion
TGM7	Deletion
CATSPER2	Deletion
LEO1	Deletion
PLA2G4E	Deletion
TRIM69	Deletion
C15orf43	Deletion
LOC145845	Deletion
TMCO5A	Deletion
ZSCAN29	Deletion
TTBK2	Deletion
CDAN1	Deletion
STRC	Deletion
CSNK1A1P	Deletion
SPRED1	Deletion
ADAL	Deletion
EXD1	Deletion
FSIP1	Deletion
RHOV	Deletion
C15orf33	Deletion
UBR1	Deletion
PATL2	Deletion
PLA2G4F	Deletion
LRRC57	Deletion
LYSMD2	Deletion
C15orf21	Deletion
SLC24A5	Deletion
FAM98B	Deletion
PLA2G4D	Deletion
GLDN	Deletion
MRPL42P5	Deletion

USP50	Deletion
C15orf52	Deletion
TNFAIP8L3	Deletion
SHC4	Deletion
CTXN2	Deletion
C15orf53	Deletion
C15orf54	Deletion
DUOXA2	Deletion
EIF2AK4	Deletion
CATSPER2P1	Deletion
CKMT1A	Deletion
SERINC4	Deletion
C15orf62	Deletion
C15orf56	Deletion
PHGR1	Deletion
LOC723972	Deletion
LOC728758	Deletion
LOC729082	Deletion
LOC1001293	Deletion
LOC1001327	Deletion
hsa-mir-614	Deletion
hsa-mir-613	Deletion
ARHGDIB	Deletion
ART4	Deletion
CDKN1B	Deletion
CREBL2	Deletion
EMP1	Deletion
EPS8	Deletion
ETV6	Deletion
GPR19	Deletion
GRIN2B	Deletion
GUCY2C	Deletion
LRP6	Deletion
MGP	Deletion
MGST1	Deletion
PDE6H	Deletion
PRB1	Deletion
PRB3	Deletion
PRB4	Deletion
PRH1	Deletion
PRH2	Deletion
PTPRO	Deletion
CSDA	Deletion
GPRC5A	Deletion
STRAP	Deletion
PRR4	Deletion

TAS2R9	Deletion
TAS2R8	Deletion
TAS2R7	Deletion
TAS2R13	Deletion
TAS2R10	Deletion
TAS2R14	Deletion
HEBP1	Deletion
DERA	Deletion
DDX47	Deletion
WBP11	Deletion
MANSC1	Deletion
MAGOHB	Deletion
STYK1	Deletion
GPRC5D	Deletion
ATF7IP	Deletion
H2AFJ	Deletion
LMO3	Deletion
KIAA1467	Deletion
BCL2L14	Deletion
RERGL	Deletion
PLBD1	Deletion
DUSP16	Deletion
APOLD1	Deletion
GSG1	Deletion
RERG	Deletion
HTR7P1	Deletion
LOH12CR1	Deletion
HIST4H4	Deletion
ERP27	Deletion
C12orf60	Deletion
TAS2R43	Deletion
TAS2R31	Deletion
TAS2R46	Deletion
TAS2R30	Deletion
TAS2R19	Deletion
TAS2R20	Deletion
TAS2R50	Deletion
C12orf36	Deletion
TAS2R42	Deletion
RPL13AP20	Deletion
C12orf69	Deletion
LOH12CR2	Deletion
PRB2	Deletion
hsa-mir-129[illegible]	Deletion
PGM5	Deletion
ZNF658	Deletion

CNTNAP3	Deletion
C9orf122	Deletion
CBWD5	Deletion
FOXD4L3	Deletion
AQP7P1	Deletion
KGFLP1	Deletion
FAM75A6	Deletion
MGC21881	Deletion
FAM74A1	Deletion
FAM74A4	Deletion
LOC440896	Deletion
ANKRD20A3|	Deletion
AQP7P3	Deletion
LOC442421	Deletion
CBWD3|445!	Deletion
FAM27A	Deletion
LOC572558	Deletion
PGM5P2	Deletion
FAM75A2|64	Deletion
LOC642929	Deletion
CBWD6	Deletion
FOXD4L6	Deletion
FOXD4L5	Deletion
LOC653501|(	Deletion
KGFLP2	Deletion
FAM75A3	Deletion
FAM75A5|72	Deletion
FAM74A3	Deletion
ANKRD20A4	Deletion
?|728788	Deletion
FOXD4L2|10	Deletion
FAM27C	Deletion
FAM95B1|10	Deletion
FAM27B	Deletion
LOC1001339	Deletion
hsa-mir-663k	Deletion
hsa-mir-128-	Deletion
ACVR2A	Deletion
BIN1	Deletion
RND3	Deletion
CACNB4	Deletion
CCNT2	Deletion
DARS	Deletion
ERCC3	Deletion
GPR17	Deletion
GPR39	Deletion

GYPC	Deletion
HNMT	Deletion
KCNJ3	Deletion
KIF5C	Deletion
LCT	Deletion
MCM6	Deletion
MGAT5	Deletion
MYO7B	Deletion
NEB	Deletion
NR4A2	Deletion
ORC4L	Deletion
POLR2D	Deletion
PROC	Deletion
TNFAIP6	Deletion
CXCR4	Deletion
KYNU	Deletion
NMI	Deletion
HS6ST1	Deletion
ZEB2	Deletion
STAM2	Deletion
MAP3K2	Deletion
NXPH2	Deletion
RAB3GAP1	Deletion
UBXN4	Deletion
R3HDM1	Deletion
EPC2	Deletion
ARL5A	Deletion
PTPN18	Deletion
MMADHC	Deletion
C2orf27A	Deletion
ARHGEF4	Deletion
LRP1B	Deletion
PLEKHB2	Deletion
RIF1	Deletion
WDR33	Deletion
SMPD4	Deletion
PRPF40A	Deletion
IWS1	Deletion
LIMS2	Deletion
MBD5	Deletion
ARHGAP15	Deletion
RPRM	Deletion
UGGT1	Deletion
SAP130	Deletion
GTDC1	Deletion
FAM128B	Deletion

YSK4	Deletion
THSD7B	Deletion
TMEM163	Deletion
AMMECR1L	Deletion
ZRANB3	Deletion
RAB6C	Deletion
CCDC115	Deletion
SFT2D3	Deletion
CCDC74A	Deletion
CCDC74B	Deletion
IMP4	Deletion
TUBA3E	Deletion
TUBA3D	Deletion
FMNL2	Deletion
GALNT13	Deletion
LYPD1	Deletion
CNTNAP5	Deletion
ACMSD	Deletion
FAM168B	Deletion
LYPD6	Deletion
LYPD6B	Deletion
LOC150527|[illegible]	Deletion
LOC150776	Deletion
LOC150786	Deletion
LOC151162	Deletion
ARL6IP6	Deletion
FAM123C	Deletion
SPOPL	Deletion
CYP27C1	Deletion
NCKAP5	Deletion
GPR148	Deletion
RBM43	Deletion
LOC389033	Deletion
LOC401010	Deletion
C2orf27B	Deletion
LOC440905	Deletion
POTEE	Deletion
NCRNA0016[illegible]	Deletion
CFC1B|6532[illegible]	Deletion
FAM128A	Deletion
POTEF	Deletion
PABPC1P2	Deletion
C2orf14	Deletion
ACCN1	Deletion
AP2B1	Deletion
LIG3	Deletion

PEX12	Deletion
RAD51L3	Deletion
CCL1	Deletion
CCL2	Deletion
CCL7	Deletion
CCL8	Deletion
CCL11	Deletion
CCL13	Deletion
CCT6B	Deletion
NLE1	Deletion
FNDC8	Deletion
SLFN12	Deletion
ZNF830	Deletion
SLFN11	Deletion
RASL10B	Deletion
RFFL	Deletion
TMEM132E	Deletion
SLFN13	Deletion
AMAC1	Deletion
UNC45B	Deletion
SLFN5	Deletion
SLFN12L	Deletion
SLFN14	Deletion
C17orf102	Deletion
SNORD7	Deletion
hsa-mir-1237	Deletion
hsa-mir-612	Deletion
hsa-mir-194-	Deletion
hsa-mir-192	Deletion
ARL2	Deletion
BAD	Deletion
C11orf2	Deletion
MRPL49	Deletion
ZNHIT2	Deletion
CAPN1	Deletion
CFL1	Deletion
CTSW	Deletion
ESRRA	Deletion
FAU	Deletion
FKBP2	Deletion
DNAJC4	Deletion
LTBP3	Deletion
MEN1	Deletion
MAP3K11	Deletion
OVOL1	Deletion
PLCB3	Deletion

PPP2R5B	Deletion
PYGM	Deletion
MAP4K2	Deletion
RELA	Deletion
DPF2	Deletion
SIPA1	Deletion
TM7SF2	Deletion
VEGFB	Deletion
SF1	Deletion
ZFPL1	Deletion
FOSL1	Deletion
RPS6KA4	Deletion
FIBP	Deletion
NRXN2	Deletion
NAALADL1	Deletion
KCNK7	Deletion
RASGRP2	Deletion
CDC42EP2	Deletion
KAT5	Deletion
SSSCA1	Deletion
EHD1	Deletion
STIP1	Deletion
CCDC85B	Deletion
ATG2A	Deletion
FAM89B	Deletion
POLA2	Deletion
PRDX5	Deletion
C11orf20	Deletion
PPP1R14B	Deletion
SAC3D1	Deletion
SNX15	Deletion
EFEMP2	Deletion
KCNK4	Deletion
TRMT112	Deletion
CDC42BPG	Deletion
SLC22A11	Deletion
GPR137	Deletion
SCYL1	Deletion
MUS81	Deletion
FERMT3	Deletion
TRPT1	Deletion
FRMD8	Deletion
RNASEH2C	Deletion
NUDT22	Deletion
SYVN1	Deletion
DKFZp761E1[illegible]	Deletion

CDCA5	Deletion
BATF2	Deletion
SLC22A12	Deletion
GPHA2	Deletion
TIGD3	Deletion
EHBP1L1	Deletion
SNX32	Deletion
C11orf85	Deletion
SLC25A45	Deletion
NEAT1	Deletion
CCDC88B	Deletion
MALAT1	Deletion
SPDYC	Deletion
PCNXL3	Deletion
SLC22A20	Deletion
FNTA	Deletion
SGK196	Deletion
HGSNAT	Deletion
POTEA	Deletion
hsa-mir-4273	Deletion
hsa-mir-4272	Deletion
hsa-mir-3136	Deletion
hsa-mir-1324	Deletion
hsa-mir-1284	Deletion
hsa-mir-135a	Deletion
hsa-let-7g	Deletion
ALAS1	Deletion
ARF4	Deletion
C3orf51	Deletion
CACNA1D	Deletion
DNASE1L3	Deletion
FHIT	Deletion
FLNB	Deletion
GBE1	Deletion
GPR27	Deletion
HTR1F	Deletion
ITIH1	Deletion
ITIH3	Deletion
ITIH4	Deletion
MITF	Deletion
CNTN3	Deletion
PDHB	Deletion
POU1F1	Deletion
PRKCD	Deletion
PTPRG	Deletion
ROBO1	Deletion

ROBO2	Deletion
ATXN7	Deletion
NEK4	Deletion
TKT	Deletion
TMF1	Deletion
TNNC1	Deletion
WNT5A	Deletion
SLMAP	Deletion
ACOX2	Deletion
BAP1	Deletion
CGGBP1	Deletion
CADPS	Deletion
SUCLG2	Deletion
HESX1	Deletion
UBA3	Deletion
MAGI1	Deletion
PSMD6	Deletion
ARL6IP5	Deletion
RPP14	Deletion
FAM107A	Deletion
NISCH	Deletion
TWF2	Deletion
PDZRN3	Deletion
FRMD4B	Deletion
STAB1	Deletion
C3orf63	Deletion
RYBP	Deletion
CHMP2B	Deletion
DNAH1	Deletion
LRIG1	Deletion
ERC2	Deletion
APPL1	Deletion
GNL3	Deletion
FOXP1	Deletion
SPCS1	Deletion
ARHGEF3	Deletion
SFMBT1	Deletion
PHF7	Deletion
TLR9	Deletion
IL17RD	Deletion
PXK	Deletion
FEZF2	Deletion
FLJ10213	Deletion
SHQ1	Deletion
PBRM1	Deletion
ZNF654	Deletion

CHDH	Deletion
IL17RB	Deletion
CACNA2D3	Deletion
DCP1A	Deletion
GLT8D1	Deletion
LMOD3	Deletion
SEMA3G	Deletion
ADAMTS9	Deletion
ABHD6	Deletion
LRTM1	Deletion
C3orf14	Deletion
SELK	Deletion
PROK2	Deletion
NT5DC2	Deletion
THOC7	Deletion
WDR82	Deletion
ID2B	Deletion
KBTBD8	Deletion
RFT1	Deletion
ACTR8	Deletion
SLC25A26	Deletion
FAM3D	Deletion
GLYCTK	Deletion
PPM1M	Deletion
C3orf49	Deletion
SNTN	Deletion
SYNPR	Deletion
ASB14	Deletion
FAM19A4	Deletion
PPP4R2	Deletion
PRICKLE2	Deletion
C3orf67	Deletion
KCTD6	Deletion
DNAH12	Deletion
PDE12	Deletion
FAM116A	Deletion
CADM2	Deletion
C3orf64	Deletion
C3orf38	Deletion
CCDC66	Deletion
LOC285401	Deletion
EIF4E3	Deletion
SPATA12	Deletion
TMEM110	Deletion
MUSTN1	Deletion
VGLL3	Deletion

FAM19A1	Deletion
LOC440957	Deletion
SNORD19	Deletion
FAM86D	Deletion
SNORD69	Deletion
GXYLT2	Deletion
HESRG	Deletion
SNORD19B	Deletion
ZNF717	Deletion
FRG2C	Deletion
hsa-mir-428	Deletion
hsa-mir-428	Deletion
hsa-mir-314	Deletion
hsa-mir-548	Deletion
ADRA1A	Deletion
BNIP3L	Deletion
CHRNA2	Deletion
CLU	Deletion
DPYSL2	Deletion
DUSP4	Deletion
EGR3	Deletion
EPHX2	Deletion
EXTL3	Deletion
PTK2B	Deletion
GNRH1	Deletion
GSR	Deletion
GTF2E2	Deletion
NRG1	Deletion
LOXL2	Deletion
NEFM	Deletion
NEFL	Deletion
NKX3-1	Deletion
PNOC	Deletion
PPP2CB	Deletion
PPP2R2A	Deletion
PPP3CC	Deletion
STC1	Deletion
WRN	Deletion
FZD3	Deletion
UBXN8	Deletion
ADAM7	Deletion
TNFRSF10D	Deletion
TNFRSF10C	Deletion
TNFRSF10B	Deletion
TNFRSF10A	Deletion
ENTPD4	Deletion

SORBS3	Deletion
DCTN6	Deletion
PNMA2	Deletion
ADAM28	Deletion
RBPMS	Deletion
TRIM35	Deletion
RHOBTB2	Deletion
KIF13B	Deletion
LEPROTL1	Deletion
SLC39A14	Deletion
ADAMDEC1	Deletion
PURG	Deletion
SLC25A37	Deletion
SCARA3	Deletion
TMEM66	Deletion
KCTD9	Deletion
PIWIL2	Deletion
ELP3	Deletion
CCDC25	Deletion
INTS9	Deletion
PBK	Deletion
ZNF395	Deletion
BIN3	Deletion
TEX15	Deletion
KIAA1967	Deletion
PDLIM2	Deletion
EBF2	Deletion
DUSP26	Deletion
HMBOX1	Deletion
RNF122	Deletion
DOCK5	Deletion
FLJ14107	Deletion
C8orf41	Deletion
STMN4	Deletion
MAK16	Deletion
FUT10	Deletion
CHMP7	Deletion
NKX2-6	Deletion
UNC5D	Deletion
PEBP4	Deletion
CDCA2	Deletion
ESCO2	Deletion
FBXO16	Deletion
R3HCC1	Deletion
SCARA5	Deletion
LOC286135	Deletion

C8orf80	Deletion
C8orf58	Deletion
C8orf75	Deletion
MBOAT4	Deletion
hsa-mir-3201	Deletion
hsa-mir-320(	Deletion
hsa-mir-319(	Deletion
hsa-mir-319(	Deletion
hsa-mir-3198	Deletion
hsa-mir-130(	Deletion
hsa-mir-1281	Deletion
hsa-mir-124(	Deletion
hsa-mir-128(	Deletion
hsa-mir-548j	Deletion
hsa-mir-301t	Deletion
hsa-mir-659	Deletion
hsa-mir-658	Deletion
hsa-mir-650	Deletion
hsa-mir-649	Deletion
hsa-mir-648	Deletion
hsa-mir-130t	Deletion
hsa-mir-185	Deletion
hsa-mir-33a	Deletion
hsa-let-7b	Deletion
hsa-let-7a-3	Deletion
ACR	Deletion
ACO2	Deletion
ADORA2A	Deletion
ADRBK2	Deletion
ADSL	Deletion
AP1B1	Deletion
ARSA	Deletion
ARVCF	Deletion
ATF4	Deletion
ATP6V1E1	Deletion
BCR	Deletion
BID	Deletion
BIK	Deletion
TSPO	Deletion
MPPED1	Deletion
CHKB	Deletion
COMT	Deletion
CPT1B	Deletion
CRKL	Deletion
CRYBA4	Deletion
CRYBB1	Deletion

CRYBB2	Deletion
CRYBB3	Deletion
CSF2RB	Deletion
CSNK1E	Deletion
CYP2D7P1	Deletion
CYP2D6	Deletion
DDT	Deletion
CYB5R3	Deletion
TYMP	Deletion
EP300	Deletion
EWSR1	Deletion
FBLN1	Deletion
XRCC6	Deletion
GGT1	Deletion
GGT3P	Deletion
GGT5	Deletion
GNAZ	Deletion
MCHR1	Deletion
GSC2	Deletion
GSTT1	Deletion
GSTT2|2953	Deletion
H1F0	Deletion
SERPIND1	Deletion
HMOX1	Deletion
IGLL1	Deletion
IL2RB	Deletion
KCNJ4	Deletion
LGALS1	Deletion
LGALS2	Deletion
LIF	Deletion
LIMK2	Deletion
MB	Deletion
MCM5	Deletion
MFNG	Deletion
MGAT3	Deletion
MIF	Deletion
MMP11	Deletion
MN1	Deletion
MPST	Deletion
MYH9	Deletion
NAGA	Deletion
NCF4	Deletion
NDUFA6	Deletion
DRG1	Deletion
NEFH	Deletion
NF2	Deletion

NHP2L1	Deletion
OSM	Deletion
PDGFB	Deletion
PI4KA	Deletion
PMM1	Deletion
41157	Deletion
POLR2F	Deletion
PPARA	Deletion
MAPK1	Deletion
MAPK11	Deletion
PRODH	Deletion
PVALB	Deletion
RAC2	Deletion
RANBP1	Deletion
RANGAP1	Deletion
RFPL1	Deletion
RPL3	Deletion
MAPK12	Deletion
SBF1	Deletion
SLC5A1	Deletion
SMTN	Deletion
SLC5A4	Deletion
SLC7A4	Deletion
SLC25A1	Deletion
SMARCB1	Deletion
SNRPD3	Deletion
SOX10	Deletion
SREBF2	Deletion
SSTR3	Deletion
ST13	Deletion
TBX1	Deletion
TCF20	Deletion
TCN2	Deletion
TEF	Deletion
TIMP3	Deletion
CLDN5	Deletion
TOP1P2	Deletion
TST	Deletion
HIRA	Deletion
UBE2L3	Deletion
UFD1L	Deletion
UPK3A	Deletion
VPREB1	Deletion
WNT7B	Deletion
XBP1	Deletion
YWHAH	Deletion

ZNF70	Deletion
ZNF74	Deletion
DGCR6	Deletion
LZTR1	Deletion
CLTCL1	Deletion
DGCR14	Deletion
SYN3	Deletion
CDC45	Deletion
PLA2G6	Deletion
TPST2	Deletion
GALR3	Deletion
NIPSNAP1	Deletion
APOL1	Deletion
THOC5	Deletion
EIF3D	Deletion
MTMR3	Deletion
CACNA1I	Deletion
TOP3B	Deletion
P2RX6	Deletion
SYNGR1	Deletion
LARGE	Deletion
SNAP29	Deletion
GRAP2	Deletion
PICK1	Deletion
GAL3ST1	Deletion
GTPBP1	Deletion
APOBEC3B	Deletion
RAB36	Deletion
CELSR1	Deletion
PPM1F	Deletion
DEPDC5	Deletion
SAPS2	Deletion
SFI1	Deletion
ZBED4	Deletion
JOSD1	Deletion
RBX1	Deletion
DGCR2	Deletion
SCO2	Deletion
HMGXB4	Deletion
TOM1	Deletion
DNAL4	Deletion
SF3A1	Deletion
PKDREJ	Deletion
CACNG2	Deletion
TAB1	Deletion
SLC25A17	Deletion

DDX17	Deletion
TXNRD2	Deletion
RASL10A	Deletion
GAS2L1	Deletion
RFPL3S	Deletion
RFPL3	Deletion
RFPL2	Deletion
RFPL1S	Deletion
NUP50	Deletion
TOB2	Deletion
KDELR3	Deletion
IFT27	Deletion
TRIOBP	Deletion
CDC42EP1	Deletion
DMC1	Deletion
RABL2B	Deletion
CHEK2	Deletion
PACSIN2	Deletion
USP18	Deletion
MORC2	Deletion
TNRC6B	Deletion
HIC2	Deletion
GRAMD4	Deletion
TTLL12	Deletion
MLC1	Deletion
ZC3H7B	Deletion
C22orf9	Deletion
TTC28	Deletion
CYTSA	Deletion
GCAT	Deletion
CBX6	Deletion
NPTXR	Deletion
PES1	Deletion
CBX7	Deletion
CABIN1	Deletion
PRAME	Deletion
SLC16A8	Deletion
SEC14L2	Deletion
MAPK8IP2	Deletion
RBM9	Deletion
SEZ6L	Deletion
RASD2	Deletion
PATZ1	Deletion
SH3BP1	Deletion
TSSK2	Deletion
PLXNB2	Deletion

SDF2L1	Deletion
PPIL2	Deletion
PITPNB	Deletion
PISD	Deletion
OSBP2	Deletion
MAFF	Deletion
IL17RA	Deletion
BRD1	Deletion
ARHGAP8	Deletion
APOL2	Deletion
POTEH	Deletion
BCL2L13	Deletion
TFIP11	Deletion
C22orf31	Deletion
TBC1D22A	Deletion
GSTTP1	Deletion
C22orf24	Deletion
CBY1	Deletion
SUN2	Deletion
DGCR11	Deletion
DGCR9	Deletion
FBXO7	Deletion
RHBDD3	Deletion
TTLL1	Deletion
POM121L1P	Deletion
SAMM50	Deletion
ATXN10	Deletion
FAM19A5	Deletion
TXN2	Deletion
TMEM184B	Deletion
SULT4A1	Deletion
GGA1	Deletion
RIBC2	Deletion
DGCR5	Deletion
DGCR10	Deletion
ARFGAP3	Deletion
SNORD43	Deletion
TRMT2A	Deletion
INPP5J	Deletion
SMC1B	Deletion
CYTH4	Deletion
RTDR1	Deletion
CSDC2	Deletion
RRP7A	Deletion
MCAT	Deletion
APOBEC3C	Deletion

PPPDE2	Deletion
SGSM3	Deletion
HSFYL1	Deletion
CECR6	Deletion
CECR5	Deletion
CECR2	Deletion
POM121L9P	Deletion
CARD10	Deletion
PARVB	Deletion
NCAPH2	Deletion
UQCR10	Deletion
POM121L8P	Deletion
YPEL1	Deletion
ZDHHC8	Deletion
VPREB3	Deletion
PLA2G3	Deletion
C22orf43	Deletion
EIF3L	Deletion
C22orf28	Deletion
GTSE1	Deletion
MTP18	Deletion
MED15	Deletion
UPB1	Deletion
TUBA8	Deletion
CECR1	Deletion
A4GALT	Deletion
MOV10L1	Deletion
SMCR7L	Deletion
DGCR8	Deletion
GNB1L	Deletion
TUG1	Deletion
FAM118A	Deletion
TTC38	Deletion
C22orf26	Deletion
MIOX	Deletion
PRR5	Deletion
PEX26	Deletion
TRMU	Deletion
ZMAT5	Deletion
41155	Deletion
SUSD2	Deletion
EIF4ENIF1	Deletion
PANX2	Deletion
TOMM22	Deletion
PDXP	Deletion
ASPHD2	Deletion

MICAL3	Deletion
MKL1	Deletion
APOBEC3G	Deletion
XPNPEP3	Deletion
PARVG	Deletion
CERK	Deletion
EFCAB6	Deletion
MRPL40	Deletion
RTN4R	Deletion
SLC2A11	Deletion
CENPM	Deletion
ALG12	Deletion
NOL12	Deletion
CRELD2	Deletion
C22orf46	Deletion
C22orf29	Deletion
KCTD17	Deletion
CCDC134	Deletion
ADM2	Deletion
FOXRED2	Deletion
BAIAP2L2	Deletion
SCUBE1	Deletion
TRABD	Deletion
PNPLA3	Deletion
THAP7	Deletion
APOL6	Deletion
APOL5	Deletion
APOL4	Deletion
APOL3	Deletion
OR11H1	Deletion
C22orf13	Deletion
SELO	Deletion
SLC25A18	Deletion
L3MBTL2	Deletion
TBC1D10A	Deletion
HDAC10	Deletion
KREMEN1	Deletion
ZNRF3	Deletion
ASCC2	Deletion
TMEM191A	Deletion
LDOC1L	Deletion
POLDIP3	Deletion
C22orf23	Deletion
MYO18B	Deletion
PHF5A	Deletion
KLHL22	Deletion

KIAA1644	Deletion
SHANK3	Deletion
DGCR6L	Deletion
RIMBP3	Deletion
MICALL1	Deletion
TUBGCP6	Deletion
KIAA1671	Deletion
HPS4	Deletion
LOC90834	Deletion
SCARF2	Deletion
GGTLC2	Deletion
LMF2	Deletion
LOC91316	Deletion
DERL3	Deletion
IGLL3	Deletion
LRP5L	Deletion
RNF185	Deletion
ISX	Deletion
RPS19BP1	Deletion
C22orf32	Deletion
RRP7B	Deletion
SERHL	Deletion
LOC96610	Deletion
PHF21B	Deletion
MGC16703	Deletion
KLHDC7B	Deletion
PIK3IP1	Deletion
FAM83F	Deletion
ELFN2	Deletion
C1QTNF6	Deletion
TNFRSF13C	Deletion
GAB4	Deletion
C22orf39	Deletion
C22orf25	Deletion
ZNF280A	Deletion
SGSM1	Deletion
EMID1	Deletion
ANKRD54	Deletion
APOBEC3D	Deletion
SELM	Deletion
ZNF280B	Deletion
CCT8L2	Deletion
XKR3	Deletion
LOC150185	Deletion
LOC150197	Deletion
AIFM3	Deletion

RIMBP3C|15	Deletion
YDJC	Deletion
ZDHHC8P1	Deletion
C22orf15	Deletion
HSCB	Deletion
CCDC117	Deletion
HORMAD2	Deletion
DUSP18	Deletion
C22orf27	Deletion
C22orf42	Deletion
ENTHD1	Deletion
DNAJB7	Deletion
CHADL	Deletion
MEI1	Deletion
FAM109B	Deletion
NFAM1	Deletion
PNPLA5	Deletion
LOC150381	Deletion
C22orf40	Deletion
CN5H6.4	Deletion
CCDC116	Deletion
CABP7	Deletion
TMPRSS6	Deletion
APOBEC3H	Deletion
WBP2NL	Deletion
TTLL8	Deletion
POLR3H	Deletion
RNF215	Deletion
APOBEC3A	Deletion
APOBEC3F	Deletion
C22orf30	Deletion
SERHL2	Deletion
BPIL2	Deletion
TMEM211	Deletion
SEC14L3	Deletion
POM121L4P	Deletion
RGL4	Deletion
ATP5L2	Deletion
LOC284900	Deletion
SEC14L4	Deletion
RPL23AP82	Deletion
SLC35E4	Deletion
C22orf33	Deletion
LOC339674	Deletion
C22orf34	Deletion
PI4KAP2	Deletion

SDC4P	Deletion
CHKB-CPT1B	Deletion
psiTPTE22	Deletion
C22orf36	Deletion
NCRNA0020	Deletion
LOC391322	Deletion
LOC400891	Deletion
CHCHD10	Deletion
LOC400927	Deletion
LOC400931	Deletion
IL17REL	Deletion
SRRD	Deletion
FAM116B	Deletion
PIM3	Deletion
FLJ39582	Deletion
P2RX6P	Deletion
PIWIL3	Deletion
MIAT	Deletion
ODF3B	Deletion
CCDC157	Deletion
PRR5-ARHGA	Deletion
LOC644165	Deletion
C22orf41	Deletion
C22orf45|64	Deletion
POM121L10	Deletion
LOC646851	Deletion
LOC648691	Deletion
GATSL3	Deletion
GSTTP2	Deletion
PI4KAP1	Deletion
LOC730668	Deletion
DDTL	Deletion
SNORD125	Deletion
CECR7	Deletion
CECR4	Deletion
LOC1001446	Deletion
FLJ41941	Deletion
LOC1002717	Deletion
CLEC3A	Deletion
WWOX	Deletion
ANKS1B	Deletion
FAM71C	Deletion
hsa-mir-318	Deletion
hsa-mir-22	Deletion
ABR	Deletion
CRK	Deletion

MYO1C	Deletion
SERPINF1	Deletion
PITPNA	Deletion
SERPINF2	Deletion
RPA1	Deletion
YWHAE	Deletion
DOC2B	Deletion
SCARF1	Deletion
RPH3AL	Deletion
PRPF8	Deletion
TIMM22	Deletion
GEMIN4	Deletion
GLOD4	Deletion
INPP5K	Deletion
RNMTL1	Deletion
VPS53	Deletion
NXN	Deletion
FAM57A	Deletion
RILP	Deletion
C17orf91	Deletion
SMYD4	Deletion
SLC43A2	Deletion
WDR81	Deletion
TUSC5	Deletion
FAM101B	Deletion
C17orf97	Deletion
ELP2P	Deletion
hsa-mir-4262	Deletion
hsa-mir-4261	Deletion
hsa-mir-3125	Deletion
hsa-mir-548s	Deletion
hsa-mir-1301	Deletion
ACP1	Deletion
ADCY3	Deletion
APOB	Deletion
RHOB	Deletion
CAD	Deletion
CENPA	Deletion
DDX1	Deletion
DNMT3A	Deletion
DTNB	Deletion
E2F6	Deletion
FKBP1B	Deletion
FTHL3	Deletion
GCKR	Deletion
GTF3C2	Deletion

HADHA	Deletion
HADHB	Deletion
HPCAL1	Deletion
ID2	Deletion
KCNF1	Deletion
KCNK3	Deletion
KCNS3	Deletion
KHK	Deletion
KIF3C	Deletion
MATN3	Deletion
MPV17	Deletion
MYCN	Deletion
ODC1	Deletion
POMC	Deletion
PPM1G	Deletion
RPS7	Deletion
RRM2	Deletion
SDC1	Deletion
SOX11	Deletion
ADAM17	Deletion
TPO	Deletion
TSSC1	Deletion
UCN	Deletion
VSNL1	Deletion
SLC30A3	Deletion
PXDN	Deletion
KLF11	Deletion
NCOA1	Deletion
ASAP2	Deletion
SLC5A6	Deletion
EIF2B4	Deletion
TAF1B	Deletion
ITGB1BP1	Deletion
OTOF	Deletion
ROCK2	Deletion
TP53I3	Deletion
GREB1	Deletion
LAPTM4A	Deletion
RNF144A	Deletion
SNX17	Deletion
SUPT7L	Deletion
PREB	Deletion
PDIA6	Deletion
MYCNOS	Deletion
CGREF1	Deletion
RAB10	Deletion

YWHAQ	Deletion
EMILIN1	Deletion
GPN1	Deletion
MAPRE3	Deletion
SLC4A1AP	Deletion
EFR3B	Deletion
MYT1L	Deletion
LPIN1	Deletion
PUM2	Deletion
NTSR2	Deletion
IFT172	Deletion
SH3YL1	Deletion
TRIB2	Deletion
GRHL1	Deletion
NRBP1	Deletion
ITSN2	Deletion
TTC15	Deletion
DNAJC27	Deletion
C2orf28	Deletion
NBAS	Deletion
SF3B14	Deletion
CPSF3	Deletion
SNTG2	Deletion
ATAD2B	Deletion
TMEM214	Deletion
C2orf18	Deletion
ASXL2	Deletion
ADI1	Deletion
ALLC	Deletion
DPYSL5	Deletion
TRIM54	Deletion
KIDINS220	Deletion
WDR35	Deletion
RDH14	Deletion
AGBL5	Deletion
C2orf43	Deletion
HS1BP3	Deletion
FNDC4	Deletion
COLEC11	Deletion
CENPO	Deletion
CCDC121	Deletion
SMC6	Deletion
NOL10	Deletion
C2orf44	Deletion
FAM49A	Deletion
C2orf16	Deletion

ZNF512	Deletion
ABHD1	Deletion
EPT1	Deletion
RSAD2	Deletion
C2orf39	Deletion
NT5C1B	Deletion
KLHL29	Deletion
CMPK2	Deletion
MBOAT2	Deletion
TMEM18	Deletion
CIB4	Deletion
OSR1	Deletion
TTC32	Deletion
ZNF513	Deletion
C2orf50	Deletion
PQLC3	Deletion
LOC150622	Deletion
TCF23	Deletion
FAM84A	Deletion
GDF7	Deletion
GPR113	Deletion
UBXN2A	Deletion
CYS1	Deletion
KRTCAP3	Deletion
ATP6V1C2	Deletion
RNASEH1	Deletion
FAM150B	Deletion
DNAJC5G	Deletion
IAH1	Deletion
C2orf70	Deletion
C2orf53	Deletion
LOC339788	Deletion
MSGN1	Deletion
GEN1	Deletion
C2orf48	Deletion
PFN4	Deletion
LOC375190	Deletion
MFSD2B	Deletion
?|391343	Deletion
C2orf79	Deletion
LOC400940	Deletion
FAM110C	Deletion
C2orf84	Deletion
RAD51AP2	Deletion
LOC730811	Deletion
OST4	Deletion

hsa-mir-4282	Deletion
hsa-mir-548u	Deletion
hsa-mir-2113	Deletion
hsa-mir-30c-1	Deletion
hsa-mir-30a	Deletion
BAI3	Deletion
BCKDHB	Deletion
CCNC	Deletion
CGA	Deletion
CNR1	Deletion
COL9A1	Deletion
COL12A1	Deletion
COL19A1	Deletion
COX7A2	Deletion
EEF1A1	Deletion
EPHA7	Deletion
GABRR1	Deletion
GABRR2	Deletion
GRIK2	Deletion
HTR1B	Deletion
HTR1E	Deletion
IMPG1	Deletion
ME1	Deletion
MYO6	Deletion
NT5E	Deletion
PGM3	Deletion
POU3F2	Deletion
RNY4	Deletion
SIM1	Deletion
ELOVL4	Deletion
MAP3K7	Deletion
TPBG	Deletion
TTK	Deletion
PTP4A1	Deletion
RNGTT	Deletion
TBX18	Deletion
HMGN3	Deletion
FHL5	Deletion
SNAP91	Deletion
CASP8AP2	Deletion
SYNCRIP	Deletion
SLC35A1	Deletion
FUT9	Deletion
PNRC1	Deletion
ASCC3	Deletion
KIAA1009	Deletion

ANKRD6	Deletion
RIMS1	Deletion
DOPEY1	Deletion
ZNF292	Deletion
MDN1	Deletion
KIAA0776	Deletion
PHF3	Deletion
ORC3L	Deletion
MTO1	Deletion
SFRS18	Deletion
IBTK	Deletion
SENP6	Deletion
FBXL4	Deletion
SLC17A5	Deletion
SNORD50A	Deletion
FILIP1	Deletion
NDUFAF4	Deletion
CYB5R4	Deletion
UBE2J1	Deletion
LGSN	Deletion
COQ3	Deletion
PHIP	Deletion
AKIRIN2	Deletion
NCRNA0012(	Deletion
DDX43	Deletion
FAM46A	Deletion
TMEM30A	Deletion
LMBRD1	Deletion
KCNQ5	Deletion
RARS2	Deletion
C6orf162	Deletion
LYRM2	Deletion
SNX14	Deletion
HACE1	Deletion
FAM135A	Deletion
RRAGD	Deletion
PRDM13	Deletion
BACH2	Deletion
SMAP1	Deletion
C6orf164	Deletion
OGFRL1	Deletion
MANEA	Deletion
C6orf155	Deletion
KHDC1	Deletion
GPR63	Deletion
SPACA1	Deletion

SH3BGRL2	Deletion
MCHR2	Deletion
C6orf168	Deletion
GJA10	Deletion
USP45	Deletion
UBE2CBP	Deletion
MRAP2	Deletion
RWDD2A	Deletion
KLHL32	Deletion
C6orf150	Deletion
RIPPLY2	Deletion
IRAK1BP1	Deletion
B3GAT2	Deletion
C6orf57	Deletion
CD109	Deletion
PM20D2	Deletion
SFRS13B	Deletion
C6orf221	Deletion
C6orf165	Deletion
PRSS35	Deletion
LCA5	Deletion
KHDRBS2	Deletion
C6orf163	Deletion
C6orf167	Deletion
DPPA5	Deletion
EYS	Deletion
GUSBL2	Deletion
GJB7	Deletion
SNHG5	Deletion
C6orf147	Deletion
OOEP	Deletion
MCART3P	Deletion
TSG1	Deletion
SNORD50B	Deletion
KHDC1L	Deletion
AFG3L1	Deletion
APRT	Deletion
C16orf3	Deletion
CA5A	Deletion
CBFA2T3	Deletion
CDH15	Deletion
CYBA	Deletion
DPEP1	Deletion
FANCA	Deletion
GALNS	Deletion
GAS8	Deletion

MC1R	Deletion
MVD	Deletion
CHMP1A	Deletion
RPL13	Deletion
SPG7	Deletion
SLC7A5	Deletion
CDK10	Deletion
C16orf7	Deletion
FAM38A	Deletion
TUBB3	Deletion
PRDM7	Deletion
TCF25	Deletion
ZCCHC14	Deletion
CPNE7	Deletion
IL17C	Deletion
ANKRD11	Deletion
TRAPPC2L	Deletion
KLHDC4	Deletion
DEF8	Deletion
BANP	Deletion
JPH3	Deletion
DBNDD1	Deletion
FBXO31	Deletion
CDT1	Deletion
MAP1LC3B	Deletion
SPIRE2	Deletion
ZNF469	Deletion
CENPBD1	Deletion
ZNF276	Deletion
RNF166	Deletion
SPATA2L	Deletion
C16orf55	Deletion
ZC3H18	Deletion
ZFPM1	Deletion
MGC23284	Deletion
ZNF778	Deletion
ACSF3	Deletion
C16orf81	Deletion
SNAI3	Deletion
CTU2	Deletion
PABPN1L	Deletion
SNORD68	Deletion
LOC1001296	Deletion
LOC1001300	Deletion
hsa-mir-432	Deletion
hsa-mir-3191	Deletion

hsa-mir-319(	Deletion
hsa-mir-320e	Deletion
hsa-mir-220c	Deletion
hsa-mir-769	Deletion
hsa-mir-642	Deletion
hsa-mir-330	Deletion
BAX	Deletion
BCAT2	Deletion
C5AR1	Deletion
CA11	Deletion
CALM3	Deletion
CD37	Deletion
CGB	Deletion
CKM	Deletion
AP2S1	Deletion
CRX	Deletion
DBP	Deletion
DMPK	Deletion
DMWD	Deletion
EMP3	Deletion
ERCC1	Deletion
ERCC2	Deletion
FOSB	Deletion
FTL	Deletion
FUT1	Deletion
FUT2	Deletion
GIPR	Deletion
GPR4	Deletion
GRIN2D	Deletion
GRLF1	Deletion
GYS1	Deletion
FOXA3	Deletion
HRC	Deletion
KCNA7	Deletion
KCNJ14	Deletion
LHB	Deletion
LIG1	Deletion
NOVA2	Deletion
NPAS1	Deletion
NTF4	Deletion
NUCB1	Deletion
PPP5C	Deletion
PTGIR	Deletion
RPL18	Deletion
RTN2	Deletion
SEPW1	Deletion

SLC1A5	Deletion
SLC8A2	Deletion
SNRNP70	Deletion
SNRPD2	Deletion
SULT2B1	Deletion
SULT2A1	Deletion
TULP2	Deletion
VASP	Deletion
SYMPK	Deletion
TEAD2	Deletion
PPFIA3	Deletion
PLA2G4C	Deletion
NAPA	Deletion
PGLYRP1	Deletion
CYTH2	Deletion
DHX34	Deletion
SAE1	Deletion
PPP1R13L	Deletion
CD3EAP	Deletion
RUVBL2	Deletion
KDELR1	Deletion
KPTN	Deletion
CARD8	Deletion
ZC3H4	Deletion
FBXO46	Deletion
SYNGR4	Deletion
PPP1R15A	Deletion
EML2	Deletion
PRKD2	Deletion
CCDC9	Deletion
IRF2BP1	Deletion
FGF21	Deletion
BBC3	Deletion
DKKL1	Deletion
GPR77	Deletion
DHDH	Deletion
SLC6A16	Deletion
STRN4	Deletion
GLTSCR2	Deletion
GLTSCR1	Deletion
EHD2	Deletion
HSD17B14	Deletion
TRPM4	Deletion
QPCTL	Deletion
FAM83E	Deletion
RASIP1	Deletion

TMEM160	Deletion
C19orf73	Deletion
PNMAL1	Deletion
TMEM143	Deletion
CABP5	Deletion
SPHK2	Deletion
MEIS3	Deletion
PNMAL2	Deletion
PLEKHA4	Deletion
ELSPBP1	Deletion
LIN7B	Deletion
HIF3A	Deletion
FKRP	Deletion
OPA3	Deletion
RSPH6A	Deletion
GRWD1	Deletion
CCDC8	Deletion
ZNF541	Deletion
CCDC114	Deletion
DKFZp434J02	Deletion
CGB5	Deletion
CGB7	Deletion
CGB8	Deletion
GNG8	Deletion
PTH2	Deletion
CGB1	Deletion
CGB2	Deletion
LMTK3	Deletion
NTN5	Deletion
PPM1N	Deletion
KLC3	Deletion
CCDC155	Deletion
DACT3	Deletion
SIX5	Deletion
IGFL2	Deletion
ZNF114	Deletion
SPACA4	Deletion
PRR24	Deletion
TPRX1	Deletion
MAMSTR	Deletion
IZUMO1	Deletion
MYPOP	Deletion
NANOS2	Deletion
IGFL1	Deletion
IGFL3	Deletion
IGFL4	Deletion

SEC1	Deletion
SNORD23	Deletion
CCDC61	Deletion
SNAR-G1	Deletion
SNAR-A2|10(	Deletion
BSPH1	Deletion
SNAR-A3|10(	Deletion
SNAR-A13|1(	Deletion
SNAR-C2|10(	Deletion
SNAR-C4	Deletion
SNAR-E	Deletion
SNAR-C3	Deletion
SNAR-G2	Deletion
hsa-mir-571	Deletion
ATP5I	Deletion
DGKQ	Deletion
GAK	Deletion
IDUA	Deletion
MYL5	Deletion
PDE6B	Deletion
ZNF141	Deletion
PCGF3	Deletion
SPON2	Deletion
CPLX1	Deletion
SLC26A1	Deletion
FGFRL1	Deletion
PIGG	Deletion
ABCA11P	Deletion
MFSD7	Deletion
TMEM175	Deletion
ZNF595	Deletion
ZNF721	Deletion
ZNF718	Deletion
RNF212	Deletion
ZNF876P	Deletion
ZNF732	Deletion
LOC1001308	Deletion
hsa-mir-1302	Deletion
hsa-mir-1234	Deletion
hsa-mir-939	Deletion
hsa-mir-937	Deletion
hsa-mir-661	Deletion
hsa-mir-151	Deletion
hsa-mir-30b	Deletion
hsa-mir-30d	Deletion
BAI1	Deletion

CYC1	Deletion
CYP11B1	Deletion
CYP11B2	Deletion
EEF1D	Deletion
GLI4	Deletion
GML	Deletion
GPR20	Deletion
GPT	Deletion
GRINA	Deletion
HSF1	Deletion
KCNQ3	Deletion
LY6E	Deletion
LY6H	Deletion
NFKBIL2	Deletion
PLEC	Deletion
PTK2	Deletion
RPL8	Deletion
ST3GAL1	Deletion
SLA	Deletion
TG	Deletion
TSTA3	Deletion
ZNF7	Deletion
ZNF16	Deletion
PSCA	Deletion
LY6D	Deletion
JRK	Deletion
DGAT1	Deletion
GPAA1	Deletion
WISP1	Deletion
FOXH1	Deletion
RECQL4	Deletion
LRRC14	Deletion
ZNF623	Deletion
HHLA1	Deletion
NDRG1	Deletion
KHDRBS3	Deletion
PTP4A3	Deletion
PUF60	Deletion
DENND3	Deletion
ZC3H3	Deletion
EFR3A	Deletion
ARC	Deletion
BOP1	Deletion
SCRIB	Deletion
LRRC6	Deletion
FBXL6	Deletion

OPLAH	Deletion
EIF2C2	Deletion
COMMD5	Deletion
CPSF1	Deletion
CYHR1	Deletion
FAM135B	Deletion
PHF20L1	Deletion
VPS28	Deletion
C8orf30A|51	Deletion
KCNK9	Deletion
C8orf55	Deletion
CHRAC1	Deletion
EXOSC4	Deletion
LY6K	Deletion
SLC39A4	Deletion
SLURP1	Deletion
SLC45A4	Deletion
ZFAT	Deletion
ZNF250	Deletion
PYCRL	Deletion
C8orf33	Deletion
LYNX1	Deletion
C8orf51	Deletion
GPR172A	Deletion
GSDMD	Deletion
ZNF696	Deletion
ARHGAP39	Deletion
ZNF34	Deletion
SHARPIN	Deletion
EPPK1	Deletion
SCRT1	Deletion
TRAPPC9	Deletion
MAF1	Deletion
PARP10	Deletion
TIGD5	Deletion
PPP1R16A	Deletion
ZNF251	Deletion
KIFC2	Deletion
NAPRT1	Deletion
HPYR1	Deletion
MFSD3	Deletion
RHPN1	Deletion
TOP1MT	Deletion
LYPD2	Deletion
TMEM71	Deletion
COL22A1	Deletion

ADCK5	Deletion
TSNARE1	Deletion
MAPK15	Deletion
ZNF707	Deletion
BREA2	Deletion
FAM83H	Deletion
LOC286094	Deletion
ZNF252	Deletion
TMED10P1	Deletion
C8orf77	Deletion
C8orf31	Deletion
ZFP41	Deletion
GPIHBP1	Deletion
NRBP2	Deletion
ZNF517	Deletion
KIAA1875	Deletion
C8ORFK29	Deletion
SPATC1	Deletion
FLJ43860	Deletion
MAFA	Deletion
MGC70857	Deletion
LRRC24	Deletion
ZFATAS	Deletion
NCRNA0005[illegible]	Deletion
C8orf73	Deletion
SCXB|64265[illegible]	Deletion
HEATR7A	Deletion
OC90	Deletion
LOC731779	Deletion
LOC1001302[illegible]	Deletion
LOC1001336[illegible]	Deletion
FGF2	Methylation
STK33	Methylation
FAM89A	Methylation
SOX7	Methylation
RUNX3	Methylation
B3GNT5	Methylation
DSC3	Methylation
PKDREJ	Methylation
DZIP1	Methylation
EOMES	Methylation
CCND2	Methylation
PRKCQ	Methylation
LEPREL1	Methylation
KLK10	Methylation
SLC34A2	Methylation

ST8SIA1	Methylation
EIF5A2	Methylation
CXCL1	Methylation
CXCL5	Methylation
GSTP1	Methylation
VIPR2	Methylation
KCNK17	Methylation
ID4	Methylation
FBLN2	Methylation
ASCL2	Methylation
CCDC3	Methylation
OSR1	Methylation
IGF2AS	Methylation
COL23A1	Methylation
GPX7	Methylation
MAL	Methylation
FUT9	Methylation
RCSD1	Methylation
KIF17	Methylation
PAK7	Methylation
DCC	Methylation
PPP1R14C	Methylation
STAC2	Methylation
GALNT12	Methylation
HAAO	Methylation
SPATA18	Methylation
ZNF132	Methylation
EFCAB1	Methylation
CLDN11	Methylation
PKIA	Methylation
DGKI	Methylation
CLIP4	Methylation
VCAN	Methylation
BCL11A	Methylation
ADAMTS5	Methylation
PTGDR	Methylation
LTF	Methylation
PDPN	Methylation
EDN3	Methylation
RECK	Methylation
ADAM12	Methylation
SFRP1	Methylation
CYP7B1	Methylation
PDGFRA	Methylation
PTH2R	Methylation
JAM2	Methylation

CHST3	Methylation
NTRK3	Methylation
ACTA1	Methylation
CPXM1	Methylation
ENTPD3	Methylation
OSBPL3	Methylation
WIF1	Methylation
FZD10	Methylation
TFPI2	Methylation
PBX4	Methylation
TCF7	Methylation
CD8B	Methylation
SPG20	Methylation
FGF10	Methylation
RRAD	Methylation
FNDC4	Methylation
COL5A1	Methylation
KCNJ2	Methylation
NEFH	Methylation
FLRT2	Methylation
D4S234E	Methylation
WNT2	Methylation
KCNK13	Methylation
PRNP	Methylation
CHRNA3	Methylation
GAS7	Methylation
C10orf107	Methylation
SFRP2	Methylation
SLIT2	Methylation
PRKCDBP	Methylation
DKK1	Methylation
FADS2	Methylation
TNFRSF10C	Methylation
NPY5R	Methylation
RARB	Methylation
PTPRM	Methylation
COL6A2	Methylation
ZNF542	Methylation
MDFI	Methylation
TSPYL5	Methylation
HAND2	Methylation
CDO1	Methylation
COL1A2	Methylation
RIC3	Methylation
HOXA5	Methylation
CD40	Methylation

PAM	Methylation
COL9A3	Methylation
DYRK3	Methylation
RASSF2	Methylation
WNT10A	Methylation
NPFFR2	Methylation
CDC14B	Methylation
HOXA9	Methylation
GNA14	Methylation
CCDC65	Methylation
VSNL1	Methylation
DIO2	Methylation
CHL1	Methylation
MRPS21	Methylation
SYT6	Methylation
CXCL12	Methylation
TOX2	Methylation
CCNJL	Methylation
CLDN1	Methylation
CHRNB1	Methylation
MGC42105	Methylation
TFPI	Methylation
FST	Methylation
LIMS2	Methylation
SNAI2	Methylation
ACN9	Methylation
CHI3L2	Methylation
SPON1	Methylation
CDH11	Methylation
PLS3	Methylation
FES	Methylation
GNG11	Methylation
GRP	Methylation
MET	Methylation
EPHB1	Methylation
NEFM	Methylation
DNM3	Methylation
IRAK3	Methylation
ALDH1A3	Methylation
ATP10A	Methylation
ELMO1	Methylation
PELI2	Methylation
RAB38	Methylation
RYR1	Methylation
ZMYND12	Methylation
DOK5	Methylation

GLT8D2	Methylation
KDELC1	Methylation
C6orf145	Methylation
CBR1	Methylation
AGTR1	Methylation
COL14A1	Methylation
PON3	Methylation
FSTL1	Methylation
AQP3	Methylation
NTRK2	Methylation
SESN3	Methylation
STAT5A	Methylation
KLK7	Methylation
CHRDL2	Methylation
AK5	Methylation
B3GNT3	Methylation
LIPG	Methylation
TGFBR2	Methylation
MAMDC2	Methylation
SLFN12	Methylation
HYDIN	Methylation
GOLM1	Methylation
RAB31	Methylation
MGAT3	Methylation
ZNF502	Methylation
CITED4	Methylation
SCGB3A1	Methylation
FZD9	Methylation
EGFR	Methylation
PID1	Methylation
PCSK5	Methylation
CDH22	Methylation
ALKBH3	Methylation
SLC5A1	Methylation
TINAGL1	Methylation
TRIM17	Methylation
SCN4B	Methylation
CST6	Methylation
EFHA2	Methylation
GDF10	Methylation
DSE	Methylation
PPP1R14A	Methylation
WASF3	Methylation
ZEB2	Methylation
NRN1	Methylation
CNN1	Methylation

GRIA3	Methylation
TLL1	Methylation
AQP5	Methylation
FBLN5	Methylation
VWA1	Methylation
LAMC1	Methylation
PTGIS	Methylation
TRPV4	Methylation
BMP8A	Methylation
TNFRSF11B	Methylation
TEKT3	Methylation
BTG3	Methylation
FARP1	Methylation
LGALS3	Methylation
PRKD1	Methylation
KLHL34	Methylation
FAM46B	Methylation
RPS6KA2	Methylation
C11orf70	Methylation
FABP3	Methylation
TBX5	Methylation
SPATA6	Methylation
SPARC	Methylation
COTL1	Methylation
GPM6B	Methylation
TMEM71	Methylation
TMEM35	Methylation
NDP	Methylation
LOXL4	Methylation
SERPINE2	Methylation
ITM2A	Methylation
SOSTDC1	Methylation
APOBEC3C	Methylation
RARRES1	Methylation
FOXF2	Methylation
MFSD7	Methylation
MYL9	Methylation
CCDC8	Methylation
CHODL	Methylation
RAC2	Methylation
PLEKHF1	Methylation
ZNF238	Methylation
RSPO1	Methylation
TMEM130	Methylation
IL32	Methylation
SCARA3	Methylation

ARHGAP22	Methylation
FBLIM1	Methylation
KCNK5	Methylation
IGF1	Methylation
STMN2	Methylation
CACNA1C	Methylation
KRT7	Methylation
WDR63	Methylation
COL1A1	Methylation
PPP1R1B	Methylation
CLEC2B	Methylation
NEK6	Methylation
TGFBR3	Methylation
CYB5R2	Methylation
IFNGR2	Methylation
TTC23	Methylation
C3	Methylation
GALM	Methylation
ASS1	Methylation
SGCD	Methylation
MOBKL2B	Methylation
LAMB1	Methylation
SYT11	Methylation
SMO	Methylation
MMP7	Methylation
HGF	Methylation
KCNA2	Methylation
CHST6	Methylation
PLAGL1	Methylation
KCNN4	Methylation
FAM124B	Methylation
HOXA4	Methylation
SLC40A1	Methylation
FAM49A	Methylation
PCDH7	Methylation
GYPC	Methylation
CARD9	Methylation
FAM113B	Methylation
LY75	Methylation
CX3CL1	Methylation
RASL10A	Methylation
POMC	Methylation
ART5	Methylation
DPYSL3	Methylation
SCN2B	Methylation
NFYC	Methylation

NLGN4X	Methylation
TRO	Methylation
SLC46A2	Methylation
RGS14	Methylation
S100A2	Methylation
ZNF532	Methylation
FMOD	Methylation
NDN	Methylation
DHRS12	Methylation
MPP6	Methylation
KCTD12	Methylation
MAP3K14	Methylation
GLRX	Methylation
PRSS27	Methylation
PSTPIP1	Methylation
JPH2	Methylation
TPST1	Methylation
PARP8	Methylation
SCPEP1	Methylation
NINJ2	Methylation
PHACTR2	Methylation
DISC1	Methylation
SCN5A	Methylation
NXN	Methylation
FOXO1	Methylation
LCN2	Methylation
SCNN1A	Methylation
PLXDC2	Methylation
LHFP	Methylation
LAMB3	Methylation
KLHL5	Methylation
PPM1M	Methylation
PHYHIP	Methylation
SERPING1	Methylation
CRIM1	Methylation
TMEM92	Methylation
C2orf39	Methylation
TNFSF12	Methylation
PLSCR4	Methylation
CALD1	Methylation
CHI3L1	Methylation
LPXN	Methylation
DIRAS3	Methylation
SLC25A20	Methylation
EFEMP1	Methylation
ACVR1	Methylation

C2orf40	Methylation
KLK5	Methylation
CHST8	Methylation
OXGR1	Methylation
ALDH2	Methylation
H2AFY2	Methylation
CST7	Methylation
RUNX1T1	Methylation
C13orf29	Methylation
KRT15	Methylation
TPM2	Methylation
HCLS1	Methylation
DYNC1I1	Methylation
FCGBP	Methylation
NAP1L3	Methylation
RUNX1	Methylation
COL18A1	Methylation
YPEL4	Methylation
BANK1	Methylation
HLA-DPB1	Methylation
TIMP1	Methylation
RAB33A	Methylation
CSDA	Methylation
GGN	Methylation
RSPO3	Methylation
TPM1	Methylation
AIF1	Methylation
HTRA1	Methylation
UNC13D	Methylation
GALNT5	Methylation
RTN1	Methylation
KRT6A	Methylation
EFEMP2	Methylation
BNC2	Methylation
SPNS3	Methylation
DMD	Methylation
GSPT2	Methylation
TRIM29	Methylation
SSPN	Methylation
STAT4	Methylation
CNN3	Methylation
A2M	Methylation
GAB3	Methylation
PDLIM4	Methylation
TES	Methylation
MSRB3	Methylation

SKAP2	Methylation
CCL23	Methylation
THBS3	Methylation
RASIP1	Methylation
PAQR8	Methylation
HSD17B2	Methylation
ARL11	Methylation
ARHGAP9	Methylation
SPARCL1	Methylation
C8orf79	Methylation
CLIC6	Methylation
DLL1	Methylation
MFAP4	Methylation
GJB5	Methylation
GMFG	Methylation
RIMS3	Methylation
RGN	Methylation
IL1RAP	Methylation
S100A4	Methylation
MIA	Methylation
VNN2	Methylation
FAP	Methylation
MRGPRF	Methylation
PLD1	Methylation
CCDC89	Methylation
PIK3CD	Methylation
SLC46A3	Methylation
GJA1	Methylation
WIPF1	Methylation
RAB3IL1	Methylation
LCN12	Methylation
CD37	Methylation
MAGEE2	Methylation
STK24	Methylation
OXT	Methylation
DDR2	Methylation
PNMA3	Methylation
MAML2	Methylation
C1S	Methylation
CUL4B	Methylation
PLEKHG5	Methylation
HOXA2	Methylation
ICAM2	Methylation
PPAPDC3	Methylation
PLAC8	Methylation
KCTD1	Methylation

CALML3	Methylation
TAGLN	Methylation
GPR62	Methylation
CD6	Methylation
KCNE1L	Methylation
KRT17	Methylation
C20orf85	Methylation
UBE2E3	Methylation
DOCK8	Methylation
TP63	Methylation
FOLR1	Methylation
OLFML2B	Methylation
ZNF185	Methylation
ACADVL	Methylation
S100A1	Methylation
TNC	Methylation
SCMH1	Methylation
TNFAIP8L2	Methylation
SOD3	Methylation
RBP5	Methylation
RUNX2	Methylation
ANPEP	Methylation
COL17A1	Methylation
C13orf16	Methylation
C5orf32	Methylation
ARHGEF4	Methylation
GLI1	Methylation
UBLCP1	Methylation
CCDC88B	Methylation
PPP2R2B	Methylation
IL2RB	Methylation
APOC2	Methylation
CLIC5	Methylation
PTGDS	Methylation
WNT5B	Methylation
CCL22	Methylation
LPP	Methylation
KRT16	Methylation
SYT8	Methylation
RELB	Methylation
MTMR2	Methylation
STARD13	Methylation
CTSK	Methylation
TLE4	Methylation
EVC2	Methylation
FAM131B	Methylation

FUT7	Methylation
EMB	Methylation
CLDN19	Methylation
KLK8	Methylation
PHYHD1	Methylation
ACTG2	Methylation
HSPB2	Methylation
PIK3R1	Methylation
EFHA1	Methylation
IL17B	Methylation
RAB20	Methylation
COG6	Methylation
ENTHD1	Methylation
MED4	Methylation
NARG1L	Methylation
