# Supplementary material for: Genome-driven integrated classification of breast cancer validated in over 7,500 samples
Source: Genome Biol. 2014 Aug 28;15(8):431. doi: 10.1186/s13059-014-0431-1 (PMC4166472; doi:10.1186/s13059-014-0431-1)

Additional file 13 – Difference in adjusted R-squared between IntClust and SCMGENE classifiers by study

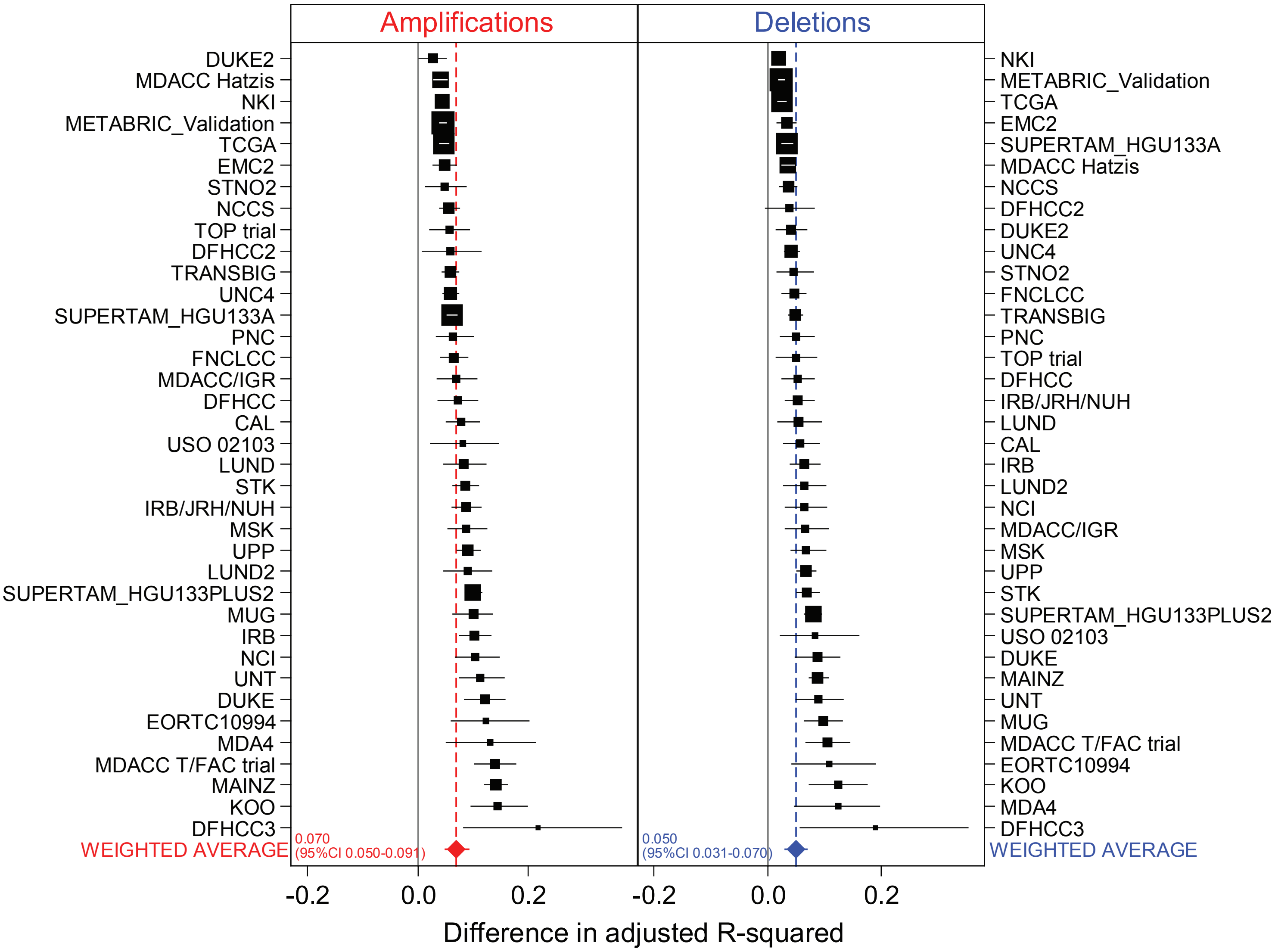

Supplement: Additional file 13: — Difference in adjusted R-squared between IntClust and SCMGENE classifiers by study. [file 13059_2014_431_MOESM13_ESM.pdf]
