# Supplementary material for: Genome-driven integrated classification of breast cancer validated in over 7,500 samples
Source: Genome Biol. 2014 Aug 28;15(8):431. doi: 10.1186/s13059-014-0431-1 (PMC4166472; doi:10.1186/s13059-014-0431-1)

Additional file 14 – Ranked list of the top fifty genes with R-squared values explained by IntClust (top panel) and those better explained by IntClust compared to PAM50 (bottom panel).

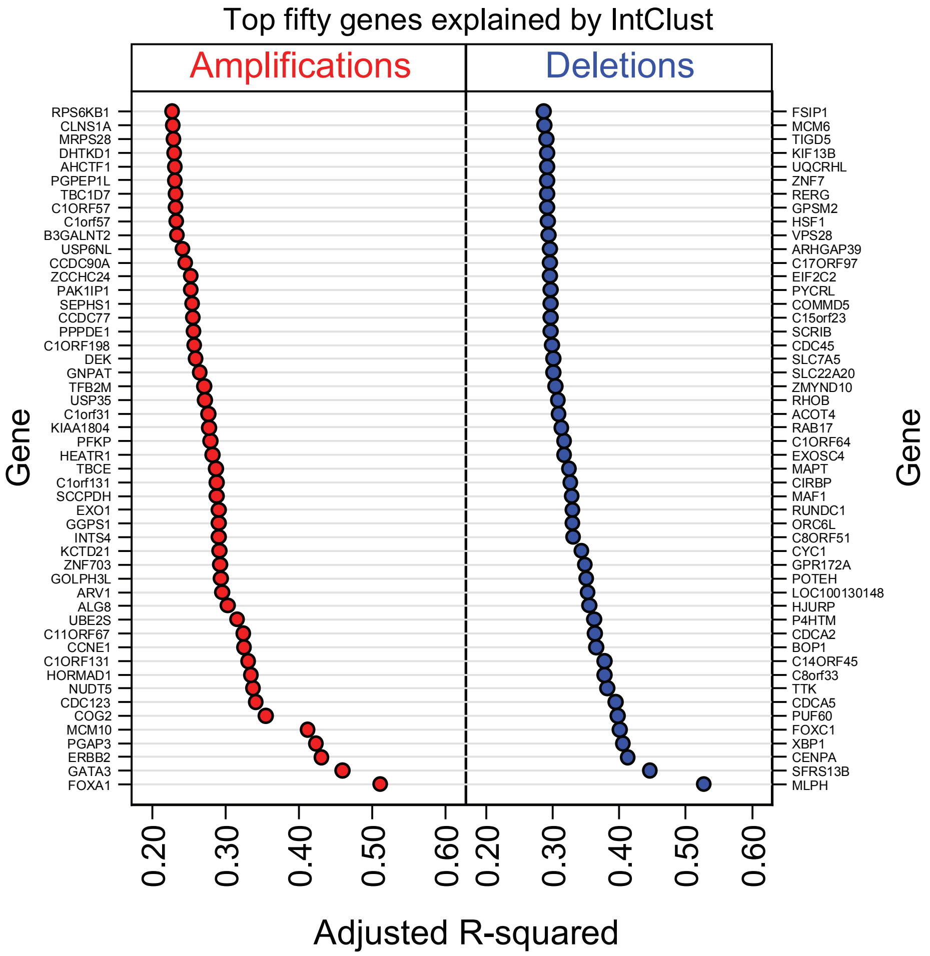

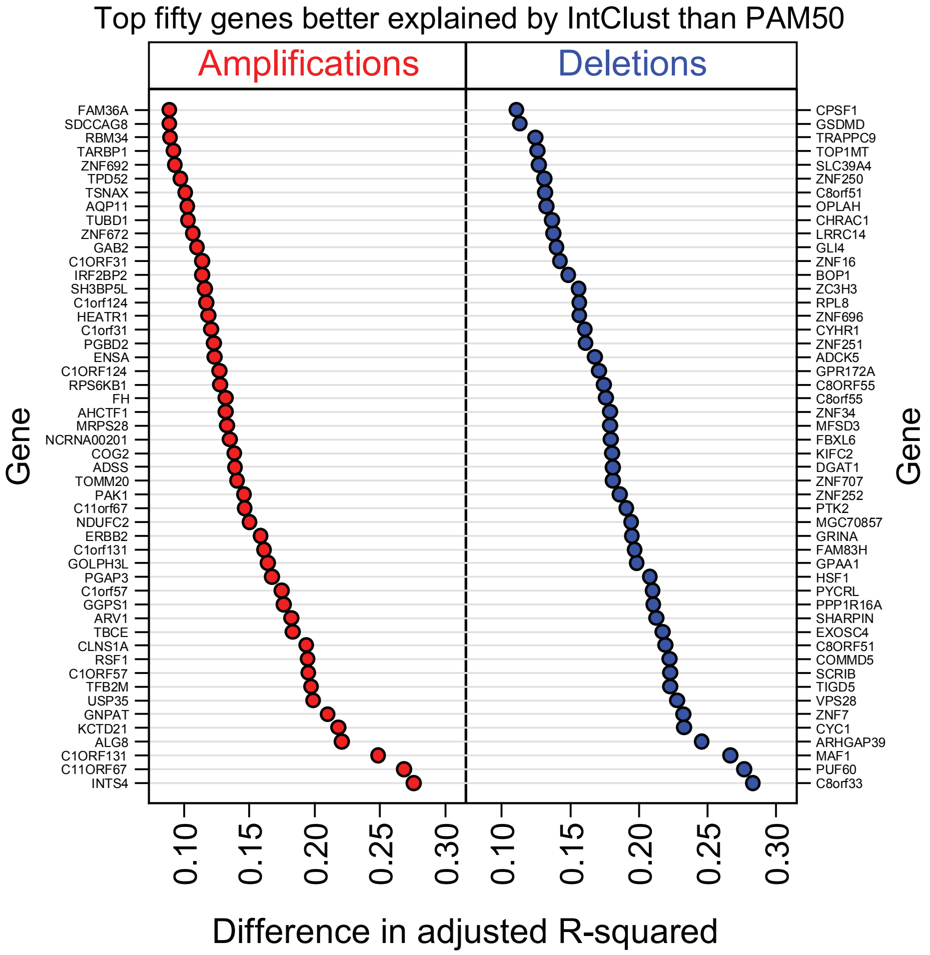

Supplement: Additional file 14: — Ranked list of the top 50 genes with R-squared values explained by IntClust (top panel) and those better explained by IntClust compared to PAM50 (bottom panel). R-squared values are based on analysis-of-variance (ANOVA) models using molecular subtype and levels of gene expression. [file 13059_2014_431_MOESM14_ESM.pdf]
