# Supplementary material for: Genome-driven integrated classification of breast cancer validated in over 7,500 samples
Source: Genome Biol. 2014 Aug 28;15(8):431. doi: 10.1186/s13059-014-0431-1 (PMC4166472; doi:10.1186/s13059-014-0431-1)
Supplement: Additional file 16: — Scatter plots depicting adjusted R-squared statistics for ANOVA models for all amplified and deleted genes by study. [file 13059_2014_431_MOESM16_ESM.pdf]

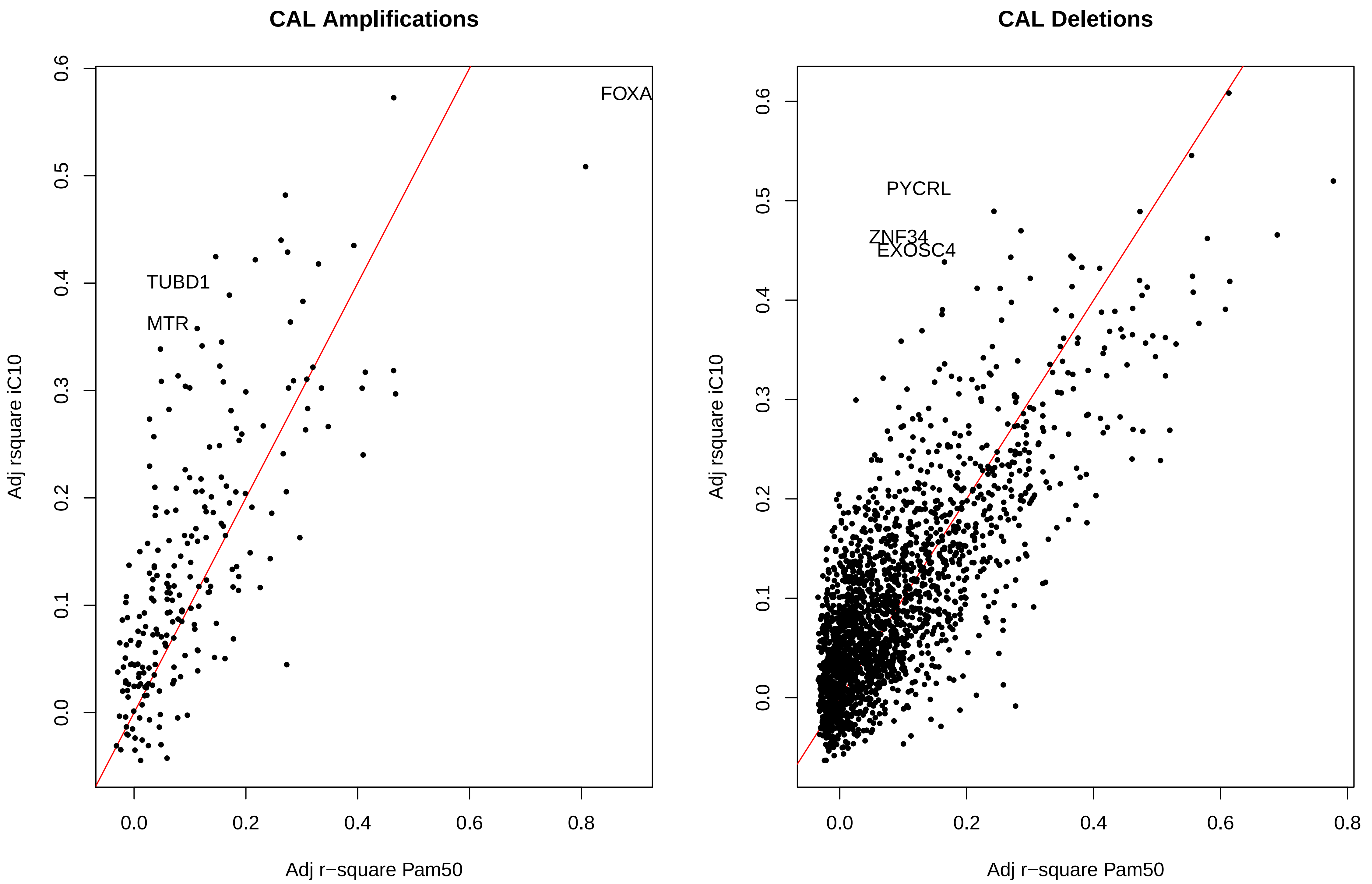

CAL Amplifications
FOXA
TUBD1
MTR
Adj rsquare iC10
Adj r−square Pam50
0.0
0.1
0.2
0.3
0.4
0.5
0.6
0.8
CAL Deletions
PYCRL
ZNF34
EXOSC4

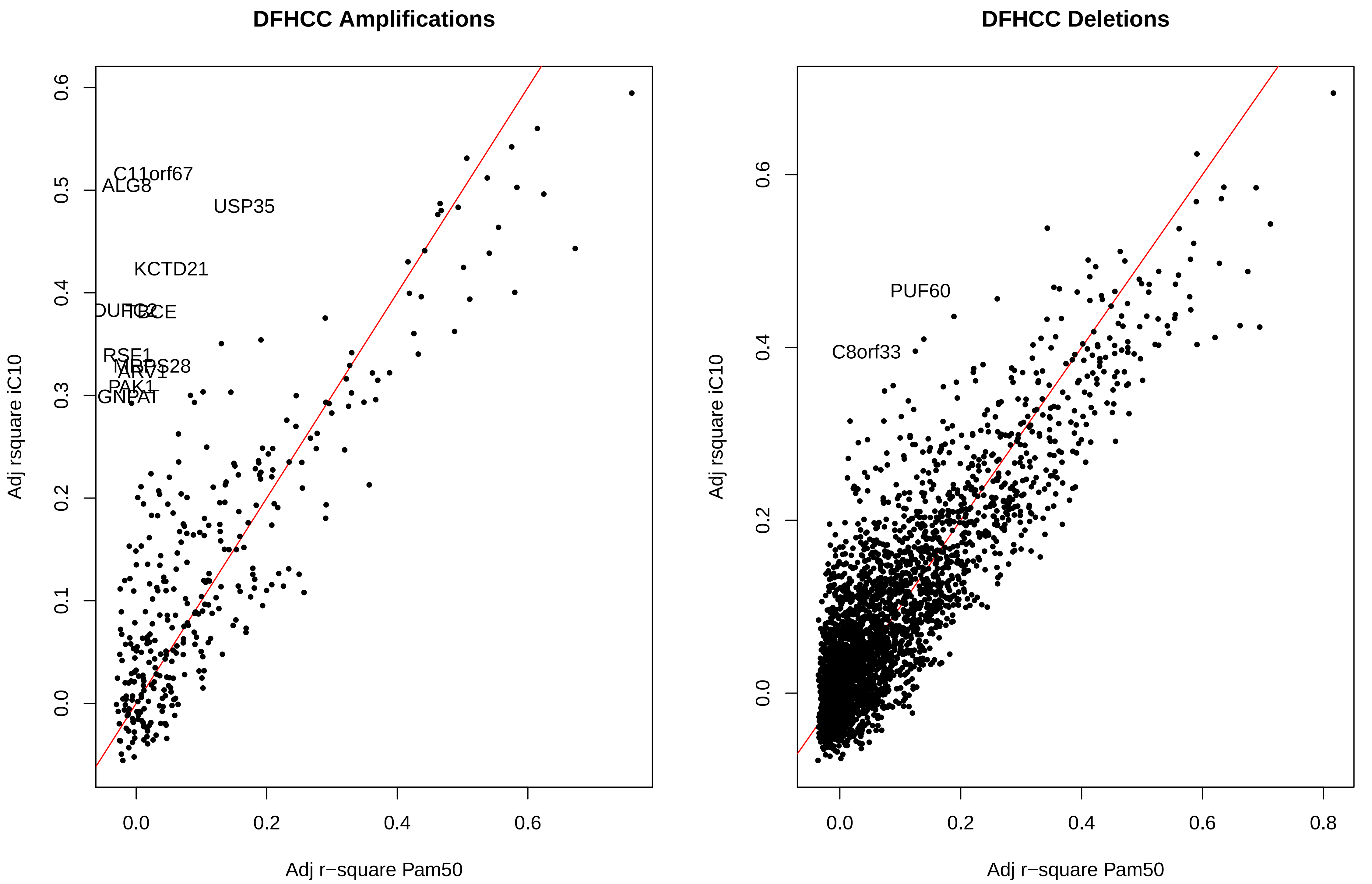
DFHCC Amplifications
Adj rsquare iC10
Adj r−square Pam50
C11orf67
ALG8
USP35
KCTD21
TBCE
RSF1
MRPS28
ARV1
PAK1
GNPAT
DFHCC Deletions
Adj rsquare iC10
Adj r−square Pam50
PUF60
C8orf33

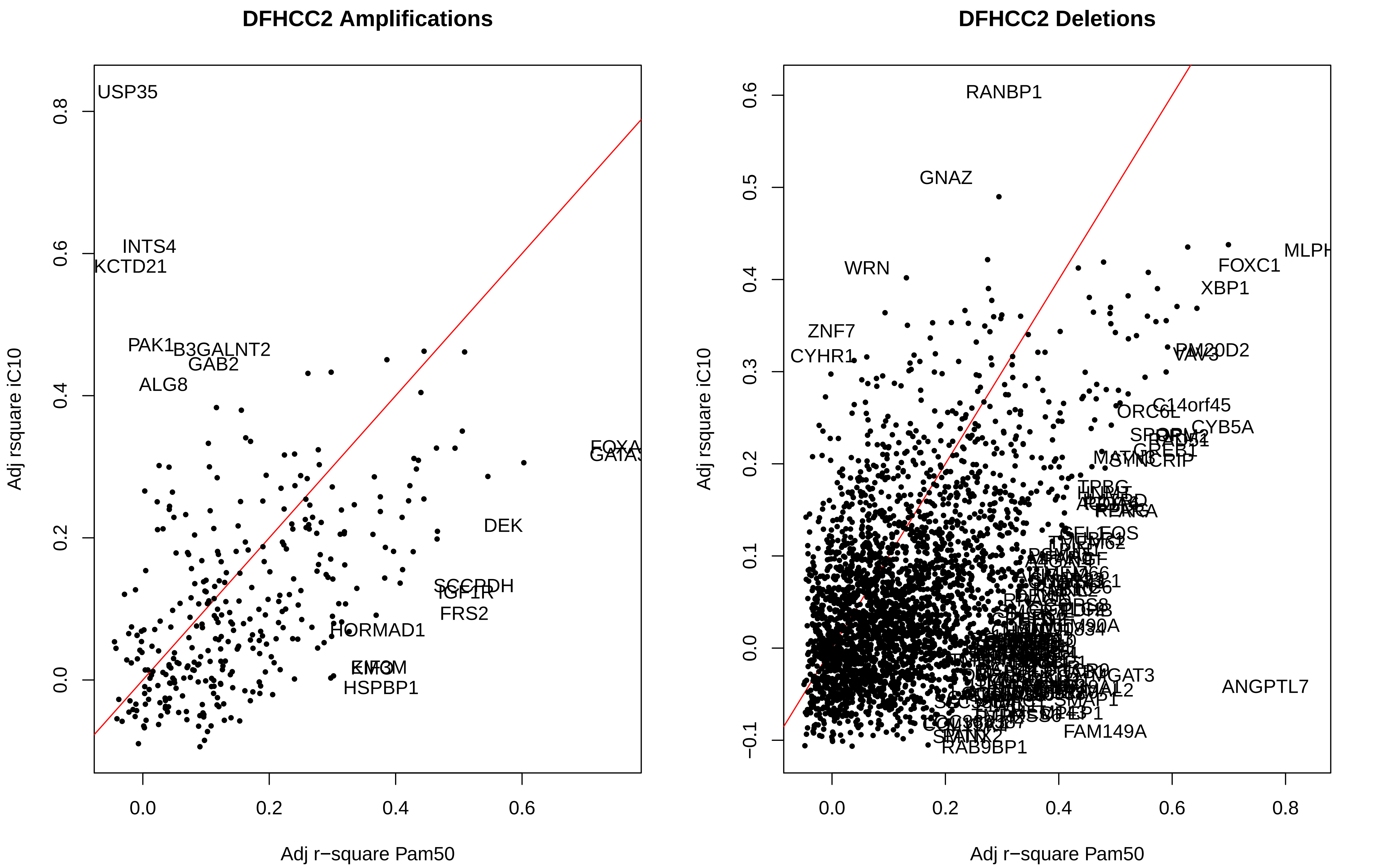

DFHCC2 Amplifications
USP35
INTS4
KCTD21
PAK1
B3GALNT2
GAB2
ALG8
DEK
SCCPDH
IGF1R
FRS2
HORMAD1
HSPBP1
Adj rsquare iC10
Adj r−square Pam50
DFHCC2 Deletions
RANBP1
GNAZ
WRN
ZNF7
CYHR1
MLPH
FOXC1
XBP1
C14orf45
ORC6L
CYB5A
ANGPTL7
FAM149A
RAB9BP1
Adj rsquare iC10
Adj r−square Pam50

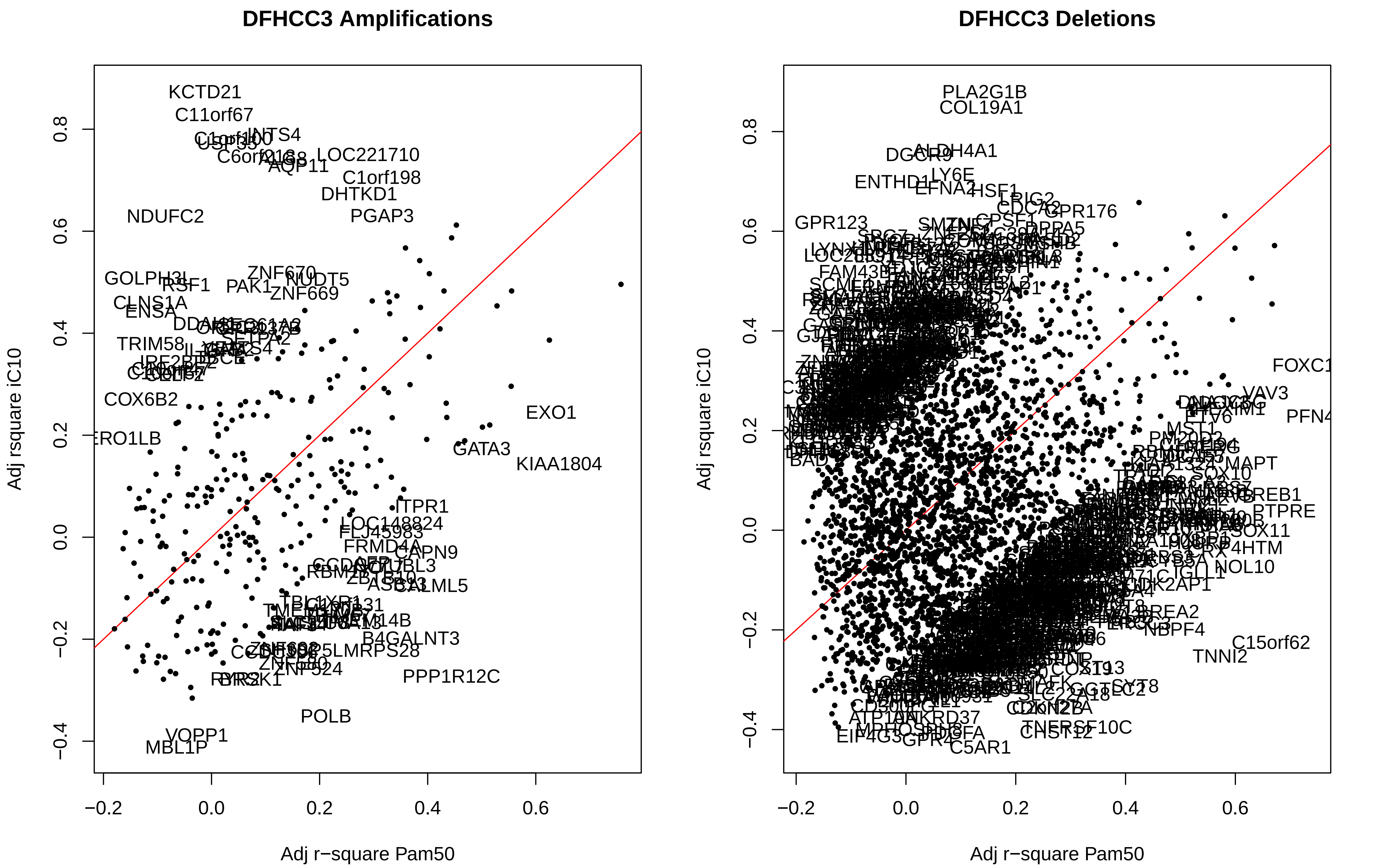

DFHCC3 Amplifications
DFHCC3 Deletions
Adj rsquare iC10
Adj r−square Pam50
−0.2
0.0
0.2
0.4
0.6
0.8
−0.4
KCTD21
C11orf67
INTS4
LOC221710
AQP11
C1orf198
DHTKD1
NDUFC2
PGAP3
GOLPH3L
RSF1
PAK1
ZNF670
NUDT5
ZNF669
CLNS1A
ENSA
TRIM58
COX6B2
EXO1
GATA3
KIAA1804
ITPR1
LOC148824
FLJ45983
FRMD4A
CAPN9
CALML5
B4GALNT3
PPP1R12C
POLB
VOPP1
MBL1P
PLA2G1B
COL19A1
ALDH4A1
ENTHD1
LY6E
EFNA2
HSF1
GPR176
GPR123
FOXC1
VAV3
ETV6
MST1
MAPT
SOX10
CREB1
PTPRE
SOX11
NOL10
CDK2AP1
NBPF4
C15orf62
TNNI2
SYT8
ANKRD37
CHST12
C5AR1

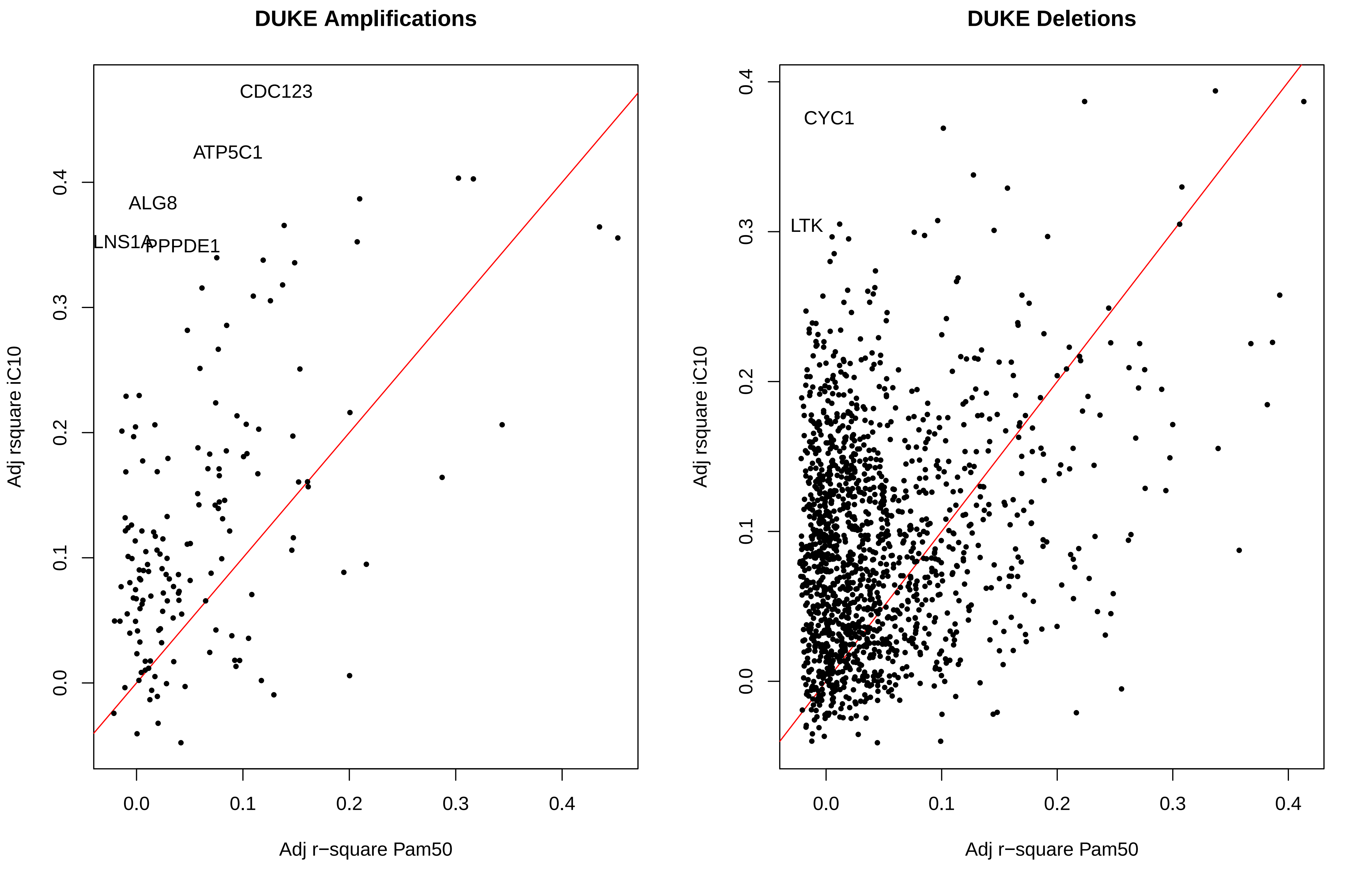
DUKE Amplifications
CDC123
ATP5C1
ALG8
LNS1A
PPPDE1
Adj rsquare iC10
Adj r−square Pam50
0.0
0.1
0.2
0.3
0.4
DUKE Deletions
CYC1
LTK
Adj rsquare iC10
Adj r−square Pam50

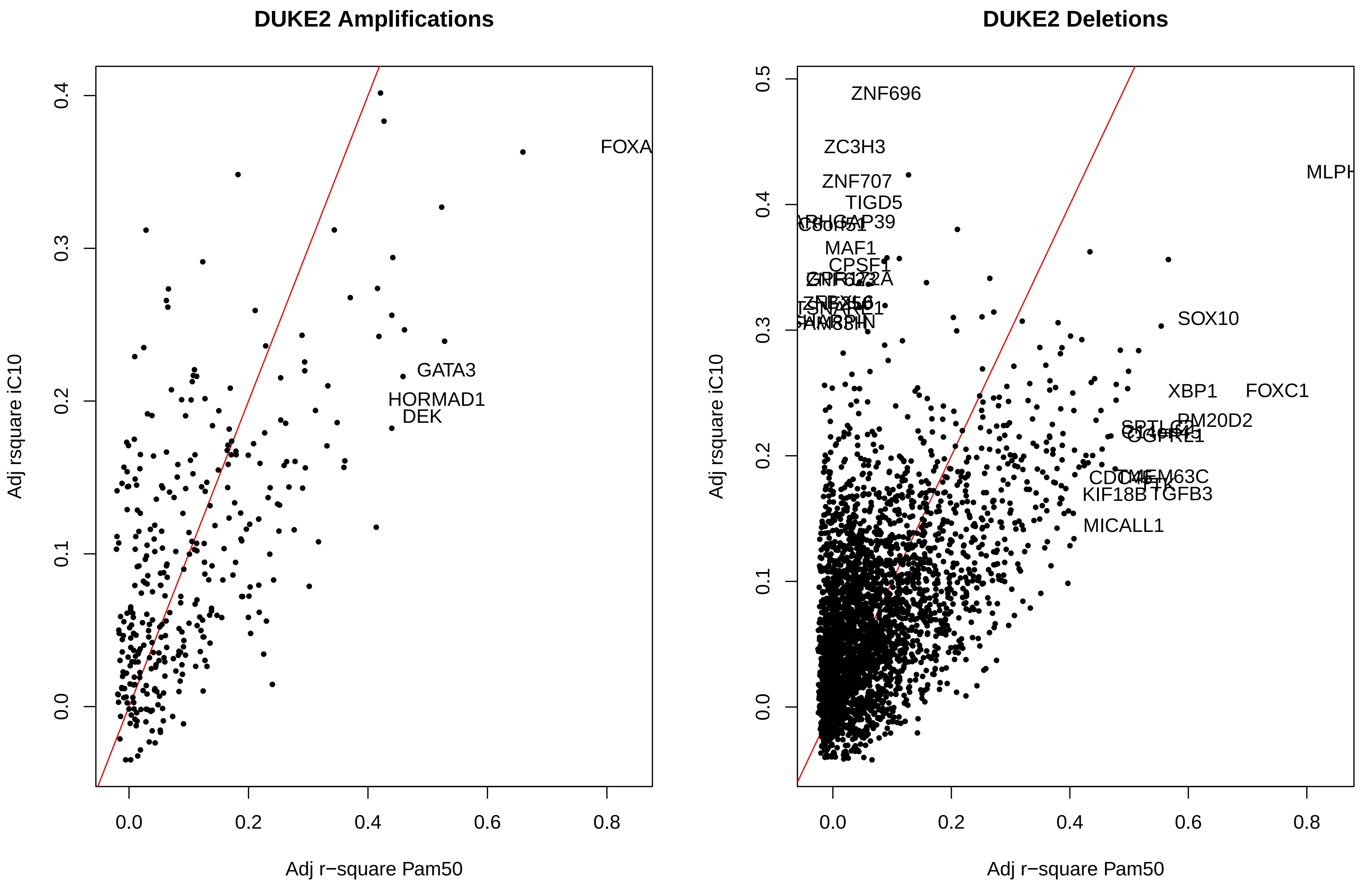

DUKE2 Amplifications
DUKE2 Deletions
Adj rsquare iC10
Adj r−square Pam50
0.0
0.1
0.2
0.3
0.4
0.5
0.6
0.8
FOXA
GATA3
HORMAD1
DEK
ZNF696
ZC3H3
ZNF707
TIGD5
MAF1
CPSF1
MLPH
SOX10
XBP1
FOXC1
PM20D2
OGFRL1
CDC45
TMEM63C
KIF18B
TGFB3
MICALL1

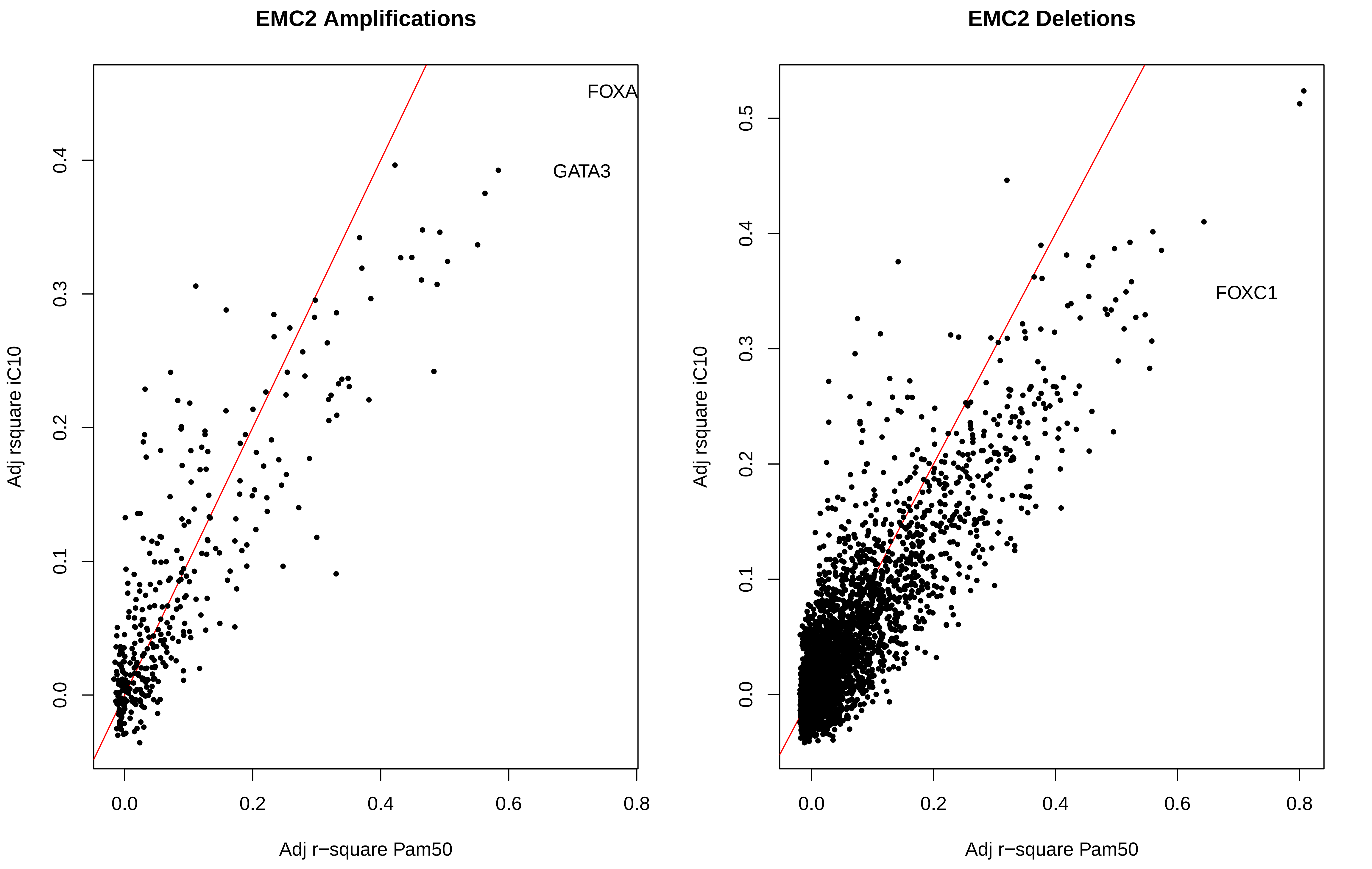
EMC2 Amplifications
FOXA
GATA3
Adj rsquare iC10
Adj r−square Pam50
0.0
0.1
0.2
0.3
0.4
0.6
0.8
EMC2 Deletions
FOXC1
0.5
Adj rsquare iC10
Adj r−square Pam50

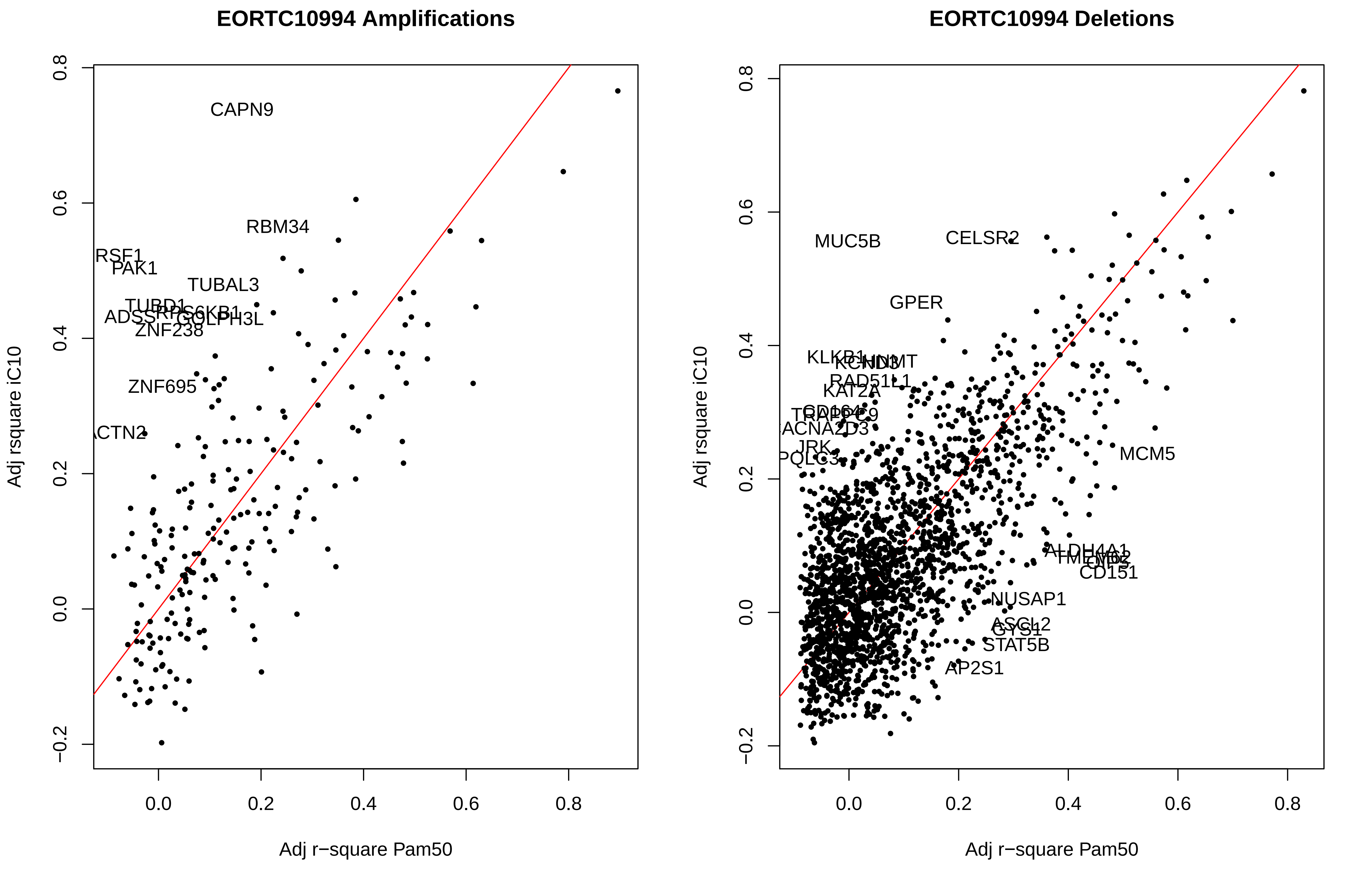
EORTC10994 Amplifications
Adj rsquare iC10
Adj r−square Pam50
CAPN9
RBM34
PAK1
TUBAL3
ZNF238
ZNF695
EORTC10994 Deletions
Adj rsquare iC10
Adj r−square Pam50
MUC5B
CELSR2
GPER
RAD51L1
KAT2A
MCM5
CD151
NUSAP1
STAT5B
AP2S1

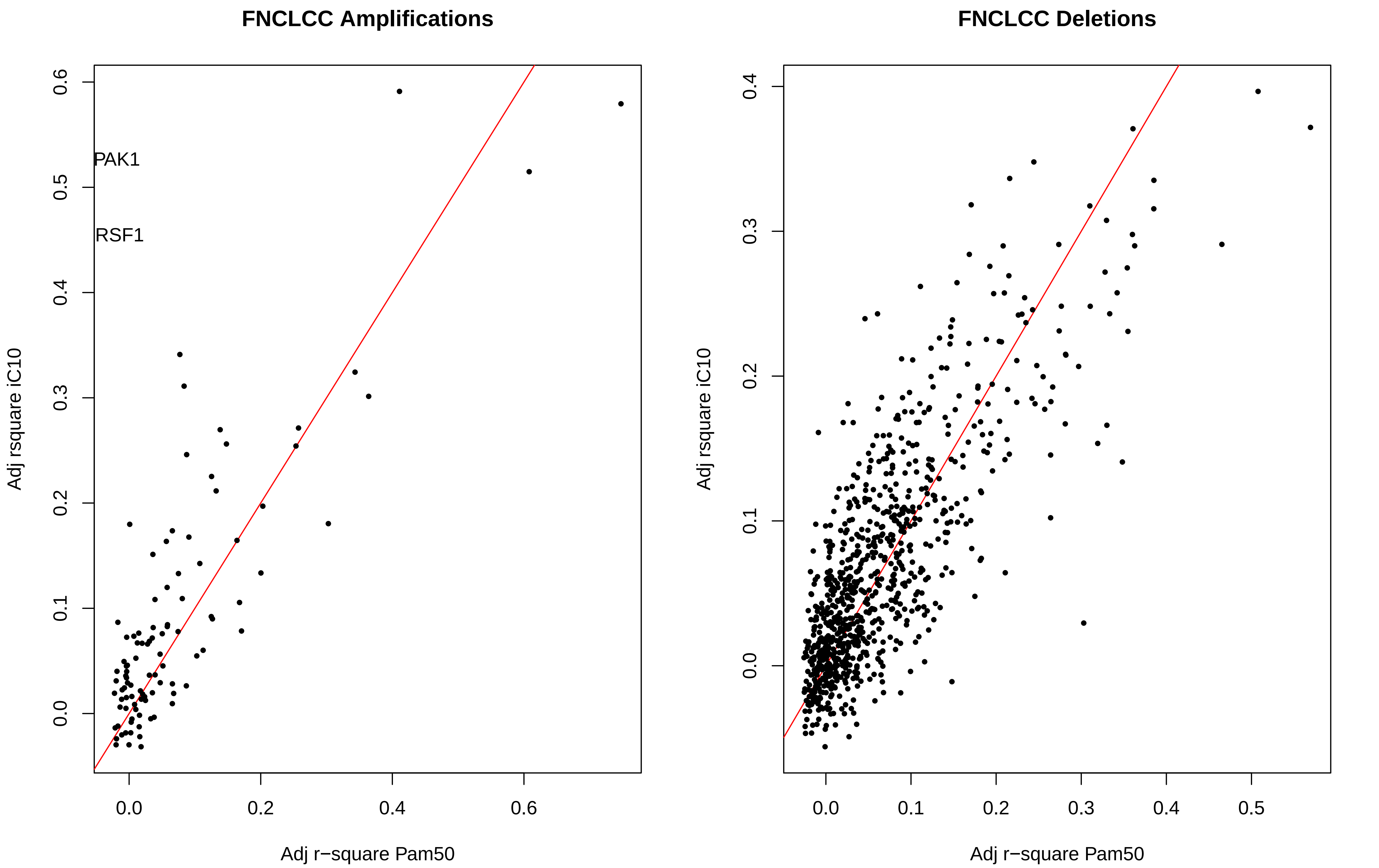
FNCLCC Amplifications
PAK1
RSF1
Adj rsquare iC10
Adj r−square Pam50
0.0
0.1
0.2
0.3
0.4
0.5
0.6
FNCLCC Deletions
Adj rsquare iC10
Adj r−square Pam50
0.0
0.1
0.2
0.3
0.4
0.5

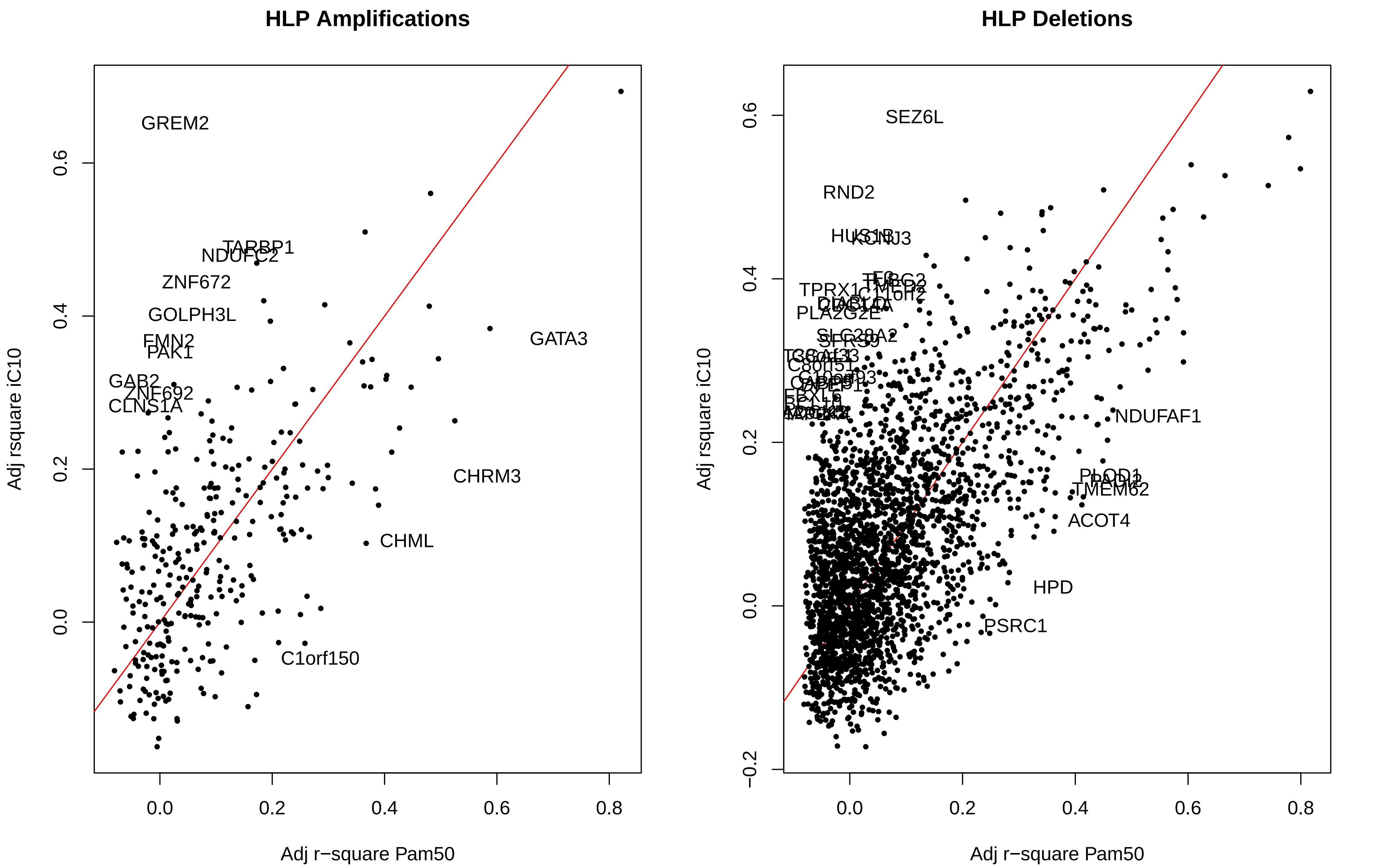
HLP Amplifications
GREM2
TARBP1
NDUFC2
ZNF672
GOLPH3L
FMN2
PAK1
GAB2
ZNF692
CLNS1A
GATA3
CHRM3
CHML
C1orf150
Adj rsquare iC10
Adj r−square Pam50
HLP Deletions
SEZ6L
RND2
KCNJ3
TPRX1
PLA2G2E
SLC28A2
SFRS9
C8orf51
NDUFAF1
PLOD1
TMEM62
ACOT4
HPD
PSRC1
Adj rsquare iC10
Adj r−square Pam50

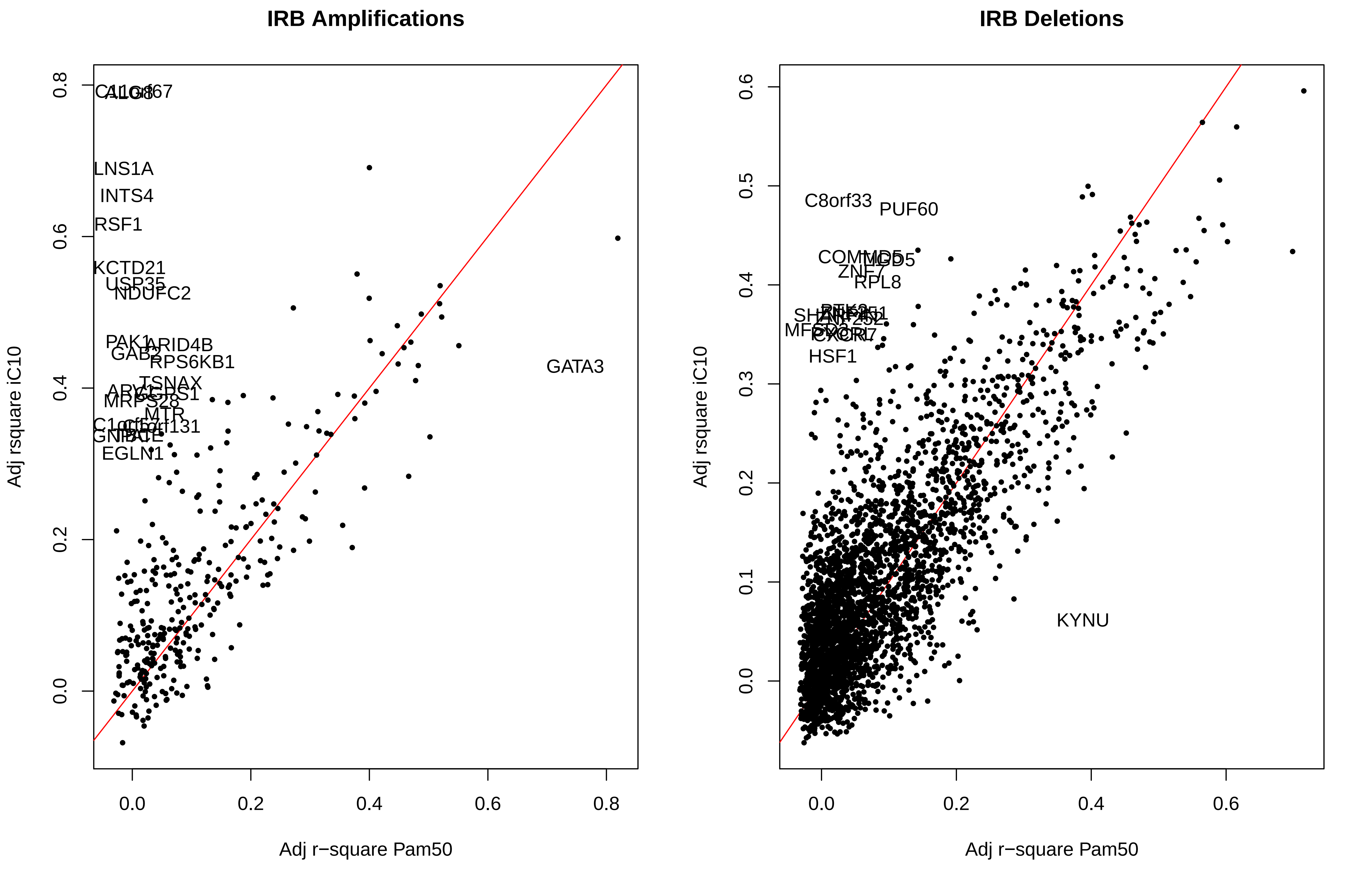
IRB Amplifications
Adj rsquare iC10
Adj r−square Pam50
0.0
0.2
0.4
0.6
0.8
C11orf67
LNS1A
INTS4
RSF1
KCTD21
USP35
NDUFC2
PAK1
ARID4B
GAB2
RPS6KB1
TSNAX
MRPS28
MTR
EGLN1
GATA3
IRB Deletions
Adj rsquare iC10
Adj r−square Pam50
0.0
0.1
0.2
0.3
0.4
0.5
0.6
C8orf33
PUF60
ZNF7
RPL8
HSF1
KYNU

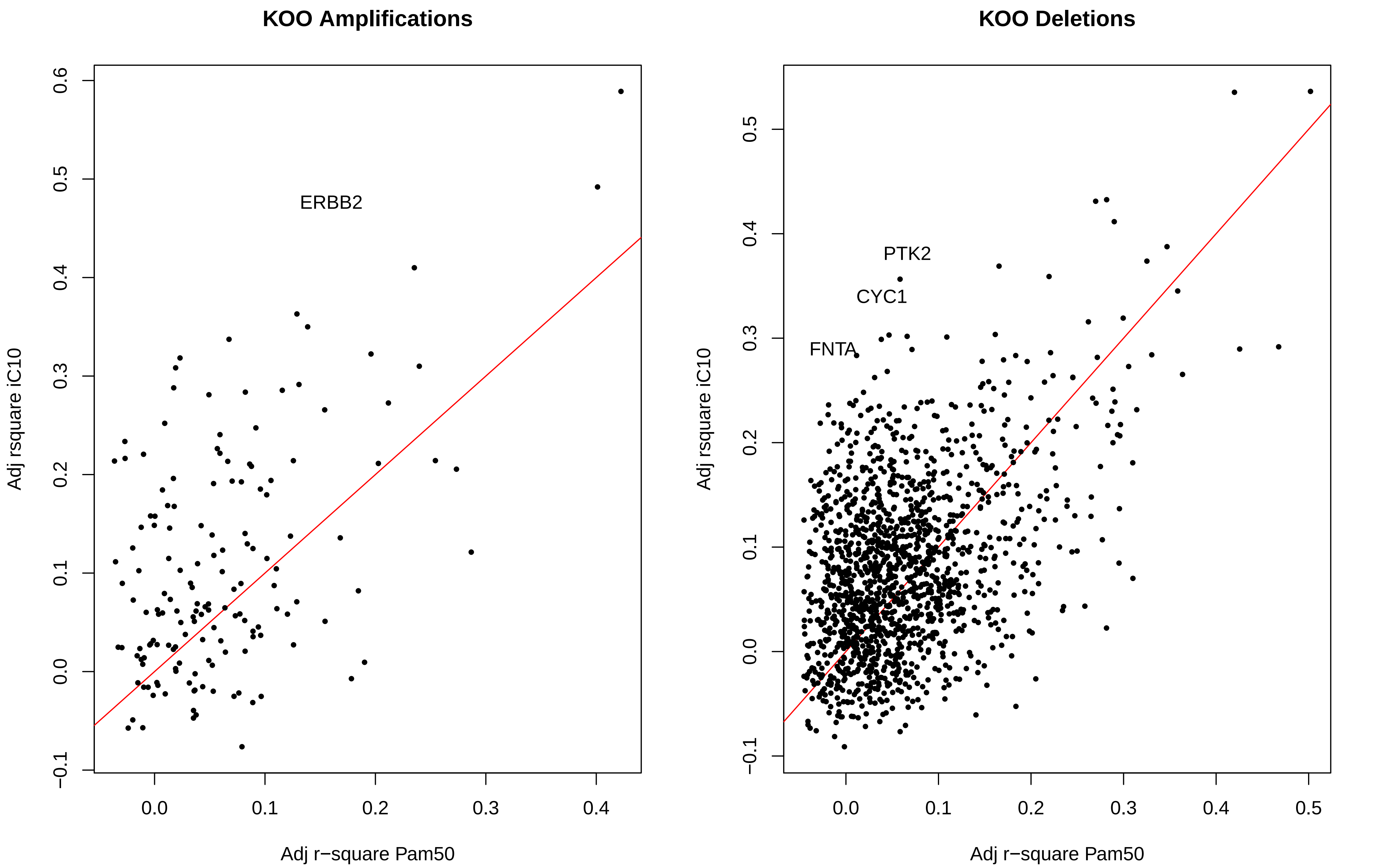
KOO Amplifications
ERBB2
Adj rsquare iC10
Adj r−square Pam50
0.0
0.1
0.2
0.3
0.4
0.5
0.6
−0.1
KOO Deletions
PTK2
CYC1
FNTA
Adj rsquare iC10
Adj r−square Pam50

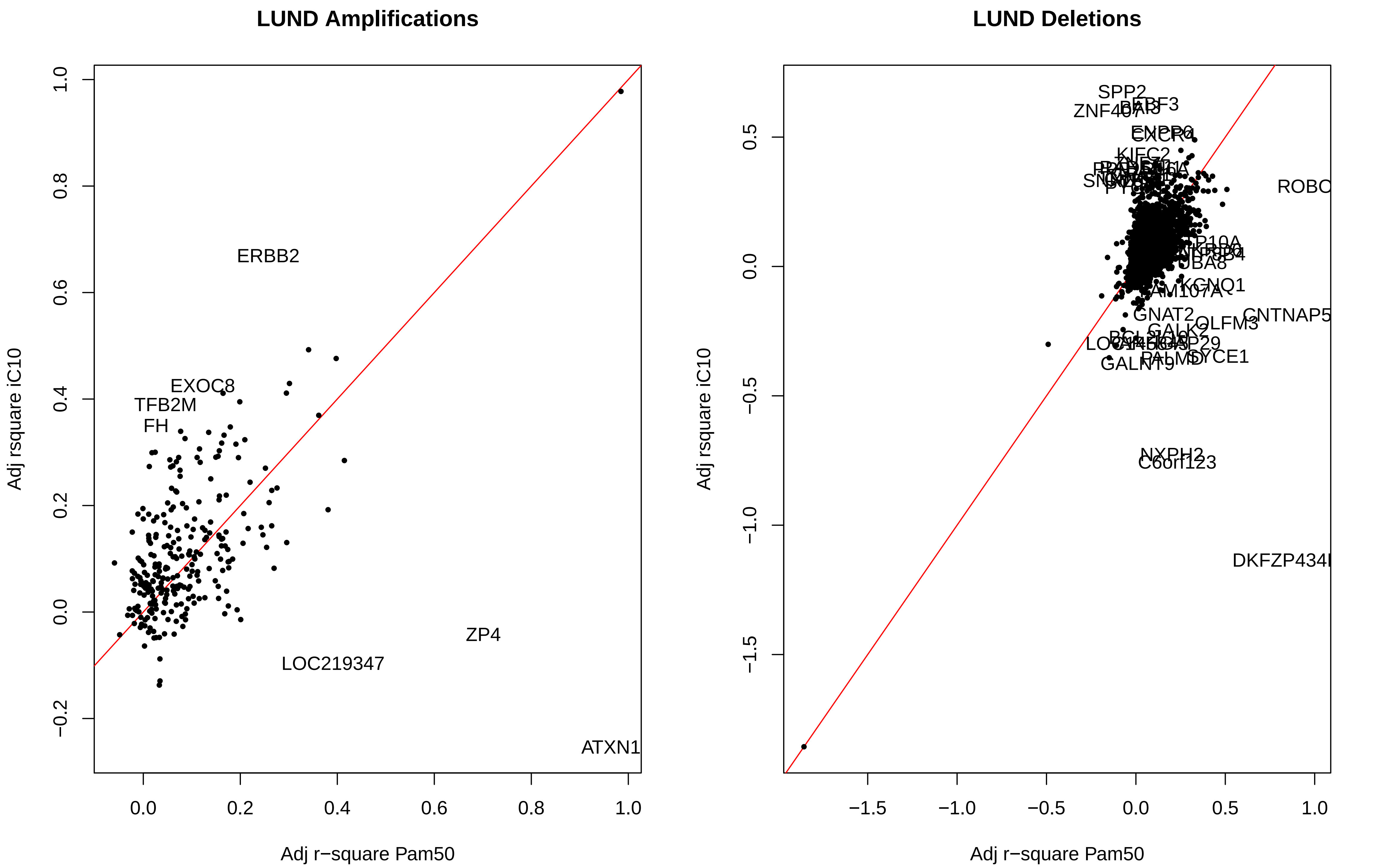

LUND Amplifications
Adj rsquare iC10
Adj r−square Pam50
−0.2
0.0
0.2
0.4
0.6
0.8
1.0
ERBB2
EXOC8
TFB2M
FH
ZP4
LOC219347
ATXN1
LUND Deletions
Adj rsquare iC10
Adj r−square Pam50
−1.5
−1.0
−0.5
0.0
0.5
1.0
SPP2
ROBO
KCNQ1
UBA8
GNAT2
OLFM3
CNTNAP5
GALNT9
SYCE1
NXPH2
C6orf123
DKFZP434I

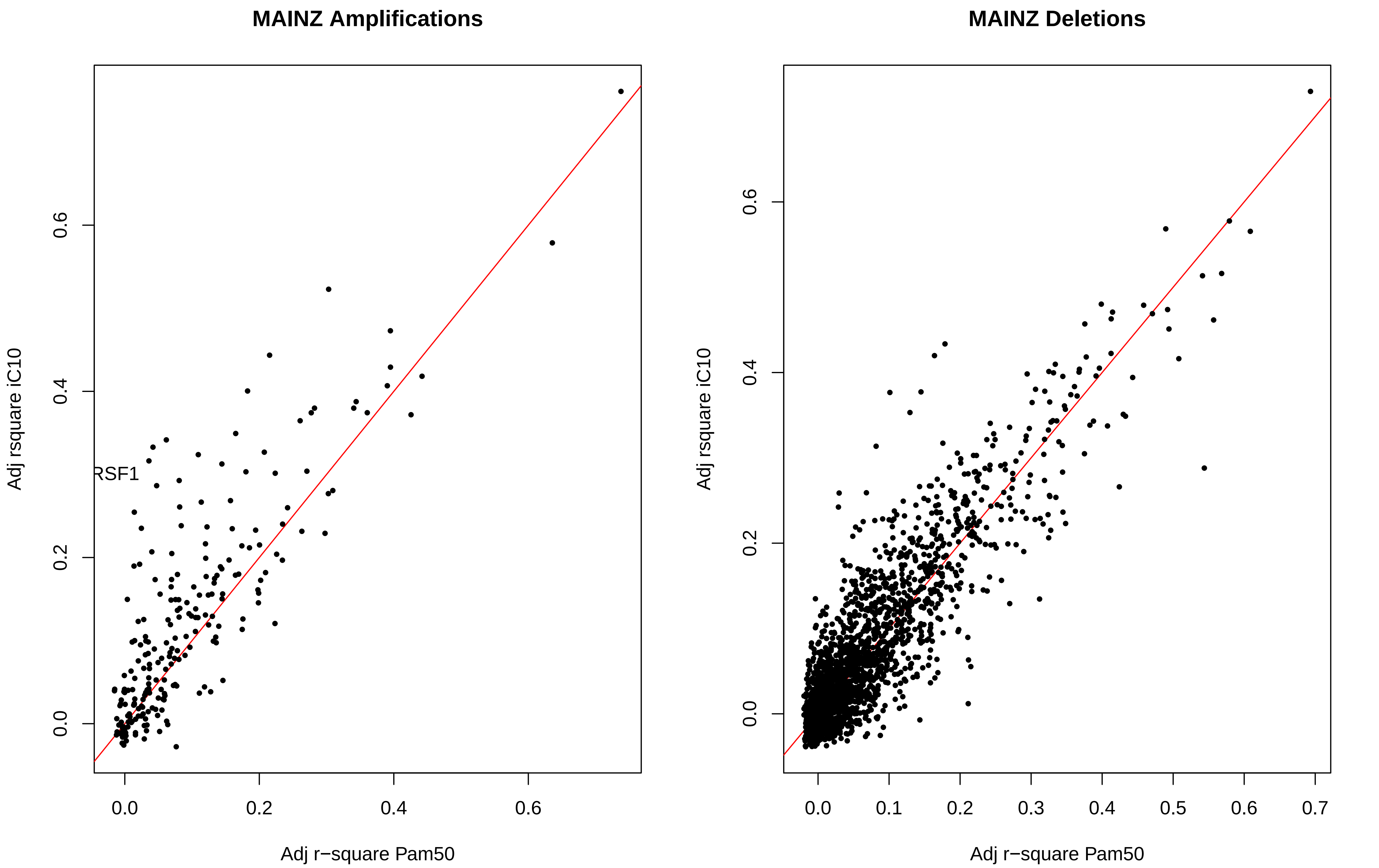
MAINZ Amplifications
MAINZ Deletions
Adj rsquare iC10
Adj r−square Pam50
RSF1
0.0
0.2
0.4
0.6
0.0
0.1
0.2
0.3
0.4
0.5
0.6
0.7

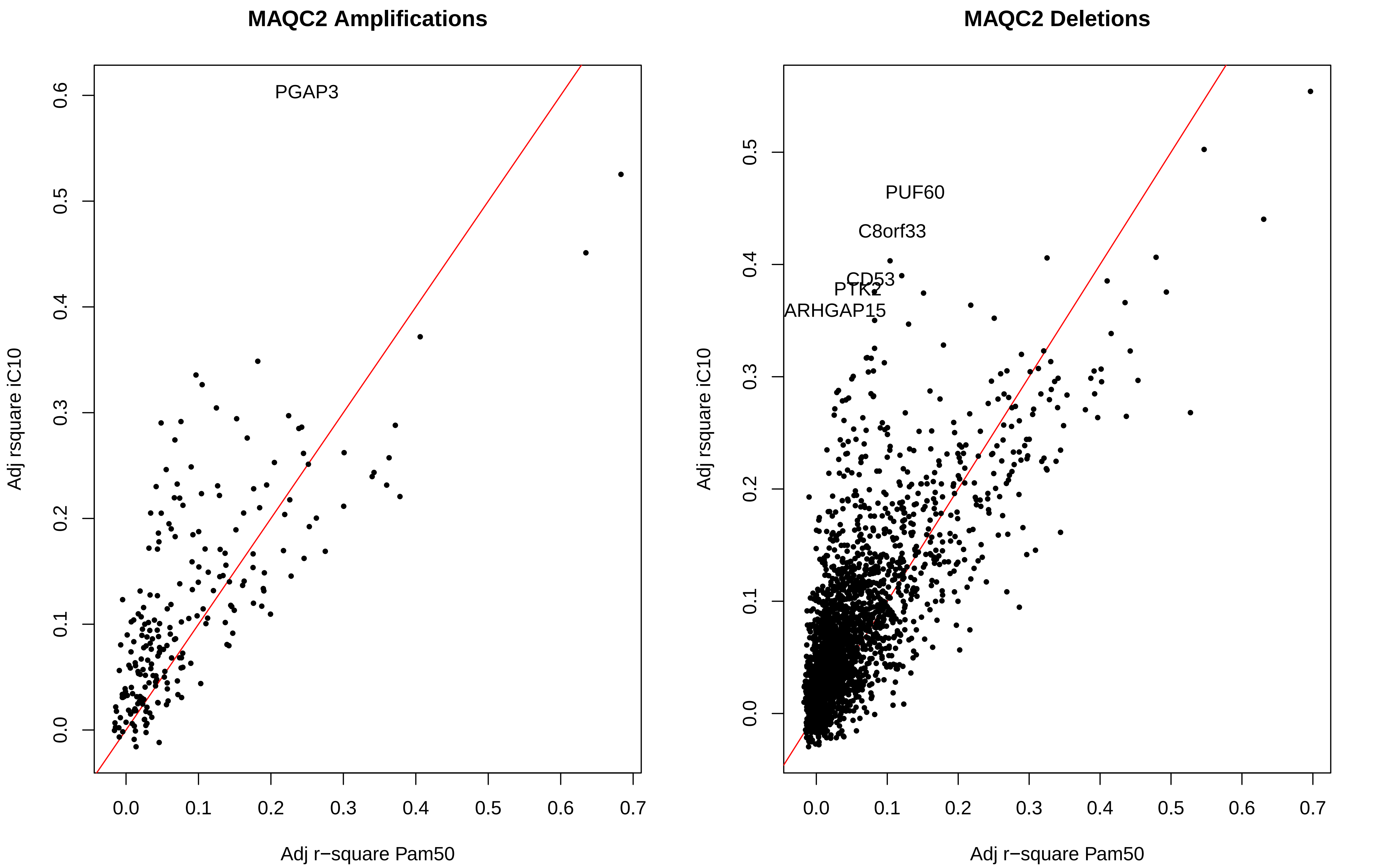
MAQC2 Amplifications
PGAP3
Adj rsquare iC10
Adj r−square Pam50
0.0
0.1
0.2
0.3
0.4
0.5
0.6
0.7
MAQC2 Deletions
PUF60
C8orf33
CD53
PTK2
ARHGAP15
Adj rsquare iC10
Adj r−square Pam50

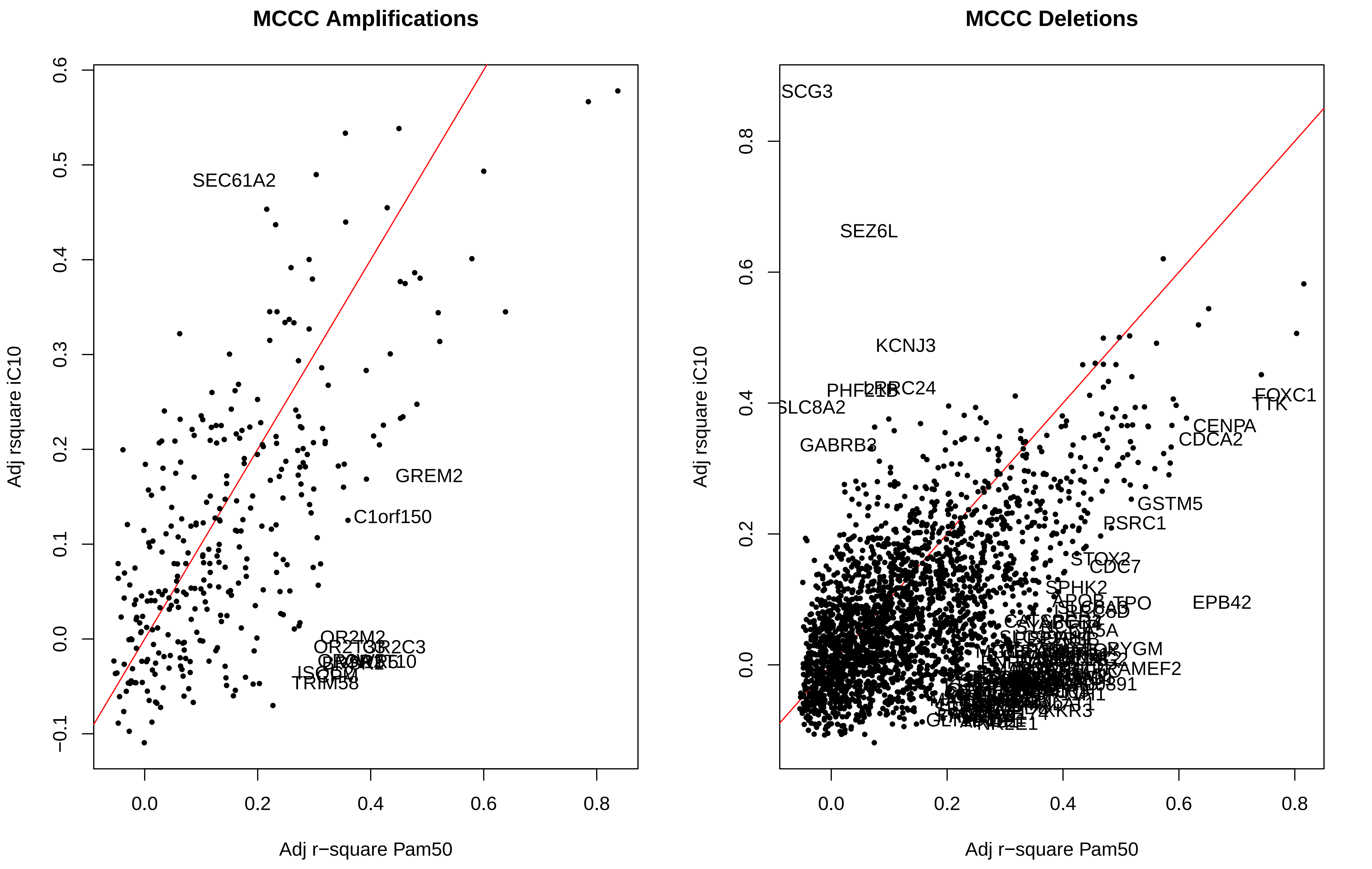
MCCC Amplifications
Adj rsquare iC10
Adj r−square Pam50
SEC61A2
GREM2
C1orf150
OR2M2
OR2T33
OR2C3
TRIM58
MCCC Deletions
SCG3
SEZ6L
KCNJ3
PHF21B
LRRC24
SLC8A2
GABRB3
FOXC1
TTK
CENPA
CDCA2
GSTM5
PSRC1
STOX2
CDC7
SPHK2
TPO
EPB42
PYGM
PRAMEF2

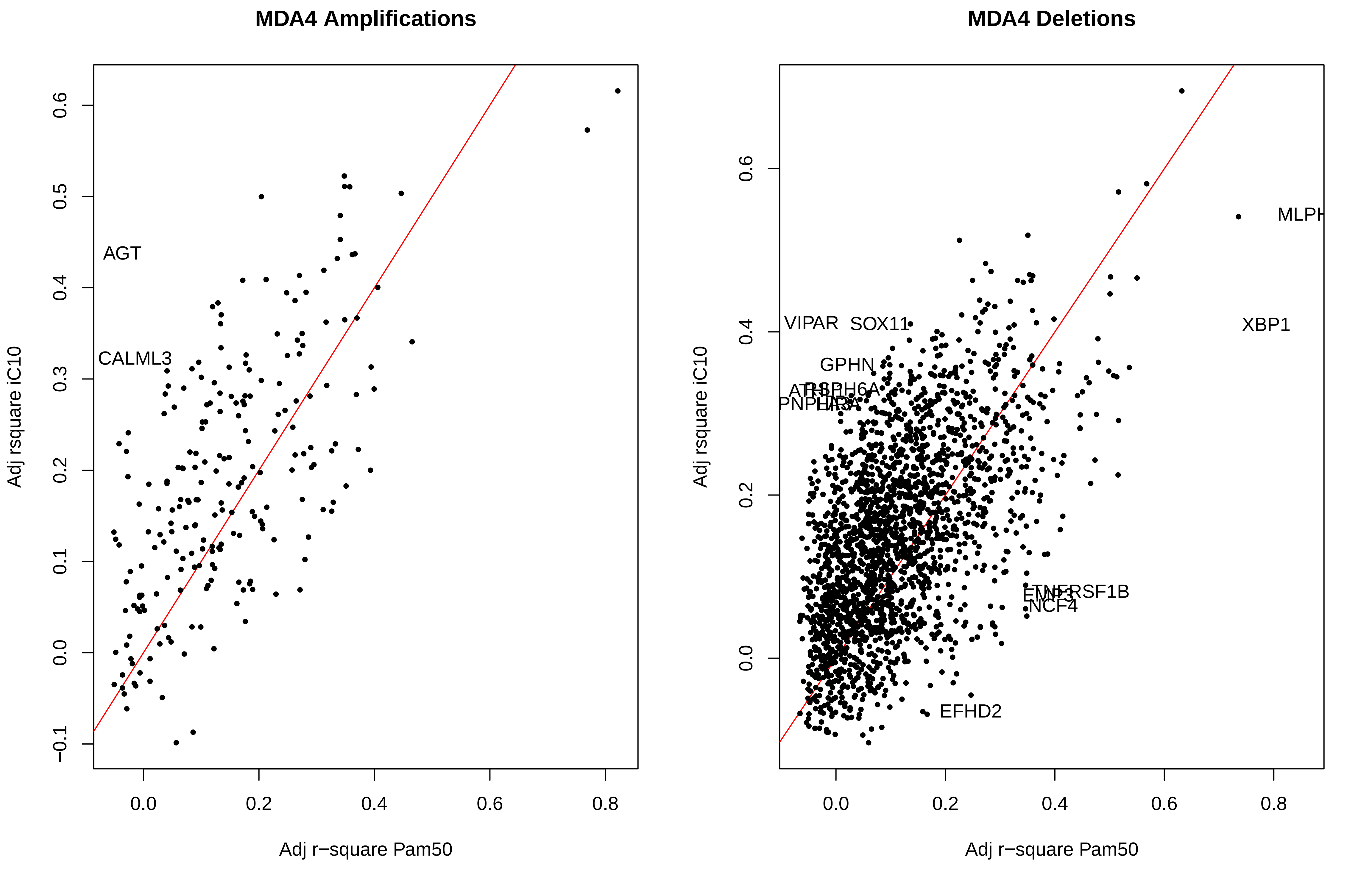

MDA4 Amplifications
AGT
CALML3
Adj rsquare iC10
Adj r−square Pam50
MDA4 Deletions
VIPAR
SOX11
GPHN
ATRSPH6A
PNPLA3
MLPH
XBP1
TNFRSF1B
EMP3
NCF4
EFHD2
Adj rsquare iC10
Adj r−square Pam50

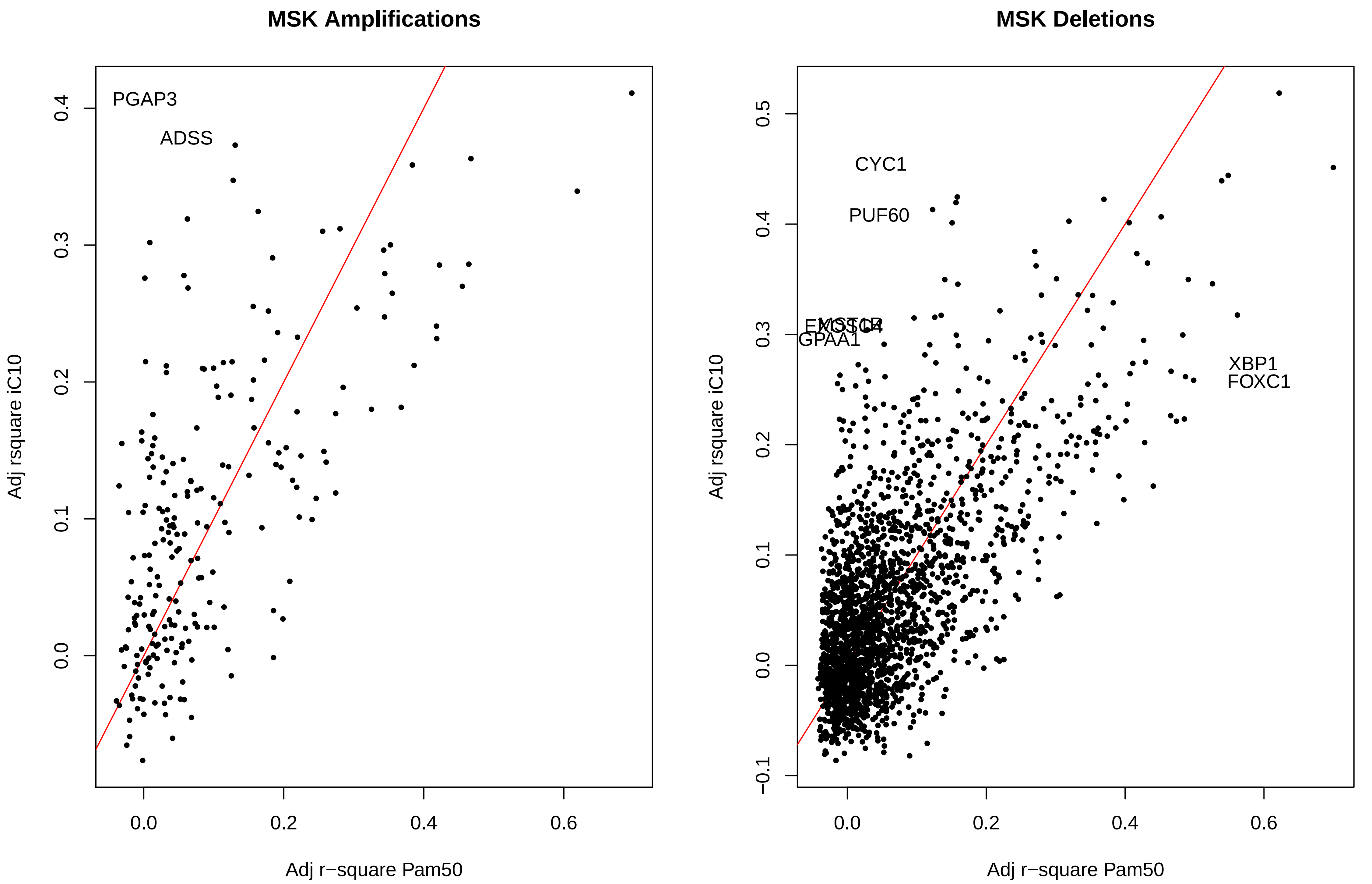
MSK Amplifications
Adj rsquare iC10
Adj r−square Pam50
0.0
0.1
0.2
0.3
0.4
0.0
0.2
0.4
0.6
PGAP3
ADSS
MSK Deletions
Adj rsquare iC10
Adj r−square Pam50
−0.1
0.0
0.1
0.2
0.3
0.4
0.5
0.0
0.2
0.4
0.6
CYC1
PUF60
EXOSC4
GPAA1
XBP1
FOXC1

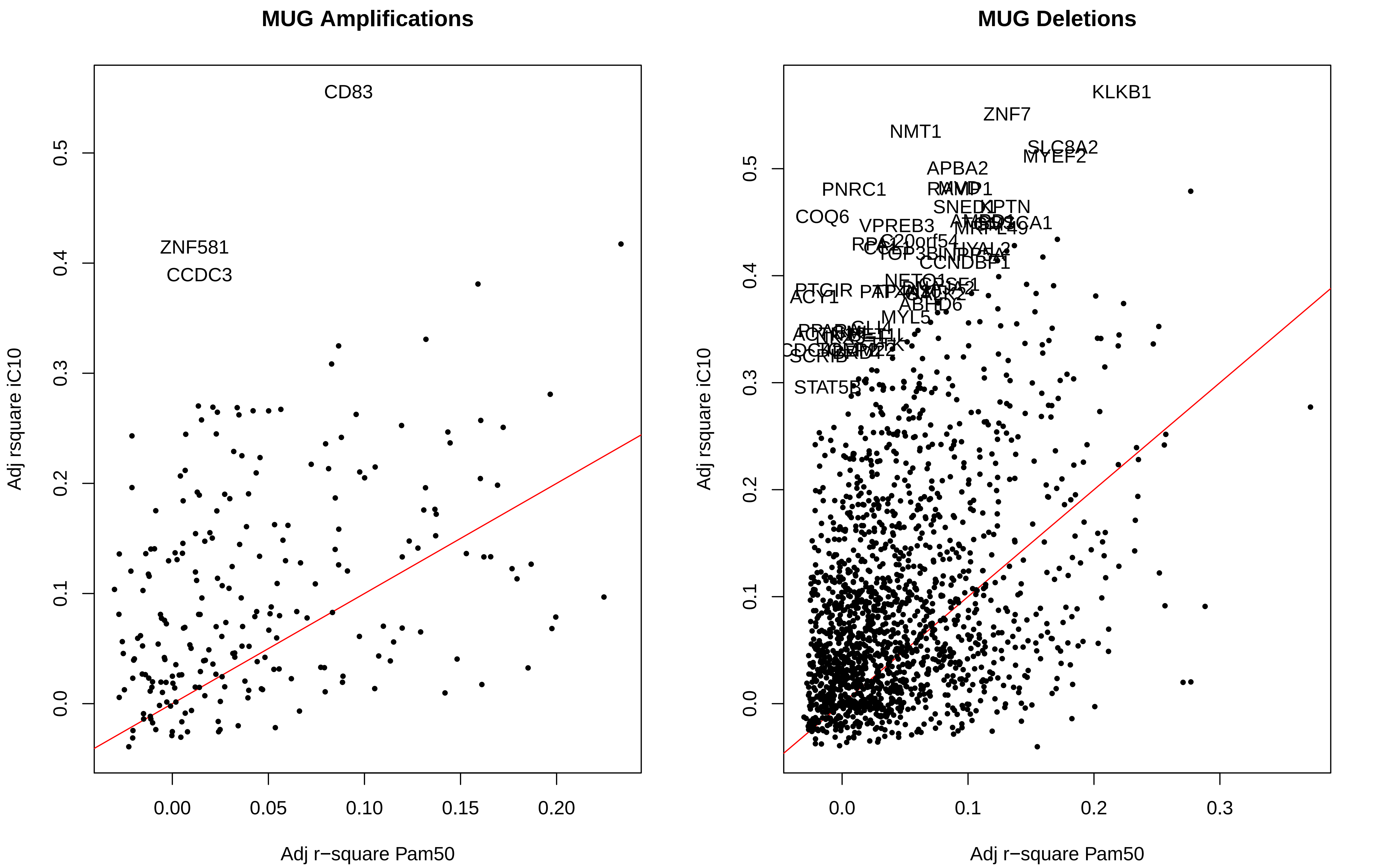

MUG Amplifications
CD83
ZNF581
CCDC3
Adj rsquare iC10
Adj r−square Pam50
0.0
0.1
0.2
0.3
0.4
0.5
0.00
0.05
0.10
0.15
0.20
MUG Deletions
KLKB1
ZNF7
NMT1
MYEF2
APBA2
PNRC1
COQ6
VPREB3
KPTN
MRPL49
CCNDBP1
PTGIR
ACY1
ABHD6
MYL5
SCRIB
STAT5B
Adj rsquare iC10
Adj r−square Pam50
0.0
0.1
0.2
0.3
0.4
0.5
0.0
0.1
0.2
0.3

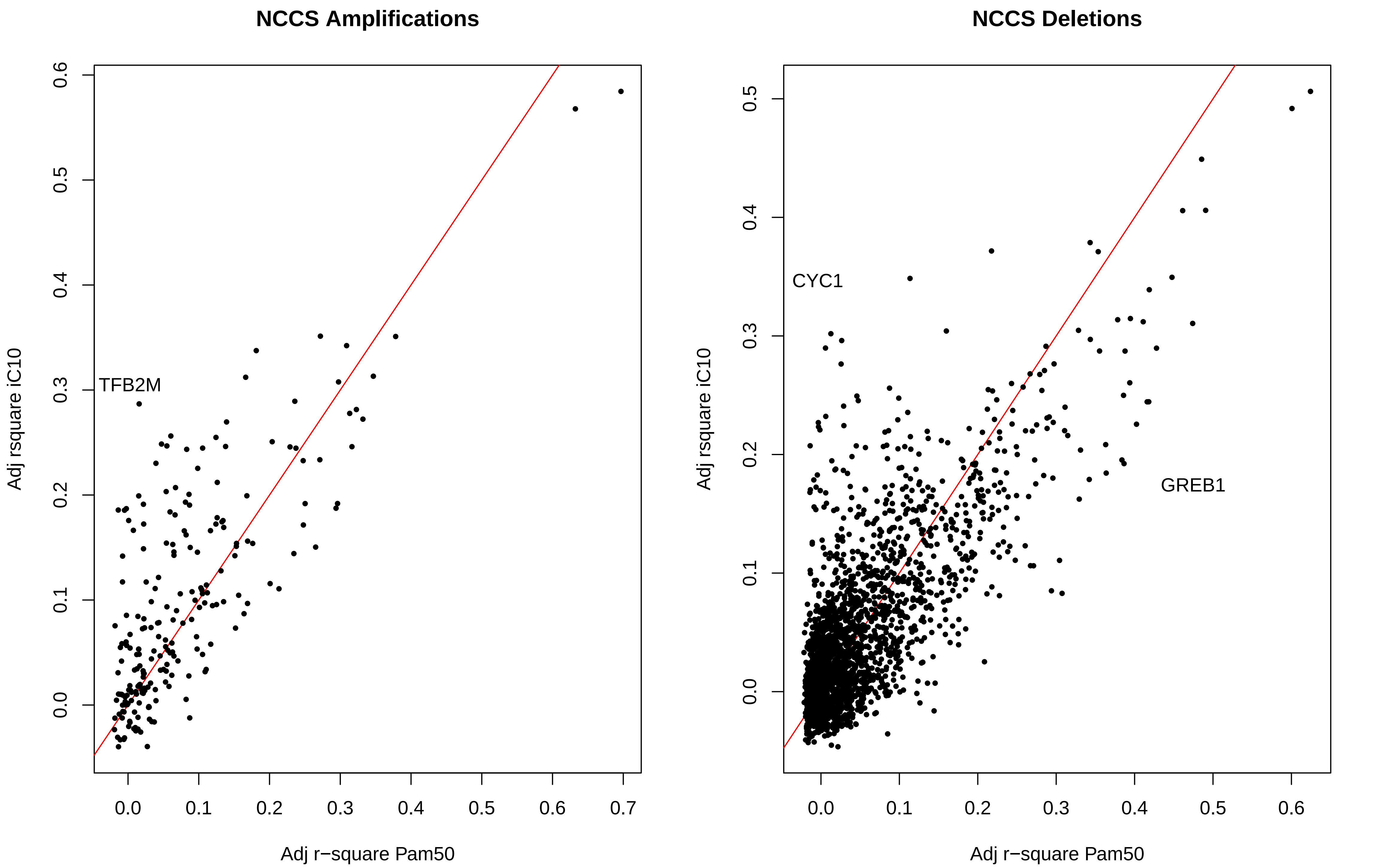
NCCS Amplifications
NCCS Deletions
TFB2M
CYC1
GREB1
Adj rsquare iC10
Adj r−square Pam50
0.0
0.1
0.2
0.3
0.4
0.5
0.6
0.7

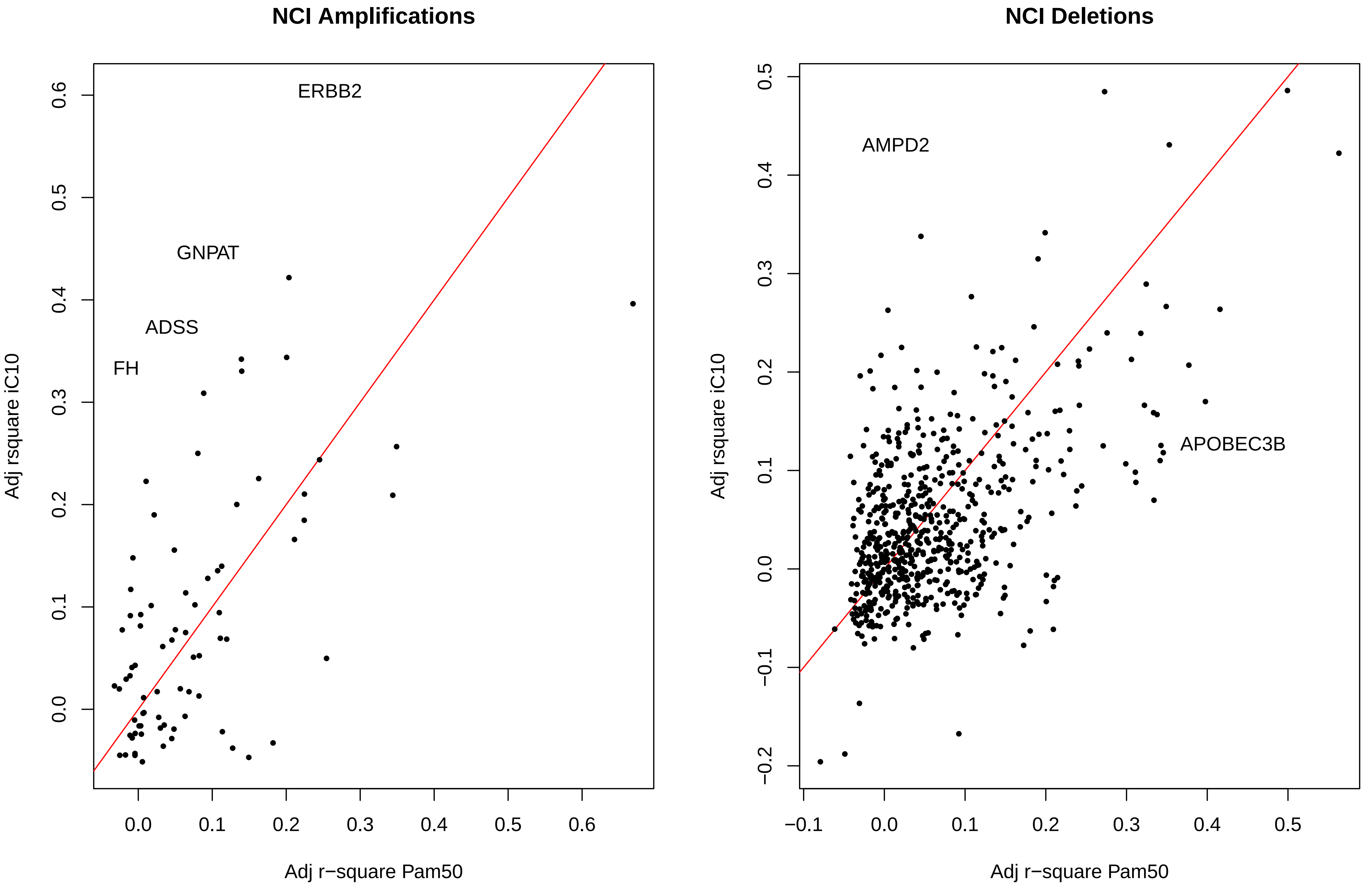

NCI Amplifications
ERBB2
GNPAT
ADSS
FH
Adj rsquare iC10
Adj r−square Pam50
0.0
0.1
0.2
0.3
0.4
0.5
0.6
NCI Deletions
AMPD2
APOBEC3B
Adj rsquare iC10
Adj r−square Pam50
−0.2
−0.1
0.0
0.1
0.2
0.3
0.4
0.5

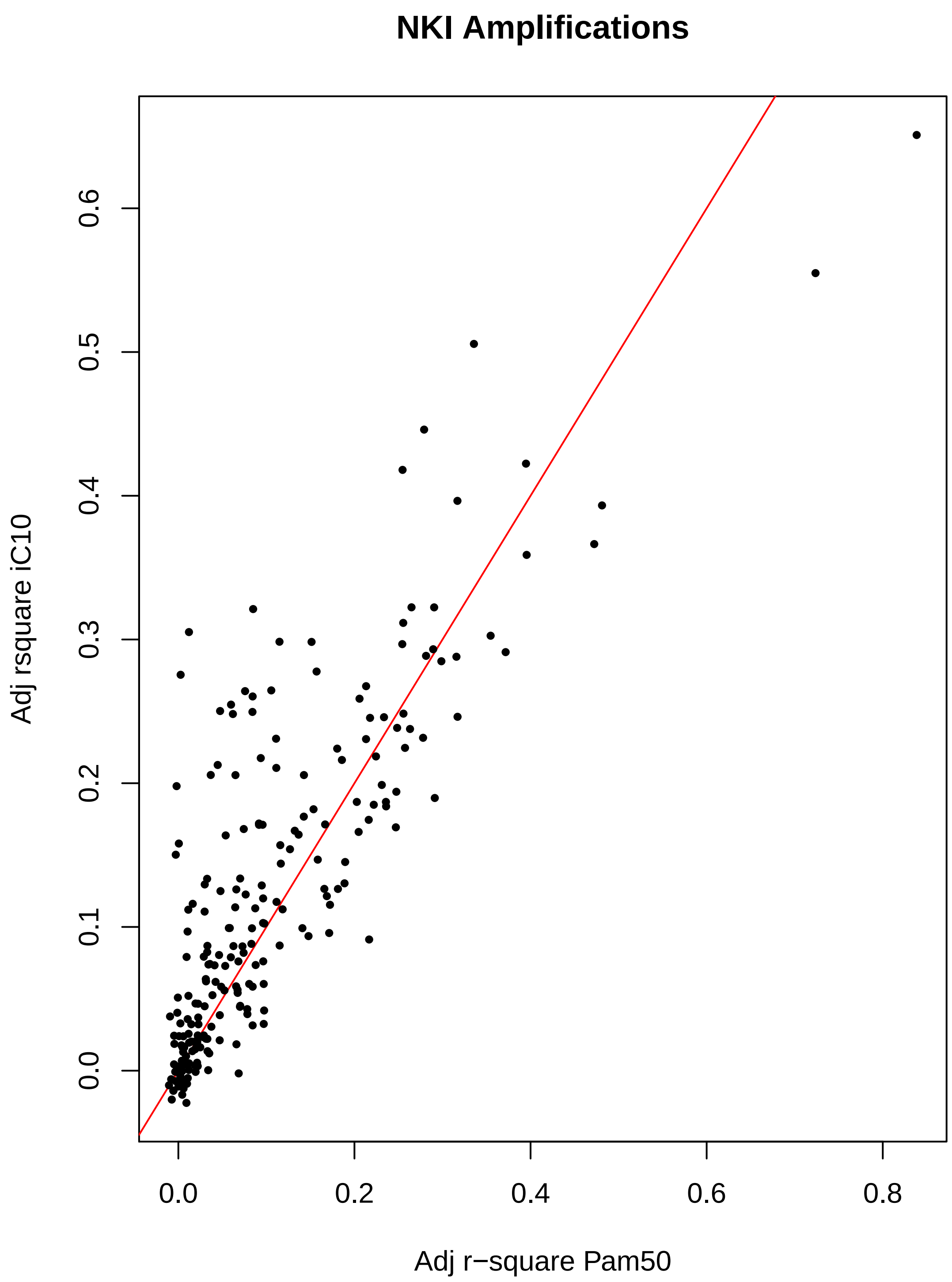
NKI Amplifications
Adj rsquare iC10
Adj r−square Pam50
0.0
0.1
0.2
0.3
0.4
0.5
0.6
0.0
0.2
0.4
0.6
0.8

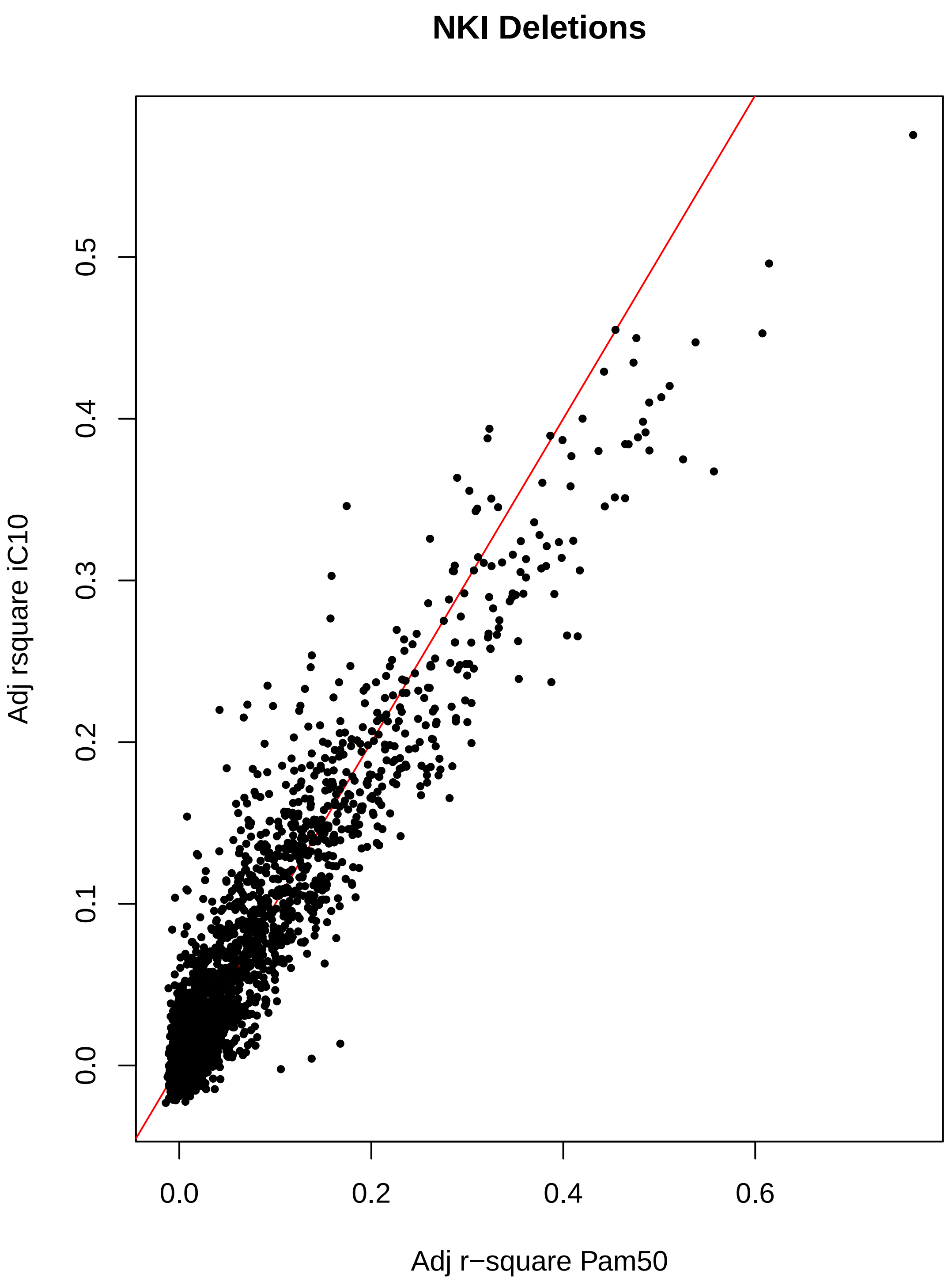
NKI Deletions
Adj rsquare iC10
Adj r−square Pam50
0.0
0.1
0.2
0.3
0.4
0.5
0.0
0.2
0.4
0.6

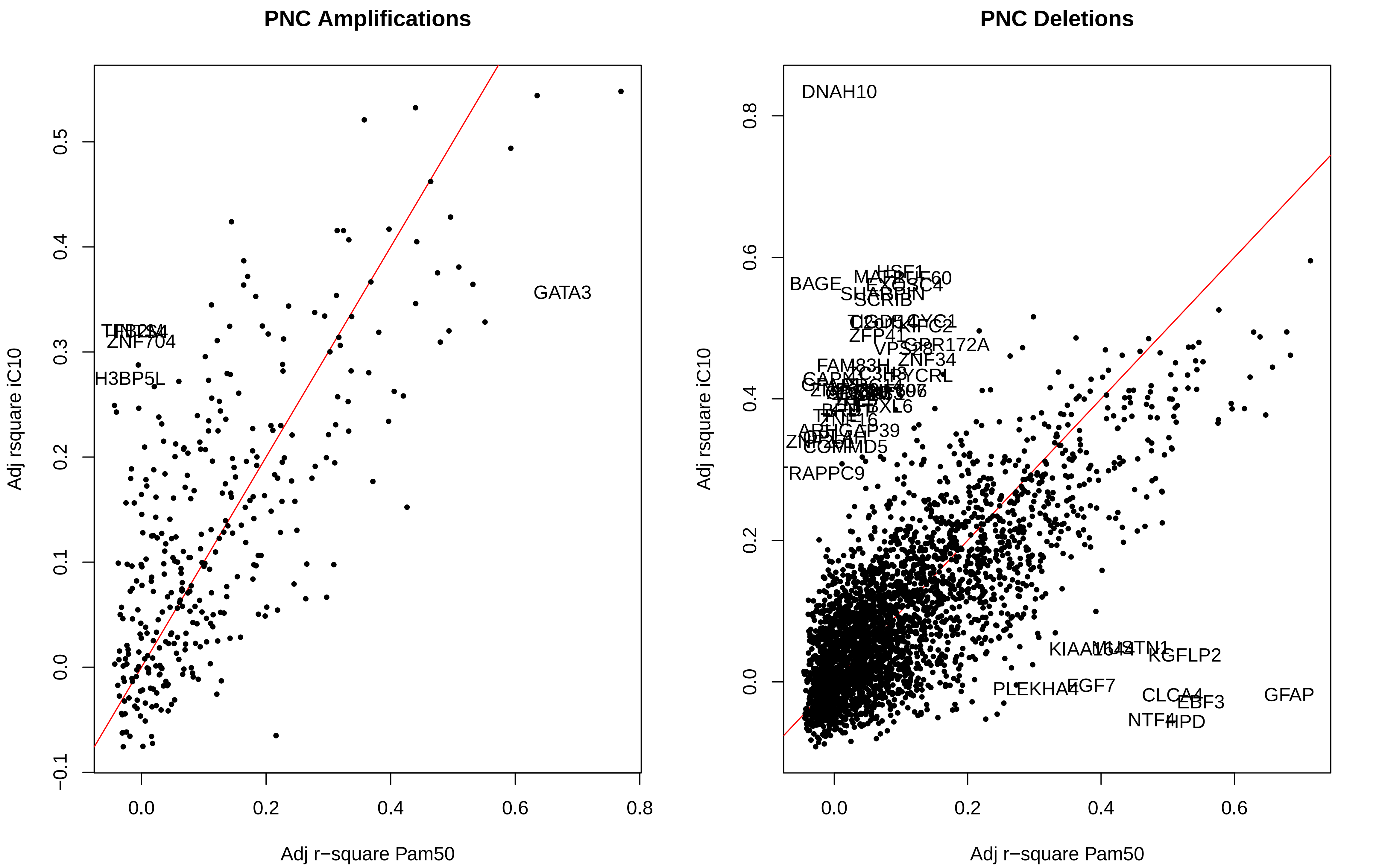
PNC Amplifications
Adj rsquare iC10
Adj r−square Pam50
0.0
0.2
0.4
0.6
0.8
−0.1
0.0
0.1
0.2
0.3
0.4
0.5
GATA3
ZNF704
H3BP5L
PNC Deletions
Adj rsquare iC10
Adj r−square Pam50
0.0
0.2
0.4
0.6
0.0
0.2
0.4
0.6
0.8
DNAH10
BAGE
SCRIB
ZFP41
ZNF34
FAM83H
ARHGAP39
COMMD5
RAPPC9
KGFLP2
PLEKHA4
EGF7
CLCA4
EBF3
GFAP
HPD

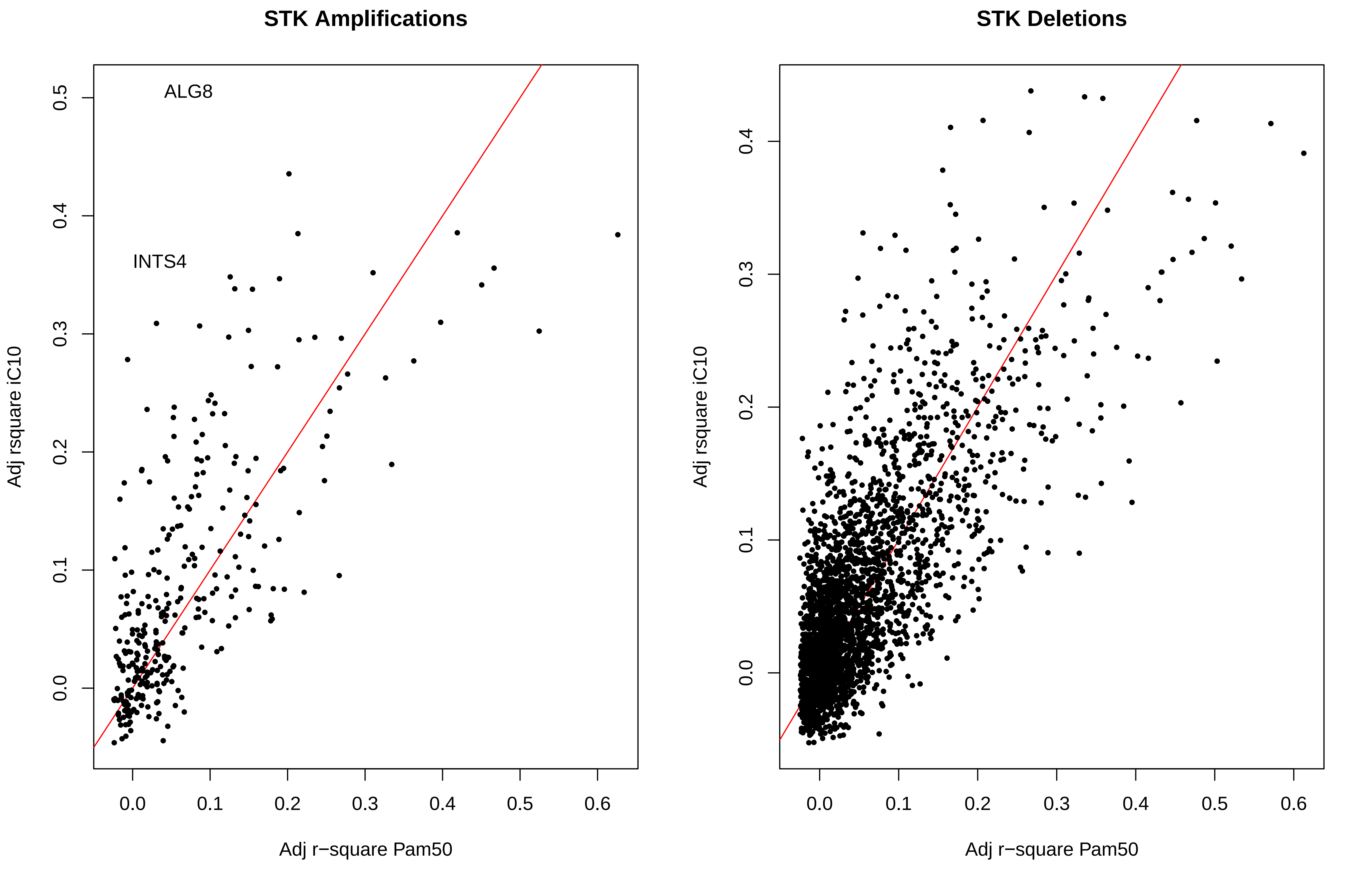

STK Amplifications
ALG8
INTS4
Adj rsquare iC10
Adj r−square Pam50
0.0
0.1
0.2
0.3
0.4
0.5
0.6
STK Deletions
Adj rsquare iC10
Adj r−square Pam50

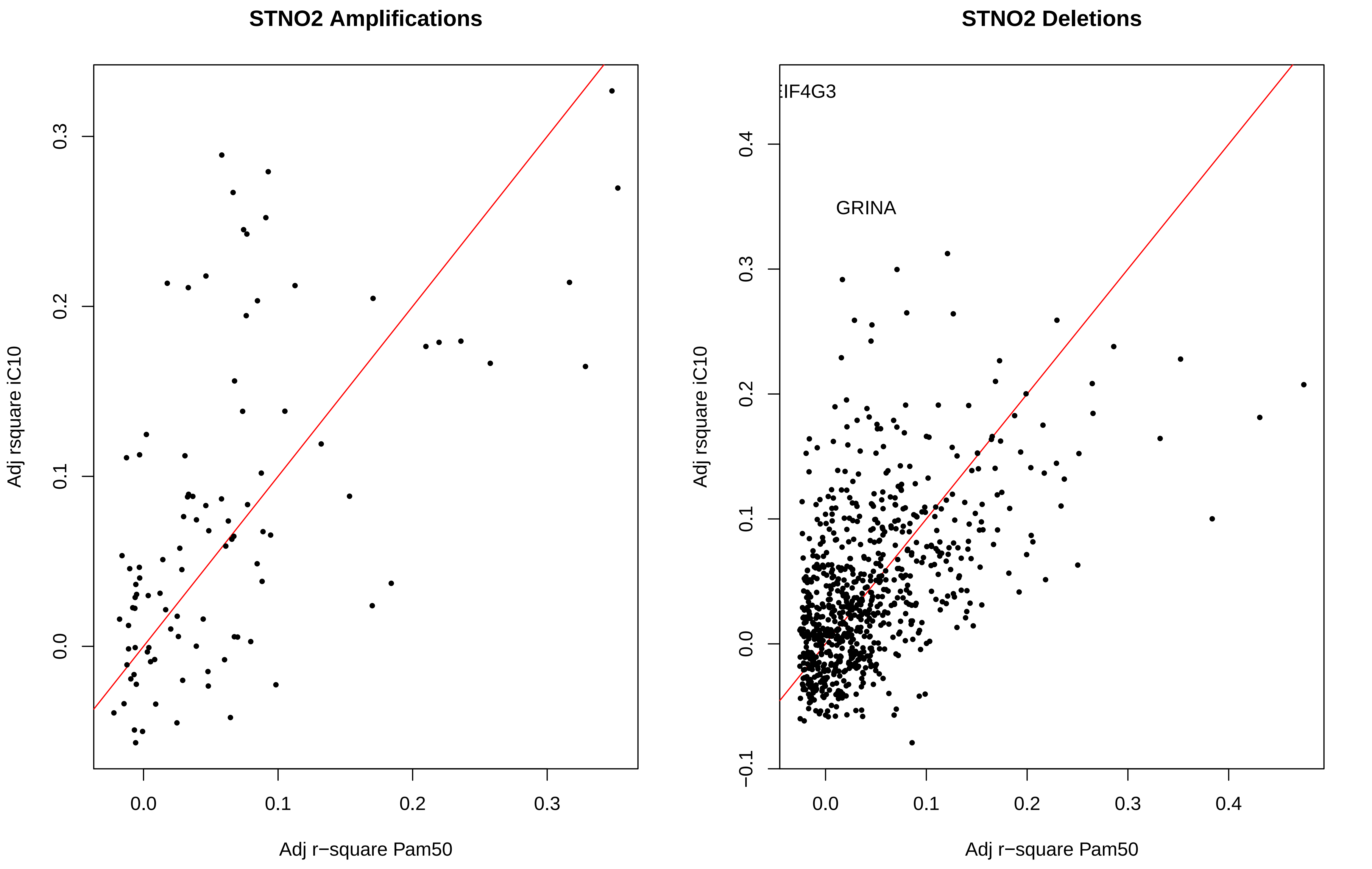

STNO2 Amplifications
Adj rsquare iC10
Adj r−square Pam50
0.0
0.1
0.2
0.3
STNO2 Deletions
EIF4G3
GRINA
−0.1
0.4

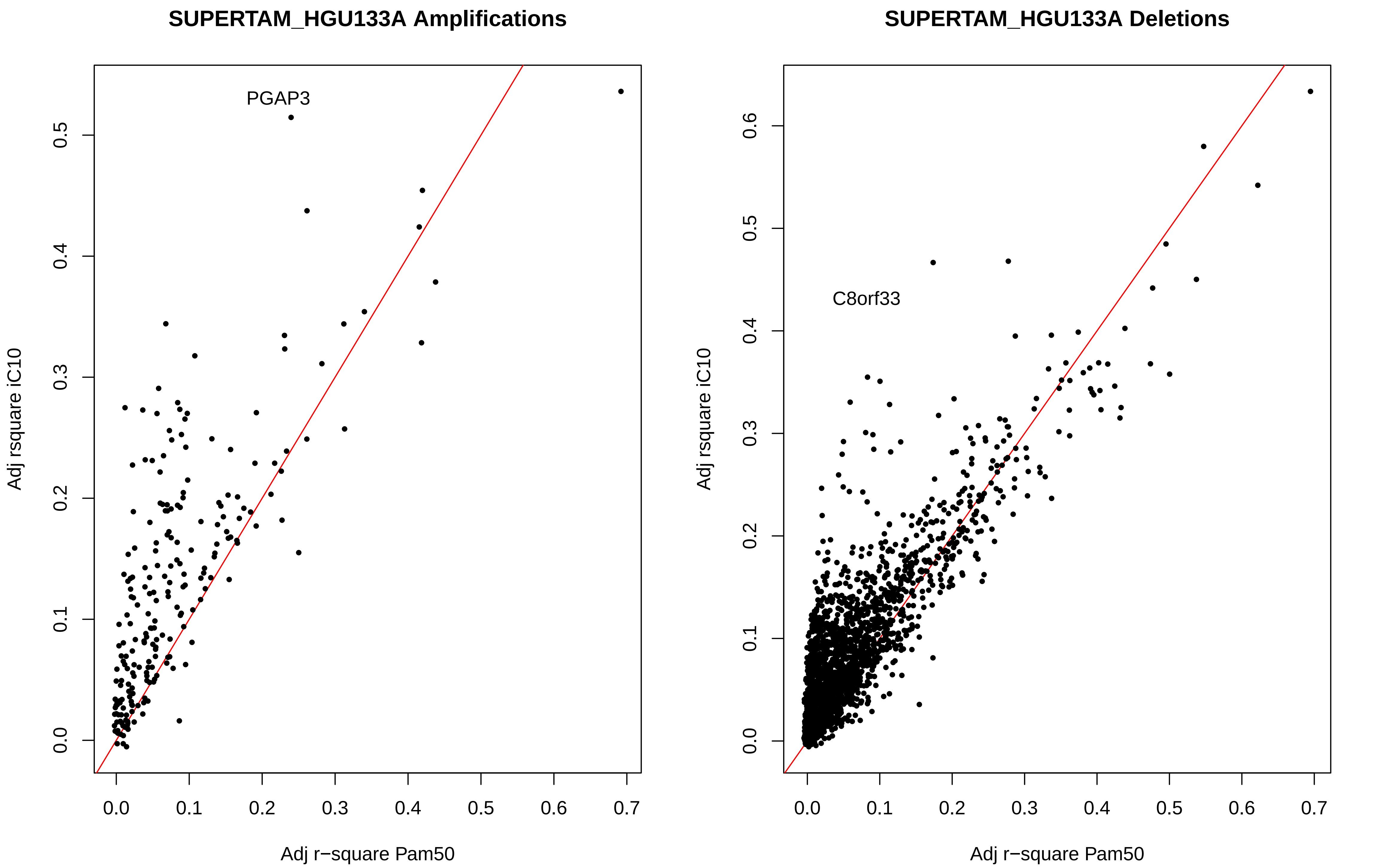
SUPERTAM_HGU133A Amplifications
PGAP3
Adj rsquare iC10
Adj r−square Pam50
0.0
0.1
0.2
0.3
0.4
0.5
0.6
0.7
SUPERTAM_HGU133A Deletions
C8orf33
Adj rsquare iC10
Adj r−square Pam50

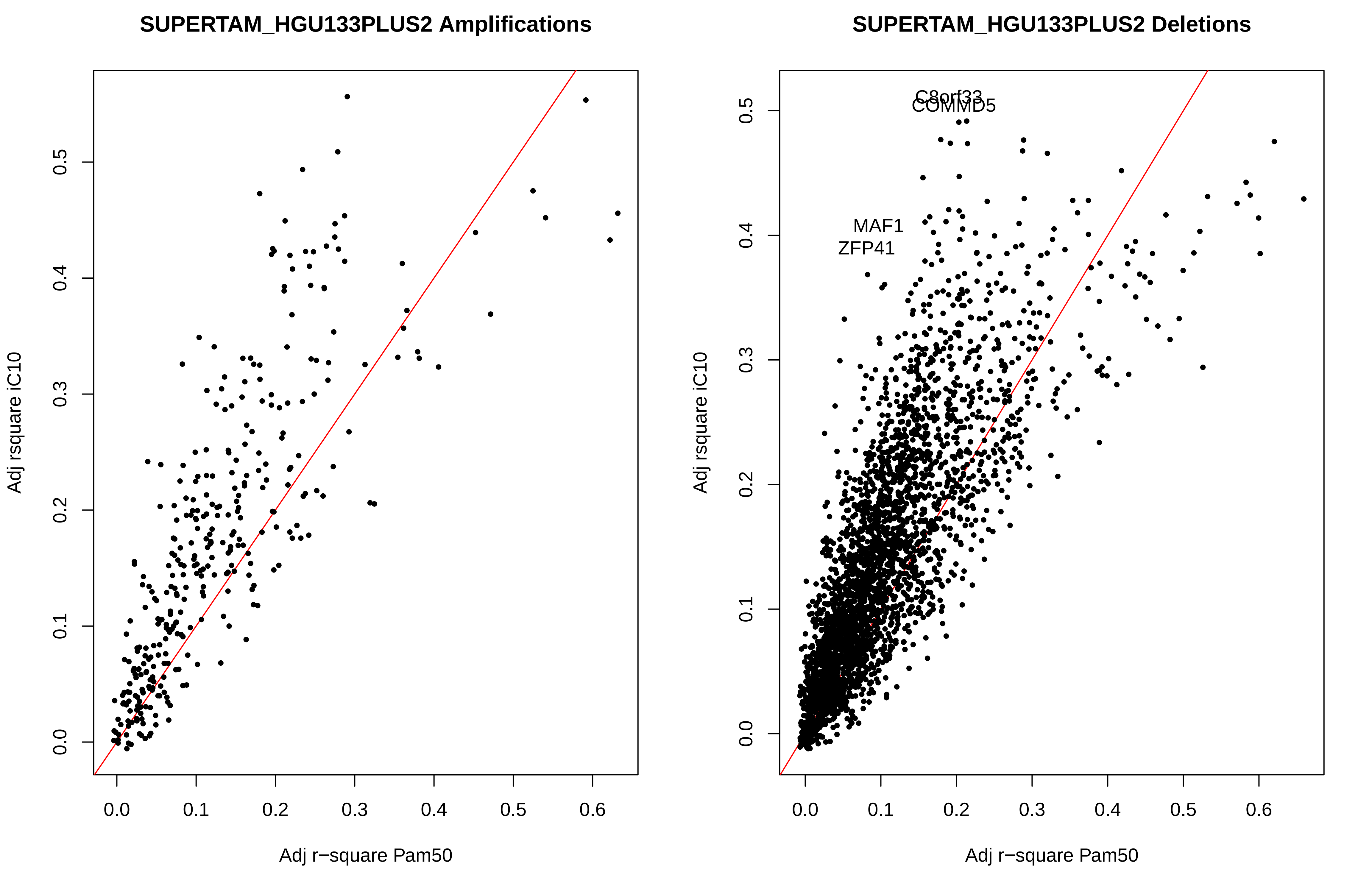
SUPERTAM_HGU133PLUS2 Amplifications
Adj rsquare iC10
Adj r−square Pam50
0.0
0.1
0.2
0.3
0.4
0.5
0.6
SUPERTAM_HGU133PLUS2 Deletions
C8orf33
COMMD5
MAF1
ZFP41
Adj rsquare iC10
Adj r−square Pam50

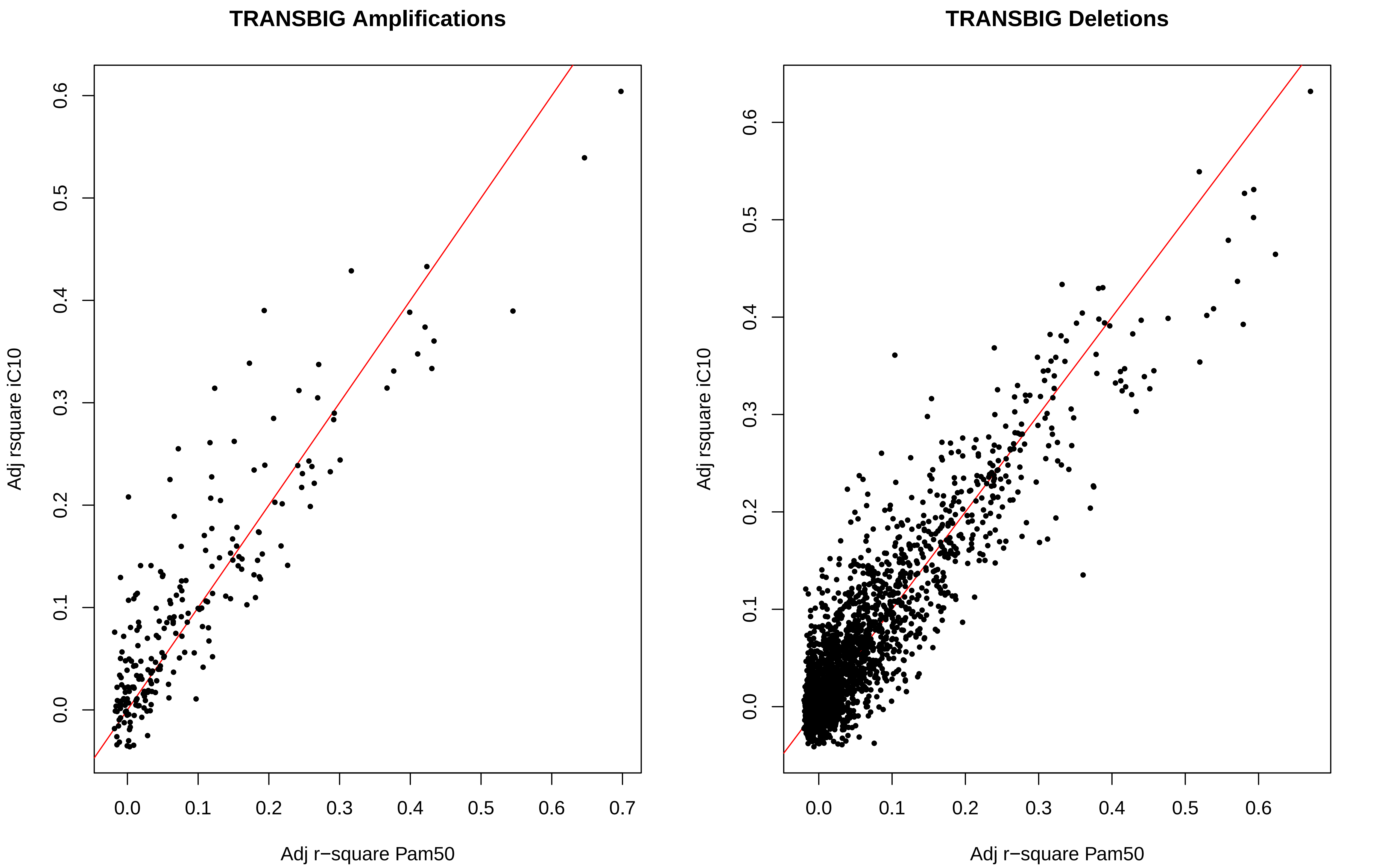
TRANSBIG Amplifications
TRANSBIG Deletions
Adj rsquare iC10
Adj r−square Pam50
0.0
0.1
0.2
0.3
0.4
0.5
0.6
0.7

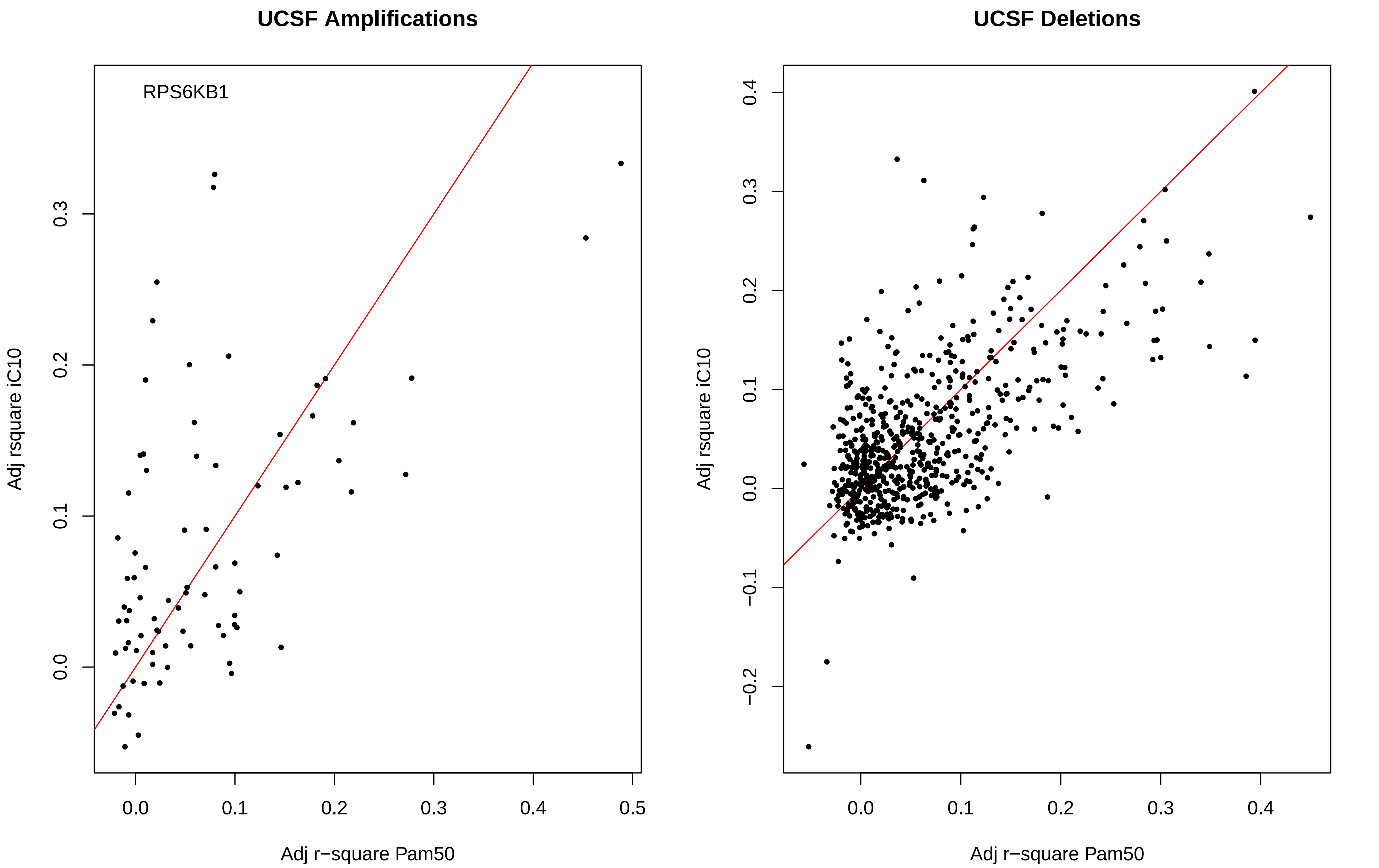
UCSF Amplifications
RPS6KB1
Adj rsquare iC10
Adj r−square Pam50
0.0
0.1
0.2
0.3
0.4
0.5
UCSF Deletions
−0.2
−0.1
0.0
0.1
0.2
0.3
0.4

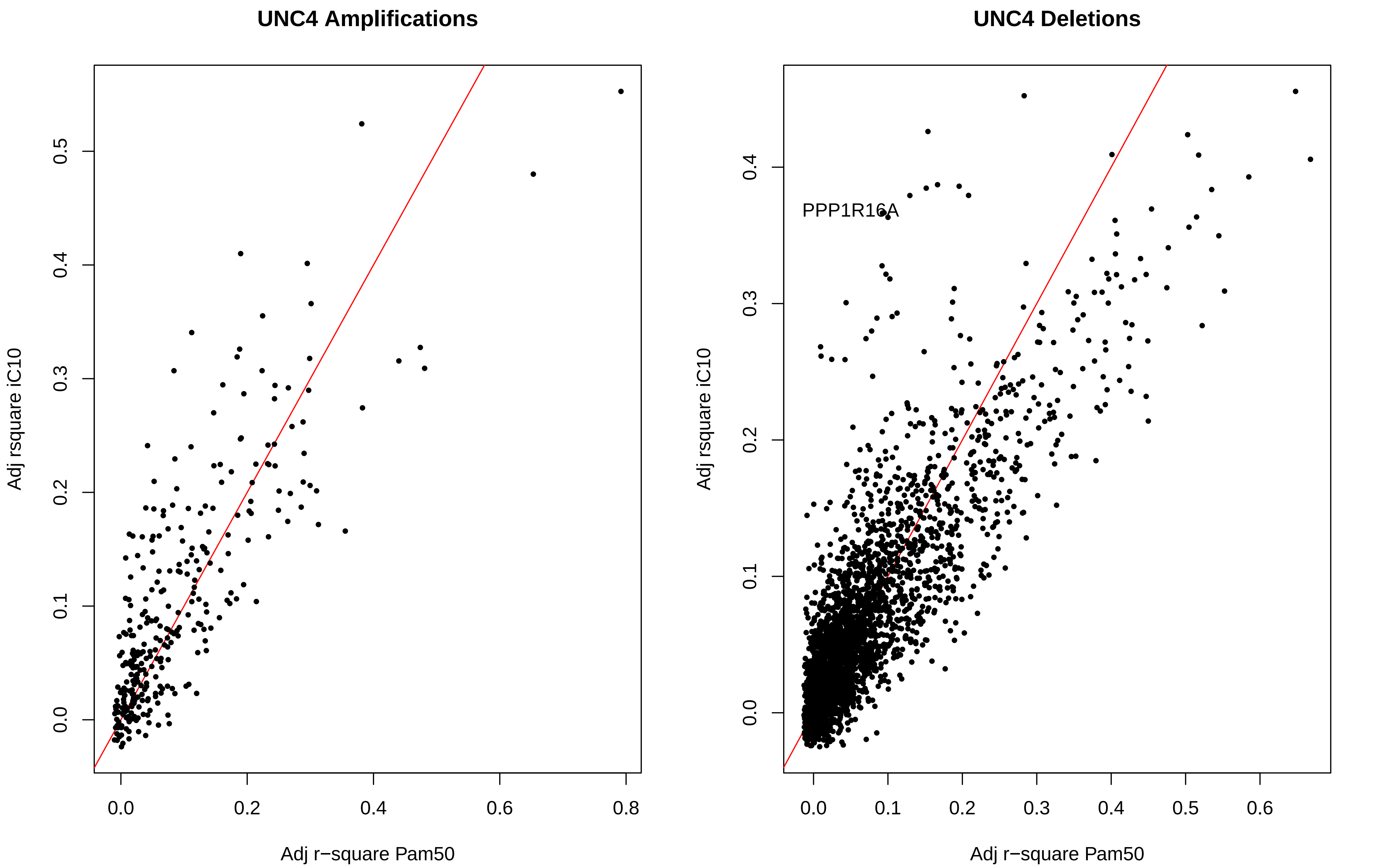
UNC4 Amplifications
Adj rsquare iC10
Adj r−square Pam50
0.0
0.1
0.2
0.3
0.4
0.5
0.0
0.2
0.4
0.6
0.8
UNC4 Deletions
Adj rsquare iC10
Adj r−square Pam50
PPP1R16A
0.0
0.1
0.2
0.3
0.4
0.0
0.1
0.2
0.3
0.4
0.5
0.6

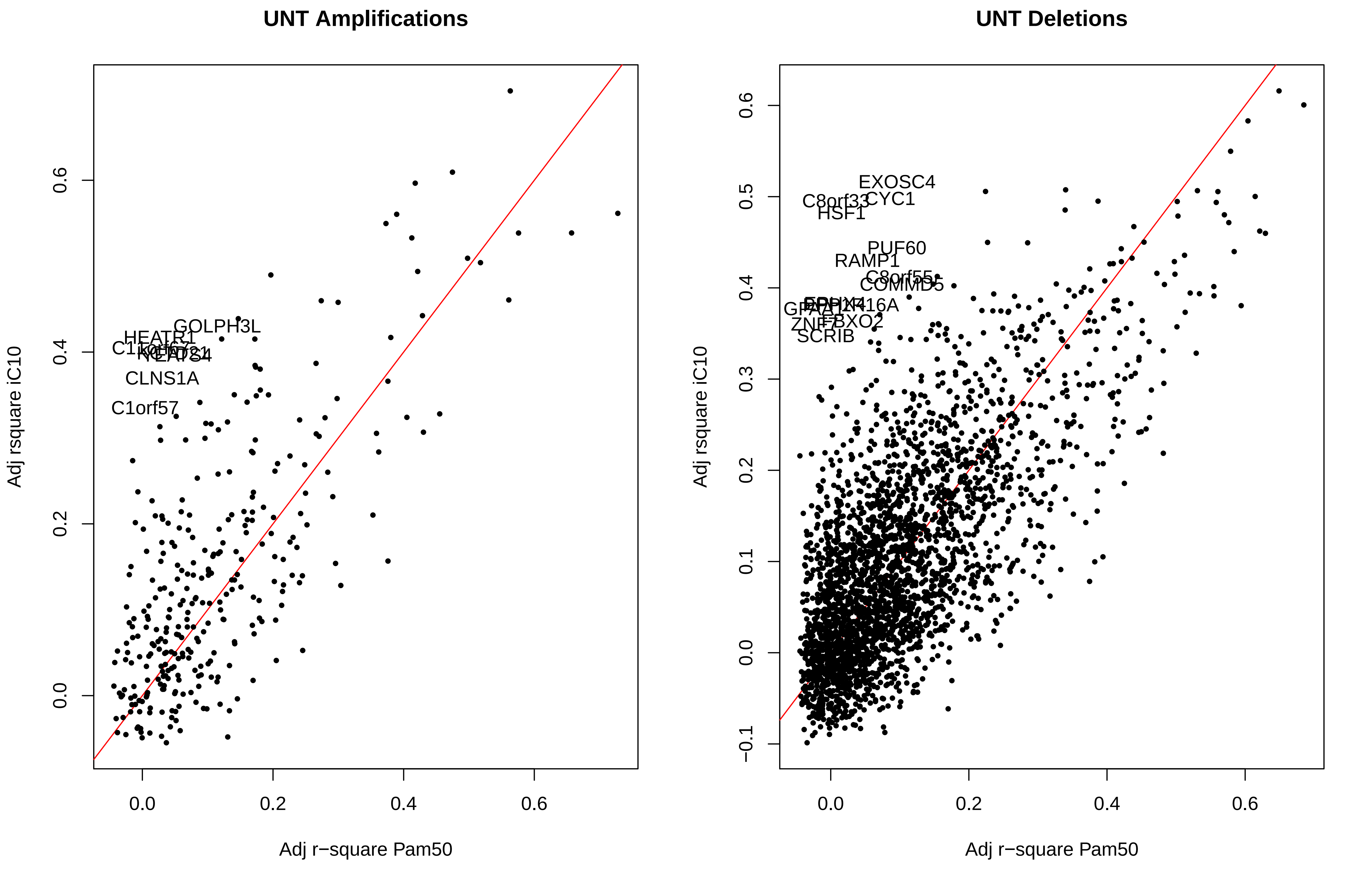

UNT Amplifications
Adj rsquare iC10
Adj r−square Pam50
GOLPH3L
HEATR1
CLNS1A
C1orf57
UNT Deletions
Adj rsquare iC10
Adj r−square Pam50
EXOSC4
C8orf33
CYC1
HSF1
PUF60
RAMP1
COMMD5
FBXO2
SCRIB

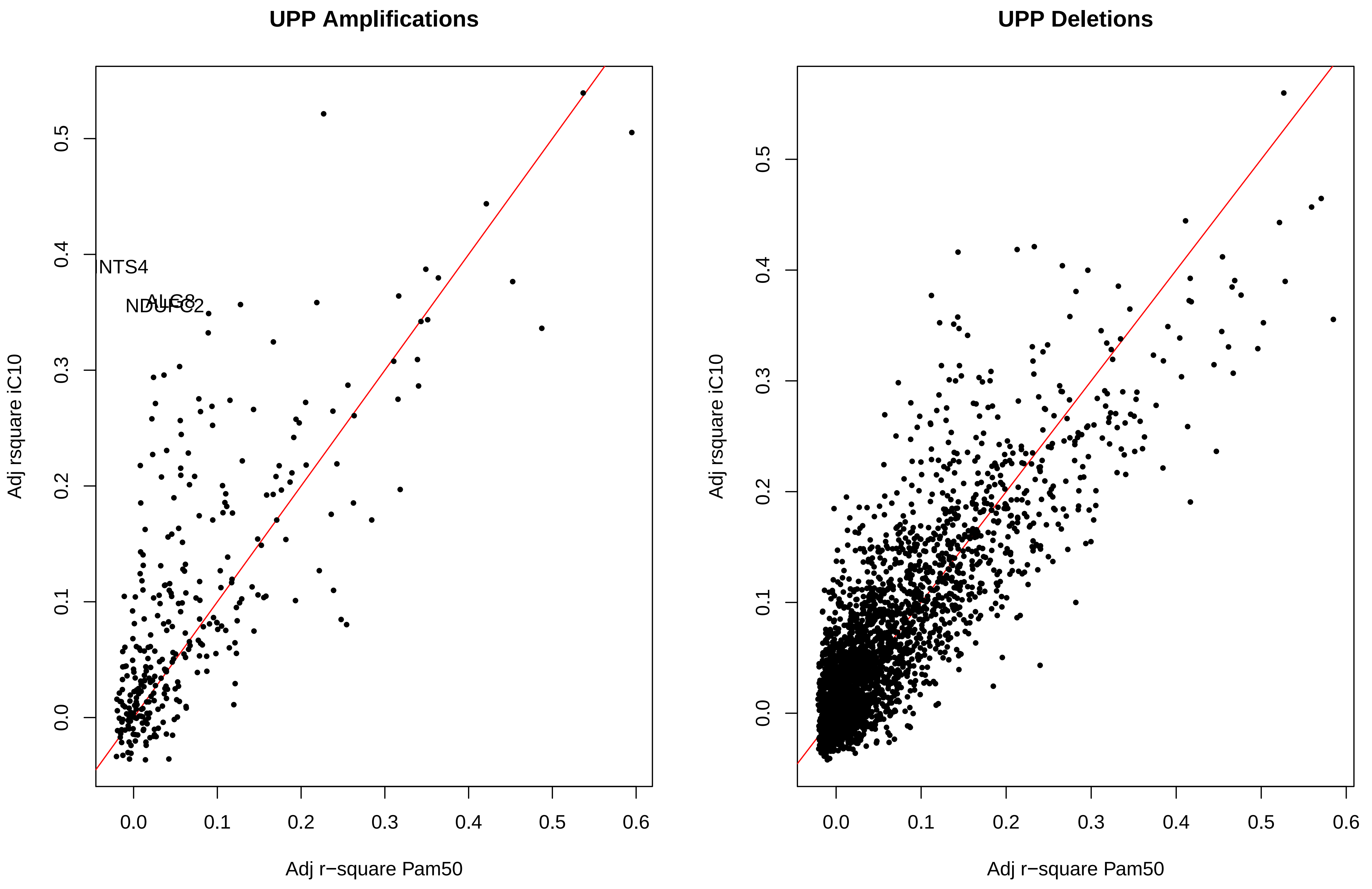
UPP Amplifications
Adj rsquare iC10
Adj r−square Pam50
INTS4
ALG8
NDUFC2
0.0
0.1
0.2
0.3
0.4
0.5
0.6
UPP Deletions
Adj rsquare iC10
Adj r−square Pam50

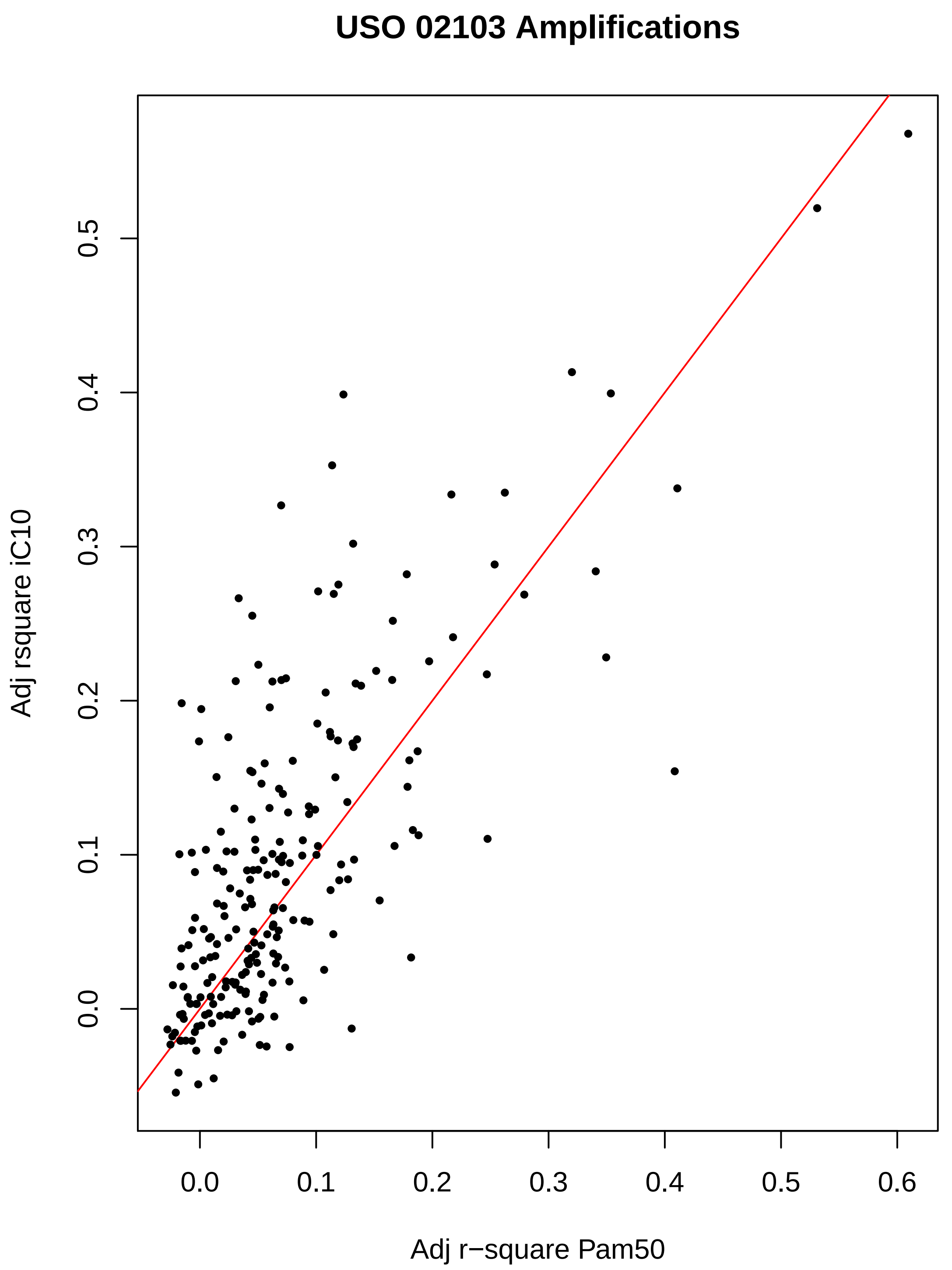
USO 02103 Amplifications
Adj rsquare iC10
Adj r−square Pam50
0.0
0.1
0.2
0.3
0.4
0.5
0.6

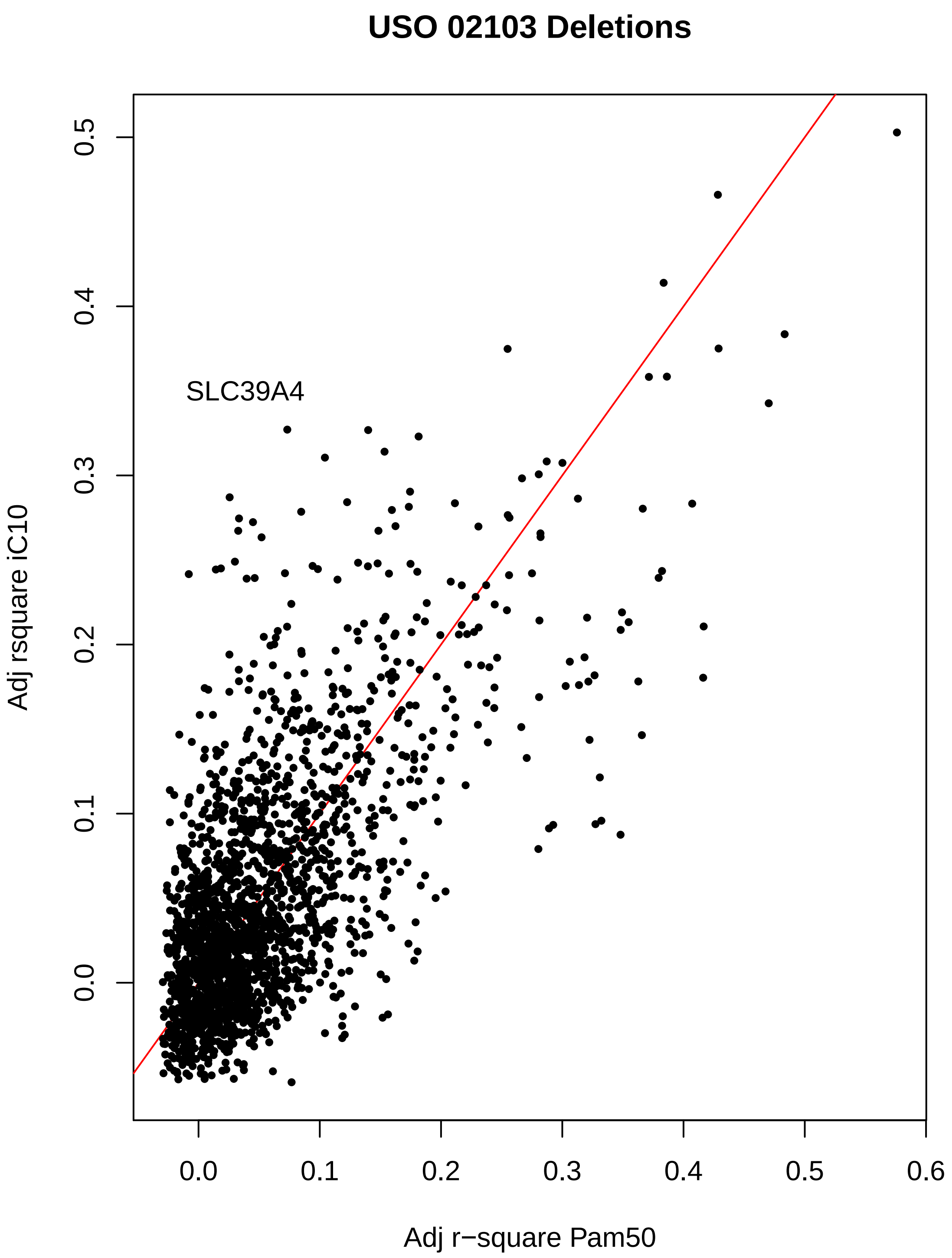
USO 02103 Deletions
SLC39A4
Adj rsquare iC10
Adj r−square Pam50
0.0
0.1
0.2
0.3
0.4
0.5
0.6

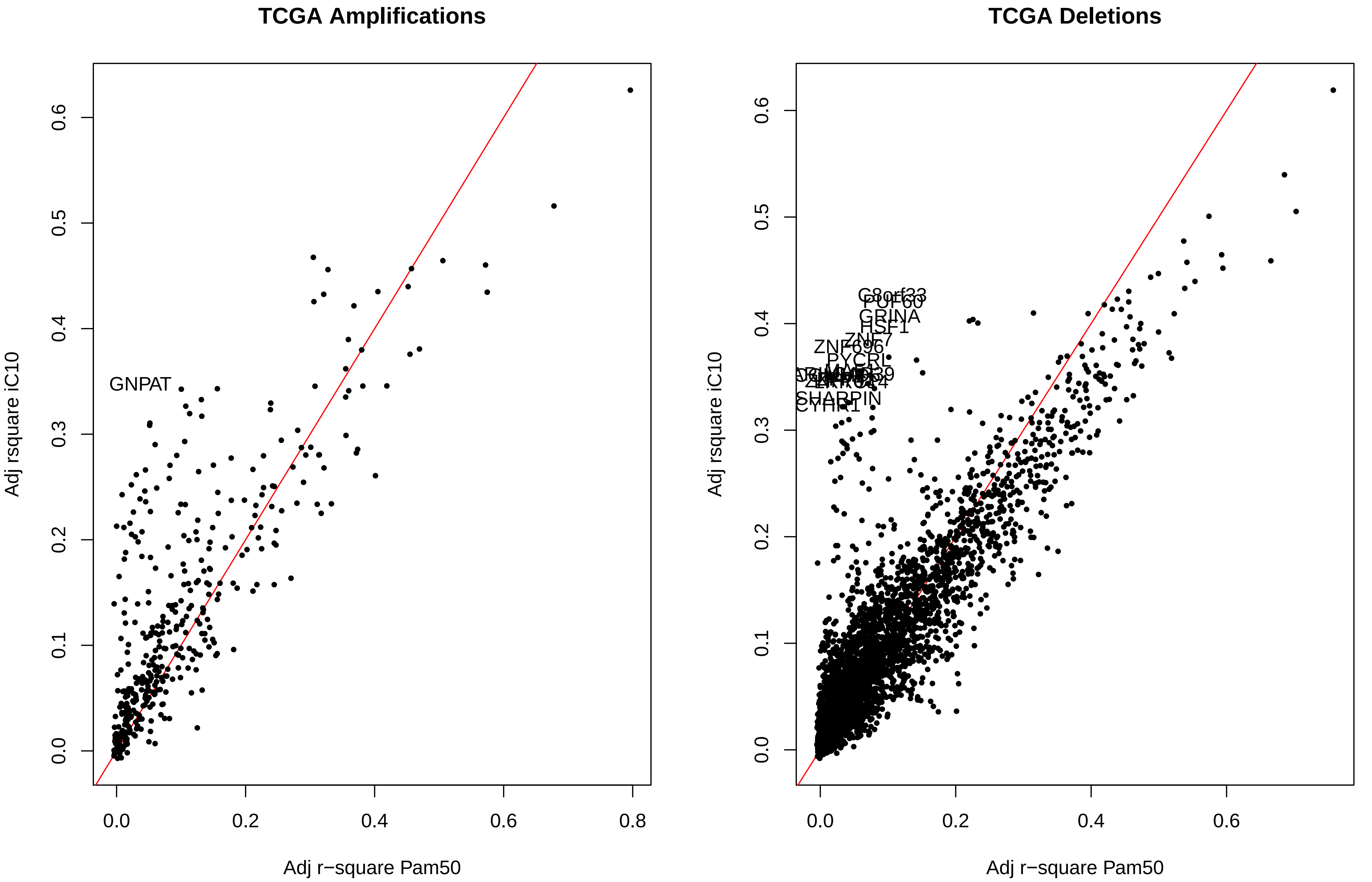
TCGA Amplifications
GNPAT
Adj rsquare iC10
Adj r−square Pam50
0.0
0.1
0.2
0.3
0.4
0.5
0.6
0.8
TCGA Deletions
C8orf33
PUF60
GRINA
HSF1
ZNF7
ZNF696
PYCRL
SHARPIN
CYHR1
Adj rsquare iC10
Adj r−square Pam50

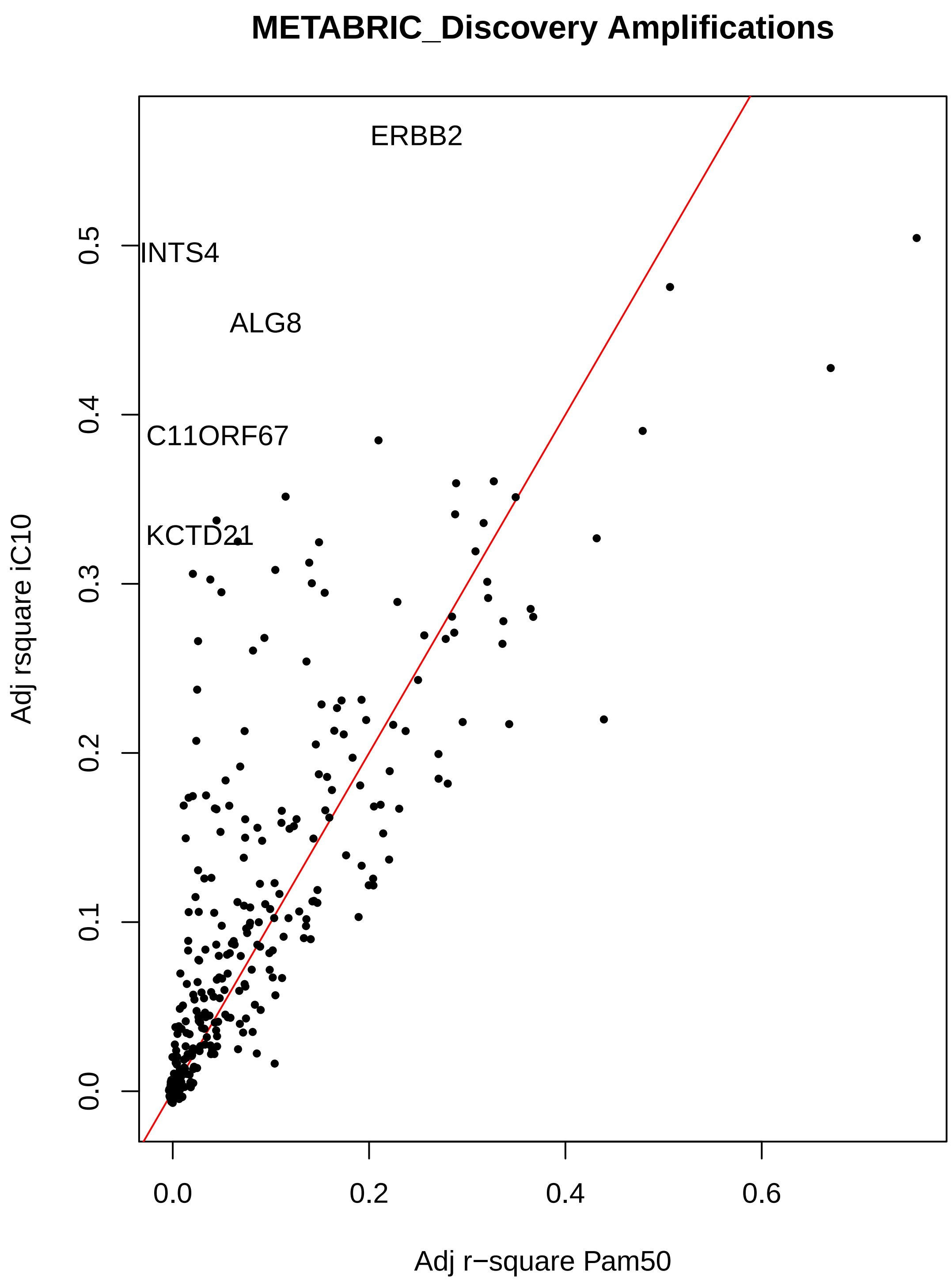

METABRIC_Discovery Amplifications
ERBB2
INTS4
ALG8
C11ORF67
KCTD21
Adj rsquare iC10
Adj r−square Pam50
0.0
0.1
0.2
0.3
0.4
0.5
0.6

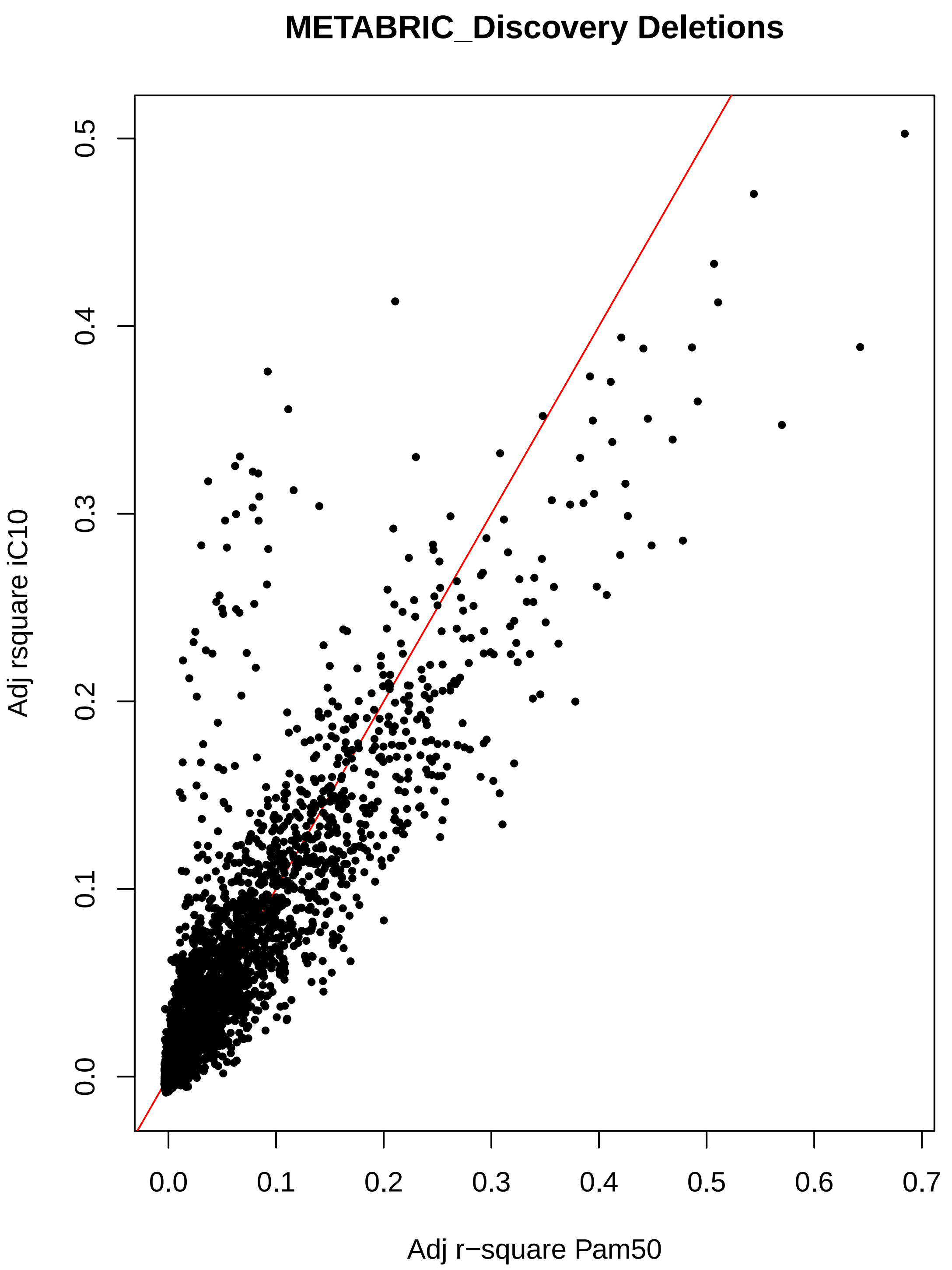

METABRIC_Discovery Deletions
Adj rsquare iC10
Adj r−square Pam50
0.0
0.1
0.2
0.3
0.4
0.5
0.6
0.7

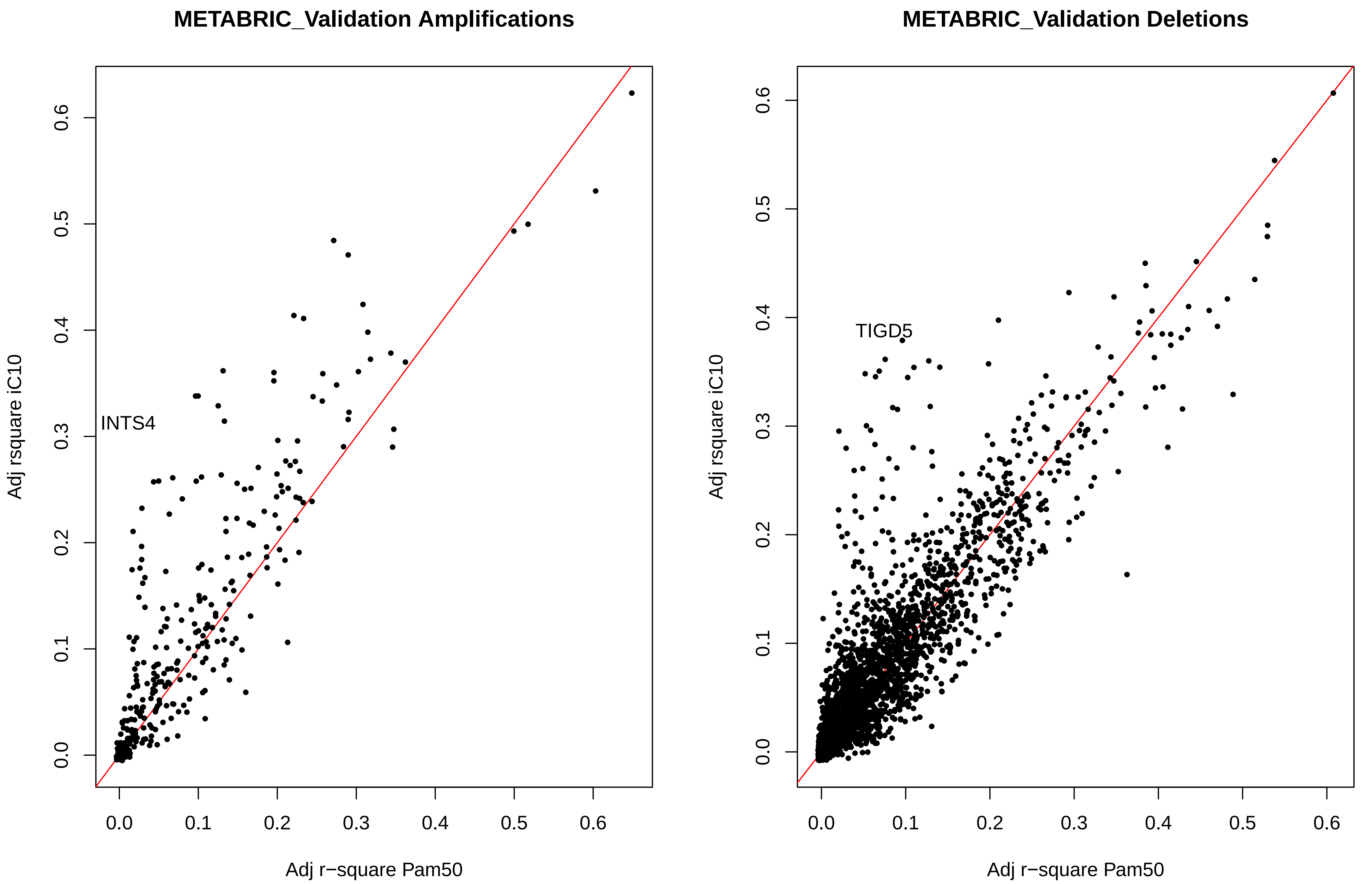
METABRIC_Validation Amplifications
Adj rsquare iC10
Adj r−square Pam50
INTS4
0.0
0.1
0.2
0.3
0.4
0.5
0.6
METABRIC_Validation Deletions
Adj rsquare iC10
Adj r−square Pam50
TIGD5

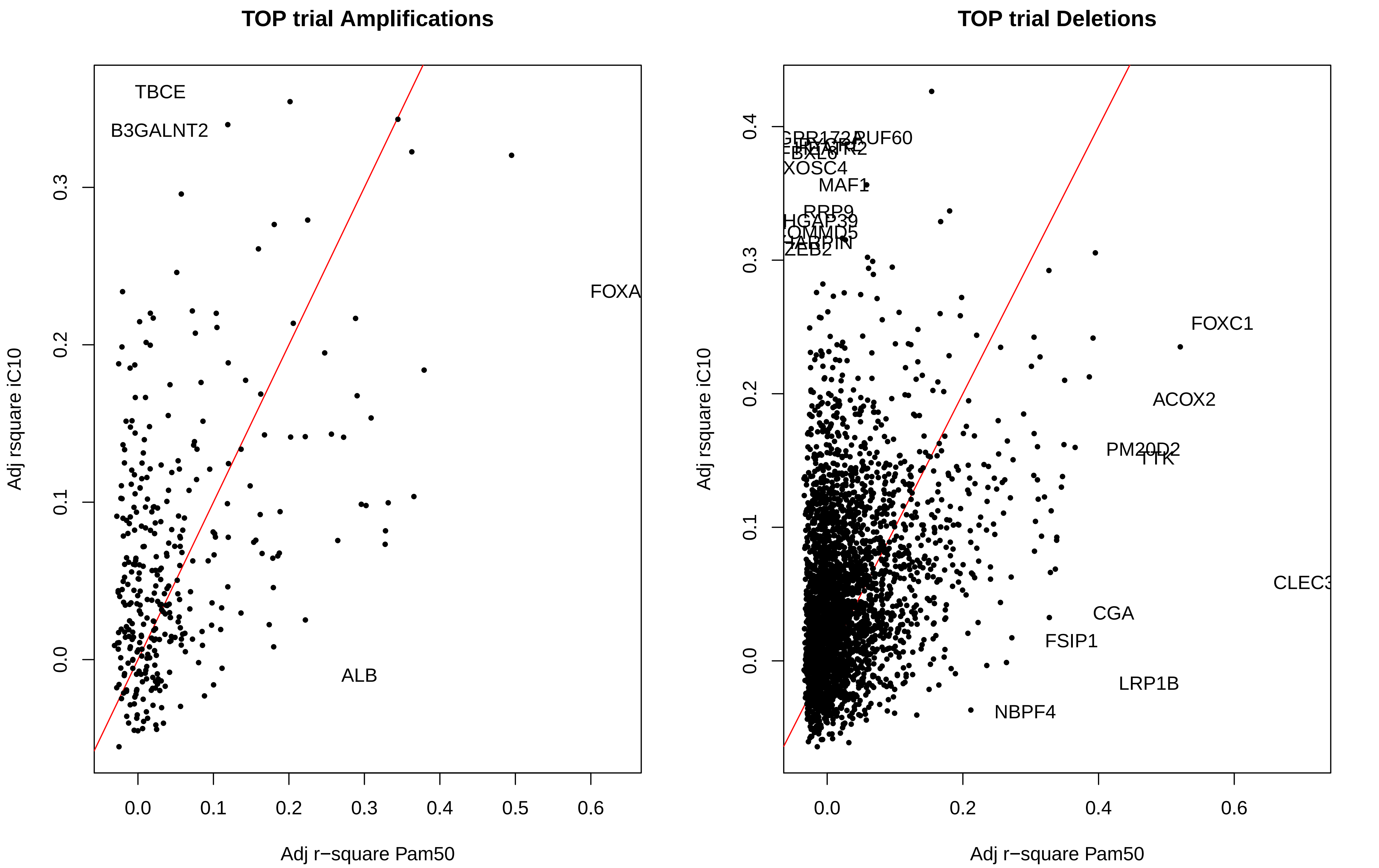
TOP trial Amplifications
Adj rsquare iC10
Adj r−square Pam50
TBCE
B3GALNT2
FOXA
ALB
0.0
0.1
0.2
0.3
0.4
0.5
0.6
TOP trial Deletions
Adj rsquare iC10
Adj r−square Pam50
XOSC4
MAF1
ZEB2
FOXC1
ACOX2
PM20D2
TTK
CLEC3
CGA
FSIP1
LRP1B
NBPF4

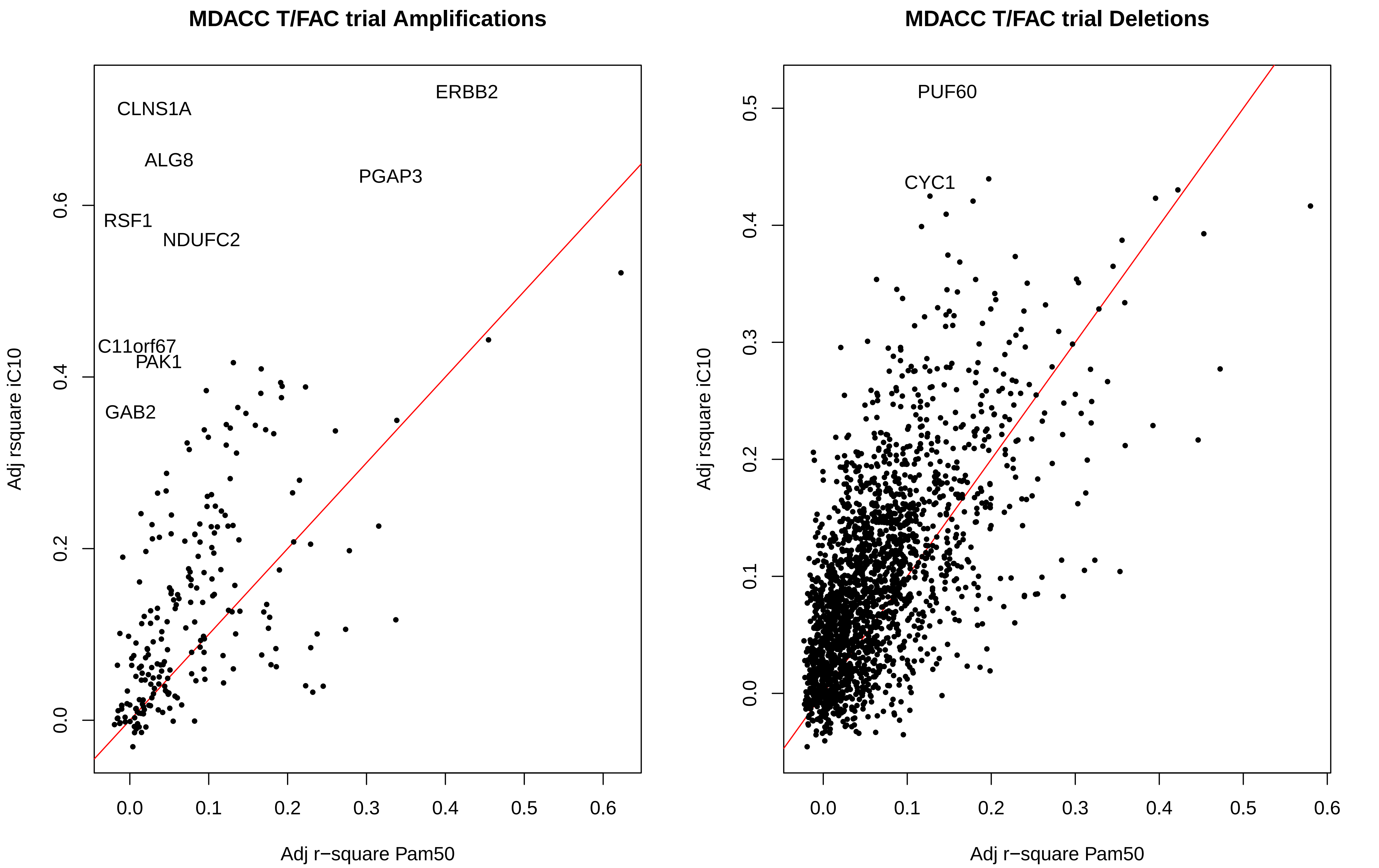
MDACC T/FAC trial Amplifications
ERBB2
CLNS1A
ALG8
PGAP3
RSF1
NDUFC2
C11orf67
PAK1
GAB2
Adj rsquare iC10
Adj r−square Pam50
0.0
0.2
0.4
0.6
0.0
0.1
0.2
0.3
0.4
0.5
0.6
MDACC T/FAC trial Deletions
PUF60
CYC1
Adj rsquare iC10
Adj r−square Pam50
0.0
0.1
0.2
0.3
0.4
0.5
0.0
0.1
0.2
0.3
0.4
0.5
0.6

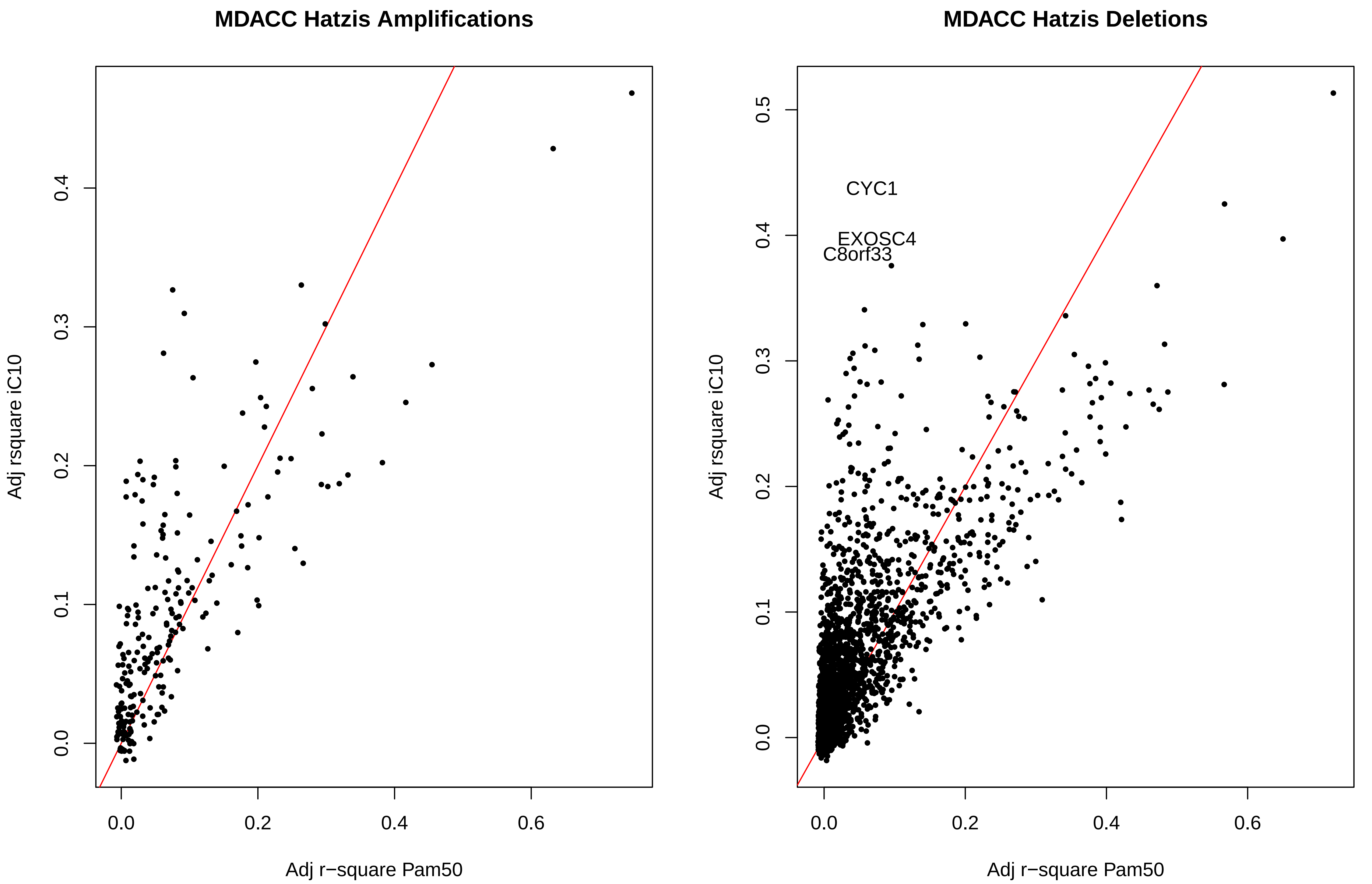
MDACC Hatzis Amplifications
MDACC Hatzis Deletions
Adj rsquare iC10
Adj r−square Pam50
0.0
0.1
0.2
0.3
0.4
0.5
0.6
CYC1
EXOSC4
C8orf33

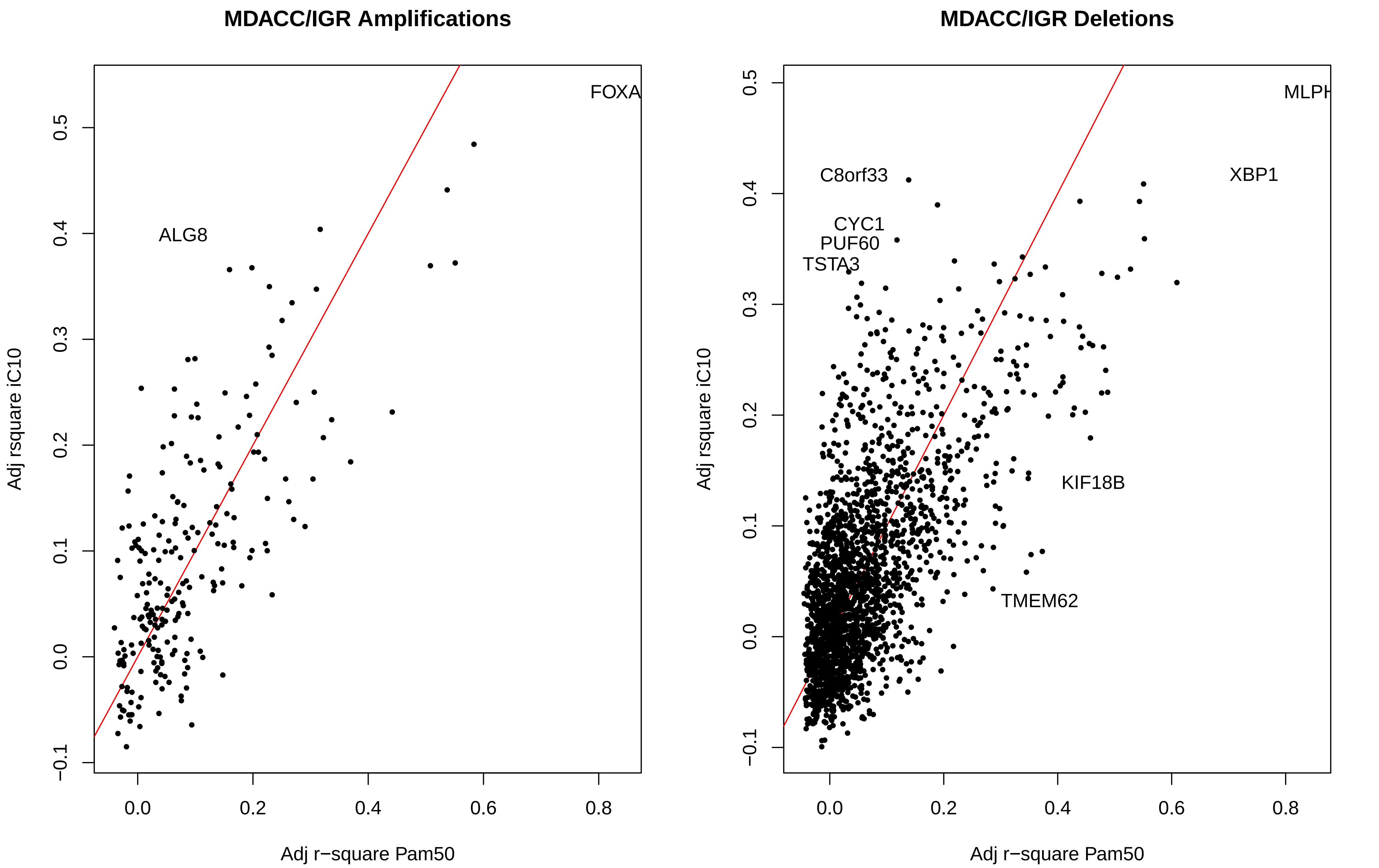
MDACC/IGR Amplifications
FOXA
ALG8
Adj rsquare iC10
Adj r−square Pam50
−0.1
0.0
0.1
0.2
0.3
0.4
0.5
0.0
0.2
0.4
0.6
0.8
MDACC/IGR Deletions
MLPH
XBP1
C8orf33
CYC1
PUF60
TSTA3
KIF18B
TMEM62
Adj rsquare iC10
Adj r−square Pam50
−0.1
0.0
0.1
0.2
0.3
0.4
0.5
0.0
0.2
0.4
0.6
0.8

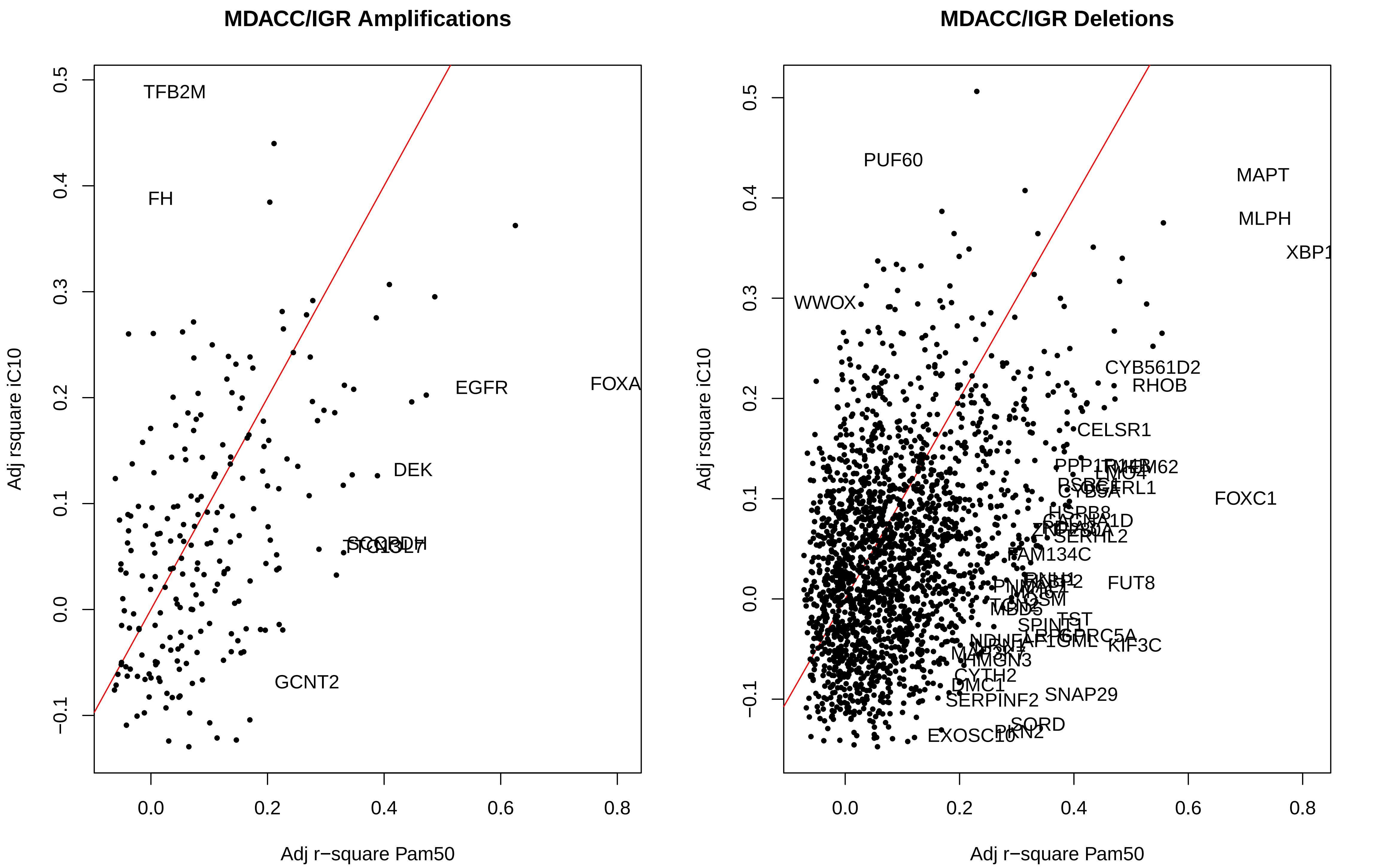

MDACC/IGR Amplifications
Adj rsquare iC10
Adj r−square Pam50
TFB2M
FH
EGFR
FOXA
DEK
GCNT2
MDACC/IGR Deletions
Adj rsquare iC10
Adj r−square Pam50
PUF60
MAPT
MLPH
XBP1
WWOX
CYB561D2
RHOB
CELSR1
FOXC1
SERHL2
FAM134C
FUT8
KIF3C
HMGN3
CYTH2
DMC1
SERPINF2
SNAP29
SORD
EXOSC10
